# Heritability of Psychological Traits and Developmental Milestones in Infancy: A Systematic Review and Meta-analysis

**DOI:** 10.1001/jamanetworkopen.2022.27887

**Published:** 2022-08-22

**Authors:** Chloe Austerberry, Maria Mateen, Pasco Fearon, Angelica Ronald

**Affiliations:** 1 Department of Clinical, Educational and Health Psychology, University College London, London, United Kingdom; 2 Centre for Family Research, University of Cambridge, Cambridge, United Kingdom; 3 Centre for Brain and Cognitive Development, Department of Psychological Sciences, Birkbeck, University of London, London, United Kingdom

## Abstract

**Importance:**

Although infancy is the most rapid period of postnatal growth and development, factors associated with variation in infant traits are not well understood.

**Objective:**

To synthesize the large twin study literature partitioning phenotypic variance in psychological traits and developmental milestones in infancy into estimates of heritability and shared and nonshared environment.

**Data Sources:**

PubMed, PsycINFO, and references of included publications were searched up to February 11, 2021.

**Study Selection:**

Peer-reviewed publications using the classical twin design to study psychological traits and developmental milestones from birth to 2 years old were included.

**Data Extraction and Synthesis:**

Data were extracted in line with Preferred Reporting Items for Systematic Reviews and Meta-Analyses and categorized using the *International Classification of Functioning, Disability and Health: Children and Youth Version*. Data were pooled in 3-level random effects models, incorporating within-cohort variance in outcome measurement and between-cohort variance. Data were analyzed from March 2021 through September 2021.

**Main Outcomes and Measures:**

The primary outcomes were monozygotic and dizygotic twin correlations. These were used to calculate genetic and shared and nonshared environment estimates.

**Results:**

Among 139 publications that were systematically retrieved, data were available on 79 044 twin pairs (31 053 monozygotic and 47 991 dizygotic pairs), 52 independent samples, and 21 countries. Meta-analyses were conducted on psychological traits and developmental milestones from 106 publications organized into 10 categories of functioning, disability, and health. Moderate to high genetic estimates for 8 categories were found, the highest of which was psychomotor functions (pooled *h^2^*, 0.59; 95% CI, 0.25-0.79; *P* < .001). Several categories of traits had substantial shared environment estimates, the highest being mental functions of language (pooled *c^2^*, 0.59; 95% CI, 0.24-0.86; *P* = .001). All examined categories of traits had moderate or high nonshared environment estimates, the highest of which were emotional functions (pooled *e^2^*, 0.42; 95% CI, 0.33-0.50; *P* < .001) and family relationships (pooled *e^2^*, 0.42; 95% CI, 0.30-0.55; *P* < .001).

**Conclusions and Relevance:**

These findings may be an important source of information to guide future gene discovery research, public perspectives on nature and nurture, and clinical insights into the degree to which family history and environments may estimate major domains of infant functioning, disability, and health in psychological traits and developmental milestones.

## Key Points

**Question:**

What are the overall genetic and shared and nonshared environment estimates for psychological traits and developmental milestones in infancy?

**Findings:**

In this systematic review and meta-analysis of 139 infant twin studies involving almost 80 000 twins globally, moderate to high genetic and shared and nonshared environment estimates were found across a range of important psychological traits and developmental milestones in infancy.

**Meaning:**

These results offer insight into the degree to which genes and environments estimate outcomes in key domains of infant functioning and suggest highly heritable traits that may be particularly suitable candidates for gene discovery.

## Teaser

This systematic review and meta-analysis investigates the correlation of genetic and shared and nonshared environment factors with infant developmental milestones.

## Introduction

Infancy represents the most rapid period of postnatal growth and development,^1^ and research suggests that it is a critical or sensitive period for a wide range of psychological and developmental milestones.^2,3,4^ Investment in early childhood is argued to be one of the most effective economic strategies through promoting long-term socioeconomic and health outcomes.^5^ Investment before age 2 years, in particular, appears to be associated with the greatest rate of return for investment.^5^ This is reflected in an increasing policy focus globally on the first thousand and one days from conception to age 2 years.^6^

Evidence suggests that complex traits are substantially but not entirely, heritable.^7^ Consequently, to gain understanding of the development of traits in infancy, it is important to draw on literature examining genetic and environmental factors associated with infant trait variation. The quantitative genetic method most widely and comprehensively performed in infancy is the classical twin design, which has been used for more than a century to partition phenotypic variance into additive genetic variance (heritability) and variance in the shared and nonshared environment. Family studies comparing biologically related siblings or parent-offspring pairs are typically unable to separate genetics from shared environment. In contrast, the classical twin design can provide separate estimates of heritability (the proportion of trait variation explained by genetic differences) and shared and nonshared environment. Twin studies are more feasible than adoption studies (which compare degree of resemblance between adoptees and their birth parents with resemblance between adoptees and their adoptive parents) to conduct at scale during infancy because adoption often occurs later in childhood. This has resulted in a far smaller and less comprehensive body of evidence in infancy from adoption studies than twin studies. The molecular genetic literature on infant traits is also small; the first genome-wide association study of infant traits was only recently conducted,^8^ and most molecular genetic studies in infancy have used candidate gene association methods, which in general have failed to yield replicable findings.^9^

A landmark meta-analysis,^7^ synthesizing virtually all twin studies of complex traits (predominantly psychiatric, metabolic, and cognitive traits) found a heritability of 49% across the lifespan when all traits and age groups were combined. The analysis combined data from infants and older children, calculating pooled estimates for children aged 0 to 11 years. Infancy is a rapid and sensitive period of development that deserves special focus. To address this, we conducted the first, to our knowledge, meta-analysis of twin studies of psychological and developmental functioning, disability, and health in infancy (birth to age 2 years), calculating pooled estimates of heritability and shared and nonshared environment.

## Methods

This study protocol was registered with PROSPERO (record number, CRD42019151532), and the systematic review and meta-analysis were performed in line with the Preferred Reporting Items for Systematic Reviews and Meta-analyses (PRISMA) reporting guideline 2020 statement and Meta-analysis of Observational Studies in Epidemiology (MOOSE) reporting guideline proposal for reporting. Given that the review involved the synthesis of anonymized information available in the public domain, it was exempt, according to University College London Research Ethics Committee (UCL REC) regulations, from requirements for ethics review by the UCL REC and the need for informed consent.

### Search Strategy

PubMed and PsycINFO databases were searched on November 30, 2018; February 5, 2020; and February 11, 2021, for twin studies (a genetically informed design described in the eMethods in the Supplement) of psychological traits and developmental milestones in infancy, using the search terms in eTable 1 in the Supplement. Search results were imported into EndNote software version 9 (Clarivate). C.A. reviewed duplicates identified by EndNote, deleting true duplicates, and screened titles and abstracts of identified records against inclusion and exclusion criteria (eTable 2 in the Supplement). Full texts were retrieved for nonexcluded records, and these, along with references of included publications, were screened by C.A. and M.M. Uncertainty about whether publications met inclusion criteria was resolved with senior researchers P.F. and A.R.

### Quality Assessment and Data Extraction

Included publications were rated by C.A. using an adaptation for twin studies of the Standard Quality Assessment Criteria for Evaluating Primary Research Papers from a Variety of Fields for Quantitative Studies^10^ (eMethods in the Supplement). Information was extracted from each included publication by C.A. and M.M. (eTable 3 in the Supplement). If publications reported overlapping data, the estimate with the larger sample size (or, if sample sizes were identical, the most recently published estimate) was retained for meta-analysis (eMethods in the Supplement).

### Classification of Phenotypes

Phenotypes were classified by C.A. using the World Health Organization *International Classification of Functioning, Disability and Health for Children and Youth* (*ICF-CY*).^11^ Uncertainty about which *ICF-CY* category to use for a phenotype was resolved through discussion with M.M. and senior researchers P.F. and A.R. Phenotypes were excluded from the meta-analysis if they could not be categorized or were in categories containing data from fewer than 5 independent samples.

### Statistical Analysis

In the R package metafor,^12^ we conducted two 3-level multilevel random-effects models (incorporating sampling variance, within-cohort variance in outcome measurements, and between-cohort variance) on twin correlations weighted by sample size from 10 categories of the *ICF-CY* containing data from 5 or more twin cohorts (eMethods in the Supplement). Zygosity was included as a moderator, with the dizygotic (DZ) group coded as the reference category in the first model to obtain a pooled monozygotic (MZ) twin correlation (MZ *r*) and standard error. The second model was identical but reparameterized with the MZ group as the reference category, producing a pooled DZ twin correlation (DZ *r*) and standard error. To allow for differences in variability in MZ and DZ subsets, models had a random error structure creating separate study-level and outcome error terms for MZ and DZ twins. As detailed in the eMethods in the Supplement, using pooled correlations and variances from the multilevel model, we calculated estimates for how much of the variation in each ICF-CY category was explained by additive genetic factors (A), the shared environment (C), and the nonshared environment (E, known collectively as ACE estimates) from standard univariate twin models estimated in the meta-analytic context using the R package metaSEM.^13^ We used 95% CIs around the pooled estimates in the twin study meta-analysis. Forest plots for analyses were produced using the R package metafor version 2.4-0 for R statistical software version 4.0.2 (R Project for Statistical Computing).^12^ We calculated *I^2^* for each of 3 levels in multilevel models. According to Cochrane guidelines, *I*^2^ ≤ 40% suggests low heterogeneity, while *I*^2^ = 30%-60% suggests moderate heterogeneity and *I*^2^ ≥ 50% indicates substantial or considerable heterogeneity.^14^ To reduce heterogeneity, analysis steps were repeated in 10 *ICF-CY* subcategories (with data from ≥5 samples) and 3 *ICF-CY* categories (with separate data from parents and observers from ≥5 samples) by parent and observer subgroup (for 6 meta-analyses in total) given that differences in rater have been found to be associated with differences in heritability estimates.^15,16^

We ran Egger tests of publication bias using the standard error as the estimator, and created funnel plots, plotting effect sizes against standard errors.^17^ In line with Cochrane recommendations, publication bias tests were run only on estimates in trait categories containing at least 10 estimates.^14^ Egger tests of publication bias were 2-sided and were considered significant at *P* < .05. Data analysis was conducted March through September 2021.

## Results

We identified 5047 publications (4675 publications in databases and 372 publications in references). After duplicate removal and screening, 139 publications were included,^18,19,20,21,22,23,24,25,26,27,28,29,30,31,32,33,34,35,36,37,38,39,40,41,42,43,44,45,46,47,48,49,50,51,52,53,54,55,56,57,58,59,60,61,62,63,64,65,66,67,68,69,70,71,72,73,74,75,76,77,78,79,80,81,82,83,84,85,86,87,88,89,90,91,92,93,94,95,96,97,98,99,100,101,102,103,104,105,106,107,108,109,110,111,112,113,114,115,116,117,118,119,120,121,122,123,124,125,126,127,128,129,130,131,132,133,134,135,136,137,138,139,140,141,142,143,144,145,146,147,148,149,150,151,152,153,154,155,156^ containing data on 79 044 twin pairs (31 053 MZ and 47 991 DZ twins), 52 twin cohorts, 21 countries, and 6 continents between 1972 and 2020. The sample included 66 407 twin pairs from Western, educated, industrialized, rich, and democratic countries (in Europe, North America, and Oceania; 84.01%) and 12 637 twin pairs from Africa, Asia, and South America (15.99%) (eResults, eFigures 2 and 3, and eTable 4 in the Supplement). We extracted 2279 estimates (twin correlations or ACE estimates, including 1097 estimates from MZ twins and 1182 estimates from DZ twins) on 377 phenotypes, organized into 17 categories and 28 subcategories of the *ICF-CY*. Data from 33 publications^22,24,28,31,32,38,39,47,51,54,56,57,58,60,65,66,69,71,80,86,99,103,105,107,114,116,119,136,143,149,150,151,152^ included in the systematic review were excluded from the meta-analysis. Detailed information on search results, phenotype categorization, and excluded data is provided in eResults, eTables 1 and 4 through 6, and eFigures 1 through 3 in the Supplement.

### Meta-analysis Results

#### Analysis of Phenotypes by Category

Among 10 categories of infant psychological and developmental functioning, disability and health displayed in Table 1 and defined in the *ICF-CY*,^11^ there were enough data from independent samples for meta-analysis (≥5 samples). Results are reported in Table 2 and the Figure. Forest plots for these meta-analyses are reported in eFigures 4 through 13 in the Supplement. More twin samples used in these meta-analyses contained parent-reported data (cohort *k* = 22) than observer-rated data (cohort *k* = 12) (eResults and eTable 7 in the Supplement).

**Table 1.** Examples of Phenotypes by Category

*ICF-CY* category	Example phenotypes
b134 Sleep functions	Nocturnal sleep duration
	Night awakenings
	Sleep problems
b140 Attention functions	Attention problems
	Task orientation
	Spectral amplitude during visual attention
b147 Psychomotor functions	Activity level
	Fine motor
	Sitting without support
b152 Emotional functions	Resistance to soothing
	Fearfulness
	Positive affect
b163 Basic cognitive functions	General cognitive ability
	Nonverbal cognitive development
	Primary cognition
b167 Mental functions of language	Reception of language
	Expressive vocabulary
	Late language acquisition
b560 Growth maintenance functions	BMI
	Head circumference
	Weight gain
d710 Basic interpersonal interactions	Disregard for others
	Reciprocal social behavior
	Shyness
d720 Complex interpersonal interactions	Disruptive behavior
	Peer aggression
	Disregard for rules
d720 Family relationships	Attachment security
	Dependence
	Separation distress

Abbreviations: BMI, body mass index (calculated as weight in kilograms divided by height in meters squared); *ICF-CY*, *International Classification of Functioning, Disability and Health for Children and Youth*.

**Table 2.** Multilevel Random Effects Models of Phenotypic Categories

*ICF-CY* category^b^	*k* Cohort^c^	*k* Estimates^d^	MZ twin pairs, No.	DZ twin pairs, No.	Pooled MZ *r* (95% CI)^e^	Pooled DZ *r* (95% CI)^f^	Pooled *h^2^* (95% CI)^g^	*P* value	Pooled *c^2^* (95% CI)^h^	*P* value	Pooled *e^2^* (95% CI)^i^	*P* value	*I^2^* ^a^		
													Level 1^j^	Level 2^k^	Level 3^l^
b134 Sleep functions	7	49	1923	4044	0.80 (0.67-0.93)	0.63 (0.49-0.76)	0.35 (0-0.73)	.06	0.45 (0.16-0.74)	.002	0.20 (0.07-0.33)	.003	0.29	25.69	74.02
b140 Attention functions	10	175	3011	6137	0.60 (0.49-0.71)	0.36 (0.25-0.47)	0.48 (0.17-0.71)	.002	0.12 (0-0.37)	.33	0.40 (0.29-0.51)	<.001	12.44	30.26	57.29
b147 Psychomotor functions	13	151	3109	6105	0.67 (0.55-0.79)	0.37 (0.25-0.49)	0.59 (0.25-0.79)	.001	0.07 (0-0.35)	.60	0.33 (0.22-0.45)	<.001	1.03	23.69	75.28
b152 Emotional functions	14	216	1756	3633	0.58 (0.50-0.66)	0.38 (0.30-0.46)	0.40 (0.16-0.64)	.001	0.18 (0-0.38)	.06	0.42 (0.34-0.50)	<.001	6.48	68.79	24.73
b163 Basic cognitive functions	5	47	2636	5371	0.79 (0.68-0.89)	0.62 (0.51-0.73)	0.34 (0.04-0.64)	.03	0.45 (0.21-0.69)	<.001	0.21 (0.11-0.32)	<.001	1.41	42.04	56.56
b167 Language	5	96	2232	2853	0.82 (0.67-0.98)	0.71 (0.55-0.86)	0.24 (0-0.68)	.28	0.59 (0.24-0.86)	.001	0.18 (0.02-0.33)	.02	0.19	69.93	29.88
b560 Growth	24	465	16653	21874	0.80 (0.76-0.83)	0.63 (0.59-0.67)	0.34 (0.23-0.45)	<.001	0.46 (0.37-0.54)	<.001	0.20 (0.17-0.24)	<.001	2.67	58.59	38.74
d710 Basic interpersonal functions	18	356	4207	8037	0.59 (0.48-0.70)	0.40 (0.29-0.51)	0.38 (0.05-0.70)	.02	0.21 (0-0.48)	.10	0.41 (0.30-0.52)	<.001	1.62	23.59	74.79
d720 Complex interpersonal functions	10	73	3244	5117	0.72 (0.61-0.82)	0.49 (0.39-0.60)	0.44 (0.15-0.75)	.003	0.27 (0.04-0.51)	.02	0.29 (0.18-0.39)	<.001	1.98	40.08	57.93
d760 Family relationships	7	29	678	1546	0.58 (0.45-0.71)	0.37 (0.24-0.50)	0.41 (0.06-0.71)	.02	0.17 (0-0.45)	.24	0.42 (0.30-0.55)	<.001	3.12	39.60	57.28

Abbreviations: DZ, dizygotic; *ICF-CY*, *International Classification of Functioning, Disability and Health, Children and Youth Version*; MZ, monozygotic.

^a^ Heterogeneity.

^b^ Definitions for categories and subcategories can be found in cited *ICF-CY* manual.^11^

^c^ Number of independent twin cohorts.

^d^ Number of estimates (twin correlations).

^e^ MZ twin correlation.

^f^ DZ twin correlation.

^g^ Heritability.

^h^ Shared environment.

^i^ Nonshared environment.

^j^ Sampling variance.

^k^ Within-cohort variance in outcome measurement.

^l^ Between-cohort variance.

**Figure.**
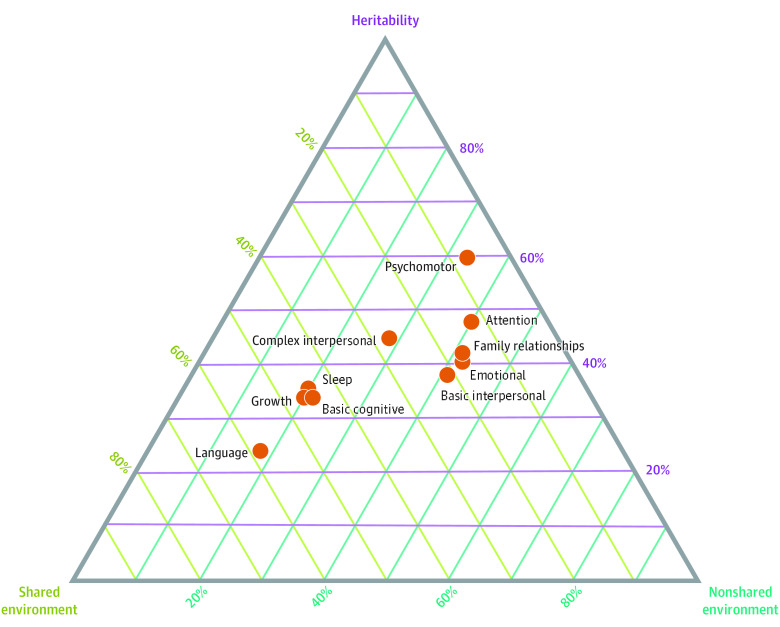
Ternary Plot of Pooled Heritability and Shared and Nonshared Environment Estimates by Phenotypic Category

##### Heritability

The highest heritability estimate was for psychomotor functions (pooled *h^2^*, 0.59; 95% CI, 0.25-0.79; *P* < .001), followed by attention functions (pooled *h^2^*, 0.48; 95% CI, 0.170.71; *P* = .002), complex interpersonal interactions (pooled *h^2^*, 0.44; 95% CI, 0.15-0.75; *P* = .003), family relationships (pooled *h^2^*, 0.41; 95% CI, 0.06-0.71; *P* = .02), and emotional functions (pooled *h^2^*, 0.40; 95% CI, 0.16-0.64; *P* = .001). Remaining categories had lower estimates with 95% CIs above 0 (pooled *h^2^* range, 0.24-0.38), apart from mental functions of language and sleep functions, which had CIs overlapping 0 (pooled *h^2^*, 0.24 and 0.35, respectively) (Table 2).

##### Shared Environment

Mental functions of language (pooled *c^2^*, 0.59; 95% CI, 0.24-0.86; *P* = .001), growth maintenance (pooled *c^2^*, 0.46; 95% CI, 0.37-0.54; *P* < .001), basic cognitive functions (pooled *c^2^*, 0.45; 95% CI, 0.21-0.69; *P* < .001), and sleep functions (pooled *c^2^*, 0.45; 95% CI, 0.16-0.74; *P* = .002) had high shared environment estimates. Complex interpersonal interactions had a lower estimate (pooled *c^2^*, 0.27; 95% CI, 0.04-0.51; *P* = .02), and estimates for psychomotor, attention, and emotional functions; family relationships; and basic interpersonal interactions had CIs overlapping with 0 (pooled *c^2^* range, 0.07-0.21) (Table 2).

##### Nonshared Environment

Categories with the highest nonshared environment estimates were emotional functions (pooled *e^2^*, 0.42; 95% CI, 0.33-0.50; *P* < .001), family relationships (pooled *e^2^*, 0.42; 95% CI, 0.30-0.55; *P* < .001), basic interpersonal interactions (pooled *e^2^*, 0.41; 95% CI, 0.30-0.52; *P* < .001), and attention functions (pooled *e^2^*, 0.40; 95% CI, 0.29-0.51; *P* < .001). Remaining categories had lower estimates with CIs above 0 (pooled *e^2^* range, 0.18-0.33) (Table 2).

##### Heterogeneity

Sampling variance contributed little to the total variance of each phenotypic category (level 1 *I^2^* range, 0.19%-12.44%) (Table 2). Within-cohort heterogeneity (ie, differences across measures within a domain and within a cohort) contributed substantially to total variance in mental functions of language, emotional functions, and growth functions (level 2 *I^2^* range, 58.59%-69.93%), and between-cohort heterogeneity contributed a lower amount (level 3 *I^2^* range, 24.73%-38.74%) to these outcomes. The remaining 7 categories each had substantial between-cohort heterogeneity (level 3 *I^2^* range, 56.56%-75.28%) and lower within-cohort heterogeneity (level 2 *I^2^* range, 23.59%-42.04%) (Table 2).

#### Analysis of Phenotypes by Subcategory and Rater

To reduce heterogeneity, we analyzed 10 subcategories of the *ICF-CY* (with data from ≥5 samples) and 3 phenotypic categories (with separate parent and observer data from ≥5 samples) by rater (for 6 subgroups: 3 with parent report and 3 with observer report). Full findings are reported in eResults and eTables 8 and 9 in the Supplement. Parent-rated phenotypes in the 3 examined categories (psychomotor and emotional functions and basic interpersonal interactions) had higher heritability and lower nonshared estimates than observer ratings and comparable shared environment estimates.

### Publication Bias

Possible publication bias was detected in the unexpected direction across all categories. Findings are in eResults, eTables 10 to 11, and eFigures 14 to 18 in the Supplement.

### Quality Assessment

Quality assessment results are displayed in eFigure 19 in the Supplement. The mean (SD) score for the 106 publications^18,19,20,21,23,25,26,27,29,30,33,34,35,36,37,40,41,42,43,44,45,46,48,49,50,52,53,55,57,59,61,62,63,64,67,68,70,72,73,74,75,76,77,78,79,81,82,83,84,85,87,88,89,90,91,92,93,94,95,96,97,98,100,101,102,104,106,108,109,110,111,112,113,115,117,118,120,121,122,123,124,125,126,127,128,129,130,131,132,133,134,135,137,138,139,140,141,142,144,145,146,147,148,153,154,155,156^ included in the meta-analysis was 75.58% (13.83%).

## Discussion

Drawing on a systematically-retrieved pooled sample of 79 044 twins, this systematic review and meta-analysis found evidence that most domains of functioning, disability, and health in psychological and developmental milestones were heritable in infancy and had moderate to high nonshared estimates. Contrary to evidence in older ages,^7^ shared environment estimates were high across several important domains of infant development.

### Heritability

Consistent with evidence in older samples,^7^ all meta-analyzed categories had heritability estimates with 95% CIs above 0 in infancy, apart from sleep and language functions. Estimates were high (≥40%) for important areas of development (psychomotor, attention, and emotional functions; family relationships [attachment and dependency]; and complex interpersonal interactions [behavioral problems]), suggesting that these categories may be particularly suitable candidates for gene mapping.

High heritability in infancy of attention functions was consistent with the high heritability of attention-deficit/hyperactivity disorder (ADHD) and ADHD traits in older samples.^157^ In accordance with the very high heritability of autism,^158^ a neurodevelopmental condition involving differences in social interaction, social cues in relationships, and regulating behaviors within interactions were among the most heritable subcategories. Absence of evidence that infant language was heritable was consistent with evidence that the heritability of cognition, including language, is low in early development, increasing with age.^159,160^

The higher heritability of parent-rated than observer-rated phenotypes may be driven by contrast bias in parental reports of phenotypes among children who were DZ twins, exaggerating DZ differences, or by assimilation bias in parental reports of MZ twins, exaggerating MZ similarity.^161^ Correlated rater bias that inflated MZ and DZ twin similarity equally would lead to inflated shared environment estimates. Without raw data from individual studies, it was not possible to test this by examining variance-covariance structures, which can uncover evidence of contrast and assimilation bias. Overall, our results suggest that individual differences in growth, motor, cognitive, and emotional development may be associated with genetic factors as early as the first 2 years of life.

### Shared Environment

Contrary to evidence in older age groups,^7^ shared environment estimates had CIs above 0 in several domains and were high for language, sleep, growth maintenance, and basic cognitive functions, reflecting a broader trend noted in the literature that shared environment estimates for language and cognition are higher in early development.^159,160^ This may have important implications for obesity prevention and efforts to promote intellectual outcomes, which are among the most robust estimators of health and longevity.^162^ Shared environment estimates had CIs overlapping with 0 for psychomotor, attention, and emotional functions; basic interpersonal interactions; and family relationships. This was consistent with pooled findings in older age groups^7^ and evidence that shared environments do not contribute as much to similarity between siblings as genetics and do not contribute as much to differences between siblings as nonshared environments.^163,164^

### Nonshared Environment

Nonshared environment estimates had 95% CIs above 0 for all phenotypic categories and were high for emotional and attention functions, family relationships, and basic interpersonal interactions. Higher nonshared estimates for observer ratings than parent ratings were consistent with wider research^165^ and may reflect the importance of each twin’s unique experience in the expression of phenotypes specifically when rated by observers. Alternatively, given that nonshared estimates also include measurement error, higher observer-rated estimates may reflect increased error in observational measurement.

### Limitations

This study has several limitations. Given that research designs all have limitations and biases, establishing robust evidence ideally involves triangulation of methods. However, the classical twin design is currently the only quantitative genetic method that has produced data from enough independent samples to conduct adequately powered meta-analyses across a comprehensive range of infant traits. The generalizability of twin findings may be limited because some infant phenotypes (eg, language and birth weight) develop differently in twins compared with individuals from singleton births.^166,167^ However, given that our aim was to examine individual differences rather than investigate how and why groups differed, mean differences between twins and singletons may not indicate issues with generalizability.

Although the twin method can be used to examine genotype-environment correlation or interaction, we did not synthesize findings on these outcomes. In twin modeling, ignored interaction between genotype and shared environment is estimated as heritability and ignored interaction between genotype and nonshared environment will be estimated as nonshared environment, potentially contributing to biased estimates.^168^

CIs for some estimates were wide. In meta-analysis, CIs depend on the precision of included studies, which are influenced by sample size and, in the case of twin modeling, the ratio of MZ to DZ pairs and relative contribution of each parameter.^169^ Furthermore, for any given sample size, there is more power to estimate *e^2^* than *h^2^* and *c^2^*, which may explain the narrower intervals around *e^2^*. CIs are also associated with the number of samples included in a meta-analysis; while adding studies may improve precision, it may also increase heterogeneity, decreasing precision. Crucially, in multilevel modeling, CIs are also dependent on the degree of between-study heterogeneity. High variability in estimates across studies is associated with wider CIs, and ignoring such heterogeneity tends to overestimate precision. The heterogeneity we observed was generally high, and so CIs were comparatively wide.

A downside of the comprehensive approach we took is that it may have increased between-study heterogeneity. We attempted to reduce this in the narrower subcategory and rater analyses. However, between-study heterogeneity was substantial in all categories and subcategories, suggesting that between-study differences likely created considerable noise in our estimates. Understanding and reducing heterogeneity will be important for future research to provide more precise twin estimates in infancy. Possible publication bias was also detected across multiple outcome domains. The impact of this on the estimates is difficult to rigorously assess.

Although individuals from Western, educated, industrialized, rich, and democratic societies represent 12% of the world’s population, twins from these areas of the world constituted more than 80% of our sample. Infants in Africa, Asia, and South America combined represented approximately 16% of our sample, highlighting a need for twin research on infants in these continents.

There was an imbalance in the amount of research conducted on the synthesized categories; for example, far more was conducted on anthropometric phenotypes included in growth maintenance functions (which included data from 24 of 52 included samples) than other domains. Important areas in which research was lacking included nonsocial autistic traits and dysregulation, eating behavior, memory, higher-level cognitive functions, and brain structure.

## Conclusions

To our knowledge, this systematic review and meta-analysis is the first study to synthesize the large and comprehensive infant twin literature on psychological traits and developmental milestones, offering insight into the possible earliest manifestations of phenotypic variance associated with genetic and environmental factors. This has the potential to improve public perceptions on nature and nurture by, for example, dispelling widely held beliefs that infants are shaped entirely by their environments or that family history entirely predetermines child health, beliefs that may place undue pressure on parents. For researchers, these results may offer a guide for future gene discovery research and efforts to uncover the causes of variation in infant traits. For clinicians, these findings may provide an indication of how family history and environmental conditions may estimate infant outcomes, including outcomes that may be early markers associated with subsequent healthy or pathological development.

## Notes

Supplement.
eMethods.

eResults.
**eTable 1.** Database and Reference List Searches **eTable 2.** Inclusion and Exclusion Criteria **eTable 3.** Information Extracted From Included Publications **eTable 4.** Publications Identified in Systematic Literature Search, Presented Alphabetically by First Author **eTable 5.** Twin Studies Identified in Systematic Literature Search, Presented Alphabetically by Study Name **eTable 6.** Phenotypes Identified in Systematic Literature Search Coded Using Classification System **eTable 7.** Estimates and Cohorts in Phenotypic Categories by Rater **eTable 8.** Multilevel Random Effects Models of Phenotypic Subcategories **eTable 9.** Multilevel Random Effects Models of Parent and Observer Ratings of 3 Phenotypic Categories **eTable 10.** Tests for Publication Bias on Twin Correlations by Phenotype Category **eTable 11.** Tests for Publication Bias on Phenotype Categories by Variance Component in Phenotype Categories With ≥10 Estimates **eFigure 1.** Prisma Flow Diagram **eFigure 2.** Bar Chart of Number of Twin Pairs by Country **eFigure 3.** Bar Chart of Number of Twin Pairs by Continent **eFigure 4.** Sleep Functions Forest Plot **eFigure 5.** Attention Functions Forest Plot **eFigure 6.** Psychomotor Functions Forest Plot **eFigure 7.** Emotional Functions Forest Plot **eFigure 8.** Basic Cognitive Functions Forest Plot **eFigure 9.** Mental Functions of Language Forest Plot **eFigure 10.** Growth Maintenance Functions Forest Plot **eFigure 11.** Basic Interpersonal Interactions Forest Plot **eFigure 12.** Complex Interpersonal Interactions Forest Plot **eFigure 13.** Family Relationships Forest Plot **eFigure 14.** Funnel Plots of Association Between Monozygotic Twin Correlation and Standard Error in Phenotype Categories With ≥10 Estimates **eFigure 15.** Funnel Plots of Association Between Dizygotic Twin Correlation and Standard Error in Phenotype Categories With ≥10 Estimates **eFigure 16.** Funnel Plots of Association Between Heritability and Standard Error in Phenotype Categories With ≥10 Estimates **eFigure 17.** Funnel Plots of Association Between Shared Environment and Standard Error in Phenotype Categories With ≥10 Estimates **eFigure 18.** Funnel Plots of Association Between Nonshared Environment and Standard Error in Phenotype Categories With ≥10 Estimates **eFigure 19.** Bar Plot of Quality Assessment Scores **eFigure 20.** Sustaining Attention Forest Plot **eFigure 21.** Psychomotor Control Forest Plot **eFigure 22.** Organization of Psychomotor Functions Forest Plot **eFigure 23.** Regulation of Emotion Forest Plot **eFigure 24.** Range of Emotion Forest Plot **eFigure 25.** Expression of Language Forest Plot **eFigure 26.** Respect and Warmth in Relationships Forest Plot **eFigure 27.** Social Cues in Relationships Forest Plot **eFigure 28.** Regulating Behaviors Within Interactions Forest Plot **eFigure 29.** Interacting According to Social Rules Forest Plot **eFigure 30.** Psychomotor Functions (Observer Report) Forest Plot **eFigure 31.** Psychomotor Functions (Parent Report) Forest Plot **eFigure 32.** Emotional Functions (Observer Report) Forest Plot **eFigure 33.** Emotional Functions (Parent Report) Forest Plot **eFigure 34.** Basic Interpersonal Interactions (Observer Report) Forest Plot **eFigure 35.** Basic Interpersonal Interactions (Parent Report) Forest Plot
eReferences.

## References

[1] Lejarraga H. Growth in infancy and childhood: A pediatric approach. In: Cameron N, Bogin B, eds. Human Growth and Development. 2nd ed. Academic Press; 2012:23-56. doi:10.1016/B978-0-12-383882-7.00002-7

[2] Kumsta R, Kreppner J, Kennedy M, Knights N, Rutter M, Sonuga-Barke E. Psychological consequences of early global deprivation: an overview of findings from the English & Romanian Adoptees study. Eur Psychol. 2015;20(2):138-151. doi:10.1027/1016-9040/a000227

[3] Rutter M; English and Romanian Adoptees (ERA) Study Team. Developmental catch-up, and deficit, following adoption after severe global early privation. J Child Psychol Psychiatry. 1998;39(4):465-476. doi:10.1017/S0021963098002236 9599775

[4] Nelson CA III, Zeanah CH, Fox NA. How early experience shapes human development: the case of psychosocial deprivation. Neural Plast. 2019;2019:1676285. doi:10.1155/2019/1676285 30774652 PMC6350537

[5] Heckman JJ. Schools, skills, and synapses. Econ Inq. 2008;46(3):289. doi:10.1111/j.1465-7295.2008.00163.x 20119503 PMC2812935

[6] Darling JC, Bamidis PD, Burberry J, Rudolf MCJ. The First thousand days: early, integrated and evidence-based approaches to improving child health: coming to a population near you? Arch Dis Child. 2020;105(9):837-841. doi:10.1136/archdischild-2019-316929 32111596

[7] Polderman TJ, Benyamin B, de Leeuw CA, . Meta-analysis of the heritability of human traits based on fifty years of twin studies. Nat Genet. 2015;47(7):702-709. doi:10.1038/ng.3285 25985137

[8] Pappa I, Szekely E, Mileva-Seitz VR, . Beyond the usual suspects: a multidimensional genetic exploration of infant attachment disorganization and security. Attach Hum Dev. 2015;17(3):288-301. doi:10.1080/14616734.2015.1037316 25939396

[9] Papageorgiou KA, Ronald A. The genetic basis of psychological traits in infancy: implications for understanding the causes of developmental psychopathology. In: Centifanti LC, Williams DM, eds. The Wiley Handbook of Developmental Psychopathology. John Wiley & Sons; 2017:235-258. doi:10.1002/9781118554470.ch11

[10] Kmet LM, Lee RC, Cook LS. Standard Quality Assessment Criteria for Evaluating Primary Research Papers from a Variety of Fields. Alberta Heritage Foundation for Medical Research; 2004. Accessed July 18, 2022. https://www.ihe.ca/advanced-search/standard-quality-assessment-criteria-for-evaluating-primary-research-papers-from-a-variety-of-fields

[11] World Health Organization. International Classification of Functioning, Disability, and Health: Children & Youth Version: ICF-CY. World Health Organization; 2007. Accessed July 18, 2022. https://apps.who.int/iris/handle/10665/43737

[12] Viechtbauer W. Conducting meta-analyses in R with the metafor package. J Stat Softw. 2010;36(3):1-48. doi:10.18637/jss.v036.i03

[13] Cheung MW. metaSEM: an R package for meta-analysis using structural equation modeling. Front Psychol. 2015;5(1521):1521. doi:10.3389/fpsyg.2014.01521 25601849 PMC4283449

[14] Higgins JPT, Thomas J, Chandler J, . Cochrane Handbook for Systematic Reviews of Interventions. 2nd ed. John Wiley & Sons; 2019. doi:10.1002/9781119536604

[15] Saudino KJ, Ronald A, Plomin R. The etiology of behavior problems in 7-year-old twins: substantial genetic influence and negligible shared environmental influence for parent ratings and ratings by same and different teachers. J Abnorm Child Psychol. 2005;33(1):113-130. doi:10.1007/s10802-005-0939-7 15759595

[16] Ronald A, Happé F, Plomin R. A twin study investigating the genetic and environmental aetiologies of parent, teacher and child ratings of autistic-like traits and their overlap. Eur Child Adolesc Psychiatry. 2008;17(8):473-483. doi:10.1007/s00787-008-0689-5 18427861

[17] Egger M, Davey Smith G, Schneider M, Minder C. Bias in meta-analysis detected by a simple, graphical test. BMJ. 1997;315(7109):629-634. doi:10.1136/bmj.315.7109.629 9310563 PMC2127453

[18] Akerman BA, Fischbein S. Within-pair similarity in MZ and DZ twins from birth to eighteen years of age. Acta Genet Med Gemellol (Roma). 1992;41(2-3):155-164. doi:10.1017/S0001566000002361 1302426

[19] Ando J, Nonaka K, Ozaki K, . The Tokyo Twin Cohort Project: overview and initial findings. Twin Res Hum Genet. 2006;9(6):817-826. doi:10.1375/twin.9.6.817 17254415

[20] Bakermans-Kranenburg MJ, van Uzendoorn MH, Bokhorst CL, Schuengel C. The importance of shared environment in infant-father attachment: a behavioral genetic study of the attachment q-sort. J Fam Psychol. 2004;18(3):545-549. doi:10.1037/0893-3200.18.3.545 15382980

[21] Beaver KM, Boutwell BB, Barnes J, Schwartz JA, Connolly EJ. A quantitative genetic analysis of the associations among language skills, peer interactions, and behavioral problems in childhood: results from a sample of twins. Merrill-Palmer Q. 2014;60(2):142-167. doi:10.13110/merrpalmquar1982.60.2.0142

[22] Bishop E, Cherny SS, Corley R, Plomin R, DeFries JC, Hewitt JK. Development genetic analysis of general cognitive ability from 1 to 12 years in a sample of adoptees, biological siblings, and twins. Intelligence. 2003;31(1):31-49. doi:10.1016/S0160-2896(02)00112-5

[23] Bokhorst CL, Bakermans-Kranenburg MJ, Fearon RM, van IJzendoorn MH, Fonagy P, Schuengel C. The importance of shared environment in mother-infant attachment security: a behavioral genetic study. Child Dev. 2003;74(6):1769-1782. doi:10.1046/j.1467-8624.2003.00637.x 14669895

[24] Boomsma DI, Orlebeke JF, van Baal GC. The Dutch Twin Register: growth data on weight and height. Behav Genet. 1992;22(2):247-251. doi:10.1007/BF01067004 1596264

[25] Brant AM, Haberstick BC, Corley RP, Wadsworth SJ, DeFries JC, Hewitt JK. The developmental etiology of high IQ. Behav Genet. 2009;39(4):393-405. doi:10.1007/s10519-009-9268-x 19377873 PMC3086674

[26] Brescianini S, Volzone A, Fagnani C, . Genetic and environmental factors shape infant sleep patterns: a study of 18-month-old twins. Pediatrics. 2011;127(5):e1296-e1302. doi:10.1542/peds.2010-0858 21482604

[27] Brescianini S, Giampietro S, Cotichini R, Lucchini R, De Curtis M. Genetic and environmental components of neonatal weight gain in preterm infants. Pediatrics. 2012;129(2):e455-e459. doi:10.1542/peds.2010-0510 22218835

[28] Caramaschi D, Booij L, Petitclerc A, Boivin M, Tremblay RE. Genetic and environmental contributions to saliva testosterone levels in male and female infant twins. Psychoneuroendocrinology. 2012;37(12):1954-1959. doi:10.1016/j.psyneuen.2012.04.008 22571885

[29] Chen CJ, Yu MW, Wang CJ, . Genetic variance and heritability of temperament among Chinese twin infants. Acta Genet Med Gemellol (Roma). 1990;39(4):485-490. doi:10.1017/S0001566000003718 2102592

[30] Chen CJ, Yu MW, Wang CJ, . Chronological changes in genetic variance and heritability of anthropometric characteristics among Chinese twin infants. Acta Genet Med Gemellol (Roma). 1990;39(4):479-484. doi:10.1017/S0001566000003706 2102591

[31] Cherny SS, Cardon LR, Fulker DW, DeFries JC. Differential heritability across levels of cognitive ability. Behav Genet. 1992;22(2):153-162. doi:10.1007/BF01066994 1596255

[32] Cherny SS, Fulker DW, Corley RP, Plomin R, DeFries JC. Continuity and change in infant shyness from 14 to 20 months. Behav Genet. 1994;24(4):365-379. doi:10.1007/BF01067538 7993315

[33] Cherny SS, Fulker D, Emde R, . A developmental-genetic analysis of continuity and change in the Bayley Mental Development Index from 14 to 24 months: the MacArthur Longitudinal Twin Study. Psychol Sci. 1994;5(6):354-360. doi:10.1111/j.1467-9280.1994.tb00285.x

[34] Custodio RJ, Junior CE, Milani SL, Simões AL, de Castro M, Moreira AC. The emergence of the cortisol circadian rhythm in monozygotic and dizygotic twin infants: the twin-pair synchrony. Clin Endocrinol (Oxf). 2007;66(2):192-197. doi:10.1111/j.1365-2265.2006.02706.x 17223987 PMC1859886

[35] Dale PS, Dionne G, Eley TC, Plomin R. Lexical and grammatical development: a behavioural genetic perspective. J Child Lang. 2000;27(3):619-642. doi:10.1017/S0305000900004281 11089341

[36] Davis DW, Finkel D, Turkheimer E, Dickens W. Genetic and environmental contributions to behavioral stability and change in children 6-36 months of age using Louisville Twin Study data. Behav Genet. 2015;45(6):610-621. doi:10.1007/s10519-015-9759-x 26477572 PMC4749475

[37] DiLalla LF, Bishop EG. Differential maternal treatment of infant twins: effects of infant behaviors. Behav Genet. 1996;26(6):535-542. doi:10.1007/BF02361226 8990532

[38] DiLalla LF, Kagan J, Reznick JS. Genetic etiology of behavioral inhibition among 2-year-old children. Infant Behav Dev. 1994;17:405-412. doi:10.1016/0163-6383(94)90032-9

[39] Dionne G, Dale PS, Boivin M, Plomin R. Genetic evidence for bidirectional effects of early lexical and grammatical development. Child Dev. 2003;74(2):394-412. doi:10.1111/1467-8624.7402005 12705562

[40] Dionne G, Tremblay R, Boivin M, Laplante D, Pérusse D. Physical aggression and expressive vocabulary in 19-month-old twins. Dev Psychol. 2003;39(2):261-273. doi:10.1037/0012-1649.39.2.261 12661885

[41] Dionne G, Touchette E, Forget-Dubois N, . Associations between sleep-wake consolidation and language development in early childhood: a longitudinal twin study. Sleep. 2011;34(8):987-995. doi:10.5665/SLEEP.1148 21804661 PMC3138173

[42] Dubois L, Girard M, Girard A, Tremblay R, Boivin M, Pérusse D. Genetic and environmental influences on body size in early childhood: a twin birth-cohort study. Twin Res Hum Genet. 2007;10(3):479-485. doi:10.1375/twin.10.3.479 17564506

[43] Dubois L, Ohm Kyvik K, Girard M, . Genetic and environmental contributions to weight, height, and BMI from birth to 19 years of age: an international study of over 12,000 twin pairs. PLoS One. 2012;7(2):e30153. doi:10.1371/journal.pone.0030153 22347368 PMC3275599

[44] Emde RN, Plomin R, Robinson JA, . Temperament, emotion, and cognition at fourteen months: the MacArthur Longitudinal Twin Study. Child Dev. 1992;63(6):1437-1455. doi:10.2307/1131567 1446561

[45] Finkel D, Matheny AP Jr. Genetic and environmental influences on a measure of infant attachment security. Twin Res. 2000;3(4):242-250. doi:10.1375/136905200320565210 11463145

[46] Fisher A, van Jaarsveld CH, Llewellyn CH, Wardle J. Genetic and environmental influences on infant sleep. Pediatrics. 2012;129(6):1091-1096. doi:10.1542/peds.2011-1571 22585775

[47] Flom M, Saudino KJ. Callous-unemotional behaviors in early childhood: genetic and environmental contributions to stability and change. Dev Psychopathol. 2017;29(4):1227-1234. doi:10.1017/S0954579416001267 27976598 PMC5472508

[48] Flom M, Saudino KJ. Do genetic factors explain the links between callous-unemotional, attention hyperactivity and oppositional defiant problems in toddlers? J Abnorm Child Psychol. 2018;46(6):1217-1228. doi:10.1007/s10802-017-0361-y 29110116 PMC5936685

[49] Flom M, White D, Ganiban J, Saudino KJ. Longitudinal links between callous-unemotional behaviors and parenting in early childhood: a genetically informed design. J Am Acad Child Adolesc Psychiatry. 2020;59(3):401-409.e2. doi:10.1016/j.jaac.2019.03.013 30877055 PMC6744356

[50] Forget-Dubois N, Boivin M, Dionne G, Pierce T, Tremblay RE, Pérusse D. A longitudinal twin study of the genetic and environmental etiology of maternal hostile-reactive behavior during infancy and toddlerhood. Infant Behav Dev. 2007;30(3):453-465. doi:10.1016/j.infbeh.2006.12.005 17683754

[51] Friedman NP, Miyake A, Robinson JL, Hewitt JK. Developmental trajectories in toddlers’ self-restraint predict individual differences in executive functions 14 years later: a behavioral genetic analysis. Dev Psychol. 2011;47(5):1410-1430. doi:10.1037/a0023750 21668099 PMC3168720

[52] Fujisawa KK, Ozaki K, Suzuki K, Yamagata S, Kawahashi I, Ando J. Genetic and environmental relationships between head circumference growth in the first year of life and sociocognitive development in the second year: a longitudinal twin study. Dev Sci. 2012;15(1):99-112. doi:10.1111/j.1467-7687.2011.01097.x 22251296

[53] Gagne JR, Goldsmith HH. A longitudinal analysis of anger and inhibitory control in twins from 12 to 36 months of age. Dev Sci. 2011;14(1):112-124. doi:10.1111/j.1467-7687.2010.00969.x 21159093 PMC3049157

[54] Gagne JR, Saudino KJ. Wait for it! a twin study of inhibitory control in early childhood. Behav Genet. 2010;40(3):327-337. doi:10.1007/s10519-009-9316-6 19936910 PMC2854273

[55] Gagne JR, Saudino KJ, Asherson P. The genetic etiology of inhibitory control and behavior problems at 24 months of age. J Child Psychol Psychiatry. 2011;52(11):1155-1163. doi:10.1111/j.1469-7610.2011.02420.x 21627653 PMC3184216

[56] Gagne JR, Saudino KJ. The development of inhibitory control in early childhood: a twin study from 2-3 years. Dev Psychol. 2016;52(3):391-399. doi:10.1037/dev0000090 26784384 PMC4839189

[57] Gagne JR, Asherson P, Saudino KJ. A twin study of inhibitory control at age two and ADHD behavior problems at age three. Behav Genet. 2020;50(4):289-300. doi:10.1007/s10519-020-09997-5 32162153

[58] Galsworthy MJ, Dionne G, Dale PS, Plomin R. Sex differences in early verbal and non-verbal cognitive development. Dev Sci. 2000;3(2):206-215. doi:10.1111/1467-7687.00114

[59] German A, Livshits G, Peter I, . Environmental rather than genetic factors determine the variation in the age of the infancy to childhood transition: a twins study. J Pediatr. 2015;166(3):731-735. doi:10.1016/j.jpeds.2014.11.047 25578994

[60] Gilmore JH, Schmitt JE, Knickmeyer RC, . Genetic and environmental contributions to neonatal brain structure: a twin study. Hum Brain Mapp. 2010;31(8):1174-1182. doi:10.1002/hbm.20926 20063301 PMC3109622

[61] Goetghebuer T, Ota MO, Kebbeh B, . Delay in motor development of twins in Africa: a prospective cohort study. Twin Res. 2003;6(4):279-284. doi:10.1375/136905203322296629 14511433

[62] Goldsmith HH, Gottesman II. Origins of variation in behavioral style: a longitudinal study of temperament in young twins. Child Dev. 1981;52(1):91-103. doi:10.2307/1129218 7195330

[63] Goldsmith HH, Lemery KS, Buss KA, Campos JJ. Genetic analyses of focal aspects of infant temperament. Dev Psychol. 1999;35(4):972-985. doi:10.1037/0012-1649.35.4.972 10442866

[64] Hawks ZW, Marrus N, Glowinski AL, Constantino JN. Early origins of autism comorbidity: neuropsychiatric traits correlated in childhood are independent in infancy. J Abnorm Child Psychol. 2019;47(2):369-379. doi:10.1007/s10802-018-0410-1 29546561 PMC6139282

[65] Herle M, Fildes A, Llewellyn CH. Emotional eating is learned not inherited in children, regardless of obesity risk. Pediatr Obes. 2018;13(10):628-631. doi:10.1111/ijpo.12428 29931803 PMC6220812

[66] Hur YM. Genetic and environmental influences on birthweight in a sample of Korean twins. J Korean Med Sci. 2005;20(3):355-360. doi:10.3346/jkms.2005.20.3.355 15953852 PMC2782186

[67] Hur YM, Luciano M, Martin NG, . A comparison of twin birthweight data from Australia, the Netherlands, the United States, Japan, and South Korea: are genetic and environmental variations in birthweight similar in Caucasians and East Asians? Twin Res Hum Genet. 2005;8(6):638-648. doi:10.1375/twin.8.6.638 16354505

[68] Ilott N, Saudino KJ, Wood A, Asherson P. A genetic study of ADHD and activity level in infancy. Genes Brain Behav. 2010;9(3):296-304. doi:10.1111/j.1601-183X.2009.00560.x 20039948 PMC4108194

[69] Ilott NE, Saudino KJ, Asherson P. Genetic influences on attention deficit hyperactivity disorder symptoms from age 2 to 3: a quantitative and molecular genetic investigation. BMC Psychiatry. 2010;10:102. doi:10.1186/1471-244X-10-102 21122117 PMC3014905

[70] Jackson DB. The association between breastfeeding duration and attachment: a genetically informed analysis. Breastfeed Med. 2016;11(6):297-304. doi:10.1089/bfm.2016.0036 27148915

[71] Jha SC, Xia K, Schmitt JE, . Genetic influences on neonatal cortical thickness and surface area. Hum Brain Mapp. 2018;39(12):4998-5013. doi:10.1002/hbm.24340 30144223 PMC6218288

[72] Johnson L, Llewellyn CH, van Jaarsveld CH, Cole TJ, Wardle J. Genetic and environmental influences on infant growth: prospective analysis of the Gemini twin birth cohort. PLoS One. 2011;6(5):e19918. doi:10.1371/journal.pone.0019918 21637764 PMC3103521

[73] Knafo A, Plomin R. Prosocial behavior from early to middle childhood: genetic and environmental influences on stability and change. Dev Psychol. 2006;42(5):771-786. doi:10.1037/0012-1649.42.5.771 16953685

[74] Koeppen-Schomerus G, Spinath FM, Plomin R. Twins and non-twin siblings: different estimates of shared environmental influence in early childhood. Twin Res. 2003;6(2):97-105. doi:10.1375/136905203321536227 12723996

[75] Kuntsi J, Rijsdijk F, Ronald A, Asherson P, Plomin R. Genetic influences on the stability of attention-deficit/hyperactivity disorder symptoms from early to middle childhood. Biol Psychiatry. 2005;57(6):647-654. doi:10.1016/j.biopsych.2004.12.032 15780852

[76] Lacourse E, Boivin M, Brendgen M, . A longitudinal twin study of physical aggression during early childhood: evidence for a developmentally dynamic genome. Psychol Med. 2014;44(12):2617-2627. doi:10.1017/S0033291713003218 24443874

[77] Levine RS, Hennekens CH, Jesse MJ. Genetic variance of weight and length in infant twins. Am J Epidemiol. 1987;126(5):929-935. doi:10.1093/oxfordjournals.aje.a114730 3661540

[78] Liu Q, Yu C, Gao W, . Genetic and environmental effects on weight, height, and BMI Under 18 years in a Chinese population-based twin sample. Twin Res Hum Genet. 2015;18(5):571-580. doi:10.1017/thg.2015.63 26379063

[79] Livshits G, Peter I, Vainder M, Hauspie R. Genetic analysis of growth curve parameters of body weight, height and head circumference. Ann Hum Biol. 2000;27(3):299-312. doi:10.1080/030144600282181 10834294

[80] Llewellyn CH, van Jaarsveld CH, Johnson L, Carnell S, Wardle J. Nature and nurture in infant appetite: analysis of the Gemini twin birth cohort. Am J Clin Nutr. 2010;91(5):1172-1179. doi:10.3945/ajcn.2009.28868 20335548

[81] Marrus N, Glowinski AL, Jacob T, . Rapid video-referenced ratings of reciprocal social behavior in toddlers: a twin study. J Child Psychol Psychiatry. 2015;56(12):1338-1346. doi:10.1111/jcpp.12391 25677414 PMC4775094

[82] Marrus N, Kennon-McGill S, Harris B, Zhang Y, Glowinski AL, Constantino JN. Use of a video scoring anchor for rapid serial assessment of social communication in toddlers. J Vis Exp. 2018;(133):57041. doi:10.3791/57041 29608153 PMC5931764

[83] Marrus N, Grant JD, Harris-Olenak B, . Genetic architecture of reciprocal social behavior in toddlers: implications for heterogeneity in the early origins of autism spectrum disorder. Dev Psychopathol. 2020;32(4):1190-1205. doi:10.1017/S0954579420000723 33161906 PMC8086896

[84] Matheny AP Jr. Bayley’s Infant Behavior Record: behavioral components and twin analyses. Child Dev. 1980;51(4):1157-1167. doi:10.2307/1129557 7193557

[85] Matheny AP Jr. A longitudinal twin study of stability of components from Bayley’s Infant Behavior Record. Child Dev. 1983;54(2):356-360. doi:10.2307/1129696 6683619

[86] Matheny AP Jr. Twin similarity in the developmental transformations of infant temperament as measured in a multi-method, longitudinal study. Acta Genet Med Gemellol (Roma). 1984;33(2):181-189. doi:10.1017/S0001566000007212 6540950

[87] Matheny AP Jr. Children’s behavioral inhibition over age and across situations: genetic similarity for a trait during change. J Pers. 1989;57(2):215-235. doi:10.1111/j.1467-6494.1989.tb00481.x 2769555

[88] Matheny AP Jr, Dolan AB, Wilson RS. Twins: within-pair similarity on Bayley's Infant Behavior Record. J Genet Psychol. 1976;128(2d Half):263-270. doi:10.1080/00221325.1976.10533996 945328

[89] Micalizzi L, Ronald A, Saudino KJ. A genetically informed cross-lagged analysis of autistic-like traits and affective problems in early childhood. J Abnorm Child Psychol. 2016;44(5):937-947. doi:10.1007/s10802-015-0088-6 26456961 PMC4829486

[90] Micalizzi L, Wang M, Saudino KJ. Difficult temperament and negative parenting in early childhood: a genetically informed cross-lagged analysis. Dev Sci. 2017;20(2). doi:10.1111/desc.12355 26490166 PMC4840089

[91] Mook-Kanamori DO, van Beijsterveldt CE, Steegers EA, . Heritability estimates of body size in fetal life and early childhood. PLoS One. 2012;7(7):e39901. doi:10.1371/journal.pone.0039901 22848364 PMC3405108

[92] Nguyen BH, Pérusse D, Paquet J, . Sleep terrors in children: a prospective study of twins. Pediatrics. 2008;122(6):e1164-e1167. doi:10.1542/peds.2008-1303 19047218

[93] Nichols PL, Broman SH. Familial resemblance in infant mental development. Dev Psychol. 1974;10(3):442-446. doi:10.1037/h0036430

[94] Orekhova EV, Stroganova TA, Posikera IN, Malykh SB. Heritability and “environmentability” of electroencephalogram in infants: the twin study. Psychophysiology. 2003;40(5):727-741. doi:10.1111/1469-8986.00073 14696726

[95] Ouellet-Morin I, Boivin M, Dionne G, . Variations in heritability of cortisol reactivity to stress as a function of early familial adversity among 19-month-old twins. Arch Gen Psychiatry. 2008;65(2):211-218. doi:10.1001/archgenpsychiatry.2007.27 18250259

[96] Ouellet-Morin I, Dionne G, Pérusse D, . Daytime cortisol secretion in 6-month-old twins: genetic and environmental contributions as a function of early familial adversity. Biol Psychiatry. 2009;65(5):409-416. doi:10.1016/j.biopsych.2008.10.003 19013558

[97] Peter I, Vainder M, Livshits G. Genetic analysis of motor milestones attainment in early childhood. Twin Res. 1999;2(1):1-9. doi:10.1375/twin.2.1.1 10392796

[98] Petitclerc A, Boivin M, Dionne G, Pérusse D, Tremblay RE. Genetic and environmental etiology of disregard for rules. Behav Genet. 2011;41(2):192-200. doi:10.1007/s10519-010-9393-6 20872238

[99] Pimpin L, Ambrosini GL, Llewellyn CH, . Dietary intake of young twins: nature or nurture? Am J Clin Nutr. 2013;98(5):1326-1334. doi:10.3945/ajcn.113.065250 24047917 PMC3798084

[100] Planalp EM, Van Hulle C, Lemery-Chalfant K, Goldsmith HH. Genetic and environmental contributions to the development of positive affect in infancy. Emotion. 2017;17(3):412-420. doi:10.1037/emo0000238 27797564 PMC5367954

[101] Plomin R, Rowe DC. Genetic and environmental etiology of social behavior in infancy. Dev Psychol. 1979;15(1):62-72. doi:10.1037/h0078078

[102] Plomin R, Emde RN, Braungart JM, . Genetic change and continuity from fourteen to twenty months: the MacArthur Longitudinal Twin Study. Child Dev. 1993;64(5):1354-1376. doi:10.2307/1131539 8222877

[103] Price TS, Eley TC, Dale PS, Stevenson J, Saudino K, Plomin R. Genetic and environmental covariation between verbal and nonverbal cognitive development in infancy. Child Dev. 2000;71(4):948-959. doi:10.1111/1467-8624.00201 11016558

[104] Price TS, Simonoff E, Asherson P, . Continuity and change in preschool ADHD symptoms: longitudinal genetic analysis with contrast effects. Behav Genet. 2005;35(2):121-132. doi:10.1007/s10519-004-1013-x 15685426

[105] Pushina NN, Orekhova EV, Stroganova TA. Age-related and individual differences in the performance of a delayed response task (the A-not-B task) in infant twins aged 7-12 months. Neurosci Behav Physiol. 2005;35(5):481-490. doi:10.1007/s11055-005-0083-4 16033196

[106] Reznick JS, Corley R, Robinson J. A longitudinal twin study of intelligence in the second year. Monogr Soc Res Child Dev. 1997;62(1):i-160. doi:10.2307/1166193 9185344

[107] Rhee SH, Cosgrove VE, Schmitz S, Haberstick BC, Corley RC, Hewitt JK. Early childhood temperament and the covariation between internalizing and externalizing behavior in school-aged children. Twin Res Hum Genet. 2007;10(1):33-44. doi:10.1375/twin.10.1.33 17539363

[108] Rhee SH, Corley RP, Friedman NP, . The etiology of observed negative emotionality from 14 to 24 months. Front Genet. 2012;3:9. doi:10.3389/fgene.2012.00009 22303413 PMC3270250

[109] Rhee SH, Friedman NP, Boeldt DL, . Early concern and disregard for others as predictors of antisocial behavior. J Child Psychol Psychiatry. 2013;54(2):157-166. doi:10.1111/j.1469-7610.2012.02574.x 23320806 PMC3547395

[110] Rhee SH, Friedman NP, Corley RP, . An examination of the developmental propensity model of conduct problems. J Abnorm Psychol. 2016;125(4):550-564. doi:10.1037/abn0000128 26653135 PMC4850109

[111] Rice ML, Zubrick SR, Taylor CL, Gayán J, Bontempo DE. Late language emergence in 24-month-old twins: heritable and increased risk for late language emergence in twins. J Speech Lang Hear Res. 2014;57(3):917-928. doi:10.1044/1092-4388(2013/12-0350) 24167238 PMC3975649

[112] Riese ML. Genetic influences on neonatal temperament. Acta Genet Med Gemellol (Roma). 1990;39(2):207-213. doi:10.1017/S0001566000005432 2239106

[113] Riese ML. Neonatal temperament in monozygotic and dizygotic twin pairs. Child Dev. 1990;61(4):1230-1237. doi:10.2307/1130890 2209192

[114] Robinson JL, Kagan J, Reznick JS, Corley R. The heritability of inhibited and uninhibited behavior: a twin study. Dev Psychol. 1992;28(6):1030-1037. doi:10.1037/0012-1649.28.6.1030

[115] Roisman GI, Fraley RC. The limits of genetic influence: a behavior-genetic analysis of infant-caregiver relationship quality and temperament. Child Dev. 2006;77(6):1656-1667. doi:10.1111/j.1467-8624.2006.00965.x 17107452

[116] Roisman GI, Fraley RC. A behavior-genetic study of parenting quality, infant attachment security, and their covariation in a nationally representative sample. Dev Psychol. 2008;44(3):831-839. doi:10.1037/0012-1649.44.3.831 18473647

[117] Ronald A, Edelson LR, Asherson P, Saudino KJ. Exploring the relationship between autistic-like traits and ADHD behaviors in early childhood: findings from a community twin study of 2-year-olds. J Abnorm Child Psychol. 2010;38(2):185-196. doi:10.1007/s10802-009-9366-5 19908138 PMC4096900

[118] Saudino KJ. Sources of continuity and change in activity level in early childhood. Child Dev. 2012;83(1):266-281. doi:10.1111/j.1467-8624.2011.01680.x 22103336 PMC3266954

[119] Saudino KJ, Zapfe JA. Genetic influences on activity level in early childhood: do situations matter? Child Dev. 2008;79(4):930-943. doi:10.1111/j.1467-8624.2008.01168.x 18717899 PMC4113970

[120] Saudino KJ, Eaton WO. Infant temperament and genetics: an objective twin study of motor activity level. Child Dev. 1991;62(5):1167-1174. doi:10.2307/1131160 1756660

[121] Saudino KJ, Plomin R, DeFries JC. Tester-rated temperament at 14, 20 and 24 months: environmental change and genetic continuity. Br J Dev Psychol. 1996;14(2):129-144. doi:10.1111/j.2044-835X.1996.tb00697.x

[122] Saudino KJ, Carter AS, Purper-Ouakil D, Gorwood P. The etiology of behavioral problems and competencies in very young twins. J Abnorm Psychol. 2008;117(1):48-62. doi:10.1037/0021-843X.117.1.48 18266485 PMC4103163

[123] Saudino KJ, Wang M, Flom M, Asherson P. Genetic and environmental links between motor activity level and attention problems in early childhood. Dev Sci. 2018;21(5):e12630. doi:10.1111/desc.12630 29119648 PMC6693496

[124] Schmitz S, Fulker DW, Plomin R, Zahn-Waxler C, Emde RN, DeFries JC. Temperament and problem behavior during early childhood. Int J Behav Dev. 1999;23(2):333-355. doi:10.1080/016502599383856

[125] Schumann L, Boivin M, Paquin S, . Persistence and innovation effects in genetic and environmental factors in negative emotionality during infancy: a twin study. PLoS One. 2017;12(4):e0176601. doi:10.1371/journal.pone.0176601 28448561 PMC5407782

[126] Silberg JL, Miguel VF, Murrelle EL, . Genetic and environmental influences on temperament in the first year of life: the Puerto Rico Infant Twin Study (PRINTS). Twin Res Hum Genet. 2005;8(4):328-336. doi:10.1375/twin.8.4.328 16176717

[127] Silberg JL, Gillespie N, Moore AA, . Shared genetic and environmental influences on early temperament and preschool psychiatric disorders in Hispanic twins. Twin Res Hum Genet. 2015;18(2):171-178. doi:10.1017/thg.2014.88 25728588 PMC4402136

[128] Silventoinen K, Pietiläinen KH, Tynelius P, Sørensen TI, Kaprio J, Rasmussen F. Genetic and environmental factors in relative weight from birth to age 18: the Swedish young male twins study. Int J Obes (Lond). 2007;31(4):615-621. doi:10.1038/sj.ijo.0803577 17384662

[129] Silventoinen K, Pietiläinen KH, Tynelius P, Sørensen TI, Kaprio J, Rasmussen F. Genetic regulation of growth from birth to 18 years of age: the Swedish young male twins study. Am J Hum Biol. 2008;20(3):292-298. doi:10.1002/ajhb.20717 18203123

[130] Silventoinen K, Karvonen M, Sugimoto M, Kaprio J, Dunkel L, Yokoyama Y. Genetics of head circumference in infancy: a longitudinal study of Japanese twins. Am J Hum Biol. 2011;23(5):630-634. doi:10.1002/ajhb.21190 21630369

[131] Silventoinen K, Kaprio J, Yokoyama Y. Genetics of pre-pubertal growth: a longitudinal study of Japanese twins. Ann Hum Biol. 2011;38(5):608-614. doi:10.3109/03014460.2011.587453 21657978

[132] Silventoinen K, Kaprio J, Dunkel L, Yokoyama Y. Genetic and environmental influences on chest circumference during infancy: a longitudinal study of Japanese twins. Paediatr Perinat Epidemiol. 2012;26(6):553-560. doi:10.1111/ppe.12003 23061691

[133] Silventoinen K, Jelenkovic A, Sund R, . Genetic and environmental effects on body mass index from infancy to the onset of adulthood: an individual-based pooled analysis of 45 twin cohorts participating in the COllaborative project of Development of Anthropometrical measures in Twins (CODATwins) study. Am J Clin Nutr. 2016;104(2):371-379. doi:10.3945/ajcn.116.130252 27413137 PMC4962159

[134] Smit DJ, Luciano M, Bartels M, . Heritability of head size in Dutch and Australian twin families at ages 0-50 years. Twin Res Hum Genet. 2010;13(4):370-380. doi:10.1375/twin.13.4.370 20707707

[135] Smith AK, Rhee SH, Corley RP, Friedman NP, Hewitt JK, Robinson JL. The magnitude of genetic and environmental influences on parental and observational measures of behavioral inhibition and shyness in toddlerhood. Behav Genet. 2012;42(5):764-777. doi:10.1007/s10519-012-9551-0 22806186 PMC3443291

[136] Smith AD, Herle M, Fildes A, Cooke L, Steinsbekk S, Llewellyn CH. Food fussiness and food neophobia share a common etiology in early childhood. J Child Psychol Psychiatry. 2017;58(2):189-196. doi:10.1111/jcpp.12647 27739065 PMC5298015

[137] Smith L, van Jaarsveld CHM, Llewellyn CH, . Genetic and environmental influences on developmental milestones and movement: results from the Gemini Cohort Study. Res Q Exerc Sport. 2017;88(4):401-407. doi:10.1080/02701367.2017.1373268 29048262

[138] Soussignan R, Boivin M, Girard A, Pérusse D, Liu X, Tremblay RE. Genetic and environmental etiology of emotional and social behaviors in 5-month-old infant twins: influence of the social context. Infant Behav Dev. 2009;32(1):1-9. doi:10.1016/j.infbeh.2008.09.002 18995913

[139] Spinath FM, Ronald A, Harlaar N, Price TS, Plomin R. Phenotypic *g* early in life: on the etiology of general cognitive ability in a large population sample of twin children aged 2-4 years. Intelligence. 2003;31(2):195-210. doi:10.1016/S0160-2896(02)00110-1

[140] Stevenson J, Fielding J. Ratings of temperament in families of young twins. Br J Dev Psychol. 1985;3(2):143-152. doi:10.1111/j.2044-835X.1985.tb00966.x

[141] Stroganova T, Tsetlin MM, Malykh SB, Malakhovskaya EV. Biological principles of individual differences of children of the second half-year of life: communication II: the nature of individual differences in temperamental features. Hum Physiol. 2000;26:281-289. doi:10.1007/BF02760188

[142] Touchette E, Dionne G, Forget-Dubois N, . Genetic and environmental influences on daytime and nighttime sleep duration in early childhood. Pediatrics. 2013;131(6):e1874-e1880. doi:10.1542/peds.2012-2284 23713101

[143] Touwslager RN, Gielen M, Derom C, . Determinants of infant growth in four age windows: a twin study. J Pediatr. 2011;158(4):566-572.e2. doi:10.1016/j.jpeds.2010.10.005 21147487

[144] Touwslager RN, Gielen M, Mulder AL, . Changes in genetic and environmental effects on growth during infancy. Am J Clin Nutr. 2011;94(6):1568-1574. doi:10.3945/ajcn.111.012757 22071713

[145] Tucker-Drob EM, Rhemtulla M, Harden KP, Turkheimer E, Fask D. Emergence of a gene x socioeconomic status interaction on infant mental ability between 10 months and 2 years. Psychol Sci. 2011;22(1):125-133. doi:10.1177/0956797610392926 21169524 PMC3532898

[146] van Dommelen P, de Gunst MC, van der Vaart AW, Boomsma DI. Genetic study of the height and weight process during infancy. Twin Res. 2004;7(6):607-616. doi:10.1375/1369052042663805 15607012

[147] Wang M, Saudino KJ. Genetic and environmental contributions to stability and change of sleep problems in toddlerhood. J Pediatr Psychol. 2012;37(6):697-706. doi:10.1093/jpepsy/jss048 22438469 PMC3381715

[148] Whitfield JB, Treloar SA, Zhu G, Martin NG. Genetic and non-genetic factors affecting birth-weight and adult body mass index. Twin Res. 2001;4(5):365-370. doi:10.1375/twin.4.5.365 11869490

[149] Wilson RS. Twins: early mental development. Science. 1972;175(4024):914-917. doi:10.1126/science.175.4024.914 5061798

[150] Wilson RS. Twins: mental development in the preschool years. Dev Psychol. 1974;10(4):580-588. doi:10.1037/h0036596

[151] Wilson RS. Synchronies in mental development: an epigenetic perspective. Science. 1978;202(4371):939-948. doi:10.1126/science.568822 568822

[152] Wilson RS. The Louisville Twin Study: developmental synchronies in behavior. Child Dev. 1983;54(2):298-316. doi:10.2307/1129693 6683617

[153] Wilson RS. Twins and chronogenetics: correlated pathways of development. Acta Genet Med Gemellol (Roma). 1984;33(2):149-157. doi:10.1017/S0001566000007170 6540946

[154] Wilson RS, Harpring EB. Mental and motor development in infant twins. Dev Psychol. 1972;7(3):277-287. doi:10.1037/h0033357

[155] Wilson RS, Matheny AP Jr. Retardation and twin concordance in infant mental development: a reassessment. Behav Genet. 1976;6(3):353-358. doi:10.1007/BF01065730 987783

[156] Woodward KE, Boeldt DL, Corley RP, . Correlates of positive parenting behaviors. Behav Genet. 2018;48(4):283-297. doi:10.1007/s10519-018-9906-2 29876694 PMC6281807

[157] Faraone SV, Larsson H. Genetics of attention deficit hyperactivity disorder. Mol Psychiatry. 2019;24(4):562-575. doi:10.1038/s41380-018-0070-0 29892054 PMC6477889

[158] Tick B, Bolton P, Happé F, Rutter M, Rijsdijk F. Heritability of autism spectrum disorders: a meta-analysis of twin studies. J Child Psychol Psychiatry. 2016;57(5):585-595. doi:10.1111/jcpp.12499 26709141 PMC4996332

[159] Davis OS, Haworth CM, Plomin R. Dramatic increase in heritability of cognitive development from early to middle childhood: an 8-year longitudinal study of 8,700 pairs of twins. Psychol Sci. 2009;20(10):1301-1308. doi:10.1111/j.1467-9280.2009.02433.x 19732386 PMC4040420

[160] Plomin R, Fulker DW, Corley R, DeFries JC. Nature, nurture, and cognitive development from 1 to 16 years: a parent–offspring adoption study. Psychol Sci. 1997;8(6):442-447. doi:10.1111/j.1467-9280.1997.tb00458.x

[161] Neale MC, Stevenson J. Rater bias in the EASI temperament scales: a twin study. J Pers Soc Psychol. 1989;56(3):446-455. doi:10.1037/0022-3514.56.3.446 2926639

[162] Deary IJ, Weiss A, Batty GD. Intelligence and personality as predictors of illness and death: how researchers in differential psychology and chronic disease epidemiology are collaborating to understand and address health inequalities. Psychol Sci Public Interest. 2010;11(2):53-79. doi:10.1177/1529100610387081 26168413

[163] Plomin R, DeFries JC, Knopik VS, Neiderhiser JM. Top 10 replicated findings from behavioral genetics. Perspect Psychol Sci. 2016;11(1):3-23. doi:10.1177/1745691615617439 26817721 PMC4739500

[164] Plomin R. Commentary: why are children in the same family so different: non-shared environment three decades later. Int J Epidemiol. 2011;40(3):582-592. doi:10.1093/ije/dyq144 21807643 PMC3147062

[165] Flom M, Wang M, Uccello KJ, Saudino KJ. Parent- and observer-rated positive affect in early childhood: genetic overlap and environmental specificity. Behav Genet. 2018;48(6):432-439. doi:10.1007/s10519-018-9924-0 30259223 PMC6186175

[166] MacGillivray I, Campbell DM, Thompson B, eds. Twinning and Twins. Wiley; 1988.

[167] Ronalds GA, De Stavola BL, Leon DA. The cognitive cost of being a twin: evidence from comparisons within families in the Aberdeen children of the 1950s cohort study. BMJ. 2005;331(7528):1306. doi:10.1136/bmj.38633.594387.3A 16299014 PMC1298851

[168] Eaves LJ. The resolution of genotype x environment interaction in segregation analysis of nuclear families. Genet Epidemiol. 1984;1(3):215-228. doi:10.1002/gepi.1370010302 6544238

[169] Verhulst B. A power calculator for the classical twin design. Behav Genet. 2017;47(2):255-261. doi:10.1007/s10519-016-9828-9 27866285 PMC5471839

