# Supplementary material for: Heritability of Psychological Traits and Developmental Milestones in Infancy: A Systematic Review and Meta-analysis
Source: JAMA Netw Open. 2022 Aug 22;5(8):e2227887. doi: 10.1001/jamanetworkopen.2022.27887 (PMC9396365; doi:10.1001/jamanetworkopen.2022.27887)
Supplement: Supplement. — eMethods. eResults. eTable 1. Database and Reference List Searches eTable 2. Inclusion and Exclusion Criteria eTable 3. Information Extracted From Included Publications eTable 4. Publications Identified in Systematic Literature Search, Presented Alphabetically by First Author eTable 5. Twin Studies Identified in Systematic Literature Search, Presented Alphabetically by Study Name eTable 6. Phenotypes Identified in Systematic Literature Search Coded Using Classification System eTable 7. Estimates and Cohorts in Phenotypic Categories by Rater eTable 8. Multilevel Random Effects Models of Phenotypic Subcategories eTable 9. Multilevel Random Effects Models of Parent and Observer Ratings of 3 Phenotypic Categories eTable 10. Tests for Publication Bias on Twin Correlations by Phenotype Category eTable 11. Tests for Publication Bias on Phenotype Categories by Variance Component in Phenotype Categories With ≥10 Estimates eFigure 1. Prisma Flow Diagram eFigure 2. Bar Chart of Number of Twin Pairs by Country eFigure 3. Bar Chart of Number of Twin Pairs by Continent eFigure 4. Sleep Functions Forest Plot eFigure 5. Attention Functions Forest Plot eFigure 6. Psychomotor Functions Forest Plot eFigure 7. Emotional Functions Forest Plot eFigure 8. Basic Cognitive Functions Forest Plot eFigure 9. Mental Functions of Language Forest Plot eFigure 10. Growth Maintenance Functions Forest Plot eFigure 11. Basic Interpersonal Interactions Forest Plot eFigure 12. Complex Interpersonal Interactions Forest Plot eFigure 13. Family Relationships Forest Plot eFigure 14. Funnel Plots of Association Between Monozygotic Twin Correlation and Standard Error in Phenotype Categories With ≥10 Estimates eFigure 15. Funnel Plots of Association Between Dizygotic Twin Correlation and Standard Error in Phenotype Categories With ≥10 Estimates eFigure 16. Funnel Plots of Association Between Heritability and Standard Error in Phenotype Categories With ≥10 Estimates eFigure 17. Funnel Plots of Association Betw [file jamanetwopen-e2227887-s001.pdf]

Supplemental Online Content

Austerberry C, Mateen M, Fearon P, Ronald A. Heritability of psychological traits and developmental milestones in infancy: a systematic review and meta-analysis. *JAMA Netw Open*. 2022;5(8):e2227887. doi:10.1001/jamanetworkopen.2022.27887

eMethods.
eResults.
eTable 1. Database and Reference List Searches
eTable 2. Inclusion and Exclusion Criteria
eTable 3. Information Extracted From Included Publications
eTable 4. Publications Identified in Systematic Literature Search, Presented Alphabetically by First Author
eTable 5. Twin Studies Identified in Systematic Literature Search, Presented Alphabetically by Study Name
eTable 6. Phenotypes Identified in Systematic Literature Search Coded Using Classification System
eTable 7. Estimates and Cohorts in Phenotypic Categories by Rater
eTable 8. Multilevel Random Effects Models of Phenotypic Subcategories
eTable 9. Multilevel Random Effects Models of Parent and Observer Ratings of 3 Phenotypic Categories
eTable 10. Tests for Publication Bias on Twin Correlations by Phenotype Category
eTable 11. Tests for Publication Bias on Phenotype Categories by Variance Component in Phenotype Categories With ≥10 Estimates
eFigure 1. Prisma Flow Diagram
eFigure 2. Bar Chart of Number of Twin Pairs by Country
eFigure 3. Bar Chart of Number of Twin Pairs by Continent
eFigure 4. Sleep Functions Forest Plot
eFigure 5. Attention Functions Forest Plot
eFigure 6. Psychomotor Functions Forest Plot
eFigure 7. Emotional Functions Forest Plot
eFigure 8. Basic Cognitive Functions Forest Plot
eFigure 9. Mental Functions of Language Forest Plot
eFigure 10. Growth Maintenance Functions Forest Plot
eFigure 11. Basic Interpersonal Interactions Forest Plot
eFigure 12. Complex Interpersonal Interactions Forest Plot
eFigure 13. Family Relationships Forest Plot
eFigure 14. Funnel Plots of Association Between Monozygotic Twin Correlation and Standard Error in Phenotype Categories With ≥10 Estimates
eFigure 15. Funnel Plots of Association Between Dizygotic Twin Correlation and Standard Error in Phenotype Categories With ≥10 Estimates
eFigure 16. Funnel Plots of Association Between Heritability and Standard Error in Phenotype Categories With ≥10 Estimates
eFigure 17. Funnel Plots of Association Between Shared Environment and Standard Error in Phenotype Categories With ≥10 Estimates
eFigure 18. Funnel Plots of Association Between Nonshared Environment and Standard Error in Phenotype Categories With ≥10 Estimates
eFigure 19. Bar Plot of Quality Assessment Scores
eFigure 20. Sustaining Attention Forest Plot
eFigure 21. Psychomotor Control Forest Plot

© 2022 Austerberry C et al. *JAMA Network Open.*

eFigure 22. Organization of Psychomotor Functions Forest Plot
eFigure 23. Regulation of Emotion Forest Plot
eFigure 24. Range of Emotion Forest Plot
eFigure 25. Expression of Language Forest Plot
eFigure 26. Respect and Warmth in Relationships Forest Plot
eFigure 27. Social Cues in Relationships Forest Plot
eFigure 28. Regulating Behaviors Within Interactions Forest Plot
eFigure 29. Interacting According to Social Rules Forest Plot
eFigure 30. Psychomotor Functions (Observer Report) Forest Plot
eFigure 31. Psychomotor Functions (Parent Report) Forest Plot
eFigure 32. Emotional Functions (Observer Report) Forest Plot
eFigure 33. Emotional Functions (Parent Report) Forest Plot
eFigure 34. Basic Interpersonal Interactions (Observer Report) Forest Plot
eFigure 35. Basic Interpersonal Interactions (Parent Report) Forest Plot
eReferences.

This supplemental material has been provided by the authors to give readers additional information about their work.

© 2022 Austerberry C et al. *JAMA Network Open.*

eMethods

Classical Twin Design
We searched databases for results from classical twin studies. The classical twin design is based on the comparison of phenotypic similarity within monozygotic (MZ) twins and dizygotic (DZ) twins. As MZ twins share 100% of their segregating genome and DZ twins share on average 50%, a higher degree of phenotypic similarity within MZ than DZ twins indicates genetic influence. When DZ twins show within-pair similarity greater than half of that of MZ twins, this suggests shared environmental influences (defined as environmental influences that make children growing up in the same family similar). Differences between MZ twins are attributed to nonshared environmental influences, which are influences of aspects of the environment that children growing up in the same family do not share.

Quality Assessment
Publications were quality assessed using a modified version of the checklist for assessing the quality of quantitative studies[1], which rates studies on a scale from 0 to 2 (2 = Yes; 1 = Partial; 0 = No; NA = Not Applicable) on the following 14 items:

1. Question/objective sufficiently described?
2. Study design evident and appropriate?
3. Method of subject/comparison group selection or source of information/input variables described and appropriate?
4. Subject (and comparison group, if applicable) characteristics sufficiently described?
5. If interventional and random allocation was possible, was it described?
6. If interventional and blinding of investigators was possible, was it reported?
7. If interventional blinding of subjects was possible, was it reported?
8. Outcome and (if applicable) exposure measure(s) well defined and robust to measurement/misclassification bias? Means of assessment reported?
9. Sample size appropriate?
10. Analytic methods described/justified and appropriate?
11. Some estimate of variance is reported for the main results?
12. Controlled for confounding?
13. Results reported in sufficient detail?
14. Conclusion supported by the results?

The modifications we made to make the tool suitable for assessing the quality of twin studies were: (1) removal of items 5 and 7, because classical twin studies do not include interventional and random allocation or interventional blinding of subjects; (2) removal of item 12 from the assessment, as the confounds that we were most interested in are controlled for by the twin design itself (e.g. age, genetic influences, shared rearing environment); (3) adaption of item 6 (rewording it to: “If possible, were investigators blind to zygosity or task outcome?”); (4) introduction of an additional item (“Sample included only same-sex twins, or same-sex and opposite-sex twins were separated in the analyses?”), because, if opposite-sex twins are treated more differently than same-sex twins, inclusion of data from opposite-sex twins introduces an additional source of difference between MZ and DZ twins and can lead to inflated heritability estimates.

Duplicate Information
It is common for univariate twin results from a single study to be reported in more than one publication because multivariate twin studies often report univariate estimates for all included phenotypes. Consequently, when one phenotype from a twin sample is analyzed in relation to different phenotypes across more than one publication, each publication will typically report univariate estimates for the focal phenotype. As a result, we searched extracted data for overlapping/duplicate information (e.g., publication reporting estimates relating to the same phenotype, measure, age, and cohort) and excluded duplicates from the meta-analysis (prioritizing inclusion of estimates with larger and more recently published estimates).

Three-Level Multilevel Random Effects Meta-Analysis
Before running the meta-analyses, we prepared the extracted data. If studies reported only heritability, shared and nonshared environment (ACE) estimates or both ACE estimates and concordances (not correlations), we used Falconer’s formulas to convert the ACE estimates into correlations.[2] Variances were then calculated using the within-twin correlations (rMZ and rDZ) and sample sizes. The correlations and variances were then used in the three-level multilevel random effects meta-analyses. We conducted three-level multilevel random effects models to facilitate the simultaneous estimation of multiple dependent effect sizes within a single cohort. This

© 2022 Austerberry C et al. *JAMA Network Open.*

allowed us to include all data published in studies that reported estimates from same-sex and opposite-sex twins separately (and sometimes same-sex male and same-sex female, separately) as well as those that reported multiple assessments (e.g., repeated measures) or multiple different measures of the same or similar phenotypes—all of which are common occurrences in large twin studies and registries. Although ideally we would have incorporated the correlations among dependent effect sizes, this was not possible as this information was not usually reported. Evidence suggest that the hierarchical approach estimates effects well even without such information [3]. Combining same-sex and opposite-sex DZ twins in analyses has the potential to inflate heritability estimates if opposite-sex twins DZ twins are systematically less similar (because of sex effects) than same-sex DZ twins, as sex limited genetic effects will end up in a lower DZ correlation and thus an inflated genetic estimate. However, as many of the synthesized studies combined data from same-sex and opposite-sex DZ twins, we were not able to run sufficiently powered analyses using only estimates from same-sex twins or modelling same sex and opposite sex twins separately. We included the following types of estimate in our analyses: estimates from combined samples of same-sex and opposite-sex DZ twins (labelled DZ in eFigures 4–13 and 20–35), DZ estimates from samples that did not specify whether twins were same-sex, opposite-sex or combined (labelled DZ in eFigures 4–13 and 20–35); estimates from same-sex DZ twins (labelled DZSS, DZF [if same-sex and female only], or DZM [if same-sex and male only] in eFigures 4–13 and 20–35) and estimates from opposite-sex DZ twins (DZOS). For example, if a study only reported one estimate per phenotype from DZ twins we included only that one estimate, whereas if a study separately reported twin correlations for a phenotype from DZOS, DZM and DZF, we extracted and separately included all three of the estimates from that study. Dependency between estimates from the same study was taken into account in the multilevel models, which accounted for within- and between-cohort variance. The script for the meta-analyses is available on the Open Science Framework (https://osf.io/4y7z8/?view_only=8d98cb5ce4224e15a401fa5dd658e878).

Meta-Analytic SEM Models

After running the multilevel random effects meta-analyses, we calculated ACE estimates by running meta-analytic SEM models. These models were estimated using the correlations and squared standard errors (variances) from each of the two the multilevel meta-analyses (one to estimate MZ and one to estimate DZ), for each phenotype category, thus allowing parameters and their confidence intervals to be estimated taking account of between-study heterogeneity. Confidence intervals for the ACE parameter estimates were obtained using likelihood-based confidence interval estimation in OpenMx. The script for the meta-analytic twin modelling and confidence interval estimation is available on the Open Science Framework (https://osf.io/4y7z8/?view_only=8d98cb5ce4224e15a401fa5dd658e878).

Forest Plots

The script used to create the Forest plots displayed in (eFigures 4–13, 20–35) is available on the Open Science Framework (https://osf.io/4y7z8/?view_only=8d98cb5ce4224e15a401fa5dd658e878). There are small discrepancies between the main results (presented in Table 1 and eTables 8 and 9) and the pooled estimates in the forest plots because the main results were estimated based on all the pooled data whereas the forest plots were created using MZ and DZ subsets, separately. The main results, based on all the pooled data, provide the most precise estimates. The phenotype category with the greatest discrepancy between the two methods was ‘family relationships’.

© 2022 Austerberry C et al. *JAMA Network Open.*

eResults

Breakdown by Geographical Location

More than half (52.55%) of the twin pairs were from European samples, around a quarter (24.09%) were from North American samples, 15.72% were based in Asia, 7.38% in Oceania and less than 1% were from African or South American samples (0.24% and 0.02%, respectively). For a breakdown of twin pairs by country, see eFigure 2, and by continent, see eFigure 3.

Exclusions From Meta-Analysis

Of the 33 publications excluded from the meta-analysis, 22 were excluded because they contained no novel data—i.e., they only contained relevant findings that were reported in one or more of the other included publications. Eighteen of the 377 phenotypes (4.8%) were excluded from the meta-analysis because they did not fit into a category of the ICF-CY (see final row of eTable 6), resulting in the exclusion of two publications. Fifty-nine phenotypes (15.6%) were excluded from the meta-analysis because they fell into a category of the ICF-CY that contained phenotypes gathered from fewer than five independent twin samples, resulting in the exclusion of nine publications. Extracted data, including the data excluded from the meta-analysis, is available on the Open Science Framework (https://osf.io/4y7z8/?view_only=8d98cb5ce4224e15a401fa5dd658e878).

Sub-category Analysis

The ten sub-categories of the ICF-CY containing data from five or more independent samples were meta-analyzed. Definitions for each sub-category can be found in the ICF-CY[4]. Lists of all phenotypes included in each are displayed in eTable 6. Results from the meta-analyses of sub-categories are reported in eTable 8. Forest plots for these analyses are reported in eFigures 20–29.

Heritability: ‘Psychomotor control’ (h^2_{pooled} = .61; 95% CI [.26–.75] $p < .001$), and ‘regulating behaviors within interactions’ (h^2_{pooled} = .58; 95% CI [.18–.87] $p = .004$) had the highest heritability estimates. ‘Sustaining attention’, ‘organization of psychomotor functions’, ‘range of emotion’, ‘regulation of emotion’, and ‘social cues in relationships’ also had high heritability estimates (h^2_{pooled} range: .41–.50). Heritability estimates for ‘respect and warmth in relationships’, ‘acting in accordance with social rules’ and ‘expression of language’ were small-to-moderate and had confidence intervals that overlapped with zero (h^2_{pooled} range: .20–.31).

Shared Environment: ‘Expression of language’ had the highest shared environmental estimate (c^2_{pooled} = .59; 95% CI [.34–.85] $p < .001$). ‘Respect and warmth in relationships’, ‘acting in accordance with social rules’, and ‘organization of psychomotor functions’ also had moderate-to-high shared environmental estimates (c^2_{pooled} range: .29–.42) with 95% confidence intervals that did not overlap with zero. Shared environmental estimates for ‘psychomotor control’, ‘sustaining attention’, ‘regulation of emotion’, ‘regulating behaviors’, ‘range of emotion’, and ‘social cues in relationships’ were small and had 95% confidence intervals that overlapped with zero (c^2_{pooled} range: .00–.18).

Nonshared Environment: Each of the ten phenotypic sub-categories had nonshared environmental estimates with 95% confidence intervals that did not overlap with zero (e^2_{pooled} range: .14–.47). The category with the highest nonshared environmental estimate was ‘regulation of emotion’ (e^2_{pooled} = .47; 95% CI [.35–.59] $p < .001$).

Heterogeneity: As displayed in eTable 8, sampling variance contributed a proportionally small amount to the total variance of each of the ten phenotypic sub-categories ($I^2_{Level\ 1}$ range: 0.21%–16.31%). Within-cohort heterogeneity contributed a substantial amount to the total variance in ‘respect and warmth in relationships’, ‘range of emotion’ and ‘expression of language’ ($I^2_{Level\ 2}$ range: 58.26%–81.71%) and between-cohort heterogeneity contributed a low or moderate amount to these outcomes ($I^2_{Level\ 3}$ range: 17.78%–41.53%). The remaining seven sub-categories each had substantial between-cohort heterogeneity ($I^2_{Level\ 3}$ = 51.30%–98.07%) and low or moderate within-cohort heterogeneity ($I^2_{Level\ 2}$ range: 0.00%–43.00%).

Analysis of Phenotypes by Category and Rater

Each estimate included in the meta-analysis was coded as: ‘parent-rated’, ‘observer-rated’, or ‘other’. ‘Other’ included estimates where the rater was not specified, as well as objective measurement such as neuroimaging, measurement of birth weight/length, cortisol sampling and actigraphy. Twenty-seven independent cohorts contained estimates coded as ‘other’. However, this was largely driven by the ‘Growth maintenance functions’ category, which contained data on anthropometric characteristics, the measurements for which were all coded as ‘other’. Overall, there were more independent cohorts with parent-reported data (k_{cohort} = 22) than observer-rated data (k_{cohort} = 12). Examining the ICF-CY categories individually, in 7 out of the 10 categories there were more

© 2022 Austerberry C et al. *JAMA Network Open.*

independent cohorts containing parent-report data than observer-rated data. It was only in the 'Basic cognitive functions' category that there were more cohorts reporting observer ratings than parent ratings. In 'Family relationships' there were an equal number of cohorts containing parent and observer reports. For a breakdown of the phenotypic categories by rater subgroup, see eTable 7. There is evidence that heritability estimates can differ by rater, thus we also conducted analyses of ICF-CY categories split by informant (parent or observer). Three of the ICF-CY categories contained data from five or more independent samples for both subgroups: (1) parent-reported and (2) observer-rated phenotypes. Data from each of these six subgroups (three parent-report and three observer-report) were meta-analyzed. Results from these analyses are reported in eTable 9. Forest plots for the analyses are reported in eFigures 30–35.

Heritability. The heritability point estimates for parent reports of 'psychomotor functions', 'basic interpersonal interactions' and 'emotional functions' were all high and had 95% confidence intervals that did not overlap with zero (h^2_{pooled} range: .49–.67). Conversely, heritability estimates for observer ratings of the same three phenotypic categories were smaller and had confidence intervals that overlapped with zero (h^2_{pooled} range: .20–.35).

Shared Environment. The shared environmental estimate for parent-reports of 'emotional functions' was small but had a confidence interval that overlapped with zero (c^2_{pooled} = .17; 95% CI [.01–.35] p = .033). The remaining two parent-report subgroups ('psychomotor functions' and 'basic interpersonal interactions') both had small shared environmental estimates with 95% confidence intervals overlapping with zero (c^2_{pooled}: .00 and .15, respectively) as did the three observer-reported subgroups c^2_{pooled} range: .12–.21). Comparable shared environment estimates in parent and observer ratings might be interpreted as indicating limited rater bias linked to shared environment. However, this comparison is likely confounded by other influences and is complicated by the fact that, in theory, studies may have had the same observer rating both twins (although, generally speaking, this is unusual, and it is hard to estimate in our specific sample as this information was typically not provided by the included studies).

Nonshared Environment. Nonshared environmental estimates for parent and observer reports of 'psychomotor functions', 'basic interpersonal interactions' and 'emotional functions' were all statistically significant. The observer-report subgroups each had higher nonshared environmental estimates (e^2_{pooled} range: .45.47) than the parent-report subgroups (e^2_{pooled} range: .30.33).

Heterogeneity. As displayed in eTable 9, a proportionally small amount of the total variance was attributed to sampling variance in each of the six observer- or parent-report subgroups ($I^2_{Level\ 1}$ range = 0.88%–18.76%). Observer ratings of 'psychomotor functions' and 'emotional functions', and parent ratings of 'emotional functions', each had moderate to substantial within-cohort heterogeneity ($I^2_{Level\ 2}$ range = 43.14%–94.31%). Conversely, parent reports of 'psychomotor functions' and 'basic interpersonal interactions', and observer reports of 'basic interpersonal interactions', each had low within-cohort heterogeneity ($I^2_{Level\ 2}$ range = 2.92%–32.18%). Parent reported 'emotional functions' had between-cohort heterogeneity of 0% and the remaining five observer- and parent-reported categories all had moderate to substantial between cohort heterogeneity ($I^2_{Level\ 3}$ = 40.38%–96.20%).

Publication Bias

Publication bias is present when the likelihood of a finding being published is influenced by the finding itself. For example, if statistically significant findings are more likely to be published than findings that are not statistically significant. We examined publication bias via two methods. First, we created funnel plots, plotting effect sizes against standard errors. These plots are reported in eFigures 14–18. As larger studies typically have lower standard errors than smaller studies, larger studies should appear towards the top of the plot and smaller ones towards the bottom. It is assumed that, in the absence of publication bias, smaller studies with higher standard errors will be scattered symmetrically across the bottom of the plot. In the presence of publication bias it is assumed that plots will be skewed, displaying asymmetry, e.g., there will be more studies with small sample sizes reporting positive than negative results. Based on visual inspection of the funnel plots of rMZ and rDZ in eFigures 14 and 15, there appears to be some asymmetry. Specifically, larger studies tended to publish findings with stronger correlations and smaller studies tended to publish findings with weaker correlations. As displayed in eFigure 15, estimates of h^2 largely displayed no patterns of asymmetry, although for 'psychomotor functions' and 'growth maintenance functions' the smaller studies tended to report lower h^2 estimates. As shown in eFigures 17 and 18, for most of the phenotypic domains, estimates of c^2 and e^2 echoed the same patterns of asymmetry seen in correlations—with smaller studies tending to publish smaller effects. As the interpretation of funnel plots depends on visual examination and is thus somewhat subjective, we also ran the Egger's regression test of publication bias. The Egger's test regresses effect sizes on their standard errors, weighted by their inverse variances. In the absence of publication bias, the regression intercept is expected to be zero and in the presence

of publication bias, it is expected to be significantly different from zero ($p < .05$). Results of Egger's regression test are presented in eTables 10 and 11. Echoing the patterns of skewness in the funnel plots, there was evidence of publication bias for all correlations by category ($p < .001$), apart from *r*DZ in 'attention functions' and 'family relationships' ($p = .061$ and .062). Results suggested potential publication bias in heritability estimates for 'psychomotor' and 'emotional' functions, 'basic interpersonal interactions' and 'complex interpersonal interactions' (*p* range: .013 to < .001) but not 'sleep', 'attention' or 'growth maintenance' functions (*p* range: .072–.385). Findings indicated publication bias for all shared environment estimates ('emotional functions', $p =$ 0.08 and all others $p < .001$), apart from 'attention functions' ($p = .402$). Results suggested publication bias in nonshared environment estimates of 'attention functions' and 'basic interpersonal interactions' ($p = .036$, .027, respectively) but not 'sleep', 'psychomotor', 'emotional' or 'growth maintenance' functions or 'complex interpersonal interactions' (*p* range: .099–.494). The possible publication bias observed in the funnel plots and Egger's test results was in the opposite direction to the publication bias that is typically hypothesized in research. Usually, it is expected that publication bias will result in more smaller studies publishing large effect sizes due to a bias to publish positive or favorable findings. However, in this case, smaller studies tended to publish findings with smaller effect sizes. These unusual results may reflect that fact that statically significant within-twin correlations and estimates of heritability, shared and non-shared environmental influence do not represent "favorable" results in the same way that statistically significant results might in, for example, intervention research. For example, it is not the statistical significance of a within-twin correlation but, rather, the relationship between within-twin MZ correlations and within-twin DZ correlations that is drawn upon to estimate the sizes of the three variance components. The three variance components each represent the proportion of variation linked to individual differences in a trait. Individually, none of them can be below 0 or above 100 and collectively they must sum to 100. Consequently, these estimates represent something quite different to effect sizes in other research contexts. Additionally, while funnel plot asymmetry and significant Egger's test results indicate possible publication bias, they could be driven by alternative explanations. One particularly plausible possibility, given our highly heterogeneous findings, is that the asymmetry was influenced by between-study heterogeneity, which can also induce skewness in funnel plots.[5]

© 2022 Austerberry C et al. *JAMA Network Open.*

eTable 1. Database and Reference List Searches

Date	Database	Search terms	N[a] publications	N publications (no duplicates)	Includes (T&A[b])	Includes (FT[c])
30/11/18	PubMed	(twin*[Title/Abstract] AND (gene[Title/Abstract] OR genome[Title/Abstract] OR genetic* [Title/Abstract] OR herita* [Title/Abstract] OR environment* [Title/Abstract]) AND (infan*[Title/Abstract] OR early [Title/Abstract]))	2735	2727	199	105
30/11/18	PsycINFO	(twin* and (gene or genome or genetic* or herita* or environment*) and (infan* or early)).ab.	1291	504	47	13
05/02/20	PubMed (from 1/10/18 onwards)	(twin*[Title/Abstract] AND (gene[Title/Abstract] OR genome[Title/Abstract] OR genetic* [Title/Abstract] OR herita* [Title/Abstract] OR environment*[Title/Abstract]) AND (infan*[Title/Abstract] OR early[Title/Abstract]))	315	230	5	1
05/02/20	PsycINFO (From 2018 onwards)	(twin* and (gene or genome or genetic* or herita* or environment*) and (infan* or early)).ab.	120	28	0	0
11/02/21	PubMed (from 1/2/20 onwards)	(twin*[Title/Abstract] AND (gene[Title/Abstract] OR genome[Title/Abstract] OR genetic* [Title/Abstract] OR herita* [Title/Abstract] OR environment*[Title/Abstract]) AND (infan*[Title/Abstract] OR early[Title/Abstract]))	190	179	4	2
11/02/21	PsycINFO (From 2020 onwards)	(twin* and (gene or genome or genetic* or herita* or environment*) and (infan* or early)).ab.	24	7	2	0
n/a	Reference list searching	n/a	372	98	98	18
			5047	3773	355	139

[a] N = Number of.
[b] T&A = After screening by title and abstract.
[c] FT = After screening by full text.

© 2022 Austerberry C et al. *JAMA Network Open.*

eTable 2. Inclusion and Exclusion Criteria

	Inclusion criteria	Exclusion criteria
**Publication type**	➢ Peer-reviewed journals<br>➢ In English	➢ Not subject to peer review<br>➢ Not published in English
**Study type**	➢ Twin studies	➢ Not reporting twin data<br>➢ Syntheses of previously published twin data (e.g., reviews/meta-analyses)
**Population**	➢ MZ[a] and DZ[b] twins<br>➢ Infants with mean age ≤ 2	➢ Mean age > 2 years<br>➢ MZ twins only<br>➢ Case studies
**Outcome**	➢ Psychological and developmental traits and milestones<br>➢ MZ/DZ correlations<br>➢ ACE estimates based only on data from MZ/DZ twins	➢ Not on psychological and developmental traits and milestones<br>➢ No heritability estimates, twin correlations, or concordances available<br>➢ Twin data not possible to separate from non-twin data<br>➢ Multivariate or gene-environment interaction analyses where univariate estimates not possible to extract

[a] MZ = monozygotic
[b] DZ = dizygotic

© 2022 Austerberry C et al. *JAMA Network Open.*

eTable 3. Information Extracted From Included Publications

Extracted data	Description
Study	Overarching twin study/sample/cohort
Country	Country in which twin population was based
Sex	Whether the sample included male and/or female twins
Phenotype	The examined trait as labelled in the publication
Age	In months, at which data was collected
Rater	Who rated the reported data (e.g., parent, observer)
Measure	How the phenotype was measured
MZ/DZ correlations	Within-twin monozygotic twin correlations and dizygotic twin correlations (based on samples of same-sex DZ twins, opposite-sex DZ twins and combined samples)
ACE estimates	Estimates of heritability (A, or h^2) and shared (C, or c^2) and nonshared (E, or e^2) environmental influence
Number of twin pairs	Entered for each extracted correlation/variance component
Method for estimating variance components	Method used to calculate ACE estimates
Continuous or dichotomous data	Was the measure used to capture the trait continuous or dichotomous
Concordant/discordant pairs	If a dichotomous measure, rates of concordance/discordance for the trait
Prevalence	If a dichotomous measure, trait prevalence rates in the twin sample being examined

© 2022 Austerberry C et al. *JAMA Network Open.*

eTable 4. Publications Identified in Systematic Literature Search, Presented Alphabetically by First Author

Publication	Status	Study	Country	Category (using codes from the ICF-CY[a])	Phenotypes Extracted from Publication
Akerman & Fischbein (1992)[6]	Included in meta-analysis	Project Metropolitan	Sweden	b560 Growth maintenance functions[b]	Height<br>Weight
Ando et al. (2006)[7]	Included in meta-analysis	Tokyo Twin Cohort Project	Japan	b134 Sleep functions<br>b560 Growth maintenance functions<br>d550 Eating[c]<br>d710 Basic interpersonal interactions	Chest circumference<br>Head circumference<br>Height<br>Milk consumption<br>Mimic, point gazing, joint attention<br>Rhythmicity<br>Time to fall asleep<br>Weight
Bakermans-Kranenburg et al. (2004)[8]	Included in meta-analysis	Netherlands Twin Register	The Netherlands	d760 Family relationships	Dependency (to father)<br>Infant-father attachment security
Beaver et al. (2014)[9]	Included in meta-analysis	Early Childhood Longitudinal Study-Birth Cohort	United States	b167 Mental functions of language	Expressive vocabulary
Bishop et al. (2003b)[10]	Excluded from meta-analysis (duplicate data)	The Colorado Twin Registry: Longitudinal Twin Sample	United States	b163 Basic cognitive functions	General cognitive ability

[a] ICF-CY = International Classification of Functioning, Disability and Health, Children and Youth Version.[4] Definitions for each of the categories and subcategories can be found in the cited ICF-CY manual: https://apps.who.int/iris/handle/10665/43737.
[b] The prefix *b* is given to coded items within the ICF-CY component of Body Functions.
[c] The prefix *d* is given to items in the component of Activities and Participation.

© 2022 Austerberry C et al. *JAMA Network Open.*

eTable 4 (Continued)

Publication	Status	Study	Country	Category (using codes from the ICF-CY)	Phenotypes Extracted from Publication
Bokhorst et al. (2003)[11]	Included in meta-analysis	Netherlands Twin Register and Multiple Births Foundation of Queen Charlotte's and Chelsea Hospital in London	The Netherlands; UK	b152 Emotional functions<br>d760 Family relationships	Attachment disorganization<br>Attachment security<br>Temperamental reactivity
Boomsma et al. (1992)[12]	Excluded from meta-analysis (duplicate data)	Netherlands Twin Register	The Netherlands	b560 Growth maintenance functions	Height<br>Weight
Brant et al. (2009)[13]	Included in meta-analysis	Colorado Twin Registry: Longitudinal Twin Sample	United States	b163 Basic cognitive functions	General cognitive ability
Brescianini et al. (2011)[14]	Included in meta-analysis	Italian Twin Register	Italy	b134 Sleep functions	Cosleeping<br>Diurnal sleep duration<br>Night awakenings<br>Nocturnal sleep duration
Brescianini et al. (2012)[15]	Included in meta-analysis	Italian Twin Register	Italy	b560 Growth maintenance functions	Weight gain
Caramaschi et al. (2012)[16]	Excluded from meta-analysis (phenotype[s] could not be categorized)	Quebec Newborn Twin Study	Canada	N/A	Testosterone

© 2022 Austerberry C et al. *JAMA Network Open.*

eTable 4 (Continued)

Publication	Status	Study	Country	Category (using codes from the ICF-CY)	Phenotypes Extracted from Publication
Chen et al. (1990a)[17]	Included in meta-analysis	Taipei City Teaching Hospitals Twin Study	Taiwan	b125 Dispositions and intra-personal functions<br>b140 Attention functions<br>b147 Psychomotor functions<br>b152 Emotional functions<br>d710 Basic interpersonal interactions<br>d720 Complex interpersonal interactions	Activity level<br>Adaptability<br>Approach/withdrawal<br>Attention/persistence<br>Distractibility<br>Intensity of reaction<br>Quality of mood<br>Rhythmicity<br>Threshold of responsiveness
Chen et al. (1990b)[18]	Included in meta-analysis	Taipei City Teaching Hospitals Twin Study	Taiwan	b560 Growth maintenance functions	Arm circumference<br>Chest circumference<br>Head circumference<br>Height<br>Weight
Cherny et al. (1992)[19]	Excluded from meta-analysis (duplicate data)	The Colorado Twin Registry: Longitudinal Twin Sample	United States	b163 Basic cognitive functions	Cognitive ability
Cherny et al. (1994a)[20]	Included in meta-analysis	Colorado Twin Registry: Longitudinal Twin Sample	United States	d710 Basic interpersonal interactions	Shyness
Cherny et al. (1994b)[21]	Excluded from meta-analysis (duplicate data)	The Colorado Twin Registry: Longitudinal Twin Sample	United States	b163 Basic cognitive functions	Cognitive ability
Custodio et al. (2007)[22]	Included in meta-analysis	University of Sao Paulo - Longitudinal Twin Study	Brazil	b134 Sleep functions	Emergence of the cortisol circadian rhythm
Dale et al. (2000)[23]	Included in meta-analysis	Twins Early Development Study	UK	b167 Mental functions of language	Grammar<br>Vocabulary

© 2022 Austerberry C et al. *JAMA Network Open.*

eTable 4 (Continued)

Publication	Status	Study	Country	Category (using codes from the ICF-CY)	Phenotypes Extracted from Publication
Davis et al. (2015)[24]	Included in meta-analysis	Louisville Twin Study	United States	b140 Attention functions<br>b147 Psychomotor functions<br>d710 Basic interpersonal interactions	Activity level<br>Affect-extraversion<br>Task orientation
DiLalla & Bishop (1996)[25]	Included in meta-analysis	Colorado Twin Registry: Twin Infant Project	United States	b140 Attention functions<br>b152 Emotional functions<br>b310 Voice functions<br>d710 Basic interpersonal interactions	Affection for mother<br>Enthusiasm for interaction with mother<br>Negative affect<br>Task orientation<br>Vocalize<br>Watch mother
Dilalla et al. (1994)[26]	Excluded from meta-analysis (duplicate data)	The Colorado Twin Registry: Longitudinal Twin Sample	United States	d710 Basic interpersonal interactions	Behavioral Inhibition
Dionne et al (2003a)[27]	Excluded from meta-analysis (duplicate data)	Twins Early Development Study	UK	b167 Mental functions of language	Grammar<br>Vocabulary
Dionne et al. (2003b)[28]	Included in meta-analysis	Quebec Newborn Twin Study	Canada	b167 Mental functions of language<br>d720 Complex interpersonal interactions	Expressive vocabulary<br>Physical aggression
Dionne et al. (2011)[29]	Included in meta-analysis	Quebec Newborn Twin Study	Canada	b134 Sleep functions<br>b167 Mental functions of language	Ratio of day/night sleep duration<br>Vocabulary
Dubois et al. (2007)[30]	Included in meta-analysis	Quebec Newborn Twin Study	Canada	b560 Growth maintenance functions	Height<br>Weight

© 2022 Austerberry C et al. *JAMA Network Open.*

eTable 4 (Continued)

Publication	Status	Study	Country	Category (using codes from the ICF-CY)	Phenotypes Extracted from Publication
Dubois et al. (2012)[31]	Included in meta-analysis	Quebec Newborn Twin Study, Child and Adolescent Twin Study in Sweden, Twin Study of Child and Adolescent Development, Danish Twin Registry, and Brisbane Longitudinal Twin Study	Canada, Sweden, Denmark, Australia	b560 Growth maintenance functions	BMI<br>Height<br>Weight
Emde et al. (1992)[32]	Included in meta-analysis	Colorado Twin Registry: Longitudinal Twin Sample	United States	b140 Attention functions<br>b144 Memory functions<br>b147 Psychomotor functions<br>b152 Emotional functions<br>b164 Higher-level cognitive functions<br>b167 Mental functions of language<br>d710 Basic interpersonal interactions	Activity<br>Affect<br>Attention/persistence<br>Behavioral Inhibition<br>Categorization<br>Emotionality<br>Empathy<br>Expressive language<br>Frustration<br>Memory for location<br>Negative affect<br>Negative hedonic tone<br>Overall mood<br>Positive affect<br>Positive hedonic tone<br>Receptive language<br>Shyness<br>Sociability<br>Task orientation<br>Word comprehension

© 2022 Austerberry C et al. *JAMA Network Open.*

eTable 4 (Continued)

Publication	Status	Study	Country	Category (using codes from the ICF-CY)	Phenotypes Extracted from Publication
Finkel et al. (2000)[33]	Included in meta-analysis	Louisville Twin Study	United States	d760 Family relationships	Attachment
Fisher et al. (2012)[34]	Included in meta-analysis	Gemini Study	UK	b134 Sleep functions	Daytime nap duration<br>Night awakenings<br>Nighttime sleep duration<br>Wake time
Flom & Saudino (2017)[35]	Excluded from meta-analysis (duplicate data)	Boston University Twin Project	United States	d710 Basic interpersonal interactions	Callous Unemotional Behavior
Flom & Saudino (2018)[36]	Included in meta-analysis	Boston University Twin Project	United States	b140 Attention functions<br>d710 Basic interpersonal interactions<br>d720 Complex interpersonal interactions	ADHD<br>Callous Unemotional Behavior<br>ODD
Flom et al. (2019)[37]	Included in meta-analysis	Boston University Twin Project	United States	d710 Basic interpersonal interactions	Callous unemotional traits
Forget-Dubois et al. (2007)[38]	Included in meta-analysis	Quebec Newborn Twin Study	Canada	d720 Complex interpersonal interactions	Difficult temperament<br>Disruptive behavior
Friedman et al. (2011)[39]	Excluded from meta-analysis (phenotype in category containing < 5 independent samples)	Colorado Twin Registry: Longitudinal Twin Sample	United States	b164 Higher-level cognitive functions	Self-restraint
Fujisawa et al. (2012)[40]	Included in meta-analysis	Tokyo Twin Cohort Project	Japan	b560 Growth maintenance functions<br>d710 Basic interpersonal interactions	Head circumference<br>Head circumference growth<br>Sociocognitive abilities
Gagne & Goldsmith (2011)[41]	Included in meta-analysis	Wisconsin Twin Panel	United States	b152 Emotional functions	Anger<br>Distress to limitations

© 2022 Austerberry C et al. *JAMA Network Open.*

eTable 4 (Continued)

Publication	Status	Study	Country	Category (using codes from the ICF-CY)	Phenotypes Extracted from Publication
Gagne & Saudino (2010)[42]	Included in meta-analysis	Boston University Twin Project	United States	b164 Higher-level cognitive functions	Inhibitory control
Gagne et al. (2011)[43]	Included in meta-analysis	Boston University Twin Project	United States	b140 Attention functions<br>b164 Higher-level cognitive functions<br>d720 Complex interpersonal interactions	ADHD<br>Externalizing<br>Inhibitory control
Gagne & Saudino (2016)[44]	Excluded from meta-analysis (duplicate data)	Boston University Twin Project	United States	b164 Higher-level cognitive functions	Inhibitory control
Gagne et al. (2020)[45]	Excluded from meta-analysis (duplicate data and phenotype in category containing < 5 independent samples)	Boston University Twin Project	United States	b140 Attention functions<br>b164 Higher-level cognitive functions	ADHD<br>Inhibitory control
Galsworthy et al. (2000)[46]	Excluded from meta-analysis (duplicate data)	Twins Early Development Study	UK	b163 Basic cognitive functions<br>b167 Mental functions of language	Non-verbal cognitive development<br>Verbal ability
German et al. (2015)[47]	Included in meta-analysis	Childcare Centers of Tel Aviv and Haifa	Israel	b560 Growth maintenance functions	Age at transition to childhood

© 2022 Austerberry C et al. *JAMA Network Open.*

eTable 4 (Continued)

Publication	Status	Study	Country	Category (using codes from the ICF-CY)	Phenotypes Extracted from Publication
Gilmore et al. (2010)[48]	Excluded from meta-analysis (phenotypes in category containing < 5 independent samples)	UNC Early Brain Development Study	United States	s110 Structure of brain[d]	Cerebellum<br>Corpus Callosum<br>Cortical grey matter<br>Cortical unmyelinated white matter<br>Frontal grey matter<br>Frontal unmyelinated white matter<br>Intracranial volume<br>Lateral Ventricles<br>Left hemisphere grey matter<br>Left hemisphere total<br>Left hemisphere unmyelinated white matter<br>Occipital grey matter<br>Occipital unmyelinated white matter<br>Parietal grey matter<br>Parietal unmyelinated white matter<br>Prefrontal grey matter<br>Prefrontal unmyelinated white matter<br>Right hemisphere grey matter<br>Right hemisphere total<br>Right hemisphere unmyelinated white matter<br>Subcortical grey matter<br>Total cerebrospinal fluid<br>Total early myelinated white matter<br>Total Frontal<br>Total grey matter<br>Total Occipital<br>Total Parietal<br>Total Prefrontal<br>Total unmyelinated white matter

[d] The prefix *s* is given to items in the ICF-CY component of Body Structures.

© 2022 Austerberry C et al. *JAMA Network Open.*

eTable 4 (Continued)

Publication	Status	Study	Country	Category (using codes from the ICF-CY)	Phenotypes Extracted from Publication
Goetghebuer et al. (2003)[49]	Included in meta-analysis	Twin study in The Gambia	The Gambia	b147 Psychomotor functions<br>b560 Growth maintenance functions	Crawl<br>Length<br>Maintain head<br>Roll over<br>Sitting<br>Sitting without support<br>Stand holding on something<br>Take two steps<br>Walk holding on something<br>Weight
Goldsmith & Gottesman (1981)[50]	Included in meta-analysis	Collaborative Perinatal Project	United States	b125 Dispositions and intra-personal functions<br>b140 Attention functions<br>b147 Psychomotor functions<br>b560 Growth maintenance functions<br>d710 Basic interpersonal interactions	Active manipulation<br>Activity level<br>Degree of social acceptance of examiner<br>Degree of social contact with mother<br>Interest in persons<br>Interest in/responsiveness to people<br>Physical development<br>Pursuit persistence<br>Response duration<br>Speed of response<br>Vigorous activity vs. psychomotor passivity
Goldsmith et al. (1999)[51]	Included in meta-analysis	Pooled Sample of twins from Oregon, Washington, Colorado, Texas, and Wisconsin	United States	b140 Attention functions<br>b147 Psychomotor functions<br>b152 Emotional functions	Activity level<br>Distress to limitations<br>Distress to novelty<br>Duration of orienting<br>Negative affect<br>Positive affect<br>Resistance to soothing<br>Smiling and laughter

© 2022 Austerberry C et al. *JAMA Network Open.*

eTable 4 (Continued)

Publication	Status	Study	Country	Category (using codes from the ICF-CY)	Phenotypes Extracted from Publication
Hawks & Marrus (2018)[52]	Included in meta-analysis	Early Reciprocal Social Behavior Study	United States	d710 Basic interpersonal interactions<br>d720 Complex interpersonal interactions	Behavior problems<br>Competence<br>Reciprocal social behavior
Herle et al. (2018)[53]	Excluded from meta-analysis (phenotype in category containing < 5 independent samples)	Gemini Study	UK	d550 Eating	Emotional overeating
Hur (2005)[54]	Excluded from meta-analysis (duplicate data)	The South Korean Twin Registry (Previously the Seoul Twin Family Study)	South Korea	b560 Growth maintenance functions	Weight

© 2022 Austerberry C et al. *JAMA Network Open.*

eTable 4 (Continued)

Publication	Status	Study	Country	Category (using codes from the ICF-CY)	Phenotypes Extracted from Publication
Hur et al. (2005)[55]	Included in meta-analysis	Brisbane Adolescent Twin Study<br>Japanese pooled sample: (1) Registry of twins recruited in Japan from associations for parents of multiples; (2) Registry of twins who applied for the secondary school attached to the Faculty of Education at the University of Tokyo between 1981 and 2003.<br>Minnesota Twin Family Study<br>South Korean pooled sample: (1) Twins born in two South Korean hospitals (1998–2003); (2) Seoul Twin Family Study<br>Netherlands Twin Register	Australia, Japan, United States, South Korea, The Netherlands	b560 Growth maintenance functions	Weight
Ilott et al. (2010a)[56]	Included in meta-analysis	Boston University Twin Project	United States	b140 Attention functions<br>b147 Psychomotor functions	Activity Level<br>ADHD

© 2022 Austerberry C et al. *JAMA Network Open.*

eTable 4 (Continued)

Publication	Status	Study	Country	Category (using codes from the ICF-CY)	Phenotypes Extracted from Publication
Ilott et al. (2010b)[57]	Excluded from meta-analysis (duplicate data)	Boston University Twin Project	United States	b140 Attention functions	ADHD
Jackson (2016)[58]	Included in meta-analysis	Early Childhood Longitudinal Study-Birth Cohort	United States	b152 Emotional functions<br>d710 Basic interpersonal interactions<br>d720 Complex interpersonal interactions<br>d760 Family relationships	Attachment security<br>Avoids others/not sociable<br>Comfortable cuddly<br>Cooperative<br>Demanding/angry<br>Dependency<br>Enjoys company<br>Independent<br>Moody/unusual<br>Seeks attention<br>Upset by separation
Jha et al. (2018)[59]	Excluded from meta-analysis (phenotypes in category containing < 5 independent samples)	UNC Early Brain Development Study	United States	s110 Structure of brain	Cortical surface area<br>Cortical thickness<br>Intracranial volume
Johnson et al. (2011)[60]	Included in meta-analysis	Gemini Study	UK	b560 Growth maintenance functions	Size (weight)<br>Tempo (weight)<br>Weight<br>Weight change<br>Weight velocity
Knafo & Plomin (2006)[61]	Included in meta-analysis	Twins Early Development Study	UK	d710 Basic interpersonal interactions	Prosocial behavior

© 2022 Austerberry C et al. *JAMA Network Open.*

eTable 4 (Continued)

Publication	Status	Study	Country	Category (using codes from the ICF-CY)	Phenotypes Extracted from Publication
Koeppen-Schomerus et al. (2003)[62]	Included in meta-analysis	Twins Early Development Study	UK	b163 Basic cognitive functions<br>b167 Mental functions of language<br>d720 Complex interpersonal interactions	Behavior problems<br>General cognitive ability<br>Nonverbal cognitive ability<br>Verbal ability
Kuntsi et al. (2005)[63]	Included in meta-analysis	Twins Early Development Study	UK	b147 Psychomotor functions	Hyperactivity
Lacourse et al. 2014[64]	Included in meta-analysis	Quebec Newborn Twin Study	Canada	d720 Complex interpersonal interactions	Physical aggression
Levine et al. (1987)[65]	Included in meta-analysis	Jackson Memorial Hospital/University of Miami Medical Center	United States	b560 Growth maintenance functions	Length<br>Weight
Liu et al. (2015)[66]	Included in meta-analysis	Chinese National Twin Registry	China	b560 Growth maintenance functions	BMI<br>Height<br>Weight
Livshits et al. (2000)[67]	Included in meta-analysis	Childcare Centers of Tel Aviv and Haifa	Israel	b560 Growth maintenance functions	Head circumference<br>Head circumference (growth curve A)<br>Head circumference (growth curve B)<br>Head circumference (growth curve C)<br>Height<br>Height (growth curve A)<br>Height (growth curve B)<br>Height (growth curve C)<br>Weight<br>Weight (growth curve A)<br>Weight (growth curve B)<br>Weight (growth curve C)

© 2022 Austerberry C et al. *JAMA Network Open.*

eTable 4 (Continued)

Publication	Status	Study	Country	Category (using codes from the ICF-CY)	Phenotypes Extracted from Publication
Llewellyn et al. (2010)[68]	Excluded from meta-analysis (phenotypes in category containing < 5 independent samples)	Gemini Study	UK	d550 Eating	Enjoyment of food<br>Food responsiveness<br>Satiety responsiveness<br>Slowness in eating
Marrus et al. (2015)[69]	Included in meta-analysis	Early Reciprocal Social Behavior Study	United States	d710 Basic interpersonal interactions	Reciprocal social behavior<br>Restrictive repetitive behavior
Marrus et al. (2018)[70]	Included in meta-analysis	Early Reciprocal Social Behavior Study	United States	d710 Basic interpersonal interactions	Reciprocal social behavior
Marrus et al. (2020)[71]	Included in meta-analysis	Early Reciprocal Social Behavior Study	United States	d710 Basic interpersonal interactions	Functional communication<br>Restrictive repetitive behavior<br>Social avoidance<br>Social motivation<br>Social orienting
Matheny (1980)[72]	Included in meta-analysis	Louisville Twin Study	United States	b140 Attention functions<br>b147 Psychomotor functions<br>b279 Additional sensory functions, other specified and unspecified<br>d710 Basic interpersonal interactions	Activity level<br>Activity Level<br>Affect-extraversion<br>Auditory-visual<br>Motor development<br>Task orientation
Matheny (1983)[73]	Included in meta-analysis	Louisville Twin Study	United States	b140 Attention functions<br>b147 Psychomotor functions	Activity level<br>Task orientation

© 2022 Austerberry C et al. *JAMA Network Open.*

eTable 4 (Continued)

Publication	Status	Study	Country	Category (using codes from the ICF-CY)	Phenotypes Extracted from Publication
Matheny (1984)[74]	Excluded from meta-analysis (phenotype[s] could not be categorized)	Louisville Twin Study	United States	N/A	General temperament
Matheny (1989)[75]	Included in meta-analysis	Louisville Twin Study	United States	b152 Emotional functions<br>d710 Basic interpersonal interactions	Approach/withdrawal<br>Behavioral inhibition<br>Emotional tone<br>Fearfulness
Matheny et al. (1976)[76]	Included in meta-analysis	Louisville Twin Study	United States	b140 Attention functions<br>b147 Psychomotor functions<br>b152 Emotional functions<br>b163 Basic cognitive functions<br>b310 Voice functions<br>d710 Basic interpersonal interactions<br>d720 Complex interpersonal interactions	Attention<br>Banging<br>Cooperative<br>Emotional tone<br>Endurance<br>Energy<br>Extraversion<br>Fearfulness<br>Fine motor<br>Goal directedness<br>Gross motor<br>Listening<br>Looking<br>Manipulating<br>Mouthing: pacifier<br>Mouthing: thumb<br>Mouthing: toys<br>Object orientation<br>Primary cognition<br>Reactivity<br>Tension<br>Vocalize
Micalizzi et al. (2016)[77]	Included in meta-analysis	Boston University Twin Project	United States	b152 Emotional functions	Affective problems<br>Autistic-like traits

© 2022 Austerberry C et al. *JAMA Network Open.*

eTable 4 (Continued)

Publication	Status	Study	Country	Category (using codes from the ICF-CY)	Phenotypes Extracted from Publication
Micalizzi et al. (2017)[78]	Included in meta-analysis	Boston University Twin Project	United States	d720 Complex interpersonal interactions	Difficult temperament
Mook-Kanamori et al. (2012)[79]	Included in meta-analysis	Netherlands Twin Register	The Netherlands	b560 Growth maintenance functions	Height<br>Length<br>Weight
Nguyen et al. (2008)[80]	Included in meta-analysis	Quebec Newborn Twin Study	Canada	b134 Sleep functions	Sleep terrors
Nichols et al. (1974)[81]	Included in meta-analysis	Collaborative Perinatal Project	United States	b163 Basic cognitive functions	Mental development

© 2022 Austerberry C et al. *JAMA Network Open.*

eTable 4 (Continued)

Publication	Status	Study	Country	Category (using codes from the ICF-CY)	Phenotypes Extracted from Publication
Orekhova et al. (2003)[82]	Included in meta-analysis	Moscow City Twin Sample	Russia	b140 Attention functions	Alpha frequency during darkness<br>EEG mu gravity frequency during visual attention<br>EEG spectral amplitude during visual attention: Alpha AF3<br>EEG spectral amplitude during visual attention: Alpha AF4<br>EEG spectral amplitude during visual attention: Alpha F7<br>EEG spectral amplitude during visual attention: Alpha F8<br>EEG spectral amplitude during visual attention: Alpha FC3<br>EEG spectral amplitude during visual attention: Alpha FC4<br>EEG spectral amplitude during visual attention: Alpha O1<br>EEG spectral amplitude during visual attention: Alpha O2<br>EEG spectral amplitude during visual attention: Alpha PO3<br>EEG spectral amplitude during visual attention: Alpha PO4<br>EEG spectral amplitude during visual attention: Alpha T5<br>EEG spectral amplitude during visual attention: Alpha T6<br>EEG spectral amplitude during visual attention: Delta AF3<br>EEG spectral amplitude during visual attention: Delta AF4<br>EEG spectral amplitude during visual attention: Delta F7<br>EEG spectral amplitude during visual attention: Delta F8<br>EEG spectral amplitude during visual attention: Delta FC3<br>EEG spectral amplitude during visual attention: Delta FC4<br>EEG spectral amplitude during visual attention: Delta O1<br>EEG spectral amplitude during visual attention: Delta O2<br>EEG spectral amplitude during visual attention: Delta PO3<br>EEG spectral amplitude during visual attention: Delta PO4<br>EEG spectral amplitude during visual attention: Delta T5<br>EEG spectral amplitude during visual attention: Delta T6<br>EEG spectral amplitude during visual attention: Theta AF3<br>EEG spectral amplitude during visual attention: Theta AF4<br>EEG spectral amplitude during visual attention: Theta F7<br>EEG spectral amplitude during visual attention: Theta F8<br>EEG spectral amplitude during visual attention: Theta FC3<br>EEG spectral amplitude during visual attention: Theta FC4<br>EEG spectral amplitude during visual attention: Theta O1<br>EEG spectral amplitude during visual attention: Theta O2<br>EEG spectral amplitude during visual attention: Theta PO3<br>EEG spectral amplitude during visual attention: Theta PO4<br>EEG spectral amplitude during visual attention: Theta T5<br>EEG spectral amplitude during visual attention: Theta T6<br>Spectral amplitude during darkness

© 2022 Austerberry C et al. *JAMA Network Open.*

eTable 4 (Continued)

Publication	Status	Study	Country	Category (using codes from the ICF-CY)	Phenotypes Extracted from Publication
Ouellet-Morin et al. (2008)[83]	Included in meta-analysis	Quebec Newborn Twin Study	Canada	b152 Emotional functions	Cortisol reactivity
Ouellet-Morin et al. (2009)[84]	Included in meta-analysis	Quebec Newborn Twin Study	Canada	b134 Sleep functions	Cortisol secretion in morning<br>Cortisol secretion on awakening
Peter et al. (1999)[85]	Included in meta-analysis	Childcare Centers of Tel Aviv and Haifa	Israel	b147 Psychomotor functions	Pulling up to a standing position<br>Sitting up<br>Turning over<br>Walking five steps
Petitclerc et al. (2011)[86]	Included in meta-analysis	Quebec Newborn Twin Study	Canada	d720 Complex interpersonal interactions	Disregard for rules
Pimpin et al. (2013)[87]	Excluded from meta-analysis (phenotypes in category containing < 5 independent samples)	Gemini Study	UK	d550 Eating	Carbohydrate intake<br>Energy intake<br>Fat intake<br>Food weight<br>Protein intake
Planalp et al. (2017)[88]	Included in meta-analysis	Waisman Center Birth to 3 year project	United States	b152 Emotional functions	Positive affect<br>Smiling and laughter

© 2022 Austerberry C et al. *JAMA Network Open.*

eTable 4 (Continued)

Publication	Status	Study	Country	Category (using codes from the ICF-CY)	Phenotypes Extracted from Publication
Plomin & Rowe (1979)[89]	Included in meta-analysis	Denver Twin Sample	United States	d710 Basic interpersonal interactions<br>d760 Family relationships	Approaching mother<br>Approaching stranger<br>Cuddliness with mother<br>Cuddliness with stranger<br>Difference of response between mother and stranger: approach<br>Difference of response between mother and stranger: cuddliness<br>Difference of response between mother and stranger: looking<br>Difference of response between mother and stranger: positive vocalizations<br>Difference of response between mother and stranger: proximity<br>Difference of response between mother and stranger: quality of play<br>Difference of response between mother and stranger: smiling<br>Difference of response between mother and stranger: touches<br>Latency to approach stranger<br>Looking at mother<br>Looking at stranger<br>Positive vocalization to mother<br>Positive vocalization to stranger<br>Proximity to mother<br>Proximity to stranger<br>Quality of play with mother<br>Quality of play with stranger<br>Separation distress<br>Smiling at mother<br>Smiling at stranger<br>Touching mother<br>Touching stranger

© 2022 Austerberry C et al. *JAMA Network Open.*

eTable 4 (Continued)

Publication	Status	Study	Country	Category (using codes from the ICF-CY)	Phenotypes Extracted from Publication
Plomin et al. (1993)[90]	Included in meta-analysis	Colorado Twin Registry: Longitudinal Twin Sample	United States	b140 Attention functions<br>b144 Memory functions<br>b147 Psychomotor functions<br>b152 Emotional functions<br>b163 Basic cognitive functions<br>b164 Higher-level cognitive functions<br>b167 Mental functions of language<br>d710 Basic interpersonal interactions	Activity level<br>Affect<br>Attention/persistence<br>Behavioral Inhibition<br>Behavioral inhibition<br>Categorization<br>Emotionality<br>Empathy<br>Expressive language<br>General cognitive ability<br>Memory for Location<br>Negative affect<br>Overall mood<br>Positive affect<br>Positive hedonic tone<br>Reactivity<br>Receptive language<br>Shyness<br>Sociability<br>Task orientation<br>Word comprehension
Price et al. (2000)[91]	Excluded from meta-analysis (duplicate data)	Twins Early Development Study	UK	b163 Basic cognitive functions<br>b167 Mental functions of language	Nonverbal cognitive development<br>Verbal ability
Price et al. (2005)[92]	Included in meta-analysis	Twins Early Development Study	UK	b140 Attention functions	ADHD

© 2022 Austerberry C et al. *JAMA Network Open.*

eTable 4 (Continued)

Publication	Status	Study	Country	Category (using codes from the ICF-CY)	Phenotypes Extracted from Publication
Pushina et al. (2005)[93]	Excluded from meta-analysis (phenotype in category containing < 5 independent samples)	Moscow City twin sample	Russia	b144 Memory functions	Working memory
Reznick et al (1997)[94]	Included in meta-analysis	Colorado Twin Registry: Longitudinal Twin Sample	United States	b140 Attention functions<br>b144 Memory functions<br>b163 Basic cognitive functions<br>b167 Mental functions of language	Expressive language<br>Memory for Location<br>Nonverbal<br>Receptive language<br>Verbal expressive<br>Verbal receptive<br>Visual attentiveness<br>Word comprehension
Rhee et al. (2007)[95]	Excluded from meta-analysis (duplicate data)	The Colorado Twin Registry: Longitudinal Twin Sample	United States	b152 Emotional functions	Emotionality
Rhee et al. (2012)[96]	Included in meta-analysis	Colorado Twin Registry: Longitudinal Twin Sample	United States	b152 Emotional functions	Negative affect
Rhee et al. (2013)[97]	Included in meta-analysis	Colorado Twin Registry: Longitudinal Twin Sample	United States	d710 Basic interpersonal interactions	Observed Disregard
Rhee et al. (2016)[98]	Included in meta-analysis	Colorado Twin Registry: Longitudinal Twin Sample	United States	d710 Basic interpersonal interactions	Disregard for others

© 2022 Austerberry C et al. *JAMA Network Open.*

eTable 4 (Continued)

Publication	Status	Study	Country	Category (using codes from the ICF-CY)	Phenotypes Extracted from Publication
Rice et al. (2014)[99]	Included in meta-analysis	Western Australian Twin Registry	Australia	b167 Mental functions of language	Combining words<br>Late language acquisition<br>Use of finiteness grammatical markers<br>Words Produced
Riese (1990a)[100]	Included in meta-analysis	Louisville Twin Study	United States	b147 Psychomotor functions<br>b152 Emotional functions<br>b560 Growth maintenance functions<br>d710 Basic interpersonal interactions	Activity-awake<br>Activity-sleep<br>Irritability<br>Reactivity<br>Reinforcement Value<br>Resistance to soothing<br>Weight
Riese (1990b)[101]	Included in meta-analysis	Louisville Twin Study	United States	b147 Psychomotor functions<br>b152 Emotional functions	Activity-awake<br>Activity-sleep<br>Irritability<br>Reactivity<br>Resistance to soothing
Robinson et al. (1992)[102]	Excluded from meta-analysis (duplicate data)	The Colorado Twin Registry: Longitudinal Twin Sample	United States	d710 Basic interpersonal interactions	Behavioral Inhibition
Roisman & Fraley (2006)[103]	Included in meta-analysis	Early Childhood Longitudinal Study-Birth Cohort	United States	b152 Emotional functions	Fussiness and demanding behavior<br>Positive and negative affect
Roisman & Fraley (2008)[104]	Excluded from meta-analysis (duplicate data)	Early Childhood Longitudinal Study-Birth Cohort	United States	d760 Family relationships	Attachment security<br>Temperamental dependency
Ronald et al. (2010)[105]	Included in meta-analysis	Boston University Twin Project	United States	b140 Attention functions<br>d710 Basic interpersonal interactions	ADHD<br>Autistic-like traits<br>Non-social autistic-like traits<br>Social autistic-like traits
Saudino (2012)[106]	Included in meta-analysis	Boston University Twin Project	United States	b147 Psychomotor functions	Activity level

© 2022 Austerberry C et al. *JAMA Network Open.*

eTable 4 (Continued)

Publication	Status	Study	Country	Category (using codes from the ICF-CY)	Phenotypes Extracted from Publication
Saudino & Zapfe (2008)[107]	Excluded from meta-analysis (duplicate data)	Boston University Twin Project	United States	b147 Psychomotor functions	Activity Level Home<br>Activity Level Lab<br>Activity Level Play
Saudino & Eaton (1991)[108]	Included in meta-analysis	Manitoba Twin Study	Canada	b147 Psychomotor functions<br>b560 Growth maintenance functions	Activity level<br>Head circumference<br>Length<br>Motor development<br>Ponderal index<br>Weight
Saudino et al. (1996)[109]	Included in meta-analysis	Colorado Twin Registry: Longitudinal Twin Sample	United States	b140 Attention functions<br>b147 Psychomotor functions<br>d710 Basic interpersonal interactions	Activity level<br>Affect-extraversion<br>Task orientation

© 2022 Austerberry C et al. *JAMA Network Open.*

eTable 4 (Continued)

Publication	Status	Study	Country	Category (using codes from the ICF-CY)	Phenotypes Extracted from Publication
Saudino et al. (2008)[110]	Included in meta-analysis	Jumeaux et plus	France	b134 Sleep functions<br>b140 Attention functions<br>b147 Psychomotor functions<br>b152 Emotional functions<br>b279 Additional sensory functions, other specified and unspecified<br>d550 Eating<br>d710 Basic interpersonal interactions<br>d720 Complex interpersonal interactions<br>d760 Family relationships	Activity/Impulsivity<br>Aggression/Defiance<br>Attention<br>Atypical Index<br>Competence<br>Compliance<br>Depression withdrawal<br>Dysregulation<br>Eating problems<br>Empathy<br>Externalizing<br>General anxiety<br>Imitation/Play<br>Inhibition to novelty<br>Internalizing<br>Maladaptive behaviors<br>Mastery Motivation<br>Negative affect<br>Peer aggression<br>Prosocial peer relations<br>Sensory Sensitivity<br>Separation distress<br>Sleep problems<br>Social relatedness
Saudino et al. (2018)[111]	Included in meta-analysis	Boston University Twin Project	United States	b140 Attention functions<br>b147 Psychomotor functions	Activity level<br>Attention problems
Schmitz et al. (1999)[112]	Included in meta-analysis	Colorado Twin Registry: Longitudinal Twin Sample	United States	b152 Emotional functions<br>d710 Basic interpersonal interactions	Emotionality<br>Shyness
Schumann et al. (2017)[113]	Included in meta-analysis	Quebec Newborn Twin Study	Canada	b152 Emotional functions	Negative affect

© 2022 Austerberry C et al. *JAMA Network Open.*

eTable 4 (Continued)

Publication	Status	Study	Country	Category (using codes from the ICF-CY)	Phenotypes Extracted from Publication
Silberg et al. (2005)[114]	Included in meta-analysis	Puerto Rican Infant Twin Study	United States	d710 Basic interpersonal interactions<br>d720 Complex interpersonal interactions	Difficult temperament<br>Unadaptability<br>Unsociability
Silberg et al. (2015)[115]	Included in meta-analysis	Puerto Rican Infant Twin Study	United States	d710 Basic interpersonal interactions<br>d720 Complex interpersonal interactions	Difficultness<br>Inhibition<br>Resistance to control<br>Sociability
Silventoinen et al. (2007)[116]	Included in meta-analysis	Swedish Young Male Twins Study	Sweden	b560 Growth maintenance functions	BMI
Silventoinen et al. (2008)[117]	Included in meta-analysis	Swedish Young Male Twins Study	Sweden	b560 Growth maintenance functions	Height
Silventoinen et al. (2011a)[118]	Included in meta-analysis	West Japan Twins and Higher Order Multiple Births Registry	Japan	b560 Growth maintenance functions	Head circumference
Silventoinen et al. (2011b)[119]	Included in meta-analysis	West Japan Twins and Higher Order Multiple Births Registry	Japan	b560 Growth maintenance functions	Height
Silventoinen et al. (2012)[120]	Included in meta-analysis	West Japan Twins and Higher Order Multiple Births Registry	Japan	b560 Growth maintenance functions	Chest Circumference<br>Chest circumference increase

© 2022 Austerberry C et al. *JAMA Network Open.*

eTable 4 (Continued)

Publication	Status	Study	Country	Category (using codes from the ICF-CY)	Phenotypes Extracted from Publication
Silventoinen et al. (2016)[121]	Included in meta-analysis	The CODATwins Project: Boston University Twin Project; Gemini Study; Guinea-Bissau Twin Study; Hungarian Twin Registry; Italian Twin Registry; Japanese Twin Cohort; Michigan State University Twin Registry; Mongolian Twin Registry; Netherlands Twin Registry; Peri/Postnatal Epigenetic Twins Study; Quebec Newborn Twin Study; Swedish Young Male Twins Study; Twins Early Development Study; West Japan Twins and Higher Order Multiple Births Registry	Canada, Guinea-Bissau, Hungary, Italy, Japan, Mongolia, Sweden, The Netherlands, United Kingdom, United States	b560 Growth maintenance functions	BMI
Smit et al. (2010)[122]	Included in meta-analysis	Netherlands Twin Register	The Netherlands	b560 Growth maintenance functions	Head circumference

© 2022 Austerberry C et al. *JAMA Network Open.*

eTable 4 (Continued)

Publication	Status	Study	Country	Category (using codes from the ICF-CY)	Phenotypes Extracted from Publication
Smith et al. (2012)[123]	Included in meta-analysis	Colorado Twin Registry: Longitudinal Twin Sample	United States	d710 Basic interpersonal interactions	Behavioral inhibition
Smith et al. (2017a)[124]	Excluded from meta-analysis (phenotypes in category containing < 5 independent samples)	Gemini Study	UK	d550 Eating	Food fussiness<br>Food neophobia
Smith et al. (2017b)[125]	Included in meta-analysis	Gemini Study	UK	b147 Psychomotor functions	Activity level<br>First Crawl<br>First Sit<br>First Steps
Soussignan et al. (2009)[126]	Included in meta-analysis	Quebec Newborn Twin Study	Canada	d710 Basic interpersonal interactions	Emotional response to social stimuli<br>Gaze aversion<br>Motor activity during social stimuli<br>Self-contact during social stimuli<br>Social gaze
Spinath et al. (2003)[127]	Included in meta-analysis	Twins Early Development Study	UK	b163 Basic cognitive functions	General cognitive ability
Stevenson & Fielding (1985)[128]	Included in meta-analysis	Department of Psychology, University of Surrey	UK	b147 Psychomotor functions<br>b152 Emotional functions<br>b164 Higher-level cognitive functions<br>d710 Basic interpersonal interactions	Activity level<br>Emotionality<br>Impulsivity<br>Sociability

© 2022 Austerberry C et al. *JAMA Network Open.*

eTable 4 (Continued)

Publication	Status	Study	Country	Category (using codes from the ICF-CY)	Phenotypes Extracted from Publication
Stroganova et al. (2000)[129]	Included in meta-analysis	Moscow City Twin Sample	Russia	b140 Attention functions<br>b152 Emotional functions<br>b164 Higher-level cognitive functions<br>b279 Additional sensory functions, other specified and unspecified<br>d710 Basic interpersonal interactions<br>d720 Complex interpersonal interactions<br>d760 Family relationships	Aggression towards father<br>Aggression towards mother<br>Autonomy<br>Control<br>Defensive reactions<br>Dependence on mother<br>Fear<br>High tension<br>Imitation of father<br>Imitation of mother<br>Love for father<br>Love for mother<br>Low tension<br>Moderate tension<br>Nonoriented discharges<br>Obedience to mother<br>Obedience to father<br>Object orientation<br>Orientation to humans<br>Passiveness<br>Reaction to father<br>Reaction to mother<br>Reaction to mother's punishment<br>Unpleasant sensations
Touchette et al. (2013)[130]	Included in meta-analysis	Quebec Newborn Twin Study	Canada	b134 Sleep functions	Daytime continuous sleep duration<br>Night-time continuous sleep duration
Touwslager et al. (2011a)[131]	Excluded from meta-analysis (duplicate data)	East Flanders Prospective Twin Survey	Belgium	b560 Growth maintenance functions	Growth in weight
Touwslager et al. (2011b)[132]	Included in meta-analysis	East Flanders Prospective Twin Survey	Belgium	b560 Growth maintenance functions	Weight gain

© 2022 Austerberry C et al. *JAMA Network Open.*

eTable 4 (Continued)

Publication	Status	Study	Country	Category (using codes from the ICF-CY)	Phenotypes Extracted from Publication
Tucker-Drob et al. (2011)[133]	Included in meta-analysis	Early Childhood Longitudinal Study-Birth Cohort	United States	b163 Basic cognitive functions	Mental ability
van Dommelen et al. (2004)[134]	Included in meta-analysis	Netherlands Twin Register	Netherlands	b560 Growth maintenance functions	Height<br>Height deceleration<br>Height jerk<br>Height snap<br>Height velocity<br>Weight<br>Weight deceleration<br>Weight jerk<br>Weight snap<br>Weight velocity
Wang & Saudino (2012)[135]	Included in meta-analysis	Boston University Twin Project	United States	b134 Sleep functions	Sleep problems
Whitfield et al. (2001)[136]	Included in meta-analysis	Australian Twin Registry	Australia	b560 Growth maintenance functions	Weight
Wilson (1972)[137]	Excluded from meta-analysis (duplicate data)	Louisville Twin Study	United States	b163 Basic cognitive functions	Cognitive ability
Wilson (1974)[138]	Excluded from meta-analysis (duplicate data)	Louisville Twin Study	United States	b163 Basic cognitive functions	Cognitive ability
Wilson (1978)[139]	Excluded from meta-analysis (duplicate data)	Louisville Twin Study	United States	b163 Basic cognitive functions	Cognitive ability

© 2022 Austerberry C et al. *JAMA Network Open.*

eTable 4 (Continued)

Publication	Status	Study	Country	Category (using codes from the ICF-CY)	Phenotypes Extracted from Publication
Wilson (1983)[140]	Excluded from meta-analysis (duplicate data)	Louisville Twin Study	United States	b163 Basic cognitive functions	Cognitive ability
Wilson (1984)[141]	Included in meta-analysis	Louisville Twin Study	United States	b163 Basic cognitive functions<br>b560 Growth maintenance functions	Cognitive ability<br>Height
Wilson et al. (1972)[142]	Included in meta-analysis	Louisville Twin Study	United States	b147 Psychomotor functions<br>b163 Basic cognitive functions	Cognitive ability<br>Motor development
Wilson & Matheny (1976)[143]	Included in meta-analysis	Louisville Twin Study	United States	b163 Basic cognitive functions	Cognitive ability
Woodward et al. (2018)[144]	Included in meta-analysis	Colorado Twin Registry: Longitudinal Twin Sample; Twin Infant Project	United States	d710 Basic interpersonal interactions	Child affection

© 2022 Austerberry C et al. *JAMA Network Open.*

eTable 5. Twin Studies Identified in Systematic Literature Search, Presented Alphabetically by Study Name

Study name	Country	Age (months)	n twin pairs	Category (using codes from the ICF-CY[a])	Phenotypes (as labeled in included publications)	Publications
Australian Twin Registry	Australia	24	3808 (1799 MZ, 2009 DZ)	b560 Growth maintenance functions[b]	Weight	Whitfield et al. (2001)[136]
Boston University Twin Project	United States	24	314 (145 MZ, 169 DZ)	b134 Sleep functions<br>b140 Attention functions<br>b147 Psychomotor functions<br>b152 Emotional functions<br>b164 Higher-level cognitive functions<br>b560 Growth maintenance functions<br>d710 Basic interpersonal interactions[c]<br>d720 Complex interpersonal interactions	Activity Level (Home/Lab/Play),<br>ADHD,<br>Affective problems,<br>Attention problems,<br>Autistic-like traits,<br>BMI,<br>Callous Unemotional Traits,<br>Difficult temperament,<br>Externalizing,<br>Inhibitory control,<br>Non-social autistic-like traits,<br>ODD,<br>Sleep problems,<br>Social autistic-like traits,	Flom & Saudino (2017)[35]<br>Flom & Saudino (2018)[36]<br>Flom et al. (2019)[37]<br>Gagne & Saudino (2010)[42]<br>Gagne et al. (2011)[43]<br>Gagne & Saudino (2016)[44]<br>Gagne et al. (2020)[45]<br>Ilott et al. (2010a)[56]<br>Ilott et al. (2010b)[57]<br>Micalizzi et al. (2016)[77]<br>Micalizzi et al. (2017)[78]<br>Ronald et al. (2010)[105]<br>Saudino (2012)[106]<br>Saudino & Zapfe (2008)[107]<br>Saudino et al. (2018)[111]<br>Silventoinen et al. (2016)[121]<br>Wang & Saudino (2012)[135]
Brisbane Adolescent Twin Study	Australia	0	1330 (501 MZ, 829 DZ)	b560 Growth maintenance functions	BMI,<br>Height,<br>Weight,	Dubois et al. (2012)[31]<br>Hur et al. (2005)[55]

[a] ICF-CY = International Classification of Functioning, Disability and Health, Children and Youth Version.[4] Definitions for each of the categories and subcategories can be found in the cited ICF-CY manual: https://apps.who.int/iris/handle/10665/43737.
[b] The prefix *b* is given to coded items within the ICF-CY component of Body Functions.
[c] The prefix *d* is given to items in the component of Activities and Participation.

© 2022 Austerberry C et al. *JAMA Network Open.*

eTable 5 (Continued)

Study name	Country	Age (months)	n twin pairs	Category (using codes from the ICF-CY)	Phenotypes (as labeled in included publications)	Publications
Child and Adolescent Twin Study in Sweden	Sweden	0	512 (172 MZ, 340 DZ)	b560 Growth maintenance functions	BMI,<br>Height,<br>Weight,	Dubois et al. (2012)[31]
Childcare Centers of Tel Aviv and Haifa	Israel	0–12	163 (51 MZ, 112 DZ)	b147 Psychomotor functions<br>b560 Growth maintenance functions	Age at transition to childhood<br>Head circumference<br>Head circumference growth<br>Height<br>Height growth<br>Pulling up to a standing position<br>Sitting up<br>Turning over<br>Walking five steps<br>Weight<br>Weight growth	German et al. (2015)[47]<br>Livshits et al. (2000)[67]<br>Peter et al. (1999)[85]
Chinese National Twin Registry	China	0–36	3091 (1448 MZ, 1643 DZ)	b560 Growth maintenance functions	BMI<br>Height<br>Weight	Liu et al. (2015)[66]

© 2022 Austerberry C et al. *JAMA Network Open.*

eTable 5 (Continued)

Study name	Country	Age (months)	n twin pairs	Category (using codes from the ICF-CY)	Phenotypes (as labeled in included publications)	Publications
Collaborative Perinatal Project	United States	8	504 (189 MZ, 315 DZ)	b125 Dispositions and intra-personal functions<br>b140 Attention functions<br>b147 Psychomotor functions<br>b163 Basic cognitive functions<br>b560 Growth maintenance functions<br>d710 Basic interpersonal interactions	Active manipulation<br>Activity level<br>Degree of social acceptance of examiner<br>Degree of social contact with mother<br>Interest in persons<br>Interest in/responsiveness to people<br>Mental development<br>Physical development<br>Pursuit persistence<br>Response duration<br>Speed of response<br>Vigorous activity vs. psychomotor passivity	Goldsmith & Gottesman (1981)[50]<br>Nichols et al. (1974)[81]

© 2022 Austerberry C et al. *JAMA Network Open.*

eTable 5 (Continued)

Study name	Country	Age (months)	n twin pairs	Category (using codes from the ICF-CY)	Phenotypes (as labeled in included publications)	Publications
Colorado Twin Registry: Longitudinal Twin Sample	United States	14, 20, 24, 7–36	887 (494 MZ, 393 DZ)	b140 Attention functions<br>b144 Memory functions<br>b147 Psychomotor functions<br>b152 Emotional functions<br>b163 Basic cognitive functions<br>b164 Higher-level cognitive functions<br>b167 Mental functions of language<br>d710 Basic interpersonal interactions	Activity level<br>Affect<br>Affect-extraversion<br>Attention/persistence<br>Behavioral inhibition<br>Categorization<br>Affection<br>Disregard for others<br>Emotionality<br>Empathy<br>Expressive language<br>Frustration<br>General cognitive ability<br>Memory for Location<br>Negative affect<br>Negative hedonic tone<br>Nonverbal<br>Observed Disregard<br>Overall mood<br>Positive affect<br>Positive hedonic tone<br>Reactivity<br>Receptive language<br>Self-restraint<br>Shyness<br>Sociability<br>Task orientation<br>Verbal expressive<br>Verbal receptive<br>Visual attentiveness<br>Word comprehension	Bishop et al. (2003b)[10]<br>Brant et al. (2009)[13]<br>Cherny et al. (1992)[19]<br>Cherny et al. (1994a)[20]<br>Cherny et al. (1994b)[21]<br>Dilalla et al. (1994)[26]<br>Emde et al. (1992)[32]<br>Friedman et al. (2011)[39]<br>Plomin et al. (1993)[90]<br>Reznick et al (1997)[94]<br>Rhee et al. (2007)[95]<br>Rhee et al. (2012)[96]<br>Rhee et al. (2013)[97]<br>Rhee et al. (2016)[98]<br>Robinson et al. (1992)[102]<br>Saudino et al. (1996)[109]<br>Schmitz et al. (1999)[112]<br>Smith et al. (2012)[123]<br>Woodward et al. (2018)[144]

© 2022 Austerberry C et al. *JAMA Network Open.*

eTable 5 (Continued)

Study name	Country	Age (months)	n twin pairs	Category (using codes from the ICF-CY)	Phenotypes (as labeled in included publications)	Publications
Colorado Twin Registry: Twin Infant Project	United States	7, 9, 7–36	168 (76 MZ, 92 DZ)	b140 Attention functions<br>b152 Emotional functions<br>b310 Voice functions<br>d710 Basic interpersonal interactions	Affection for mother<br>Child affection<br>Enthusiasm for interaction with mother<br>Negative affect<br>Task orientation<br>Watch mother<br>Vocalize	DiLalla & Bishop (1996)[25]<br>Woodward et al. (2018)[144]
Danish Twin Registry	Denmark	0	793 (141 MZ, 652 DZ)	b560 Growth maintenance functions	BMI<br>Height<br>Weight	Dubois et al. (2012)[31]

© 2022 Austerberry C et al. *JAMA Network Open.*

eTable 5 (Continued)

Study name	Country	Age (months)	n twin pairs	Category (using codes from the ICF-CY)	Phenotypes (as labeled in included publications)	Publications
Denver Twin Temperament Study	United States	3–16, 22	70 (35 MZ, 35 DZ)	b140 Attention functions<br>b147 Psychomotor functions<br>b152 Emotional functions<br>d710 Basic interpersonal interactions<br>d760 Family relationships	Activity level<br>Approaching mother<br>Approaching stranger<br>Cuddliness with mother<br>Cuddliness with stranger<br>Difference of response between mother and stranger: approach<br>Difference of response between mother and stranger: cuddliness<br>Difference of response between mother and stranger: looking<br>Difference of response between mother and stranger: positive vocalizations<br>Difference of response between mother and stranger: proximity<br>Difference of response between mother and stranger: quality of play<br>Difference of response between mother and stranger: smiling<br>Difference of response between mother and stranger: touches<br>Distress to limitations<br>Distress to novelty<br>Duration of orienting<br>Latency to approach stranger<br>Looking at mother<br>Looking at stranger<br>Negative affect<br>Positive affect<br>Positive vocalization to mother<br>Positive vocalization to stranger<br>Proximity to mother<br>Proximity to stranger<br>Quality of play with mother<br>Quality of play with stranger<br>Resistance to soothing<br>Separation distress<br>Smiling and laughter<br>Smiling at mother<br>Smiling at stranger<br>Touching mother<br>Touching stranger	Goldsmith et al. (1999)[51]<br>Plomin & Rowe (1979)[89]

© 2022 Austerberry C et al. *JAMA Network Open.*

eTable 5 (Continued)

Study name	Country	Age (months)	n twin pairs	Category (using codes from the ICF-CY)	Phenotypes (as labeled in included publications)	Publications
Department of Psychology, University of Surrey	United Kingdom	0–24, 12–24	118 (41 MZ, 77 DZ)	b147 Psychomotor functions<br>b152 Emotional functions<br>b164 Higher-level cognitive functions<br>d710 Basic interpersonal interactions	Activity level<br>Emotionality<br>Impulsivity<br>Sociability	Stevenson & Fielding (1985)[128]
Early Childhood Longitudinal Study-Birth Cohort	United States	9, 24	976 (238 MZ, 738 DZ)	b152 Emotional functions<br>b163 Basic cognitive functions<br>b167 Mental functions of language<br>d710 Basic interpersonal interactions<br>d720 Complex interpersonal interactions<br>d760 Family relationships	Attachment security<br>Avoids others/not sociable<br>Comfortable cuddly<br>Cooperative<br>Demanding/angry<br>Dependency<br>Enjoys company<br>Expressive vocabulary<br>Fussiness and demanding behavior<br>Independent<br>Mental ability<br>Moody/unusual<br>Positive and negative affect<br>Seeks attention<br>Upset by separation	Beaver et al. (2014)[9]<br>Jackson (2016)[58]<br>Roisman & Fraley (2006)[103]<br>Roisman & Fraley (2008)[104]<br>Tucker-Drob et al. (2011)[133]
Early Reciprocal Social Behavior Study	United States	18, 24	317 (126 MZ, 191 DZ)	d710 Basic interpersonal interactions<br>d720 Complex interpersonal interactions	Behavior problems<br>Competence<br>Functional communication<br>Reciprocal social behavior<br>Social avoidance<br>Social motivation<br>Social orienting	Hawks & Marrus (2018)[52]<br>Marrus et al. (2015)[69]<br>Marrus et al. (2018)[70]<br>Marrus et al. (2020)[71]
East Flanders Prospective Twin Survey	Belgium	0–1, 1–6, 6–12, 12–24	280 (190 MZ, 90 DZ)	b560 Growth maintenance functions	Weight gain	Touwslager et al. (2011a)[131]<br>Touwslager et al. (2011b)[132]

© 2022 Austerberry C et al. *JAMA Network Open.*

eTable 5 (Continued)

Study name	Country	Age (months)	n twin pairs	Category (using codes from the ICF-CY)	Phenotypes (as labeled in included publications)	Publications
Gemini Study	United Kingdom	0, 3, 6, 16	2757 (1174 MZ, 1583 DZ)	b134 Sleep functions<br>b147 Psychomotor functions<br>b560 Growth maintenance functions<br>d550 Eating	Activity level<br>BMI<br>Carbohydrate intake<br>Daytime nap duration<br>Emotional overeating<br>Energy intake<br>Enjoyment of food<br>Fat intake<br>First Crawl<br>First Sit<br>First Steps<br>Food fussiness<br>Food neophobia<br>Food responsiveness<br>Food weight<br>Night awakenings<br>Night-time sleep duration<br>Protein intake<br>Satiety responsiveness<br>Size (weight)<br>Slowness in eating<br>Tempo (weight)<br>Wake time<br>Weight<br>Weight change<br>Weight velocity	Fisher et al. (2012)[34]<br>Herle et al. (2018)[53]<br>Johnson et al. (2011)[60]<br>Llewellyn et al. (2010)[68]<br>Pimpin et al. (2013)[87]<br>Silventoinen et al. (2016)[121]<br>Smith et al. (2017a)[124]<br>Smith et al. (2017b)[125]
Guinea-Bissau Twin Study	Guinea-Bissau	0–2	108 (16 MZ, 92 DZ)	b560 Growth maintenance functions	BMI	Silventoinen et al. (2016)[121]
Hungarian Twin Registry	Hungary	2	389 (230 MZ, 159 DZ)	b560 Growth maintenance functions	BMI	Silventoinen et al. (2016)[121]

© 2022 Austerberry C et al. *JAMA Network Open.*

eTable 5 (Continued)

Study name	Country	Age (months)	n twin pairs	Category (using codes from the ICF-CY)	Phenotypes (as labeled in included publications)	Publications
Italian Twin Registry (previously the Mercurio project)	Italy	12–24, 18, 24	7432 (3270 MZ, 4162 DZ)	b134 Sleep functions<br>b560 Growth maintenance functions	BMI<br>Cosleeping<br>Diurnal sleep duration<br>Night awakenings<br>Nocturnal sleep duration<br>Weight gain	Brescianini et al. (2011)[14]<br>Silventoinen et al. (2016)[121]
Jackson Memorial Hospital/University of Miami Medical Center	United States	.5, 1, 3, 6, 9, 12	166 (67 MZ, 99 DZ)	b560 Growth maintenance functions	Length<br>Weight	Levine et al. (1987)[65]
Japanese Twin Cohort	Japan	1–2	2169 (1345 MZ, 824 DZ)	b560 Growth maintenance functions	BMI	Silventoinen et al. (2016)[121]

© 2022 Austerberry C et al. *JAMA Network Open.*

eTable 5 (Continued)

Study name	Country	Age (months)	n twin pairs	Category (using codes from the ICF-CY)	Phenotypes (as labeled in included publications)	Publications
Jumeaux et Plus ("Twins and more")	France	24	1950 (393 MZ, 1557 DZ)	b134 Sleep functions<br>b140 Attention functions<br>b147 Psychomotor functions<br>b152 Emotional functions<br>b279 Additional sensory functions<br>d550 Eating<br>d710 Basic interpersonal interactions<br>d720 Complex interpersonal interactions<br>d760 Family relationships	Activity/Impulsivity<br>Aggression/Defiance<br>Attention<br>Atypical Index<br>Competence<br>Compliance<br>Depression withdrawal<br>Dysregulation<br>Eating problems<br>Empathy<br>Externalizing<br>General anxiety<br>Imitation/Play<br>Inhibition to novelty<br>Internalizing<br>Maladaptive behaviors<br>Mastery Motivation<br>Negative affect<br>Peer aggression<br>Prosocial peer relations<br>Sensory Sensitivity<br>Separation distress<br>Sleep problems<br>Social relatedness	Saudino et al. (2008)[110]

© 2022 Austerberry C et al. *JAMA Network Open.*

eTable 5 (Continued)

Study name	Country	Age (months)	n twin pairs	Category (using codes from the ICF-CY)	Phenotypes (as labeled in included publications)	Publications
Louisville Twin Study	United States	0, 3, 6, 9, 12, 18, 24	615 (289 MZ, 326 DZ)	b140 Attention functions<br>b147 Psychomotor functions<br>b152 Emotional functions<br>b163 Basic cognitive functions<br>b279 Additional sensory functions<br>b310 Voice functions<br>b560 Growth maintenance functions<br>d710 Basic interpersonal interactions<br>d720 Complex interpersonal interactions<br>d760 Family relationships	Activity level<br>Activity-awake<br>Activity-sleep<br>Affect-extraversion<br>Approach/withdrawal<br>Attachment<br>Attention<br>Auditory-visual<br>Banging<br>Behavioral inhibition<br>Cognitive ability<br>Cooperative<br>Emotional tone<br>Endurance<br>Energy<br>Extraversion<br>Fearfulness<br>Fine motor<br>General temperament<br>Goal directedness<br>Gross motor<br>Height<br>Irritability<br>Listening<br>Looking<br>Manipulating<br>Motor development<br>Mouthing: pacifier<br>Mouthing: thumb<br>Mouthing: toys<br>Object orientation<br>Primary cognition<br>Reactivity<br>Resistance to soothing<br>Task orientation<br>Tension<br>Vocalize<br>Weight	Davis et al. (2015)[24]<br>Finkel et al. (2000)[33]<br>Matheny (1980)[72]<br>Matheny (1983)[73]<br>Matheny (1984)[74]<br>Matheny (1989)[75]<br>Matheny et al. (1976)[76]<br>Riese (1990a)[100]<br>Riese (1990b)[101]<br>Wilson (1972)[137]<br>Wilson (1974)[138]<br>Wilson (1978)[139]<br>Wilson (1983)[140]<br>Wilson (1984)[141]<br>Wilson & Harpring (1972)[142]<br>Wilson & Matheny (1976)[143]

© 2022 Austerberry C et al. *JAMA Network Open.*

eTable 5 (Continued)

Study name	Country	Age (months)	n twin pairs	Category (using codes from the ICF-CY)	Phenotypes (as labeled in included publications)	Publications
Manitoba Twin Study	Canada	7	60 twin pairs (39 MZ, 21 DZ)	b 147 Psychomotor functions<br>b 560 Growth maintenance functions	Activity level<br>Head circumference<br>Length<br>Motor development<br>Ponderal index<br>Weight	Saudino & Eaton (1991)[108]
Michigan State University Twin Registry	United States	24	10253 (3076 MZ, 7177 DZ)	b560 Growth maintenance functions	BMI	Silventoinen et al. (2016)[121]
Minnesota Twin Family Study	United States	0	1068 (682 MZ, 386 DZ)	b560 Growth maintenance functions	Weight	Hur et al. (2005)[55]
Mongolian Twin Registry	Mongolia	0–24	83 (36 MZ, 47 DZ)	b560 Growth maintenance functions	BMI	Silventoinen et al. (2016)[121]

© 2022 Austerberry C et al. *JAMA Network Open.*

eTable 5 (Continued)

Study name	Country	Age (months)	n twin pairs	Category (using codes from the ICF-CY)	Phenotypes (as labeled in included publications)	Publications
Moscow City twin sample	Russia	7–12	94 (49 MZ, 45 DZ)	b140 Attention functions<br>b144 Memory functions<br>b152 Emotional functions<br>b164 Higher-level cognitive functions<br>b279 Additional sensory functions<br>d710 Basic interpersonal interactions<br>d720 Complex interpersonal interactions<br>d760 Family relationships	Aggression towards father<br>Aggression towards mother<br>Autonomy<br>Control<br>Defensive reactions<br>Dependence on mother<br>EEG alpha frequency during darkness<br>EEG mu gravity frequency during visual attention<br>EEG spectral amplitude during darkness<br>EEG spectral amplitude during visual attention<br>Fear<br>High tension<br>Imitation of father<br>Imitation of mother<br>Love for father<br>Love for mother<br>Low tension<br>Moderate tension<br>Nonoriented discharges<br>Obedience to mother<br>Obedience to father<br>Object orientation<br>Orientation to humans<br>Passiveness<br>Reaction to father<br>Reaction to mother<br>Reaction to mother's punishment<br>Unpleasant sensations<br>Working memory	Orekhova et al. (2003)[82]<br>Pushina et al. (2005)[93]<br>Stroganova et al. (2000)[129]

© 2022 Austerberry C et al. *JAMA Network Open.*

eTable 5 (Continued)

Study name	Country	Age (months)	n twin pairs	Category (using codes from the ICF-CY)	Phenotypes (as labeled in included publications)	Publications
Multiple Births Foundation of Queen Charlotte's and Chelsea Hospital in London	United Kingdom	12–14	62 (30 MZ, 32 DZ)	b152 Emotional functions<br>d760 Family relationships	Attachment disorganization<br>Attachment security<br>Temperamental reactivity	Bokhorst et al. (2003)[11]
Netherlands Twin Register	The Netherlands	0, 1, 2, 3, 4, 6, 8, 9, 13, 14, 15.5, 24	16848 (5259 MZ, 11589 DZ)	b152 Emotional functions<br>b560 Growth maintenance functions<br>d760 Family relationships	Attachment disorganization<br>Attachment security<br>BMI<br>Dependency (to father)<br>Head circumference<br>Height<br>Height deceleration<br>Height jerk<br>Height snap<br>Height velocity<br>Infant-father attachment security<br>Length<br>Temperamental reactivity<br>Weight<br>Weight deceleration<br>Weight jerk<br>Weight snap<br>Weight velocity	Bakermans-Kranenburg et al. (2004)[8]<br>Bokhorst et al. (2003)[11]<br>Boomsma et al. (1992)[12]<br>Mook-Kanamori et al. (2012)[79]<br>Silventoinen et al. (2016)[121]<br>Smit et al. (2010)[122]<br>van Dommelen et al. (2004)[134]
Peri/Postnatal Epigenetic Twins Study	Australia	0–24	221 (91 MZ, 130 DZ)	b560 Growth maintenance functions	BMI	Silventoinen et al. (2016)[121]

© 2022 Austerberry C et al. *JAMA Network Open.*

eTable 5 (Continued)

Study name	Country	Age (months)	n twin pairs	Category (using codes from the ICF-CY)	Phenotypes (as labeled in included publications)	Publications
Pooled Sample of twins from Oregon, Washington, Colorado, Texas, and Wisconsin	United States	3–16	302 (121 MZ, 181 DZ)	b140 Attention functions<br>b147 Psychomotor functions<br>b152 Emotional functions	Activity level<br>Distress to limitations<br>Distress to novelty<br>Duration of orienting<br>Negative affect<br>Positive affect<br>Resistance to soothing<br>Smiling and laughter	Goldsmith et al. (1999)[51]
Project Metropolitan	Sweden	0	131 (28 MZ, 103 DZ)	b560 Growth maintenance functions	Height<br>Weight	Akerman & Fischbein (1992)[6]
Puerto Rican Infant Twin Study	United States	12, 0–32	865 (377 MZ, 488 DZ)	d710 Basic interpersonal interactions<br>d720 Complex interpersonal interactions	Difficult temperament<br>Difficultness<br>Inhibition<br>Resistance to control<br>Sociability<br>Unadaptability<br>Unsociability	Silberg et al. (2005)[114]

© 2022 Austerberry C et al. *JAMA Network Open.*

eTable 5 (Continued)

Study name	Country	Age (months)	n twin pairs	Category (using codes from the ICF-CY)	Phenotypes (as labeled in included publications)	Publications
Quebec Newborn Twin Study	Canada	0, 5, 6, 18, 19, 20, 18–24	1029 (419 MZ, 610 DZ)	b134 Sleep functions<br>b152 Emotional functions<br>b167 Mental functions of language<br>b560 Growth maintenance functions<br>d710 Basic interpersonal interactions<br>d720 Complex interpersonal interactions	BMI<br>Cortisol reactivity<br>Cortisol secretion in morning<br>Cortisol secretion on awakening<br>Daytime continuous sleep duration<br>Difficult temperament<br>Disregard for rules<br>Disruptive behavior<br>Emotional response to social stimuli<br>Gaze aversion<br>Height<br>Motor activity during social stimuli<br>Negative affect<br>Nighttime continuous sleep duration<br>Physical aggression<br>Ratio of day/night sleep duration<br>Self-contact during social stimuli<br>Sleep terrors<br>Social gaze<br>Testosterone<br>Vocabulary<br>Weight	Caramaschi et al. (2012)[16]<br>Dionne et al. (2003b)[28]<br>Dionne et al. (2011)[29]<br>Dubois et al. (2007)[30]<br>Dubois et al. (2012)[31]<br>Forget-Dubois et al. (2007)[38]<br>Lacourse et al. 2014[64]<br>Nguyen et al. (2008)[80]<br>Ouellet-Morin et al. (2008)[83]<br>Ouellet-Morin et al. (2009)[84]<br>Petitclerc et al. (2011)[86]<br>Schumann et al. (2017)[113]<br>Silventoinen et al. (2016)[121]<br>Soussignan et al. (2009)[126]<br>Touchette et al. (2013)[130]
Registry of twins recruited in Japan from associations for parents of multiples.	Japan	0	1045 (775 MZ, 270 DZ)	b560 Growth maintenance functions	Weight	Hur et al. (2005)[55]

© 2022 Austerberry C et al. *JAMA Network Open.*

eTable 5 (Continued)

Study name	Country	Age (months)	n twin pairs	Category (using codes from the ICF-CY)	Phenotypes (as labeled in included publications)	Publications
Registry of twins who applied for the secondary school attached to the Faculty of Education at the University of Tokyo between 1981 and 2003.	Japan	0	1045 (775 MZ, 270 DZ)	b560 Growth maintenance functions	Weight	Hur et al. (2005)[55]
Childcare Centers of Tel Aviv and Haifa	Israel	0–12	93 (64 DZ, 29 MZ)	b147 Psychomotor functions	Pulling up to a standing position<br>Sitting up<br>Turning over<br>Walking five steps	Peter et al. (1999)[85]
Seoul Twin Family Study	South Korea	0	686 (384 MZ, 302 DZ)	b560 Growth maintenance functions	Weight	Hur et al. (2005)[55]
South Korea Twin Registry	South Korea	0	433 (255 MZ,178 DZ)	b560 Growth maintenance functions	Weight	Hur (2005)[54]
Swedish Young Male Twins Study	Sweden	0, 12, 24	375 (231 MZ, 144 DZ)	b560 Growth maintenance functions	BMI<br>Height	Silventoinen et al. (2007)[116]<br>Silventoinen et al. (2008)[117]<br>Silventoinen et al. (2016)[121]

© 2022 Austerberry C et al. *JAMA Network Open.*

eTable 5 (Continued)

Study name	Country	Age (months)	n twin pairs	Category (using codes from the ICF-CY)	Phenotypes (as labeled in included publications)	Publications
Taipei City Teaching Hospitals Twin Study	Taiwan	1, 2, 4, 6, 9, 12	521 (428 MZ, 93 DZ)	b125 Dispositions and intra-personal functions<br>b140 Attention functions<br>b147 Psychomotor functions<br>b152 Emotional functions<br>b560 Growth maintenance functions<br>d710 Basic interpersonal interactions<br>d720 Complex interpersonal interactions	Activity level<br>Adaptability<br>Approach/withdrawal<br>Arm circumference<br>Attention/persistence<br>Chest circumference<br>Distractibility<br>Head circumference<br>Height<br>Intensity of reaction<br>Quality of mood<br>Rhythmicity<br>Threshold of responsiveness<br>Weight	Chen et al. (1990a)[17]<br>Chen et al. (1990b)[18]
Tokyo Twin Cohort Project	Japan	0, 4, 10, 19	1728 (669 MZ, 1059 DZ)	b134 Sleep functions<br>b560 Growth maintenance functions<br>d550 Eating<br>d710 Basic interpersonal interactions	Chest circumference<br>Head circumference<br>Head circumference growth<br>Height<br>Milk consumption<br>Mimic, point gazing, joint attention<br>Rhythmicity<br>Sociocognitive abilities<br>Time to fall asleep<br>Weight	Ando et al. (2006)[7]<br>Fujisawa et al. (2012)[40]

© 2022 Austerberry C et al. *JAMA Network Open.*

eTable 5 (Continued)

Study name	Country	Age (months)	n twin pairs	Category (using codes from the ICF-CY)	Phenotypes (as labeled in included publications)	Publications
Twin study in The Gambia	The Gambia	0, 5–18	84 (22 MZ, 62 DZ)	b147 Psychomotor functions<br>b560 Growth maintenance functions	Crawl<br>Length<br>Maintain head<br>Roll over<br>Sitting<br>Sitting without support<br>Stand holding on something<br>Take two steps<br>Walk holding on something<br>Weight	Goetghebuer et al. (2003)[49]
Twin Study of Child and Adolescent Development	Sweden	0	823 (376 MZ, 447 DZ)	b560 Growth maintenance functions	BMI<br>Height<br>Weight	Dubois et al. (2012)[31]
Twins born in two South Korean hospitals (1998–2003)	South Korea	0	603 (338 MZ, 265 DZ)	b560 Growth maintenance functions	Weight	Hur et al. (2005)[55]
Twins Early Development Study	United Kingdom	24	9065 (3082 MZ, 5983 DZ)	b140 Attention functions<br>b147 Psychomotor functions<br>b163 Basic cognitive functions<br>b167 Mental functions of language<br>b560 Growth maintenance functions<br>d710 Basic interpersonal interactions<br>d720 Complex interpersonal interactions	ADHD<br>Behavior problems<br>BMI<br>General cognitive ability<br>Grammar<br>Hyperactivity<br>Nonverbal cognitive ability<br>Prosocial behavior<br>Verbal ability	Dale et al. (2000)[23]<br>Dionne et al. (2003a)[27]<br>Galsworthy et al. (2000)[46]<br>Knafo & Plomin (2006)[61]<br>Koeppen-Schomerus et al. (2003)[62]<br>Kuntsi et al. (2005)[63]<br>Price et al. (2000)[91]<br>Price et al. (2005)[92]<br>Silventoinen et al. (2016)[121]<br>Spinath et al. (2003)[127]

© 2022 Austerberry C et al. *JAMA Network Open.*

eTable 5 (Continued)

Study name	Country	Age (months)	n twin pairs	Category (using codes from the ICF-CY)	Phenotypes (as labeled in included publications)	Publications
UNC Early Brain Development Study	United States	0, 0–3	180 (63 MZ, 117 DZ)	s110 Structure of brain[d]	Cerebellum<br>Corpus callosum<br>Cortical grey matter<br>Cortical surface area<br>Cortical thickness<br>Cortical unmyelinated white matter<br>Frontal grey matter<br>Frontal unmyelinated white matter<br>Intracranial volume<br>Lateral ventricles<br>Left hemisphere grey matter<br>Left hemisphere total<br>Left hemisphere unmyelinated white matter<br>Occipital grey matter<br>Occipital unmyelinated white matter<br>Parietal grey matter<br>Parietal unmyelinated white matter<br>Prefrontal grey matter<br>Prefrontal unmyelinated white matter<br>Right hemisphere grey matter<br>Right hemisphere total<br>Right hemisphere unmyelinated white matter<br>Subcortical grey matter<br>Total cerebrospinal fluid<br>Total early myelinated white matter<br>Total frontal<br>Total grey matter<br>Total occipital<br>Total parietal<br>Total prefrontal<br>Total unmyelinated white matter	Gilmore et al. (2010)[48]<br>Jha et al. (2018)[59]

[d] The prefix *s* is given to items in the component of Body Structures.

© 2022 Austerberry C et al. *JAMA Network Open.*

eTable 5 (Continued)

University of Sao Paulo Longitudinal Twin Study	Brazil	0–6	17 (10 MZ, 7 DZ)	b134 Sleep functions	Emergence of cortisol circadian rhythm	Custodio et al. (2007)[22]
Waisman Center Birth to 3 year project	United States	6, 12	531 (180 MZ, 351 DZ)	b152 Emotional functions	Positive affect<br>Smiling and laughter	Planalp et al. (2017)[88]
West Japan Twins and Higher Order Multiple Births Registry	Japan	0, 1–3, 3–5, 5–7, 7–9, 9–11, 11–13, 12, 24	767 (407 MZ, 360 DZ)	b560 Growth maintenance functions	BMI<br>Chest circumference<br>Chest circumference increase<br>Head circumference<br>Height	Silventoinen et al. (2011a)[118]<br>Silventoinen et al. (2011b)[119]<br>Silventoinen et al. (2012)[120]<br>Silventoinen et al. (2016)[121]
Western Australian Twin Registry	Australia	24	473 (160 MZ, 313 DZ)	b167 Mental functions of language	Combining words<br>Late language acquisition<br>Use of finiteness grammatical markers<br>Words Produced	Rice et al. (2014)[99]
Wisconsin Twin Panel	United States	12	735 (261 MZ, 474 DZ)	b152 Emotional functions	Anger<br>Distress to limitations	Gagne & Goldsmith (2011)[41]

© 2022 Austerberry C et al. *JAMA Network Open.*

eTable 6. Phenotypes Identified in Systematic Literature Search Coded Using Classification System

ICF-CY[a] component	ICF-CY domain	ICF-CY first level item	ICF-CY second level item (category)	ICF-CY third-level item (sub-category)	Phenotypes (as labeled in included publications)
Body functions	Mental functions	Global mental functions	b125 Dispositions and intra-personal functions	b1251 Responsivity	• Response duration<br>• Speed of response<br>• Threshold of responsiveness
Body functions	Mental functions	Global mental functions	b134 Sleep functions	b1340 Amount of sleep	• Cortisol secretion in morning<br>• Cortisol secretion on awakening<br>• Daytime continuous sleep duration<br>• Daytime nap duration<br>• Diurnal sleep duration<br>• Emergence of the cortisol circadian rhythm<br>• Night-time continuous sleep duration<br>• Night-time sleep duration<br>• Nocturnal sleep duration<br>• Rhythmicity<br>• Wake time
Body functions	Mental functions	Global mental functions	b134 Sleep functions	b1341 Onset of sleep	• Time to fall asleep
Body functions	Mental functions	Global mental functions	b134 Sleep functions	b1342 Maintenance of sleep	• Night awakenings<br>•
Body functions	Mental functions	Global mental functions	b134 Sleep functions	b1343 Quality of sleep	• Sleep problems<br>• Sleep terrors
Body functions	Mental functions	Global mental functions	b134 Sleep functions	b1348 Sleep functions, other specified	• Ratio of day/night sleep duration

[a] ICF-CY = International Classification of Functioning, Disability and Health, Children and Youth Version.[4] Definitions for each of the categories and subcategories can be found in the cited ICF-CY manual: https://apps.who.int/iris/handle/10665/43737.

© 2022 Austerberry C et al. *JAMA Network Open.*

eTable 6 (Continued)

ICF-CY component	ICF-CY domain	ICF-CY first level item	ICF-CY second level item (category)	ICF-CY third-level item (sub-category)	Phenotypes (as labeled in included publications)
Body functions	Mental functions	Specific mental functions	b140 Attention functions	b1400 Sustaining attention	• ADHD<br>• Attention<br>• Attention problems<br>• Attention/persistence<br>• Distractibility<br>• Duration of orienting<br>• Endurance<br>• Goal directedness<br>• Listening<br>• Looking<br>• Mastery motivation<br>• Object orientation<br>• Pursuit persistence<br>• Task orientation<br>• Visual attentiveness
Body functions	Mental functions	Specific mental functions	b140 Attention functions	b1408 Attention functions, other specified	• Mu frequency during visual attention<br>• Spectral amplitude during visual attention
Body functions	Mental functions	Specific mental functions	b144 Memory functions	b1440 Short-term memory	• Memory for location<br>• Working memory

© 2022 Austerberry C et al. *JAMA Network Open.*

eTable 6 (Continued)

ICF-CY component	ICF-CY domain	ICF-CY first level item	ICF-CY second level item (category)	ICF-CY third-level item (sub-category)	Phenotypes (as labeled in included publications)
Body functions	Mental functions	Specific mental functions	b147 Psychomotor functions	b1470 Psychomotor control	• Activity<br>• Activity level<br>• Activity level: Home<br>• Activity level: Lab<br>• Activity level: Play<br>• Activity-awake<br>• Activity-sleep<br>• Activity/impulsivity<br>• Energy<br>• Hyperactivity<br>• Vigorous activity vs. psychomotor passivity

© 2022 Austerberry C et al. *JAMA Network Open.*

eTable 6 (Continued)

ICF-CY component	ICF-CY domain	ICF-CY first level item	ICF-CY second level item (category)	ICF-CY third-level item (sub-category)	Phenotypes (as labeled in included publications)
Body functions	Mental functions	Specific mental functions	b147 Psychomotor functions	b1472 Organization of psychomotor functions	• Active manipulation<br>• Banging<br>• Crawl<br>• Fine motor<br>• First crawl<br>• First sit<br>• First steps<br>• Gross motor<br>• Maintain head<br>• Manipulating<br>• Motor development<br>• Mouthing: pacifier<br>• Mouthing: thumb<br>• Mouthing: toys<br>• Pulling up to standing position<br>• Roll over<br>• Sitting<br>• Sitting up<br>• Sitting without support<br>• Stand holding on something<br>• Take two steps<br>• Turning over<br>• Walk holding on something<br>• Walking five steps

© 2022 Austerberry C et al. *JAMA Network Open.*

eTable 6 (Continued)

ICF-CY component	ICF-CY domain	ICF-CY first level item	ICF-CY second level item (category)	ICF-CY third-level item (sub-category)	Phenotypes (as labeled in included publications)
Body functions	Mental functions	Specific mental functions	b152 Emotional functions	b1521 Regulation of emotion	• Affective problems<br>• Cortisol reactivity<br>• Distress to limitations<br>• Emotionality<br>• Moody/unusual<br>• Quality of mood<br>• Reactivity<br>• Resistance to soothing<br>• Temperamental reactivity
Body functions	Mental functions	Specific mental functions	b152 Emotional functions	b1522 Range of emotion	• Affect<br>• Anger<br>• Demanding/angry<br>• Depression withdrawal<br>• Emotional tone<br>• Fear<br>• Fearfulness<br>• Frustration<br>• Fussiness and demanding behavior<br>• General anxiety<br>• High tension<br>• Intensity of reaction<br>• Internalizing<br>• Irritability<br>• Low tension<br>• Moderate tension<br>• Negative affect<br>• Negative hedonic tone<br>• Nonoriented discharges<br>• Overall mood<br>• Positive Affect<br>• Positive and negative affect<br>• Positive hedonic tone<br>• Smiling and laughter<br>• Tension

© 2022 Austerberry C et al. *JAMA Network Open.*

eTable 6 (Continued)

ICF-CY component	ICF-CY domain	ICF-CY first level item	ICF-CY second level item (category)	ICF-CY third-level item (sub-category)	Phenotypes (as labeled in included publications)
Body functions	Mental functions	Specific mental functions	b163 Basic cognitive functions	N/A	• Cognitive ability<br>• General cognitive ability<br>• Mental ability<br>• Mental development<br>• Nonverbal<br>• Nonverbal cognitive ability<br>• Nonverbal cognitive development<br>• Primary cognition
Body functions	Mental functions	Specific mental functions	b164 Higher-level cognitive functions	b1641 Higher-level cognitive functions, other specified	• Categorization<br>• Control<br>• Impulsivity<br>• Inhibitory control<br>• Self-restraint

© 2022 Austerberry C et al. *JAMA Network Open.*

eTable 6 (Continued)

ICF-CY component	ICF-CY domain	ICF-CY first level item	ICF-CY second level item (category)	ICF-CY third-level item (sub-category)	Phenotypes (as labeled in included publications)
Body functions	Mental functions	Specific mental functions	b167 Mental functions of language	b1670 Reception of language	• Reception of language<br>• Verbal receptive<br>• Word comprehension
Body functions	Mental functions	Specific mental functions	b167 Mental functions of language	b1671 Expression of language	• Combining words<br>• Expressive language<br>• Expressive vocabulary<br>• Grammar<br>• Late language acquisition<br>• Use of finiteness grammatical markers<br>• Verbal ability<br>• Verbal expressive<br>• Vocabulary<br>• Words Produced
Body functions	Sensory functions and pain	Additional sensory functions	b279 Additional sensory functions, other specified and unspecified	N/A	• Auditory-visual<br>• Sensory Sensitivity<br>• Unpleasant sensations
Body functions	Voice and speech functions	N/A	b310 Voice functions	b3100 Production of voice	• Vocalize

© 2022 Austerberry C et al. *JAMA Network Open.*

eTable 6 (Continued)

ICF-CY component	ICF-CY domain	ICF-CY first level item	ICF-CY second level item (category)	ICF-CY third-level item (sub-category)	Phenotypes (as labeled in included publications)
Body functions	Functions of the digestive, metabolic and endocrine systems	Functions related to the metabolism and endocrine system	b560 Growth maintenance functions	N/A	• Age at transition to childhood<br>• Arm circumference<br>• BMI<br>• Chest circumference<br>• Chest circumference increase<br>• Head circumference<br>• Head circumference growth<br>• Head circumference growth curve parameters<br>• Height<br>• Height growth curve parameters<br>• Height deceleration<br>• Height jerk<br>• Height snap<br>• Height velocity<br>• Length<br>• Physical development<br>• Ponderal index<br>• Weight<br>• Weight growth<br>• Weight growth curve parameters<br>• Weight deceleration<br>• Weight gain<br>• Weight jerk<br>• Weight size<br>• Weight snap<br>• Weight tempo<br>• Weight velocity

© 2022 Austerberry C et al. *JAMA Network Open.*

eTable 6 (Continued)

ICF-CY component	ICF-CY domain	ICF-CY first level item	ICF-CY second level item (category)	ICF-CY third-level item (sub-category)	Phenotypes (as labeled in included publications)
Activities and participation	Self-care	N/A	d550 Eating	N/A	• Carbohydrate intake<br>• Eating problems<br>• Emotional overeating<br>• Energy intake<br>• Enjoyment of food<br>• Fat intake<br>• Food fussiness<br>• Food neophobia<br>• Food responsiveness<br>• Food weight<br>• Milk consumption<br>• Protein intake<br>• Satiety responsiveness<br>• Slowness in eating
Activities and participation	Interpersonal interactions and relationships	General interpersonal interactions	d710 Basic interpersonal interactions	d7100 Respect and warmth in relationships	• Affection for mother<br>• Callous unemotional behavior<br>• Callous unemotional traits<br>• Child affection<br>• Disregard for others<br>• Empathy<br>• Observed Disregard<br>• Prosocial behavior<br>• Prosocial Peer Relations

© 2022 Austerberry C et al. *JAMA Network Open.*

eTable 6 (Continued)

ICF-CY component	ICF-CY domain	ICF-CY first level item	ICF-CY second level item (category)	ICF-CY third-level item (sub-category)	Phenotypes (as labeled in included publications)
Activities and participation	Interpersonal interactions and relationships	General interpersonal interactions	d710 Basic interpersonal interactions	d7104 Social cues in relationships	• Affect-extraversion<br>• Approach/withdrawal<br>• Approaching mother<br>• Approaching stranger<br>• Avoids others/not sociable<br>• Behavioral inhibition<br>• Competence<br>• Degree of social acceptance of examiner<br>• Degree of social contact with mother<br>• Emotional response to social stimuli<br>• Enjoys company<br>• Enthusiasm for interaction with mother<br>• Extraversion<br>• Gaze aversion<br>• Imitation/Play<br>• Imitation of father<br>• Imitation of mother<br>• Inhibition<br>• Inhibition to novelty<br>• Interest in persons<br>• Interest in/responsiveness to people<br>• Latency to approach stranger<br>• Looking at mother<br>• Looking at stranger<br>• Mimic, point gazing, joint attention<br>• Orientation to humans<br>• Positive vocalization to mother<br>• Positive vocalization to stranger<br>• Proximity to stranger<br>• Quality of play with mother<br>• Quality of play with stranger<br>• Reaction to father<br>• Reaction to mother<br>• Reaction to mother's punishment<br>• Reciprocal social behavior<br>• Seeks attention<br>• Shyness<br>• Smiling at mother<br>• Smiling at stranger<br>• Sociability<br>• Social autistic-like traits<br>• Social gaze<br>• Social relatedness<br>• Sociocognitive abilities<br>• Unsociability<br>• Watch Mother

© 2022 Austerberry C et al. *JAMA Network Open.*

eTable 6 (Continued)

ICF-CY component	ICF-CY domain	ICF-CY first level item	ICF-CY second level item (category)	ICF-CY third-level item (sub-category)	Phenotypes (as labeled in included publications)
Activities and participation	Interpersonal interactions and relationships	General interpersonal interactions	d710 Basic interpersonal interactions	d7105 Physical contact in relationships	• Comfortable cuddly<br>• Cuddliness with mother<br>• Cuddliness with stranger<br>• Touching mother<br>• Touching stranger
Activities and participation	Interpersonal interactions and relationships	General interpersonal interactions	d710 Basic interpersonal interactions	d7106 Differentiation of familiar persons	• Difference of response between mother and stranger: approach<br>• Difference of response between mother and stranger: cuddliness<br>• Difference of response between mother and stranger: looking<br>• Difference of response between mother and stranger: positive vocalizations<br>• Difference of response between mother and stranger: proximity<br>• Difference of response between mother and stranger: quality of play<br>• Difference of response between mother and stranger: smiling<br>• Difference of response between mother and stranger: touches

© 2022 Austerberry C et al. *JAMA Network Open.*

eTable 6 (Continued)

ICF-CY component	ICF-CY domain	ICF-CY first level item	ICF-CY second level item (category)	ICF-CY third-level item (sub-category)	Phenotypes (as labeled in included publications)
Activities and participation	Interpersonal interactions and relationships	General interpersonal interactions	d720 Complex interpersonal interactions	d7202 Regulating behaviors within interactions	• Adaptability<br>• Aggression towards father<br>• Aggression towards mother<br>• Aggression/defiance<br>• Defensive reactions<br>• Difficult temperament<br>• Difficultness<br>• Disruptive behavior<br>• Externalizing<br>• Peer aggression<br>• Physical aggression<br>• Unadaptability
Activities and participation	Interpersonal interactions and relationships	General interpersonal interactions	d720 Complex interpersonal interactions	d7203 Interacting according to social rules	• Behavior problems<br>• Compliance<br>• Cooperative<br>• Disregard for rules<br>• Maladaptive behavior<br>• Obedience to mother<br>• Obedience to father<br>• ODD<br>• Resistance to control

© 2022 Austerberry C et al. *JAMA Network Open.*

eTable 6 (Continued)

ICF-CY component	ICF-CY domain	ICF-CY first level item	ICF-CY second level item (category)	ICF-CY third-level item (sub-category)	Phenotypes (as labeled in included publications)
Activities and participation	Interpersonal interactions and relationships	Particular interpersonal relationships	d760 Family relationships	d7601 Child-parent relationships	• Attachment<br>• Attachment disorganization<br>• Attachment security<br>• Dependence on mother<br>• Dependency<br>• Dependency to father<br>• Independent<br>• Infant-father attachment security<br>• Proximity to mother<br>• Separation distress<br>• Temperamental dependency<br>• Upset by separation
Body structures	Structures of the nervous system	N/A	s110 Structure of brain	s1100 Structure of cortical lobes	• Frontal grey matter<br>• Occipital grey matter<br>• Parietal grey matter<br>• Prefrontal grey matter<br>• Total Frontal<br>• Total Occipital<br>• Total Parietal<br>• Total Prefrontal
Body structures	Structures of the nervous system	N/A	s110 Structure of brain	s1104 Structure of cerebellum	• Cerebellum

© 2022 Austerberry C et al. *JAMA Network Open.*

eTable 6 (Continued)

ICF-CY component	ICF-CY domain	ICF-CY first level item	ICF-CY second level item (category)	ICF-CY third-level item (sub-category)	Phenotypes (as labeled in included publications)
Body structures	Structures of the nervous system	N/A	s110 Structure of brain	s1107 Structure of white matter	• Total unmyelinated white matter<br>• Total early myelinated white matter<br>• Cortical unmyelinated white matter<br>• Prefrontal unmyelinated white matter<br>• Frontal unmyelinated white matter<br>• Parietal unmyelinated white matter<br>• Occipital unmyelinated white matter<br>• Right hemisphere unmyelinated white matter<br>• Left hemisphere unmyelinated white matter<br>• Corpus Callosum
Body structures	Structures of the nervous system	N/A	s110 Structure of brain	s1108 Structure of brain, other specified	• Cortical grey matter<br>• Cortical surface area<br>• Cortical thickness<br>• Intracranial volume<br>• Lateral Ventricles<br>• Left hemisphere grey matter<br>• Left hemisphere total<br>• Right hemisphere grey matter<br>• Right hemisphere total<br>• Subcortical grey matter<br>• Total cerebrospinal fluid<br>• Total grey matter

© 2022 Austerberry C et al. *JAMA Network Open.*

eTable 6 (Continued)

ICF-CY component	ICF-CY domain	ICF-CY first level item	ICF-CY second level item (category)	ICF-CY third-level item (sub-category)	Phenotypes (as labeled in included publications)
Uncategorized	N/A	N/A	N/A	N/A	• Autistic-like traits<br>• Alpha frequency during darkness<br>• Atypical index<br>• Autonomy<br>• Cosleeping<br>• Distress to novelty<br>• Dysregulation<br>• General temperament<br>• Love for father<br>• Love for mother<br>• Motor activity during social stimuli<br>• Non-social autistic-like traits<br>• Passiveness<br>• Restrictive repetitive behavior<br>• Rhythmicity<br>• Self-contact/comfort during social stimuli<br>• Spectral amplitude during darkness<br>• Testosterone

© 2022 Austerberry C et al. *JAMA Network Open.*

eTable 7. Estimates and Cohorts in Phenotypic Categories by Rater

ICF-CY category	Rater	k_{cohort} [a]	$k_{estimate}$ [b]	$\%_{estimate}$
b134 Sleep	Parent	6	43	87.76
	Observer	0	0	0.00
	Other	2	6	12.24
b140 Attention	Parent	6	33	18.86
	Observer	4	66	37.71
	Other	1	76	43.43
b147 Psychomotor	Parent	10	45	29.80
	Observer	5	84	55.63
	Other	3	22	14.57
b152 Emotional	Parent	11	90	41.67
	Observer	6	114	52.78
	Other	2	12	5.56
b163 Basic cognitive	Parent	1	7	14.89
	Observer	4	40	85.11
	Other	0	0	0.00
b167 Language	Parent	5	38	39.58
	Observer	1	58	60.42
	Other	0	0	0.00
b560 Growth	Parent	0	0	0.00
	Observer	0	0	0.00
	Other	24	465	100.00
d710 Basic interpersonal	Parent	11	136	38.20
	Observer	7	202	56.74
	Other	2	18	5.06
d720 Complex interpersonal	Parent	8	59	80.82
	Observer	1	4	5.48
	Other	1	10	13.70
d760 Family relationships	Parent	3	17	58.62
	Observer	3	10	34.48
	Other	1	2	6.90
Total	Parent	22	468	28.24
	Observer	12	578	34.88
	Other	27	611	36.87

[a] k_{cohort} = number of independent twin cohorts.

[b] $k_{estimate}$ = number of estimates (twin correlations).

eTable 8. Multilevel Random Effects Models of Phenotypic Subcategories

ICF-CY sub-category	k_{cohort} [a]	$k_{estimate}$ [b]	n_{MZ} [c]	n_{DZ} [d]	r_{MZ} [e] pooled	r_{MZ} 95% CI [f]	r_{DZ} [g] pooled	r_{DZ} 95% CI	h^2 [h] pooled	h^2 95% CI	h^2 p [i]	c^2 [j] pooled	c^2 95% CI	c^2 p	e^2 [k] pooled	e^2 95% CI	e^2 p	I^2 [l] Level 1 [m]	I^2 Level 2 [n]	I^2 Level 3 [o]
Sustaining attention	10	101	3007	6135	.62	.49–.75	.37	.24–.50	.50	.14–.75	.007	.12	0–.41	.424	.38	.25–.51	<.001	6.53	42.17	51.30
Psychomotor control	11	83	3059	6287	.62	.48–.76	.29	.15–.43	.61	.26–.75	.001	.00	0–.27	1.000	.39	.26–.51	<.001	3.93	18.92	77.15
Organization of psychomotor	6	68	1022	1620	.79	.70–.88	.54	.45–.64	.49	.22–.76	<.001	.29	.08–.51	.006	.21	.12–.30	<.001	1.77	43.00	55.23
Regulation of emotion	10	48	980	1692	.53	.41–.65	.33	.20–.45	.41	.07–.66	.016	.12	0–.39	.379	.47	.35–.59	<.001	16.31	13.02	70.67
Range of emotion	11	168	1514	3271	.59	.49–70	.38	.28–.49	.42	.13–.69	.005	.17	0–.41	.153	.41	.30–.51	<.001	5.19	71.82	23.00
Expression of language	5	46	2244	2864	.86	.75–.98	.73	.61–.84	.27	0–.59	.099	.59	.34–.85	<.001	.14	.03–.25	<.001	0.21	58.26	41.53
Respect and warmth	7	39	3078	6131	.63	.45–.80	.53	.35–.70	.20	0–.69	.405	.42	.04–.69	.028	.37	.20–.53	.015	0.52	81.71	17.78
Social cues	15	285	1983	4030	.58	.46–.70	.38	.26–.50	.41	.06–.70	.021	.18	0–.46	.207	.42	.30–.54	<.001	2.20	20.69	77.10
Regulating behaviors	6	43	1056	2385	.72	.58–.87	.43	.28–.58	.58	.18–.87	.004	.14	0–.47	.393	.28	.14–.42	<.001	2.47	36.86	60.67
Social rules	9	30	3173	5070	.72	.60–.83	.56	.45–.68	.31	0–.63	.052	.41	.16–.66	.001	.28	.18–.39	<.001	1.93	0.00	98.07

[a] k_{cohort} = number of independent twin cohorts.
[b] $k_{estimate}$ = number of estimates (twin correlations).
[c] n_{MZ} = number of monozygotic (MZ) twin pairs.
[d] n_{DZ} = number of dizygotic (DZ) twin pairs.
[e] r_{MZ} = MZ twin correlation.
[f] 95% CI = 95% confidence interval.
[g] r_{DZ} = DZ twin correlation.
[h] h^2 = heritability.
[i] p = p-value
[j] c^2 = shared environment.
[k] e^2 = nonshared environment.
[l] I^2 = heterogeneity.
[m] Level 1 = sampling variance.
[n] Level 2 = within-cohort variance in outcome measurement.
[o] Level 3 = between-cohort variance.

© 2022 Austerberry C et al. *JAMA Network Open.*

eTable 9. Multilevel Random Effects Models of Parent and Observer Ratings of 3 Phenotypic Categories

ICF-CY category and rater	k_{cohort} [a]	$k_{estimate}$ [b]	n_{MZ} [c]	n_{DZ} [d]	r_{MZ} [e] pooled	r_{MZ} 95% CI [f]	r_{DZ} [g] pooled	r_{DZ} 95% CI	h^2 [h] pooled	h^2 95% CI	h^2 p [i]	c^2 [j] pooled	c^2 95% CI	c^2p	e^2 [k] pooled	$e^2_{95\% CI}$	e^2p	I^2 [l] Level 1 [m]	I^2 Level 2 [n]	I^2 Level 3 [o]
Psychomotor (obs [p])	5	84	503	588	0.55	.37–.74	0.38	.20–.56	0.35	0–.74	.182	0.21	0–.57	.313	0.45	.27–.63	<.001	7.84	45.02	47.14
Psychomotor (par [q])	10	45	2862	5732	0.69	.52–.85	0.30	.13–.47	0.67	.30–.82	.001	0.00	0–.29	1.000	0.33	.19–.47	<.001	0.88	2.92	96.20
Emotional (obs)	6	114	793	1261	0.40	.27–.53	0.26	.13–.39	0.28	0–.53	.129	0.12	0–.40	.391	0.60	.47–.73	<.001	16.49	43.14	40.38
Emotional (par)	11	90	1579	3492	0.67	.60–.74	0.42	.35–.49	0.49	.28–.71	<.001	0.17	.01–.35	.033	0.33	.26–.40	<.001	5.69	94.31	0.00
Basic interpersonal (obs)	7	202	839	945	0.37	.25–.49	0.27	.15–.39	0.20	0–.50	.254	0.17	0–.40	.221	0.63	.51–.75	<.001	18.76	32.15	49.09
Basic interpersonal (par)	11	136	3518	7229	0.70	.57–.82	0.42	.29–.55	0.55	.19–.83	.002	0.15	0–.44	.307	0.30	.18–.43	<.001	1.21	32.18	66.61

[a] k_{cohort} = number of independent twin cohorts.
[b] $k_{estimate}$ = number of estimates (twin correlations).
[c] n_{MZ} = number of monozygotic (MZ) twin pairs.
[d] n_{DZ} = number of dizygotic (DZ) twin pairs.
[e] r_{MZ} = MZ twin correlation.
[f] $_{95\% CI}$ = 95% confidence interval.
[g] r_{DZ} = DZ twin correlation.
[h] h^2 = heritability.
[i] p = p-value
[j] c^2 = shared environment.
[k] e^2 = nonshared environment.
[l] I^2 = heterogeneity.
[m] $_{Level\ 1}$ = sampling variance.
[n] $_{Level\ 2}$ = within-cohort variance in outcome measurement.
[o] $_{Level\ 3}$ = between-cohort variance.
[p] obs = observer rated.
[q] par = parent rated.

© 2022 Austerberry C et al. *JAMA Network Open.*

eTable 10. Tests for Publication Bias on Twin Correlations by Phenotype Category

	Egger's r_{MZ} [a]			Egger's r_{DZ} [b]		
ICF-CY category	$k_{estimate}$ [c]	z	p	$k_{estimate}$	z	p
b134 Sleep	24	-10.38	< .001	25	-3.55	< .001
b140 Attention	86	-8.51	< .001	89	-1.88	.061
b147 Psychomotor	75	-11.20	< .001	76	-4.17	< .001
b152 Emotional	105	-10.68	< .001	111	-4.76	< .001
b163 Basic cognitive	23	-6.26	< .001	24	-7.24	< .001
b167 Language	48	-22.50	< .001	48	-15.36	< .001
b560 Growth	216	-7.77	< .001	249	-4.78	< .001
d710 Basic interpersonal	174	-11.55	< .001	182	-10.05	< .001
d720 Complex interpersonal	34	-7.07	< .001	39	-3.40	< .001
d760 Family relationships	14	-4.84	< .001	15	-1.86	.062

[a] Egger's rMZ = Egger's test on monozygotic twin correlations.
[b] Egger's rMZ = Egger's test on dizygotic twin correlations.
[c] $k_{estimate}$ = number of estimates (twin correlations).

© 2022 Austerberry C et al. *JAMA Network Open.*

eTable 11. Tests for Publication Bias on Phenotype Categories by Variance Component in Phenotype Categories With ≥10 Estimates[a]

ICF-CY category	$k_{estimate}$[e]	Egger's h^2[b]		Egger's c^2[c]		Egger's e^2[d]	
		z	*p*	*z*	*p*	*z*	*p*
b134 Sleep	20	-1.80	.072	-3.62	< .001	-0.78	.438
b140 Attention	12	-1.12	.264	-0.84	.402	-2.10	.036
b147 Psychomotor	15	-5.26	< .001	-3.73	< .001	0.68	.494
b152 Emotional	20	-3.07	.002	-2.67	.008	-1.04	.297
b560 Growth	90	-0.87	.385	-5.64	< .001	-1.07	.285
d710 Basic interpersonal	41	-2.47	.013	-8.40	< .001	-2.21	.027
d720 Complex interpersonal	15	-3.07	.002	-5.52	< .001	-1.65	.099

[a] Because many studies only reported twin correlations, and not h^2, c^2 and e^2 estimates, the number of estimates included in the Egger's tests of h^2, c^2 and e^2 was smaller for all phenotypic categories than the number of estimates included in Egger's tests of twin correlations. There were too few estimates (< 10 estimates) to meet our criteria to create funnel plots or run Egger's tests on estimates of h^2, c^2 and e^2 for 'basic cognitive functions', 'mental functions of language' and 'family relationships'.
[b] Egger's h^2 = Egger's test on heritability estimates.
[c] Egger's c^2 = Egger's test on shared environment estimates.
[d] Egger's e^2 = Egger's test on nonshared environment estimates.
[e] $k_{estimate}$ = number of estimates (twin correlations).

© 2022 Austerberry C et al. *JAMA Network Open.*

eFigure 1. Prisma Flow Diagram

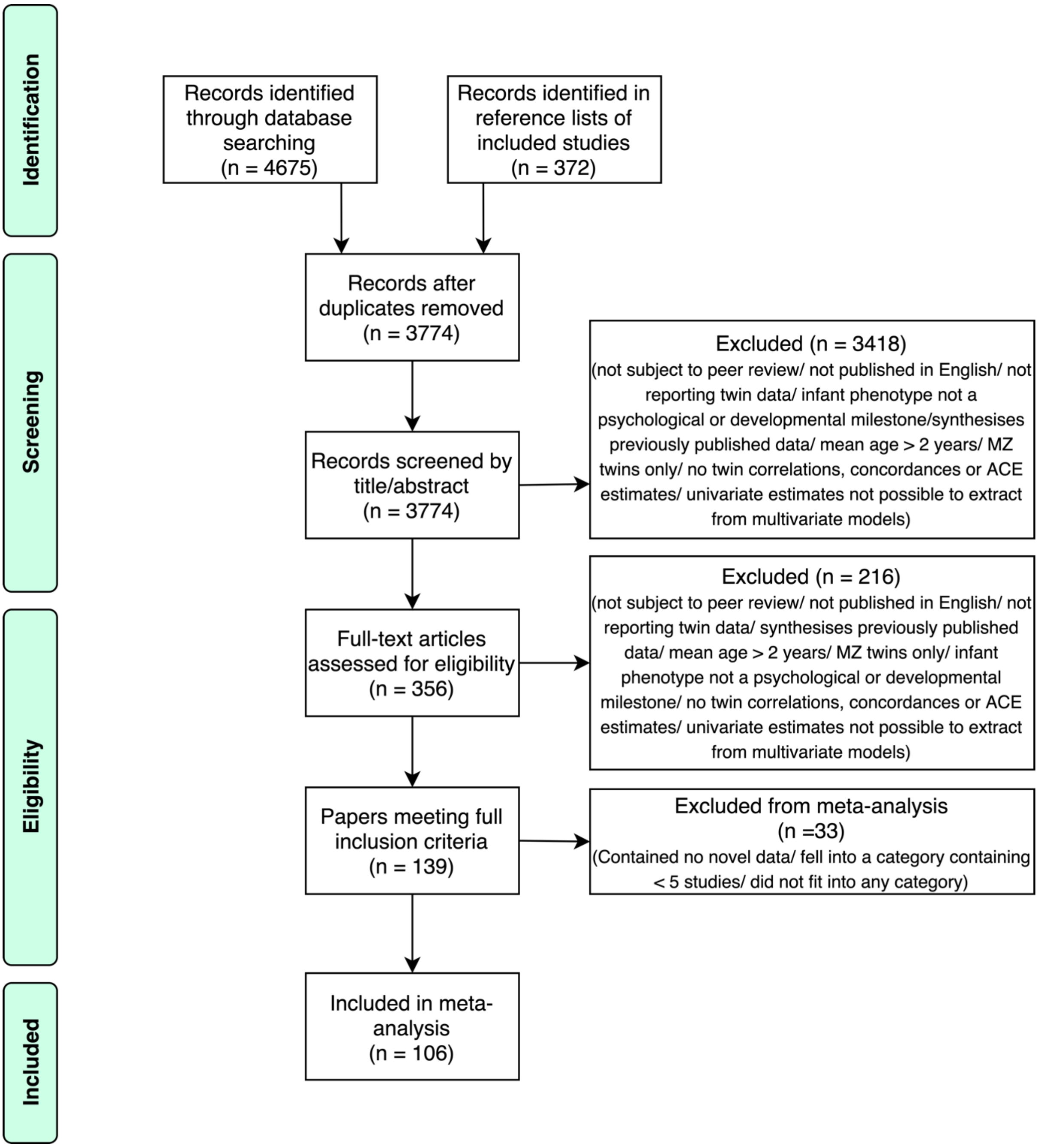

© 2022 Austerberry C et al. *JAMA Network Open.*

eFigure 2. Bar Chart of Number of Twin[a] Pairs by Country

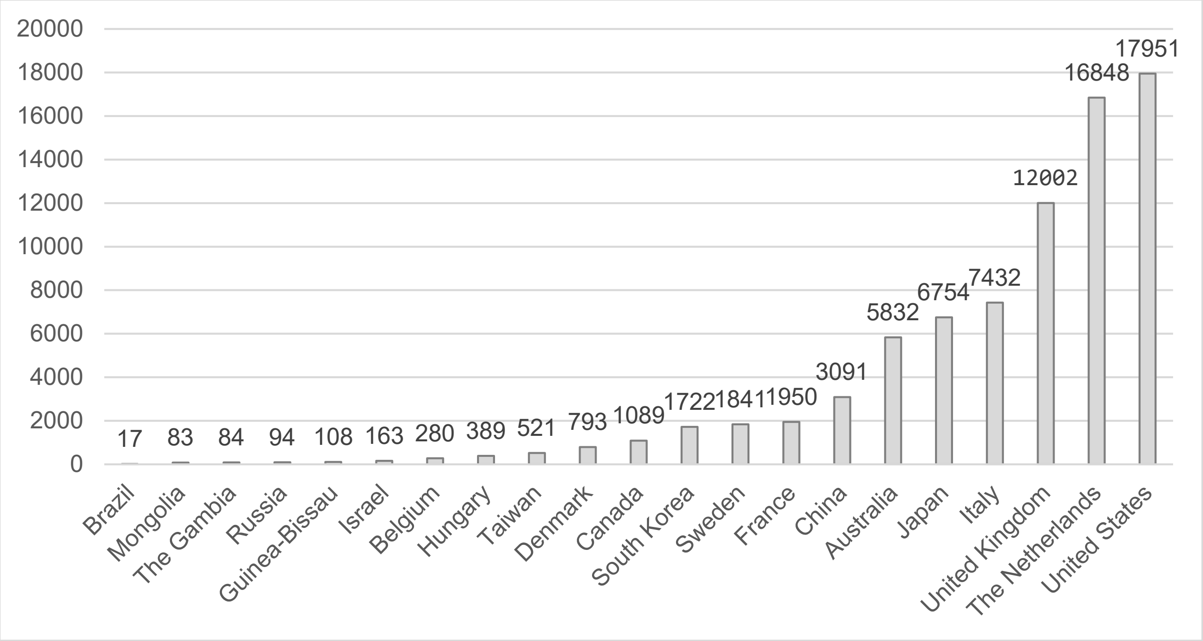

[a] Total number of twin pairs = 79,044

© 2022 Austerberry C et al. *JAMA Network Open.*

eFigure 3. Bar Chart of Number of Twin[a] Pairs by Continent

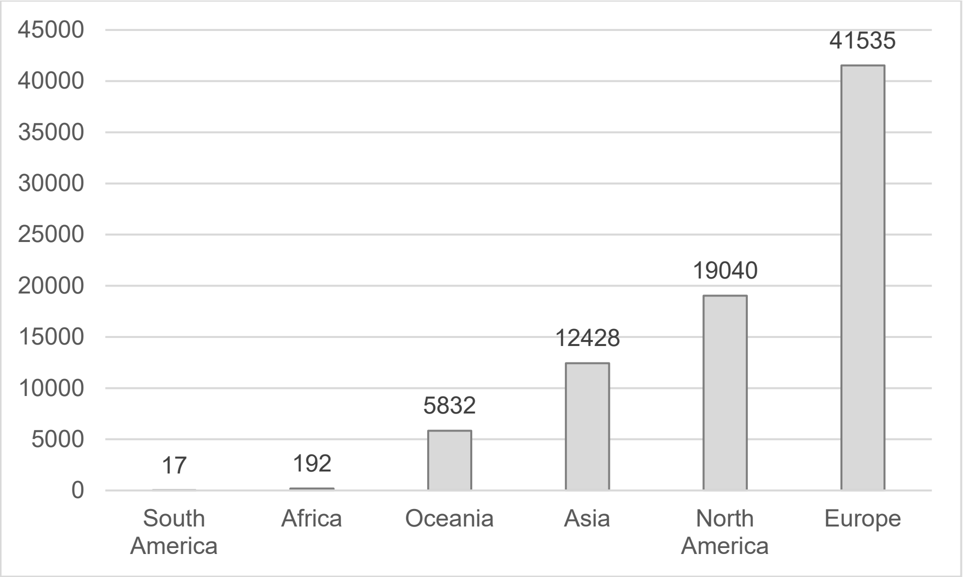

[a] Total number of twin pairs = 79,044

© 2022 Austerberry C et al. *JAMA Network Open.*

eFigure 4. Sleep Functions Forest Plot

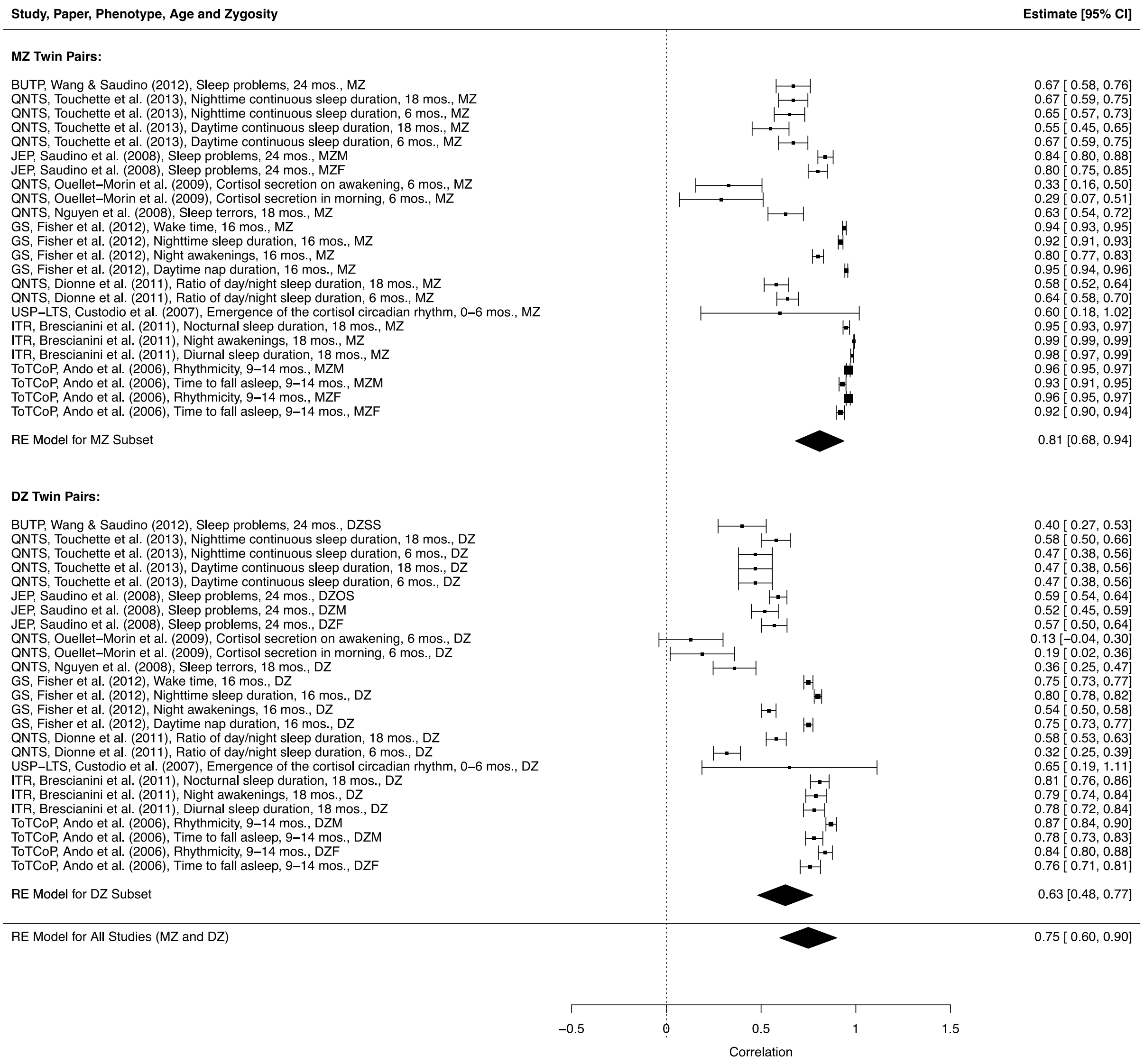

© 2022 Austerberry C et al. *JAMA Network Open.*

eFigure 5. Attention Functions Forest Plot

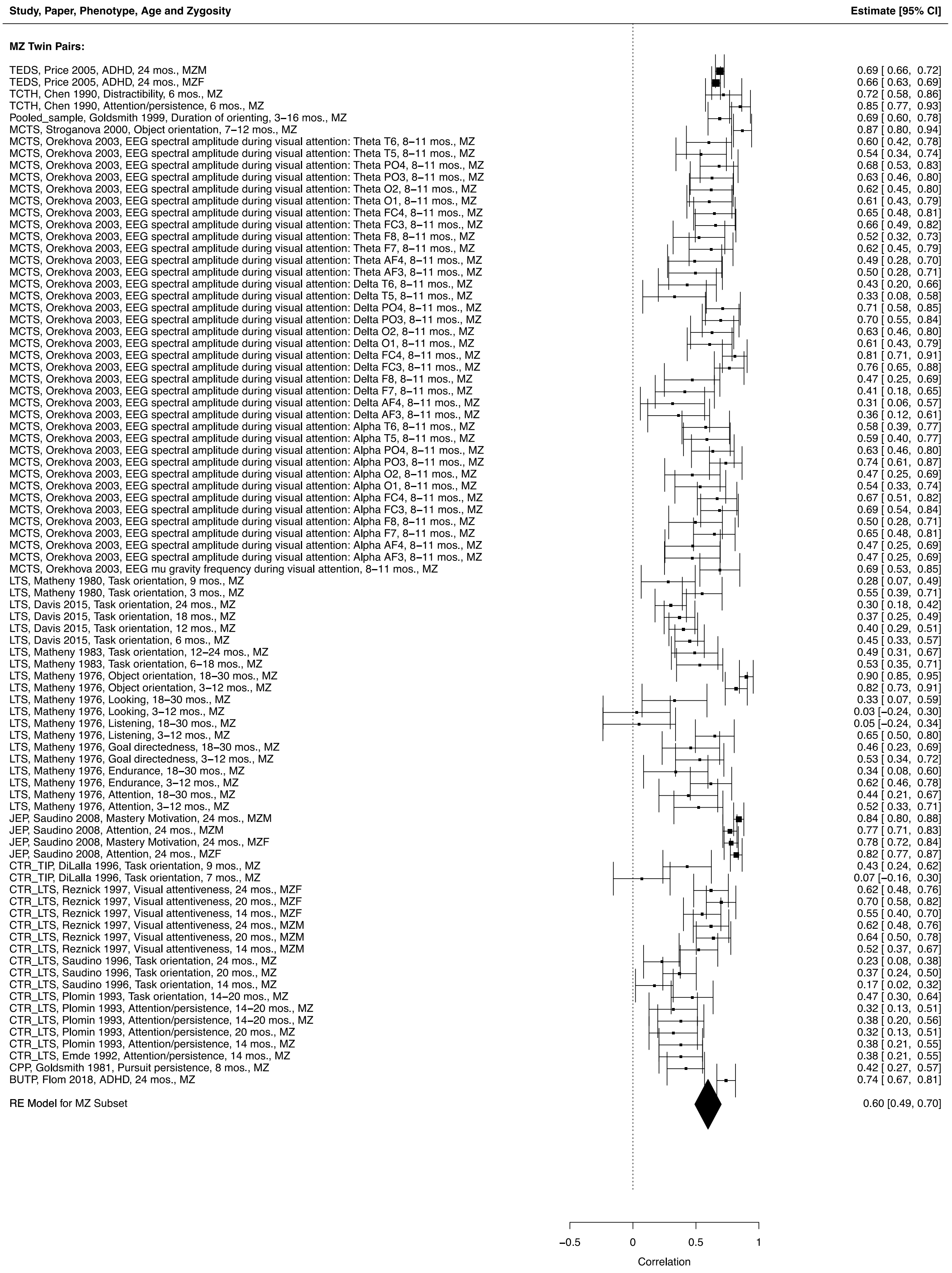

© 2022 Austerberry C et al. *JAMA Network Open.*

eFigure 5 (Continued)

DZ Twin Pairs:

Study	Correlation [95% CI]
TEDS, Price 2005, ADHD, 24 mos., DZOS	0.21 [0.17, 0.25]
TEDS, Price 2005, ADHD, 24 mos., DZM	0.14 [0.08, 0.20]
TEDS, Price 2005, ADHD, 24 mos., DZF	0.15 [0.09, 0.21]
TCTH, Chen 1990, Distractibility, 6 mos., DZ	0.61 [0.31, 0.91]
TCTH, Chen 1990, Attention/persistence, 6 mos., DZ	0.62 [0.33, 0.91]
Pooled_sample, Goldsmith 1999, Duration of orienting, 3–16 mos., DZ	0.45 [0.33, 0.57]
MCTS, Stroganova 2000, Object orientation, 7–12 mos., DZ	0.66 [0.48, 0.84]
MCTS, Orekhova 2003, EEG spectral amplitude during visual attention: Theta T6, 8–11 mos., DZ	0.54 [0.33, 0.75]
MCTS, Orekhova 2003, EEG spectral amplitude during visual attention: Theta T5, 8–11 mos., DZ	0.22 [−0.07, 0.51]
MCTS, Orekhova 2003, EEG spectral amplitude during visual attention: Theta PO4, 8–11 mos., DZ	0.53 [0.31, 0.74]
MCTS, Orekhova 2003, EEG spectral amplitude during visual attention: Theta PO3, 8–11 mos., DZ	0.47 [0.23, 0.70]
MCTS, Orekhova 2003, EEG spectral amplitude during visual attention: Theta O2, 8–11 mos., DZ	0.41 [0.15, 0.66]
MCTS, Orekhova 2003, EEG spectral amplitude during visual attention: Theta O1, 8–11 mos., DZ	0.35 [0.08, 0.62]
MCTS, Orekhova 2003, EEG spectral amplitude during visual attention: Theta FC4, 8–11 mos., DZ	0.62 [0.44, 0.81]
MCTS, Orekhova 2003, EEG spectral amplitude during visual attention: Theta FC3, 8–11 mos., DZ	0.50 [0.27, 0.73]
MCTS, Orekhova 2003, EEG spectral amplitude during visual attention: Theta F8, 8–11 mos., DZ	0.50 [0.28, 0.73]
MCTS, Orekhova 2003, EEG spectral amplitude during visual attention: Theta F7, 8–11 mos., DZ	0.44 [0.19, 0.68]
MCTS, Orekhova 2003, EEG spectral amplitude during visual attention: Theta AF4, 8–11 mos., DZ	0.63 [0.44, 0.81]
MCTS, Orekhova 2003, EEG spectral amplitude during visual attention: Theta AF3, 8–11 mos., DZ	0.62 [0.43, 0.81]
MCTS, Orekhova 2003, EEG spectral amplitude during visual attention: Delta T6, 8–11 mos., DZ	0.30 [0.03, 0.58]
MCTS, Orekhova 2003, EEG spectral amplitude during visual attention: Delta T5, 8–11 mos., DZ	0.26 [−0.02, 0.54]
MCTS, Orekhova 2003, EEG spectral amplitude during visual attention: Delta PO4, 8–11 mos., DZ	0.36 [0.10, 0.62]
MCTS, Orekhova 2003, EEG spectral amplitude during visual attention: Delta PO3, 8–11 mos., DZ	0.20 [−0.09, 0.49]
MCTS, Orekhova 2003, EEG spectral amplitude during visual attention: Delta O2, 8–11 mos., DZ	0.41 [0.16, 0.66]
MCTS, Orekhova 2003, EEG spectral amplitude during visual attention: Delta O1, 8–11 mos., DZ	0.28 [0.00, 0.56]
MCTS, Orekhova 2003, EEG spectral amplitude during visual attention: Delta FC4, 8–11 mos., DZ	0.38 [0.12, 0.63]
MCTS, Orekhova 2003, EEG spectral amplitude during visual attention: Delta FC3, 8–11 mos., DZ	0.34 [0.07, 0.61]
MCTS, Orekhova 2003, EEG spectral amplitude during visual attention: Delta F8, 8–11 mos., DZ	0.42 [0.17, 0.67]
MCTS, Orekhova 2003, EEG spectral amplitude during visual attention: Delta F7, 8–11 mos., DZ	0.36 [0.10, 0.62]
MCTS, Orekhova 2003, EEG spectral amplitude during visual attention: Delta AF4, 8–11 mos., DZ	0.61 [0.43, 0.80]
MCTS, Orekhova 2003, EEG spectral amplitude during visual attention: Delta AF3, 8–11 mos., DZ	0.44 [0.19, 0.68]
MCTS, Orekhova 2003, EEG spectral amplitude during visual attention: Alpha T6, 8–11 mos., DZ	0.42 [0.18, 0.67]
MCTS, Orekhova 2003, EEG spectral amplitude during visual attention: Alpha T5, 8–11 mos., DZ	0.11 [−0.19, 0.41]
MCTS, Orekhova 2003, EEG spectral amplitude during visual attention: Alpha PO4, 8–11 mos., DZ	0.27 [−0.01, 0.55]
MCTS, Orekhova 2003, EEG spectral amplitude during visual attention: Alpha PO3, 8–11 mos., DZ	0.38 [0.12, 0.64]
MCTS, Orekhova 2003, EEG spectral amplitude during visual attention: Alpha O2, 8–11 mos., DZ	0.36 [0.09, 0.62]
MCTS, Orekhova 2003, EEG spectral amplitude during visual attention: Alpha O1, 8–11 mos., DZ	0.31 [0.04, 0.59]
MCTS, Orekhova 2003, EEG spectral amplitude during visual attention: Alpha FC4, 8–11 mos., DZ	0.52 [0.30, 0.74]
MCTS, Orekhova 2003, EEG spectral amplitude during visual attention: Alpha FC3, 8–11 mos., DZ	0.45 [0.21, 0.69]
MCTS, Orekhova 2003, EEG spectral amplitude during visual attention: Alpha F8, 8–11 mos., DZ	0.38 [0.12, 0.64]
MCTS, Orekhova 2003, EEG spectral amplitude during visual attention: Alpha F7, 8–11 mos., DZ	0.28 [0.00, 0.56]
MCTS, Orekhova 2003, EEG spectral amplitude during visual attention: Alpha AF4, 8–11 mos., DZ	0.49 [0.26, 0.72]
MCTS, Orekhova 2003, EEG spectral amplitude during visual attention: Alpha AF3, 8–11 mos., DZ	0.47 [0.23, 0.70]
MCTS, Orekhova 2003, EEG mu gravity frequency during visual attention, 8–11 mos., DZ	0.50 [0.26, 0.74]
LTS, Matheny 1980, Task orientation, 9 mos., DZSS	0.00 [−0.34, 0.34]
LTS, Matheny 1980, Task orientation, 3 mos., DZSS	0.19 [−0.08, 0.46]
LTS, Davis 2015, Task orientation, 24 mos., DZ	0.38 [0.27, 0.49]
LTS, Davis 2015, Task orientation, 18 mos., DZ	0.30 [0.18, 0.42]
LTS, Davis 2015, Task orientation, 12 mos., DZ	0.33 [0.22, 0.44]
LTS, Davis 2015, Task orientation, 6 mos., DZ	0.36 [0.25, 0.47]
LTS, Matheny 1983, Task orientation, 12–24 mos., DZSS	0.21 [−0.09, 0.51]
LTS, Matheny 1983, Task orientation, 6–18 mos., DZSS	0.18 [−0.15, 0.51]
LTS, Matheny 1976, Object orientation, 18–30 mos., DZSS	0.67 [0.46, 0.88]
LTS, Matheny 1976, Object orientation, 3–12 mos., DZSS	0.66 [0.44, 0.88]
LTS, Matheny 1976, Looking, 18–30 mos., DZSS	0.33 [−0.01, 0.67]
LTS, Matheny 1976, Looking, 3–12 mos., DZSS	0.41 [0.09, 0.73]
LTS, Matheny 1976, Listening, 18–30 mos., DZSS	0.36 [0.03, 0.69]
LTS, Matheny 1976, Listening, 3–12 mos., DZSS	0.53 [0.25, 0.81]
LTS, Matheny 1976, Goal directedness, 18–30 mos., DZSS	−0.11 [−0.49, 0.27]
LTS, Matheny 1976, Goal directedness, 3–12 mos., DZSS	0.20 [−0.17, 0.57]
LTS, Matheny 1976, Endurance, 18–30 mos., DZSS	0.33 [−0.01, 0.67]
LTS, Matheny 1976, Endurance, 3–12 mos., DZSS	0.01 [−0.37, 0.39]
LTS, Matheny 1976, Attention, 18–30 mos., DZSS	0.34 [0.00, 0.68]
LTS, Matheny 1976, Attention, 3–12 mos., DZSS	0.23 [−0.13, 0.59]
JEP, Saudino 2008, Mastery Motivation, 24 mos., DZOS	0.59 [0.54, 0.64]
JEP, Saudino 2008, Attention, 24 mos., DZOS	0.52 [0.47, 0.57]
JEP, Saudino 2008, Mastery Motivation, 24 mos., DZM	0.59 [0.53, 0.65]
JEP, Saudino 2008, Attention, 24 mos., DZM	0.60 [0.54, 0.66]
JEP, Saudino 2008, Mastery Motivation, 24 mos., DZF	0.54 [0.47, 0.61]
JEP, Saudino 2008, Attention, 24 mos., DZF	0.52 [0.45, 0.59]
CTR_TIP, DiLalla 1996, Task orientation, 9 mos., DZ	0.32 [0.13, 0.51]
CTR_TIP, DiLalla 1996, Task orientation, 7 mos., DZ	0.26 [0.07, 0.45]
CTR_LTS, Reznick 1997, Visual attentiveness, 24 mos., DZF	0.08 [−0.17, 0.33]
CTR_LTS, Reznick 1997, Visual attentiveness, 20 mos., DZF	0.30 [0.07, 0.53]
CTR_LTS, Reznick 1997, Visual attentiveness, 14 mos., DZF	0.14 [−0.09, 0.37]
CTR_LTS, Reznick 1997, Visual attentiveness, 24 mos., DZM	0.47 [0.29, 0.65]
CTR_LTS, Reznick 1997, Visual attentiveness, 20 mos., DZM	0.35 [0.14, 0.56]
CTR_LTS, Reznick 1997, Visual attentiveness, 14 mos., DZM	0.48 [0.31, 0.65]
CTR_LTS, Saudino 1996, Task orientation, 24 mos., DZ	0.15 [−0.01, 0.31]
CTR_LTS, Saudino 1996, Task orientation, 20 mos., DZ	0.02 [−0.15, 0.19]
CTR_LTS, Saudino 1996, Task orientation, 14 mos., DZ	0.13 [−0.04, 0.30]
CTR_LTS, Plomin 1993, Task orientation, 14–20 mos., DZSS	0.00 [−0.21, 0.21]
CTR_LTS, Plomin 1993, Attention/persistence, 14–20 mos., DZSS	−0.25 [−0.45, −0.05]
CTR_LTS, Plomin 1993, Attention/persistence, 14–20 mos., DZSS	−0.04 [−0.25, 0.17]
CTR_LTS, Plomin 1993, Attention/persistence, 20 mos., DZSS	−0.25 [−0.45, −0.05]
CTR_LTS, Plomin 1993, Attention/persistence, 14 mos., DZSS	−0.04 [−0.24, 0.16]
CTR_LTS, Emde 1992, Attention/persistence, 14 mos., DZSS	−0.04 [−0.24, 0.16]
CPP, Goldsmith 1981, Pursuit persistence, 8 mos., DZ	0.35 [0.23, 0.47]
BUTP, Flom 2018, ADHD, 24 mos., DZSS	0.39 [0.26, 0.52]
RE Model for DZ Subset	0.36 [0.24, 0.47]
RE Model for All Studies (MZ and DZ)	0.51 [0.40, 0.62]

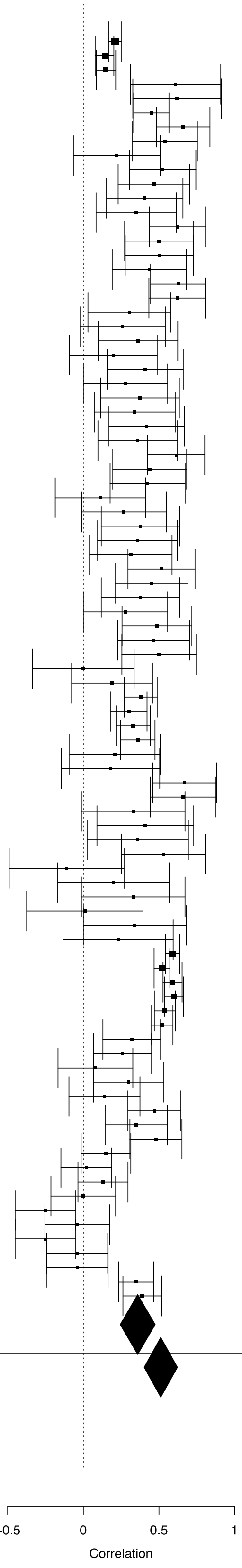

© 2022 Austerberry C et al. *JAMA Network Open.*

eFigure 6. Psychomotor Functions Forest Plot

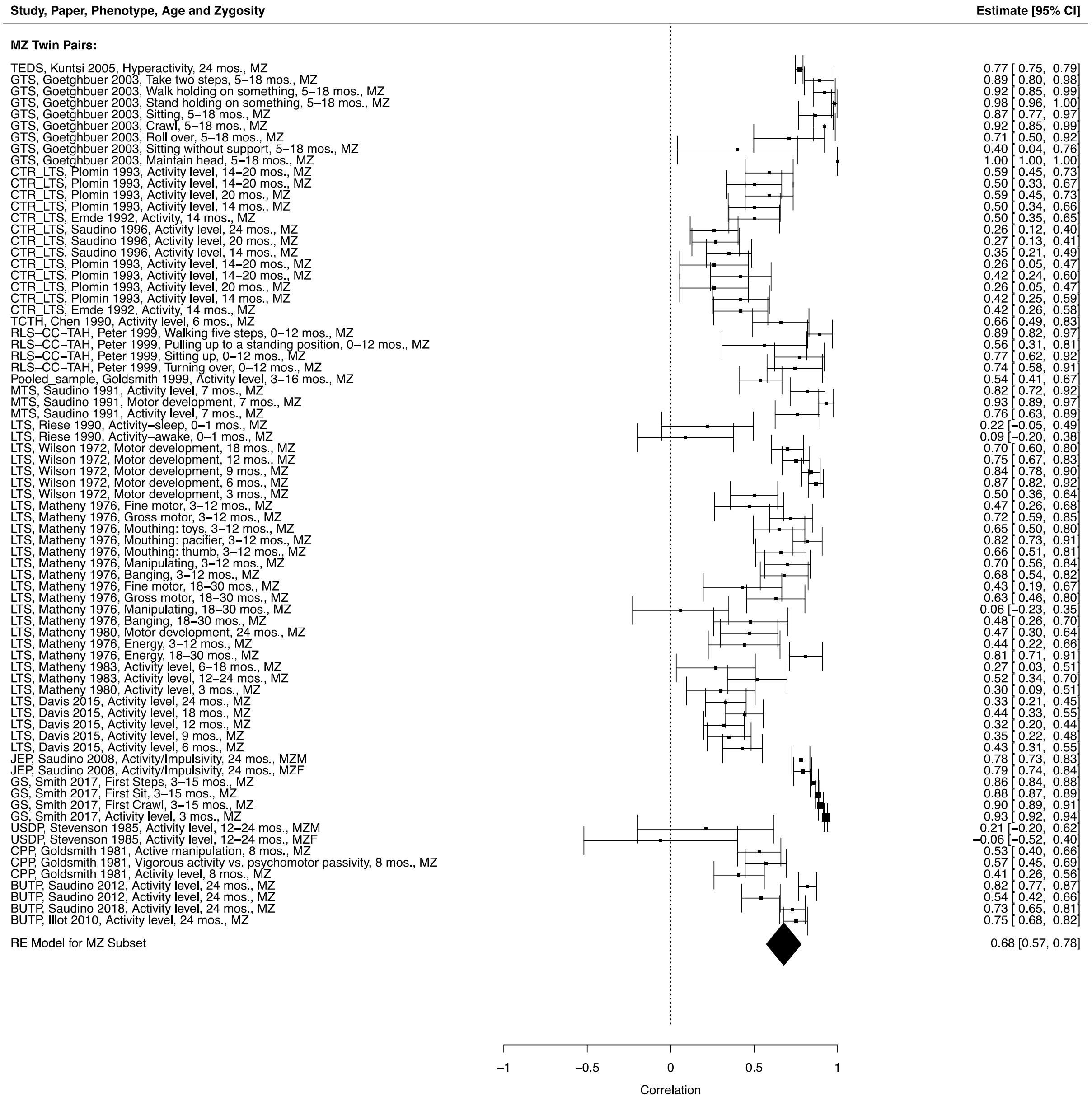

© 2022 Austerberry C et al. *JAMA Network Open.*

eFigure 6 (Continued)

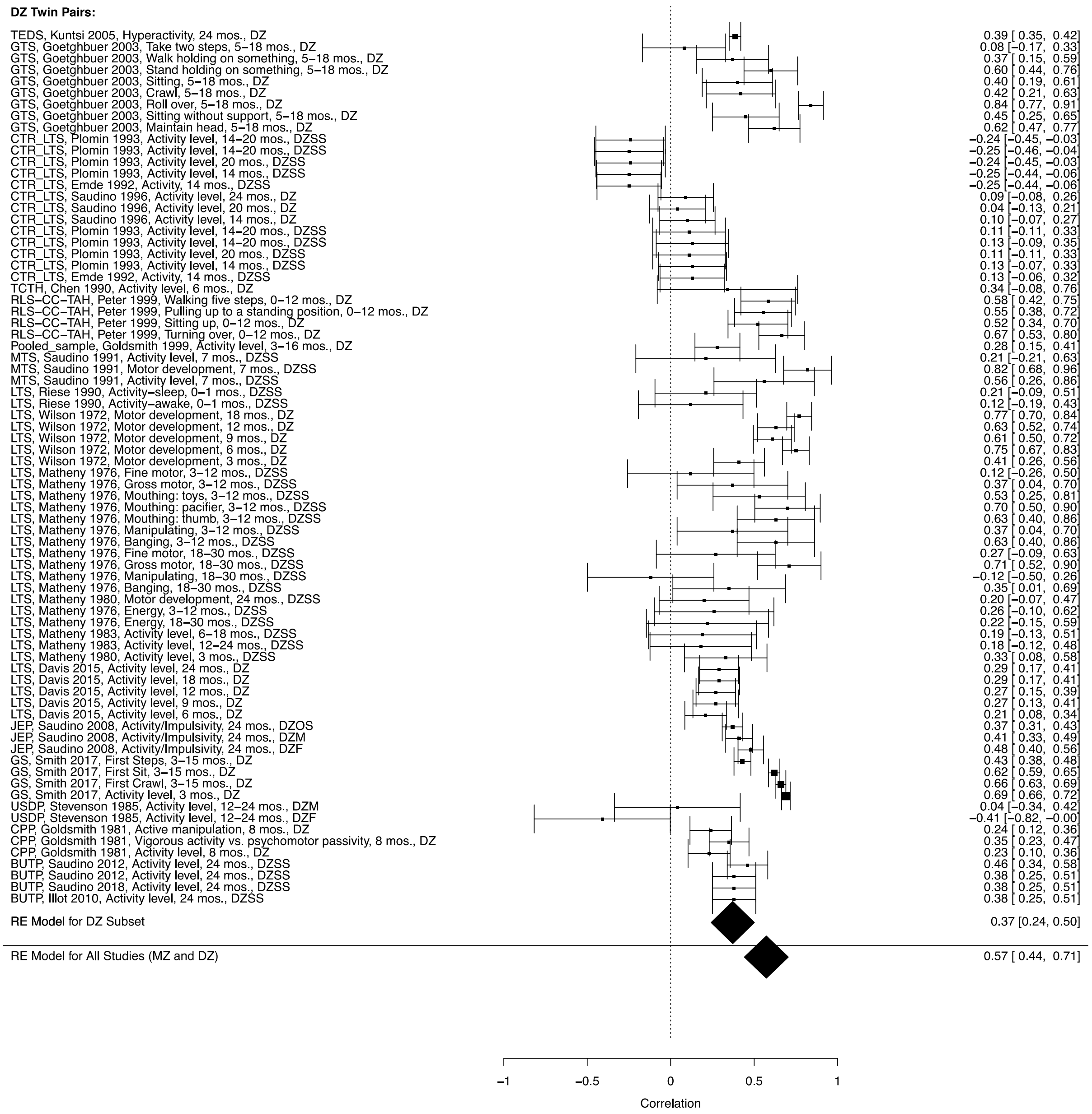

© 2022 Austerberry C et al. *JAMA Network Open.*

eFigure 7. Emotional Functions Forest Plot

Study, Paper, Phenotype, Age and Zygosity	Estimate [95% CI]
**MZ Twin Pairs:**	
WTP, Gagne 2011, Anger, 12 mos., MZ	0.38 [0.23, 0.53]
WTP, Gagne 2011, Distress to limitations, 12 mos., MZ	0.62 [0.51, 0.73]
WC_BTTYP, Planalp 2017, Positive affect, 12 mos., MZ	0.39 [0.29, 0.49]
WC_BTTYP, Planalp 2017, Positive affect, 6 mos., MZ	0.33 [0.20, 0.46]
WC_BTTYP, Planalp 2017, Smiling and laughter, 6 mos., MZ	0.85 [0.81, 0.89]
WC_BTTYP, Planalp 2017, Smiling and laughter, 12 mos., MZ	0.84 [0.80, 0.88]
USDP, Stevenson 1985, Emotionality, 0–24 mos., MZM	−0.05 [−0.48, 0.38]
USDP, Stevenson 1985, Emotionality, 0–24 mos., MZF	0.61 [0.32, 0.90]
TCTH, Chen 1990, Quality of mood, 6 mos., MZ	0.81 [0.71, 0.91]
TCTH, Chen 1990, Intensity of reaction, 6 mos., MZ	0.71 [0.56, 0.86]
QNTS, Schumann 2017, Negative affect, 18 mos., MZM	0.54 [0.40, 0.68]
QNTS, Schumann 2017, Negative affect, 5 mos., MZM	0.28 [0.11, 0.45]
QNTS, Schumann 2017, Negative affect, 5 mos., MZF	0.42 [0.27, 0.57]
QNTS, Schumann 2017, Negative affect, 18 mos., MZF	0.31 [0.15, 0.47]
QNTS, Ouellet−Morin 2008, Cortisol reactivity, 19 mos., MZ	0.44 [0.24, 0.64]
Pooled_sample, Goldsmith 1999, Positive affect, 3–16 mos., MZ	0.74 [0.66, 0.82]
Pooled_sample, Goldsmith 1999, Negative affect, 3–16 mos., MZ	0.64 [0.53, 0.75]
Pooled_sample, Goldsmith 1999, Resistance to soothing, 3–16 mos., MZ	0.53 [0.40, 0.66]
Pooled_sample, Goldsmith 1999, Smiling and laughter, 3–16 mos., MZ	0.72 [0.63, 0.81]
Pooled_sample, Goldsmith 1999, Distress to limitations, 3–16 mos., MZ	0.66 [0.56, 0.76]
NTR_MBF−QCCH, Bokhorst 2003, Temperamental reactivity, 12–14 mos., MZ	0.77 [0.66, 0.88]
MCTS, Stroganova 2000, Nonoriented discharges, 7–12 mos., MZ	0.66 [0.49, 0.83]
MCTS, Stroganova 2000, Moderate tension, 7–12 mos., MZ	0.85 [0.77, 0.93]
MCTS, Stroganova 2000, Low tension, 7–12 mos., MZ	0.87 [0.80, 0.94]
MCTS, Stroganova 2000, High tension, 7–12 mos., MZ	0.77 [0.65, 0.89]
MCTS, Stroganova 2000, Fear, 7–12 mos., MZ	0.71 [0.56, 0.86]
LTS, Matheny 1989, Emotional tone, 24 mos., MZ	0.87 [0.79, 0.95]
LTS, Matheny 1989, Emotional tone, 18 mos., MZ	0.83 [0.72, 0.94]
LTS, Matheny 1989, Emotional tone, 12 mos., MZ	0.59 [0.36, 0.82]
LTS, Riese 1990, Resistance to soothing, 0–1 mos., MZ	0.06 [−0.23, 0.35]
LTS, Riese 1990, Reactivity, 0–1 mos., MZ	0.12 [−0.17, 0.41]
LTS, Riese 1990, Reactivity, 0–1 mos., MZ	0.12 [−0.17, 0.41]
LTS, Riese 1990, Irritability, 0–1 mos., MZ	0.30 [0.04, 0.56]
LTS, Matheny 1989, Fearfulness, 24 mos., MZ	0.80 [0.68, 0.92]
LTS, Matheny 1989, Fearfulness, 18 mos., MZ	0.77 [0.63, 0.91]
LTS, Matheny 1989, Fearfulness, 12 mos., MZ	0.76 [0.61, 0.91]
LTS, Matheny 1976, Tension, 3–12 mos., MZ	0.63 [0.47, 0.79]
LTS, Matheny 1976, Tension, 18–30 mos., MZ	0.59 [0.40, 0.78]
LTS, Matheny 1976, Reactivity, 18–30 mos., MZ	0.44 [0.21, 0.67]
LTS, Matheny 1976, Reactivity, 3–12 mos., MZ	0.43 [0.21, 0.65]
LTS, Matheny 1976, Fearfulness, 3–12 mos., MZ	0.70 [0.56, 0.84]
LTS, Matheny 1976, Emotional tone, 3–12 mos., MZ	0.64 [0.48, 0.80]
LTS, Matheny 1976, Emotional tone, 18–30 mos., MZ	0.51 [0.30, 0.72]
JEP, Saudino 2008, Negative affect, 24 mos., MZM	0.68 [0.61, 0.75]
JEP, Saudino 2008, Internalizing, 24 mos., MZM	0.79 [0.74, 0.84]
JEP, Saudino 2008, General anxiety, 24 mos., MZM	0.75 [0.69, 0.81]
JEP, Saudino 2008, Depression withdrawal, 24 mos., MZM	0.70 [0.63, 0.77]
JEP, Saudino 2008, Negative affect, 24 mos., MZF	0.75 [0.69, 0.81]
JEP, Saudino 2008, Internalizing, 24 mos., MZF	0.81 [0.76, 0.86]
JEP, Saudino 2008, General anxiety, 24 mos., MZF	0.78 [0.72, 0.84]
JEP, Saudino 2008, Depression withdrawal, 24 mos., MZF	0.76 [0.70, 0.82]
ECLS−B, Jackson 2016, Moody/unusual, 24 mos., MZ	0.68 [0.61, 0.75]
ECLS−B, Jackson 2016, Demanding/angry, 24 mos., MZ	0.67 [0.60, 0.74]
ECLS−B, Roisman 2006, Fussiness and demanding behaviour, 9 mos., MZ	0.48 [0.36, 0.60]
ECLS−B, Roisman 2006, Positive and negative affect, 9 mos., MZ	0.64 [0.55, 0.73]
CTR_TIP, DiLalla 1996, Negative affect, 7 mos., MZ	0.08 [−0.14, 0.30]
CTR_TIP, DiLalla 1996, Negative affect, 9 mos., MZ	−0.06 [−0.30, 0.18]
CTR_LTS, Emde 1992, Frustration, 14 mos., MZ	0.26 [0.07, 0.45]
CTR_LTS, Plomin 1993, Reactivity, 14–20 mos., MZ	0.37 [0.18, 0.56]
CTR_LTS, Plomin 1993, Reactivity, 14–20 mos., MZ	0.26 [0.05, 0.47]
CTR_LTS, Plomin 1993, Reactivity, 20 mos., MZ	0.37 [0.18, 0.56]
CTR_LTS, Plomin 1993, Reactivity, 14 mos., MZ	0.26 [0.07, 0.45]
CTR_LTS, Plomin 1993, Positive hedonic tone, 14 mos., MZ	0.53 [0.38, 0.68]
CTR_LTS, Emde 1992, Positive hedonic tone, 14 mos., MZ	0.53 [0.39, 0.67]
CTR_LTS, Plomin 1993, Positive hedonic tone, 20 mos., MZ	0.56 [0.41, 0.71]
CTR_LTS, Plomin 1993, Positive affect, 14–20 mos., MZ	0.56 [0.41, 0.71]
CTR_LTS, Plomin 1993, Positive affect, 14–20 mos., MZ	0.53 [0.37, 0.69]
CTR_LTS, Plomin 1993, Positive affect, 14–20 mos., MZ	0.84 [0.77, 0.91]
CTR_LTS, Plomin 1993, Positive affect, 14–20 mos., MZ	0.84 [0.77, 0.91]
CTR_LTS, Plomin 1993, Positive affect, 20 mos., MZ	0.84 [0.77, 0.91]
CTR_LTS, Plomin 1993, Positive affect, 14 mos., MZ	0.84 [0.78, 0.90]
CTR_LTS, Emde 1992, Overall mood, 14 mos., MZ	0.19 [−0.00, 0.38]
CTR_LTS, Plomin 1993, Affect, 14–20 mos., MZ	0.27 [0.07, 0.47]
CTR_LTS, Plomin 1993, Affect, 14–20 mos., MZ	0.19 [−0.02, 0.40]
CTR_LTS, Plomin 1993, Overall mood, 20 mos., MZ	0.27 [0.07, 0.47]
CTR_LTS, Plomin 1993, Affect, 14 mos., MZ	0.19 [−0.01, 0.39]
CTR_LTS, Rhee 2012, Negative affect, 14 mos., MZ	0.38 [0.27, 0.49]
CTR_LTS, Rhee 2012, Negative affect, 24 mos., MZ	0.35 [0.23, 0.47]
CTR_LTS, Rhee 2012, Negative affect, 20 mos., MZ	0.34 [0.22, 0.46]
CTR_LTS, Rhee 2012, Negative affect, 24 mos., MZ	0.31 [0.19, 0.43]
CTR_LTS, Rhee 2012, Negative affect, 14 mos., MZ	0.25 [0.13, 0.37]
CTR_LTS, Rhee 2012, Negative affect, 20 mos., MZ	0.15 [0.02, 0.28]
CTR_LTS, Rhee 2012, Negative affect, 14 mos., MZ	0.31 [0.19, 0.43]
CTR_LTS, Rhee 2012, Negative affect, 24 mos., MZ	0.30 [0.18, 0.42]
CTR_LTS, Rhee 2012, Negative affect, 20 mos., MZ	0.29 [0.17, 0.41]
CTR_LTS, Rhee 2012, Negative affect, 24 mos., MZ	0.52 [0.42, 0.62]
CTR_LTS, Rhee 2012, Negative affect, 20 mos., MZ	0.50 [0.40, 0.60]
CTR_LTS, Rhee 2012, Negative affect, 14 mos., MZ	0.28 [0.16, 0.40]
CTR_LTS, Emde 1992, Negative hedonic tone, 14 mos., MZ	0.11 [−0.09, 0.31]
CTR_LTS, Plomin 1993, Negative affect, 14–20 mos., MZ	0.13 [−0.09, 0.35]
CTR_LTS, Plomin 1993, Negative affect, 14–20 mos., MZ	0.11 [−0.11, 0.33]
CTR_LTS, Plomin 1993, Negative affect, 14–20 mos., MZ	0.72 [0.61, 0.83]
CTR_LTS, Plomin 1993, Negative affect, 14–20 mos., MZ	0.71 [0.60, 0.82]
CTR_LTS, Plomin 1993, Negative affect, 20 mos., MZ	0.72 [0.61, 0.83]
CTR_LTS, Plomin 1993, Negative affect, 14 mos., MZ	0.71 [0.61, 0.81]
CTR_LTS, Plomin 1993, Emotionality, 14–20 mos., MZ	0.51 [0.35, 0.67]
CTR_LTS, Plomin 1993, Emotionality, 14–20 mos., MZ	0.35 [0.16, 0.54]
CTR_LTS, Schmitz 1999, Emotionality, 20 mos., MZ	0.51 [0.40, 0.62]
CTR_LTS, Schmitz 1999, Emotionality, 14 mos., MZ	0.42 [0.30, 0.54]
CTR_LTS, Schmitz 1999, Emotionality, 24 mos., MZ	0.37 [0.24, 0.50]
CTR_LTS, Plomin 1993, Affect, 14–20 mos., MZ	0.33 [0.13, 0.53]
CTR_LTS, Plomin 1993, Affect, 14–20 mos., MZ	0.25 [0.04, 0.46]
CTR_LTS, Plomin 1993, Affect, 20 mos., MZ	0.33 [0.13, 0.53]
CTR_LTS, Emde 1992, Affect, 14 mos., MZ	0.25 [0.07, 0.43]
BUTP, Micalizzi 2016, Affective problems, 24 mos., MZ	0.65 [0.56, 0.74]
RE Model for MZ Subset	0.58 [0.49, 0.67]

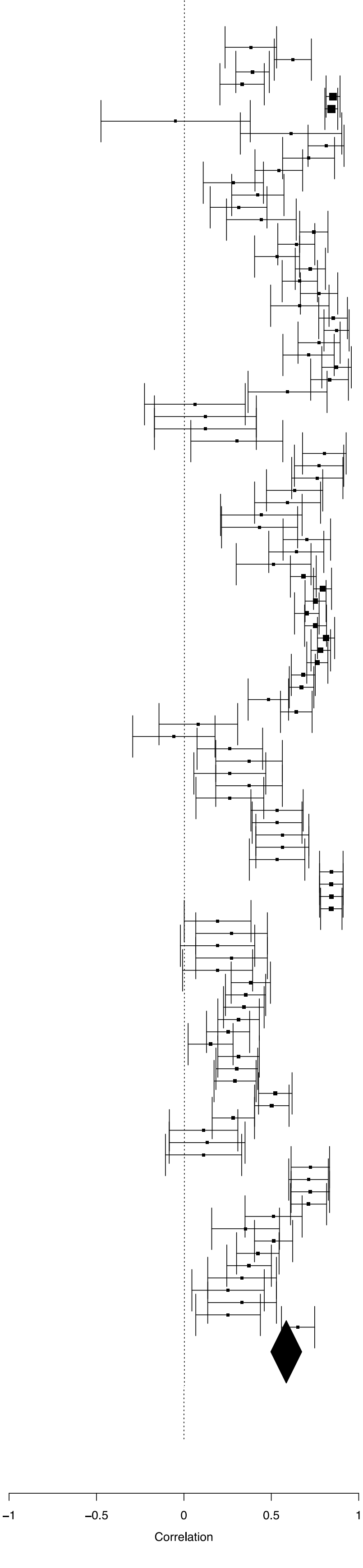

© 2022 Austerberry C et al. *JAMA Network Open.*

eFigure 7 (Continued)

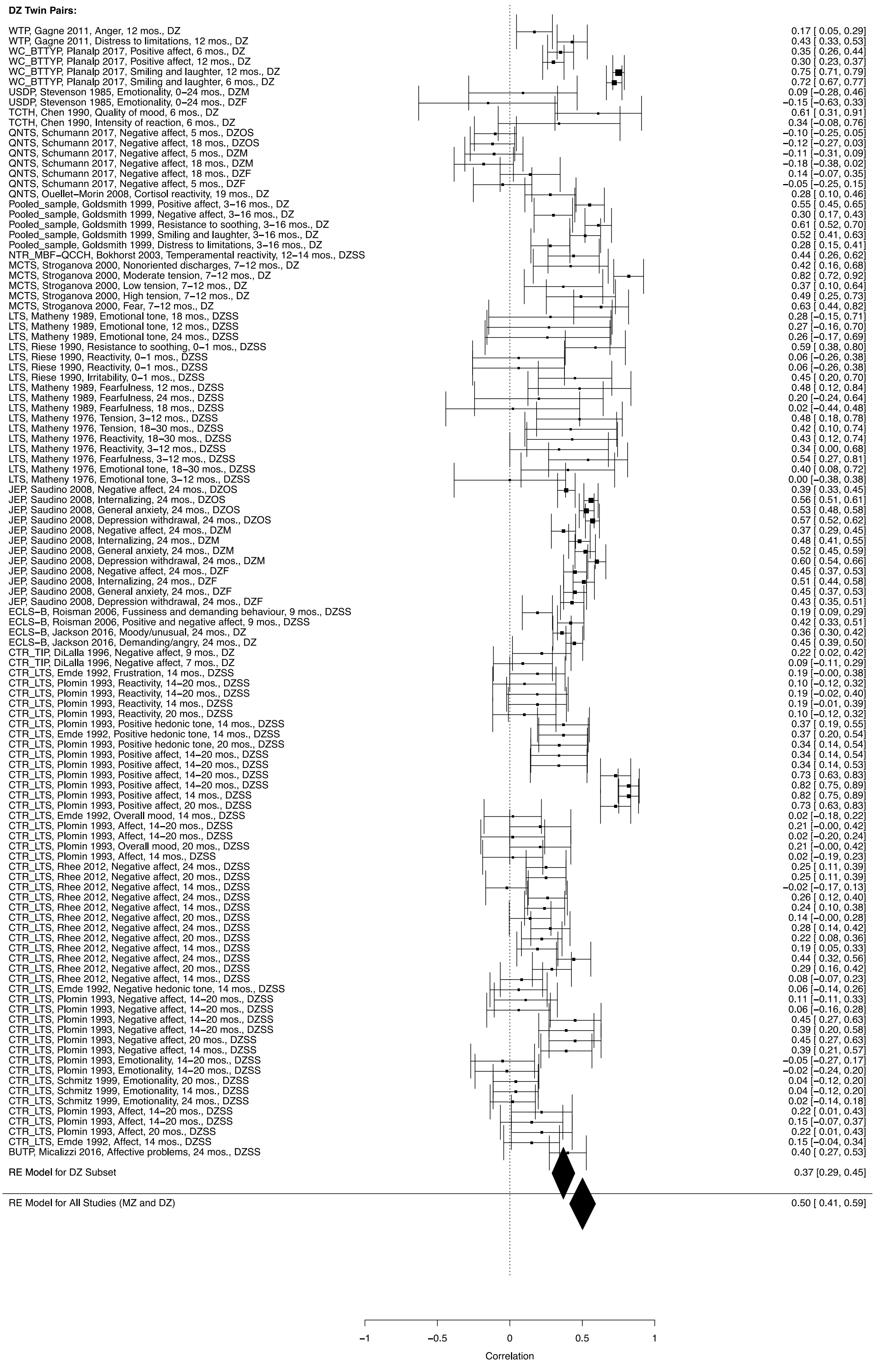

© 2022 Austerberry C et al. *JAMA Network Open.*

eFigure 8. Basic Cognitive Functions Forest Plot

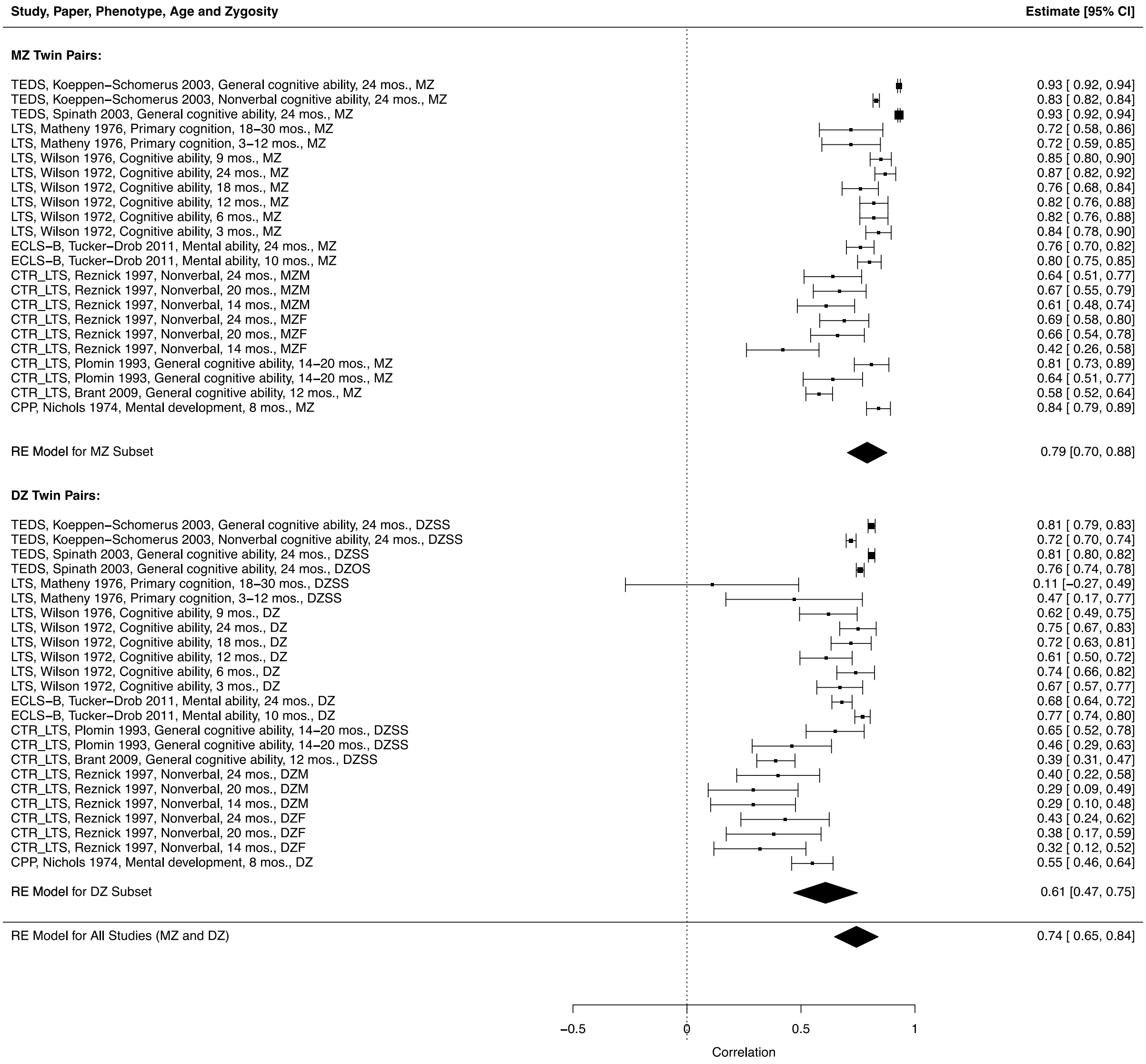

© 2022 Austerberry C et al. *JAMA Network Open.*

eFigure 9. Mental Functions of Language Forest Plot

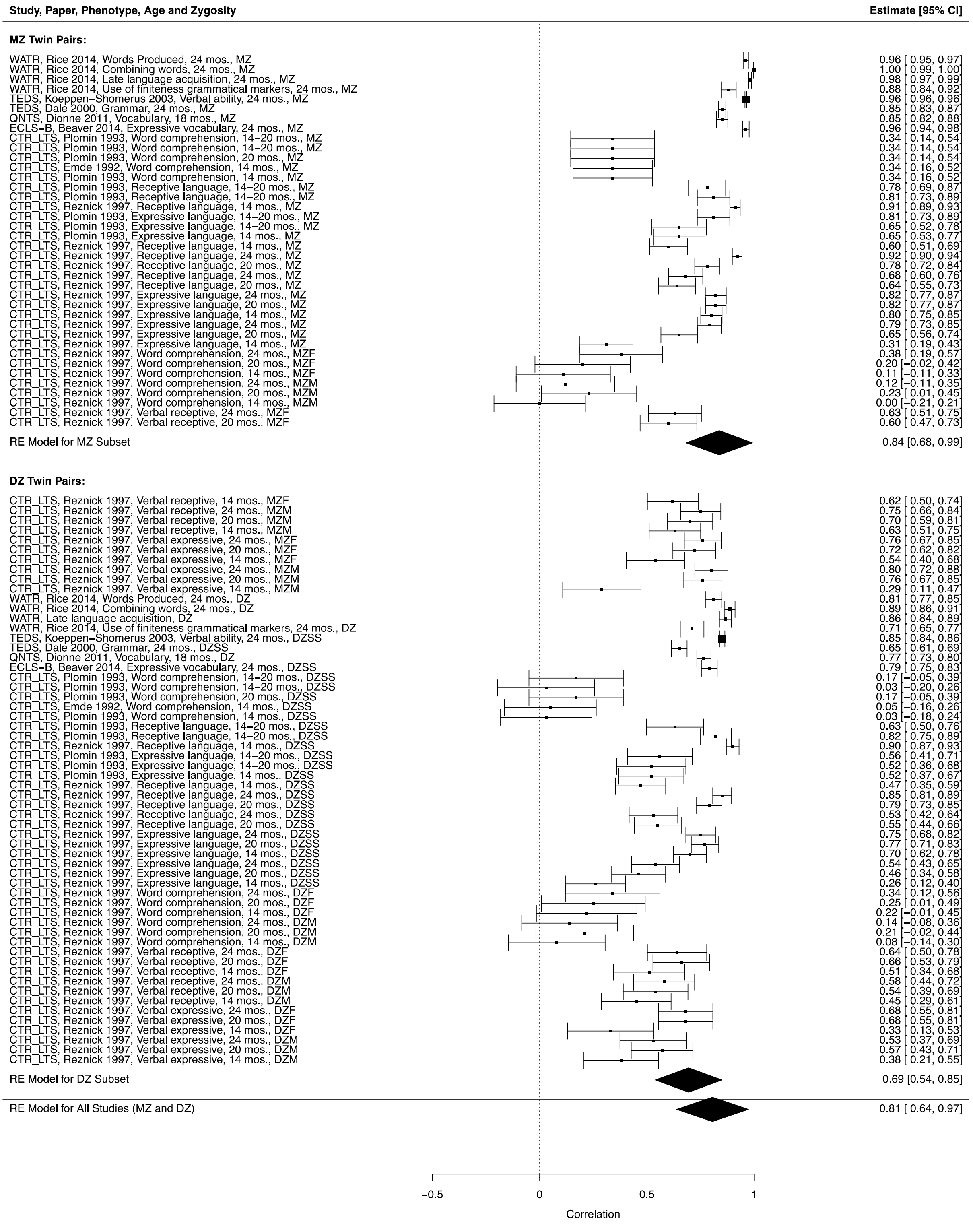

© 2022 Austerberry C et al. *JAMA Network Open.*

eFigure 10. Growth Maintenance Functions Forest Plot

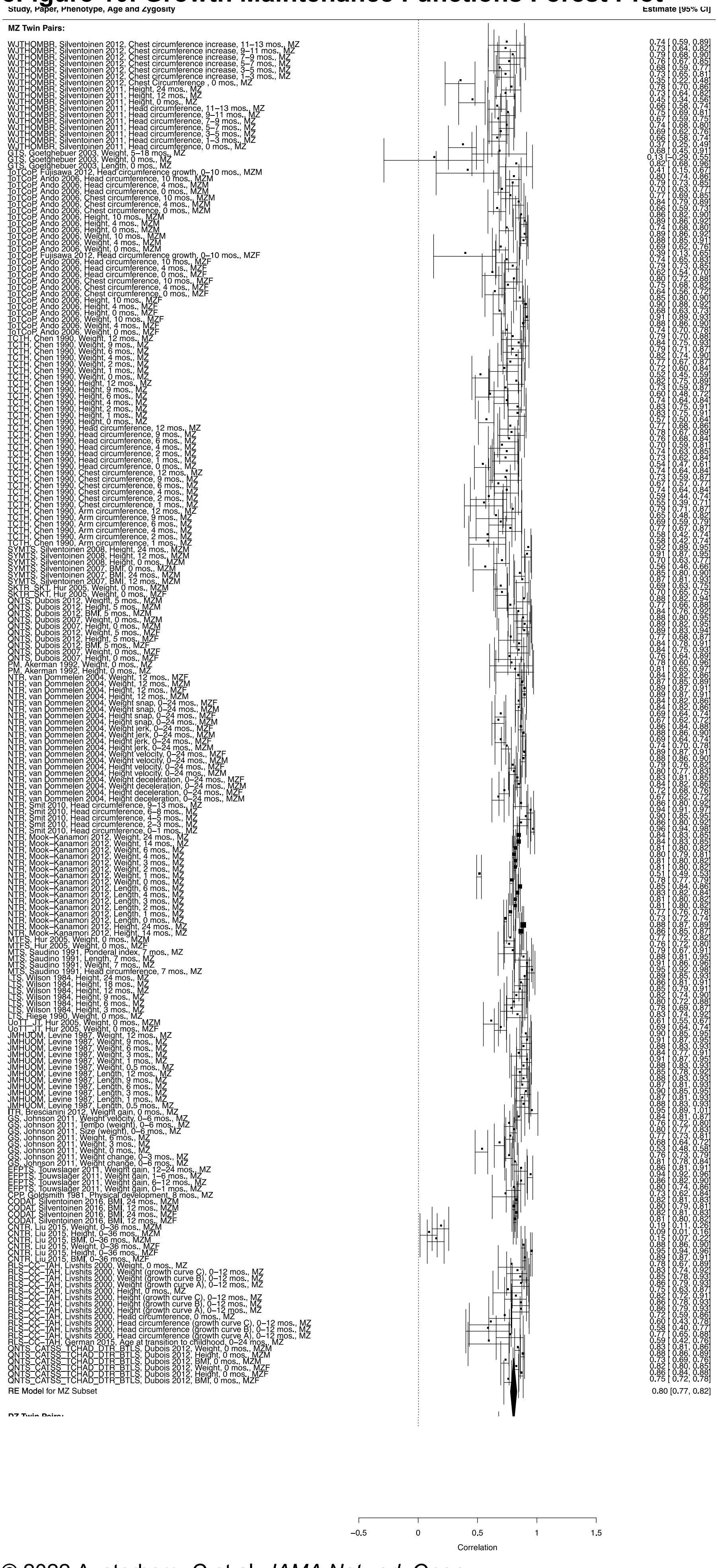

© 2022 Austerberry C et al. *JAMA Network Open.*

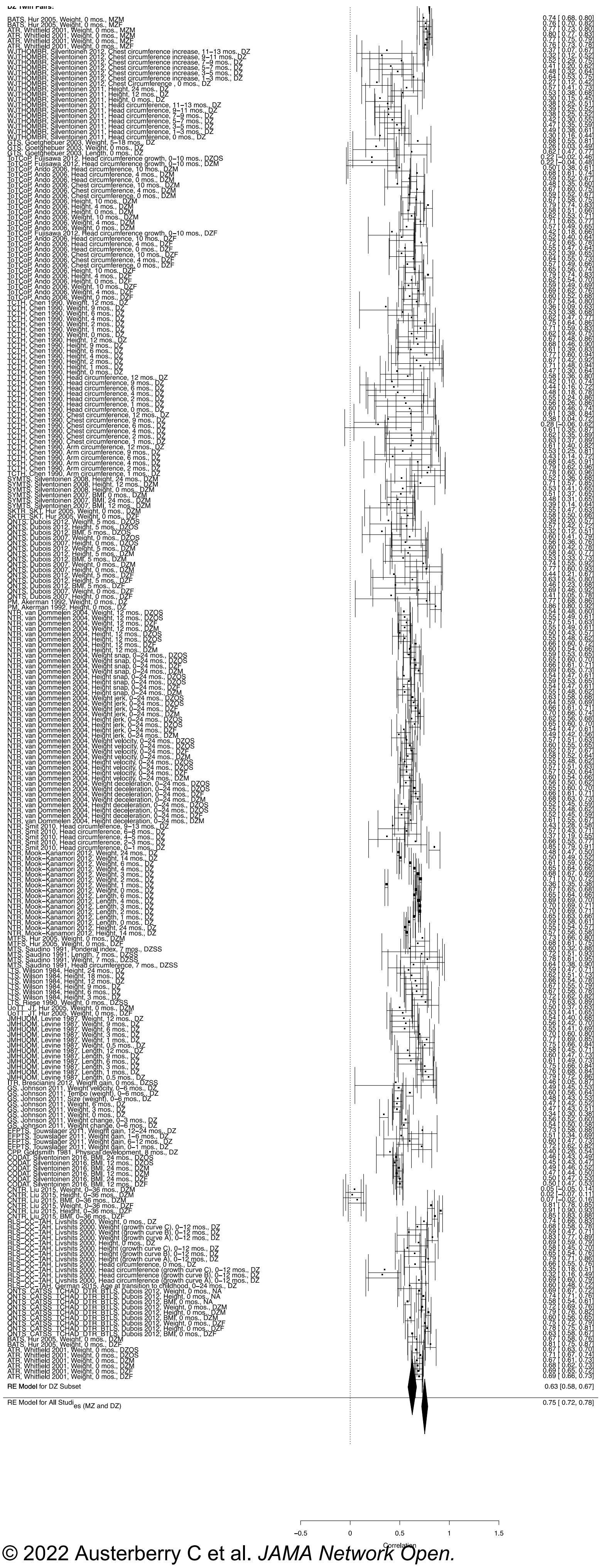

© 2022 Austerberry C et al. *JAMA Network Open.*

eFigure 11. Basic Interpersonal Interactions Forest Plot

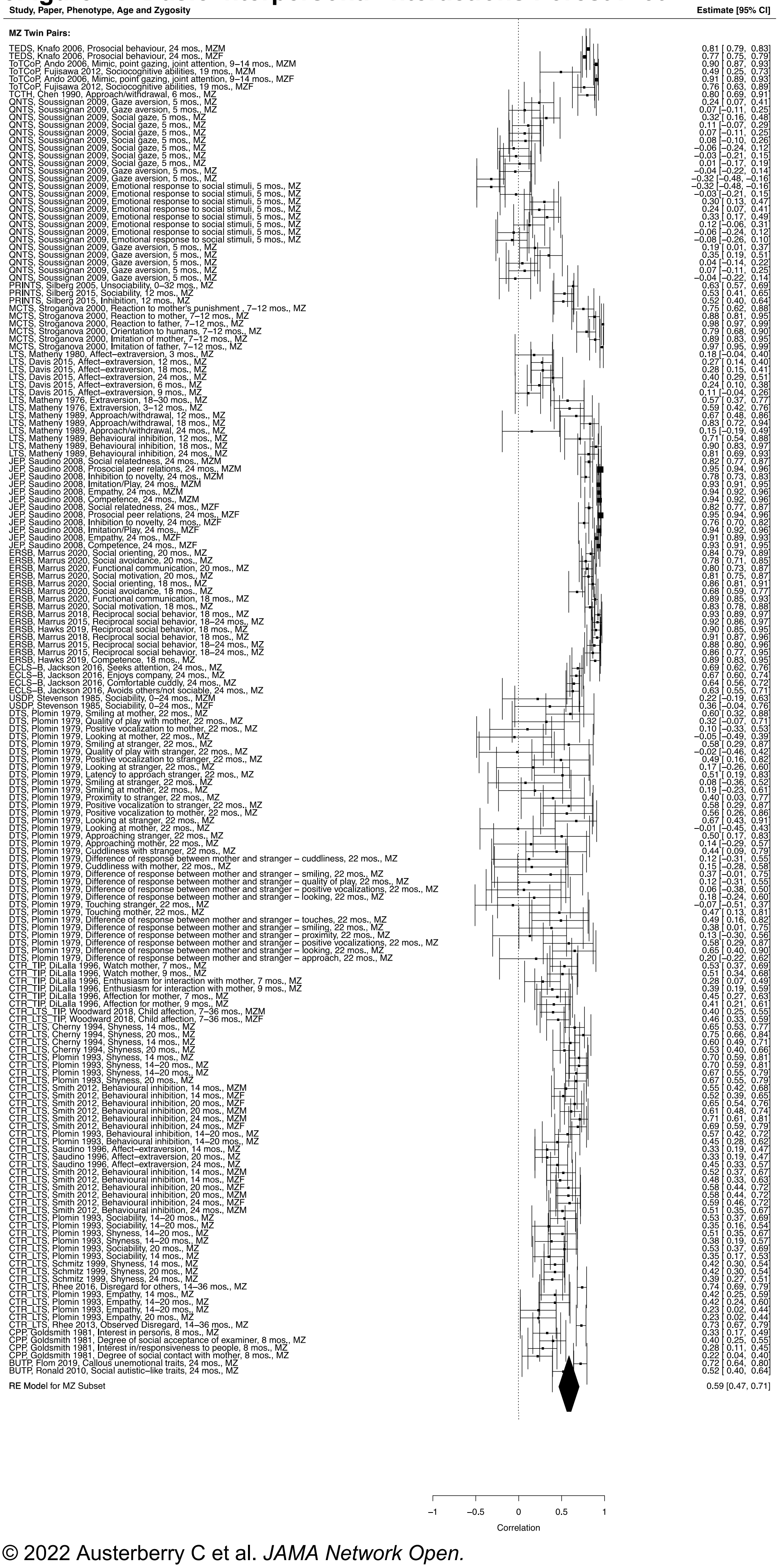

© 2022 Austerberry C et al. *JAMA Network Open.*

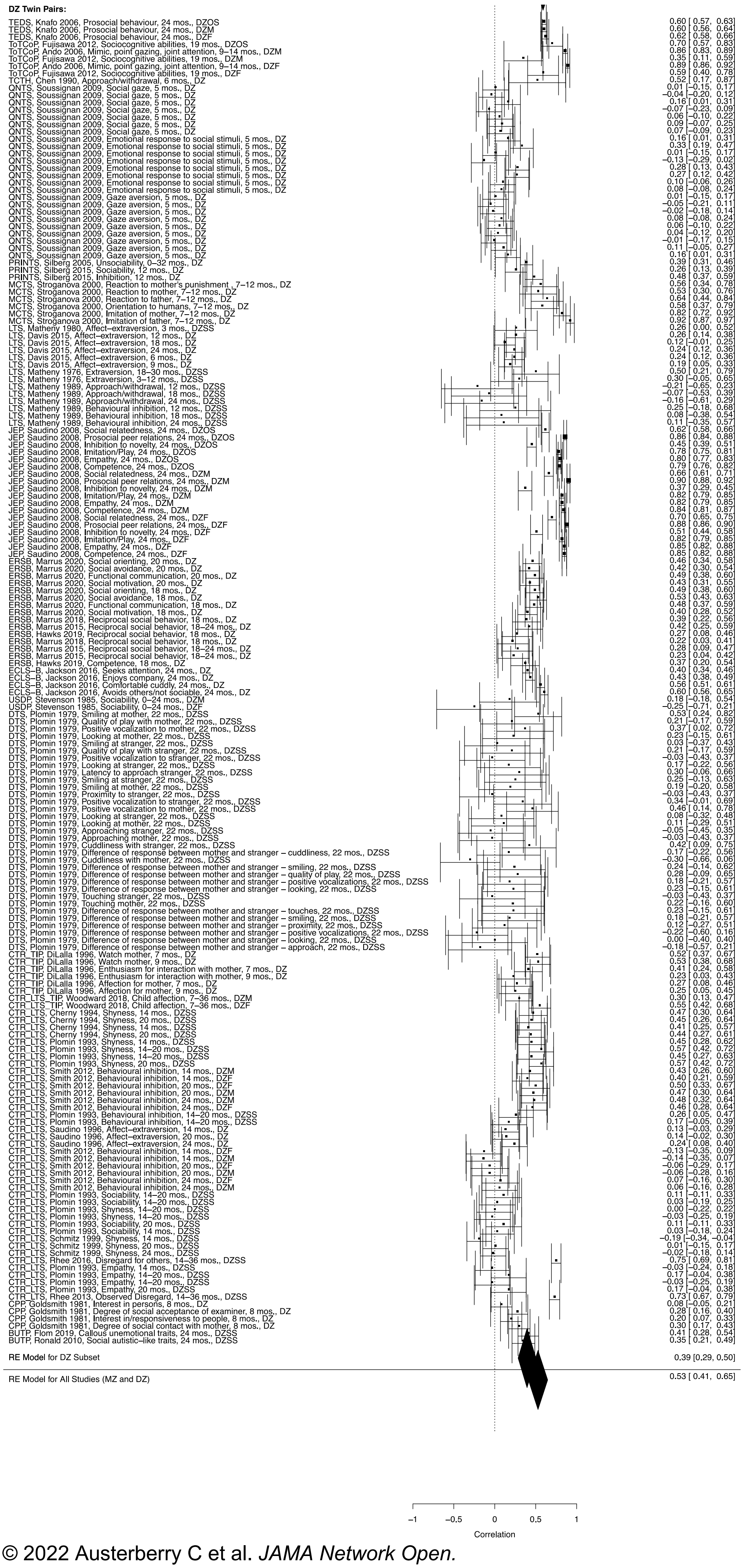

© 2022 Austerberry C et al. *JAMA Network Open.*

eFigure 12. Complex Interpersonal Interactions Forest Plot

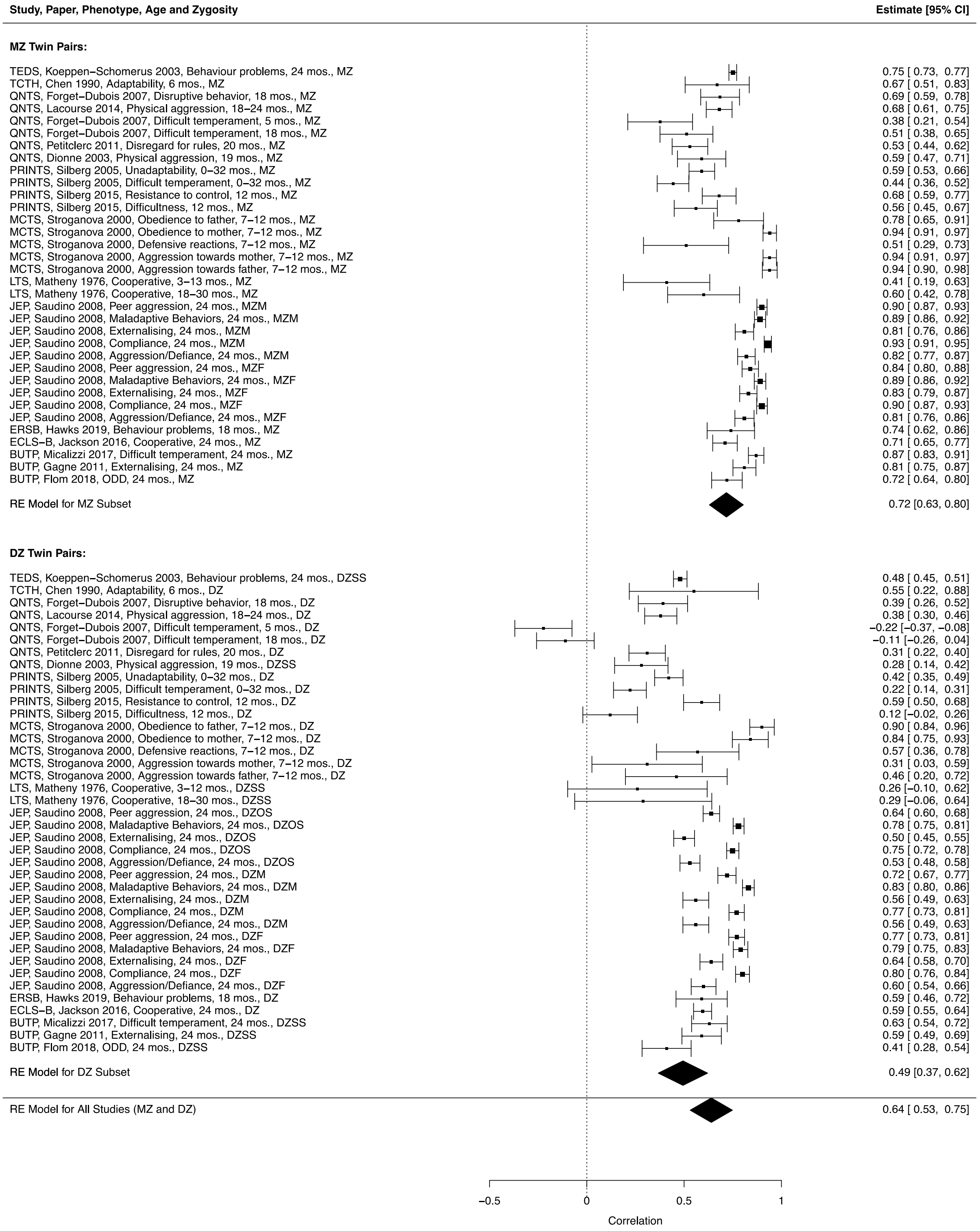

© 2022 Austerberry C et al. *JAMA Network Open.*

eFigure 13. Family Relationships Forest Plot

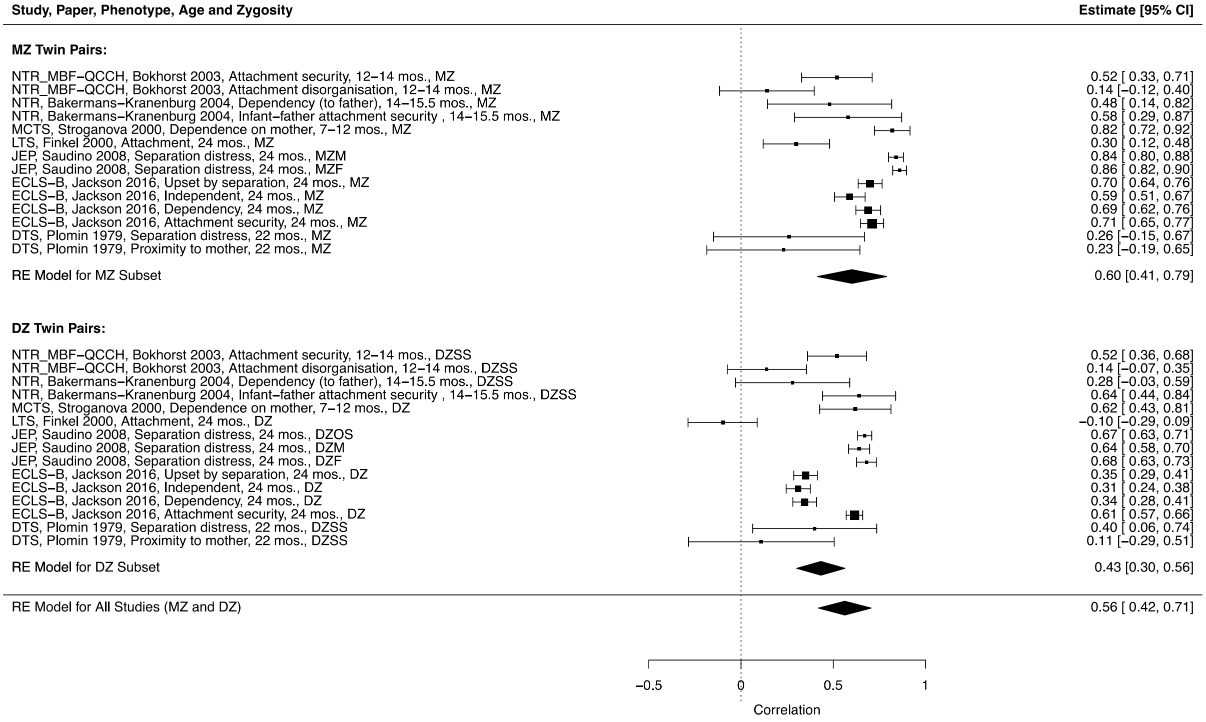

© 2022 Austerberry C et al. *JAMA Network Open.*

eFigure 14. Funnel Plots of Association Between Monozygotic Twin Correlation and Standard Error in Phenotype Categories With ≥10 Estimates

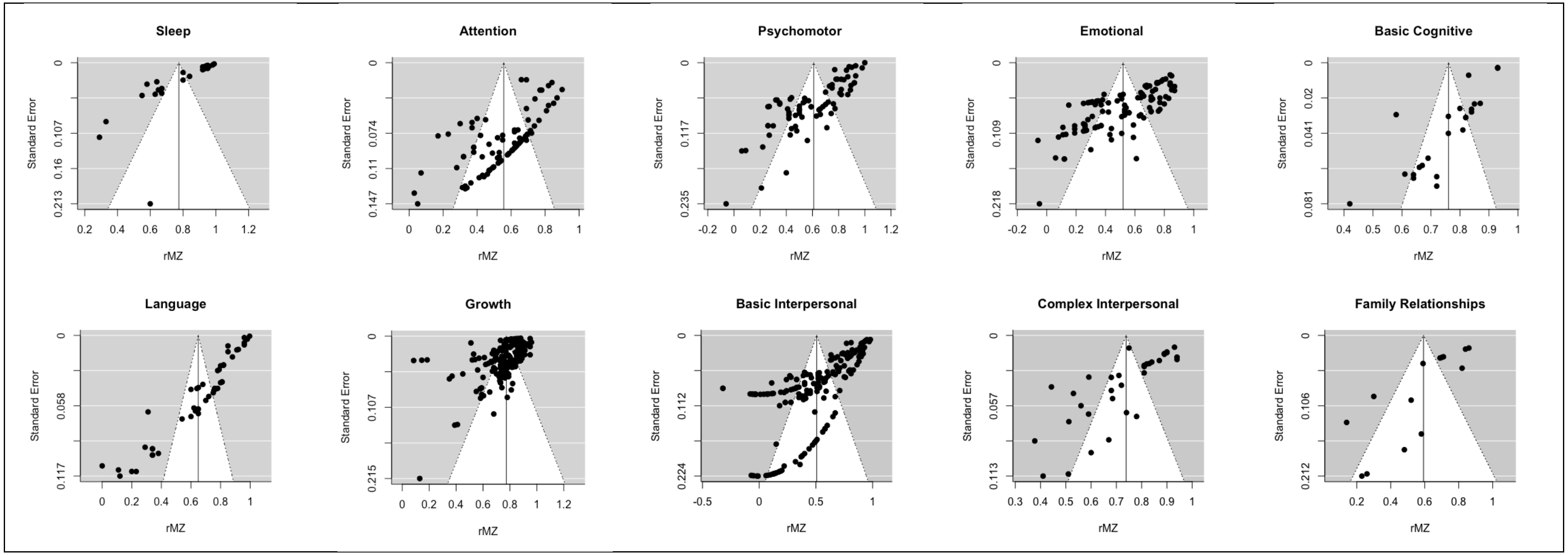

© 2022 Austerberry C et al. *JAMA Network Open.*

eFigure 15. Funnel Plots of Association Between Dizygotic Twin Correlation and Standard Error in Phenotype Categories With ≥10 Estimates

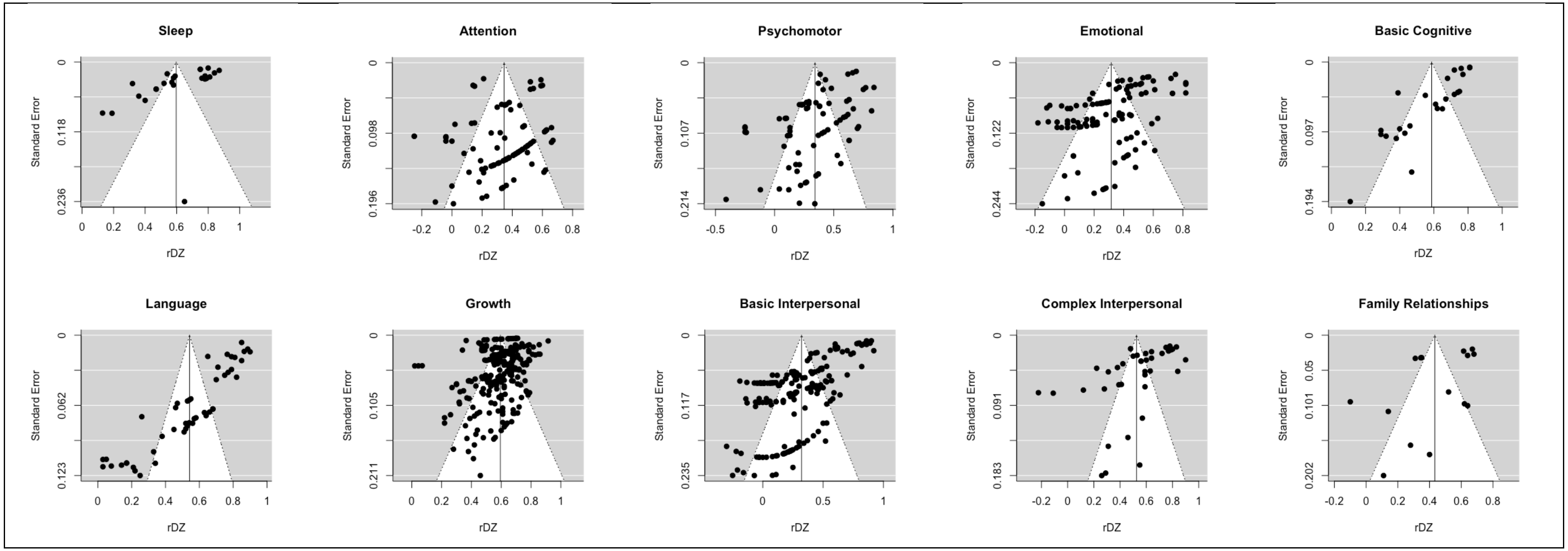

Note. r_{DZ} = dizygotic twin correlation

© 2022 Austerberry C et al. *JAMA Network Open.*

eFigure 16. Funnel Plots of Association Between Heritability and Standard Error in Phenotype Categories With ≥10 Estimates

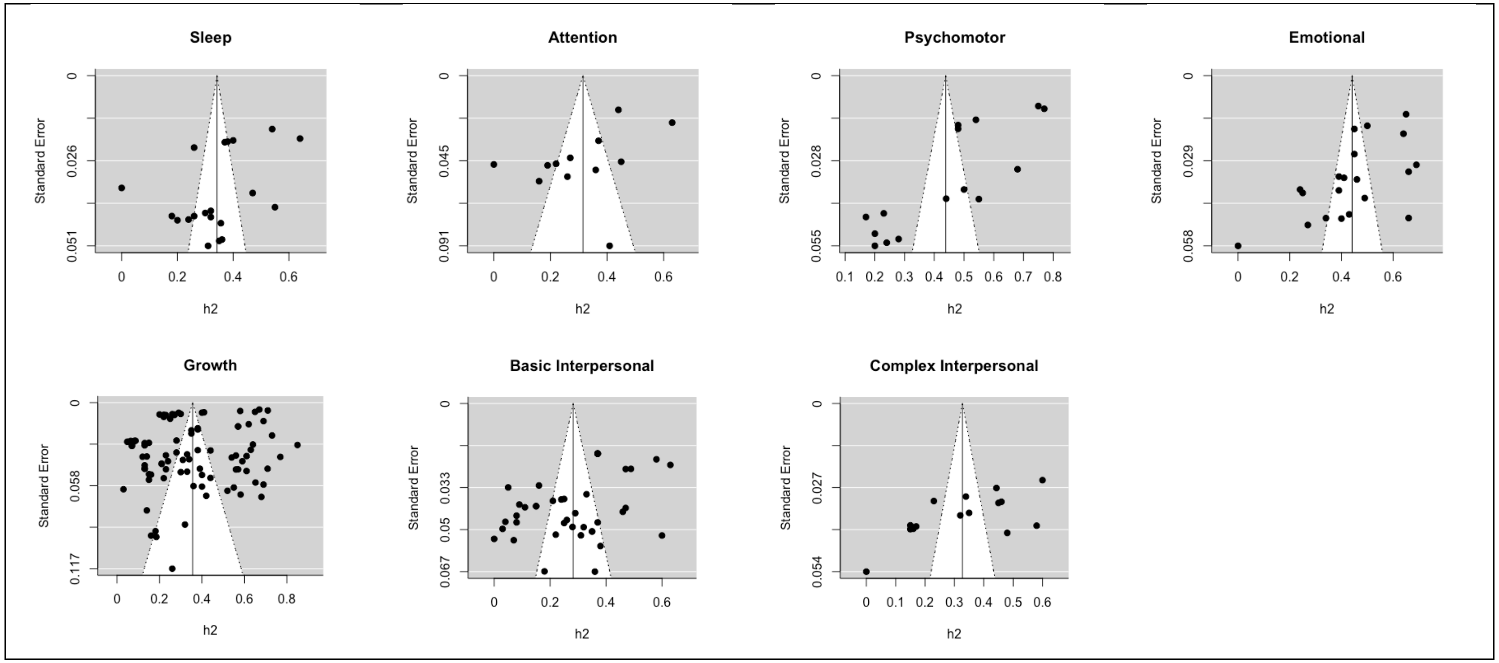

© 2022 Austerberry C et al. *JAMA Network Open.*

eFigure 17. Funnel Plots of Association Between Shared Environment and Standard Error in Phenotype Categories With ≥10 Estimates

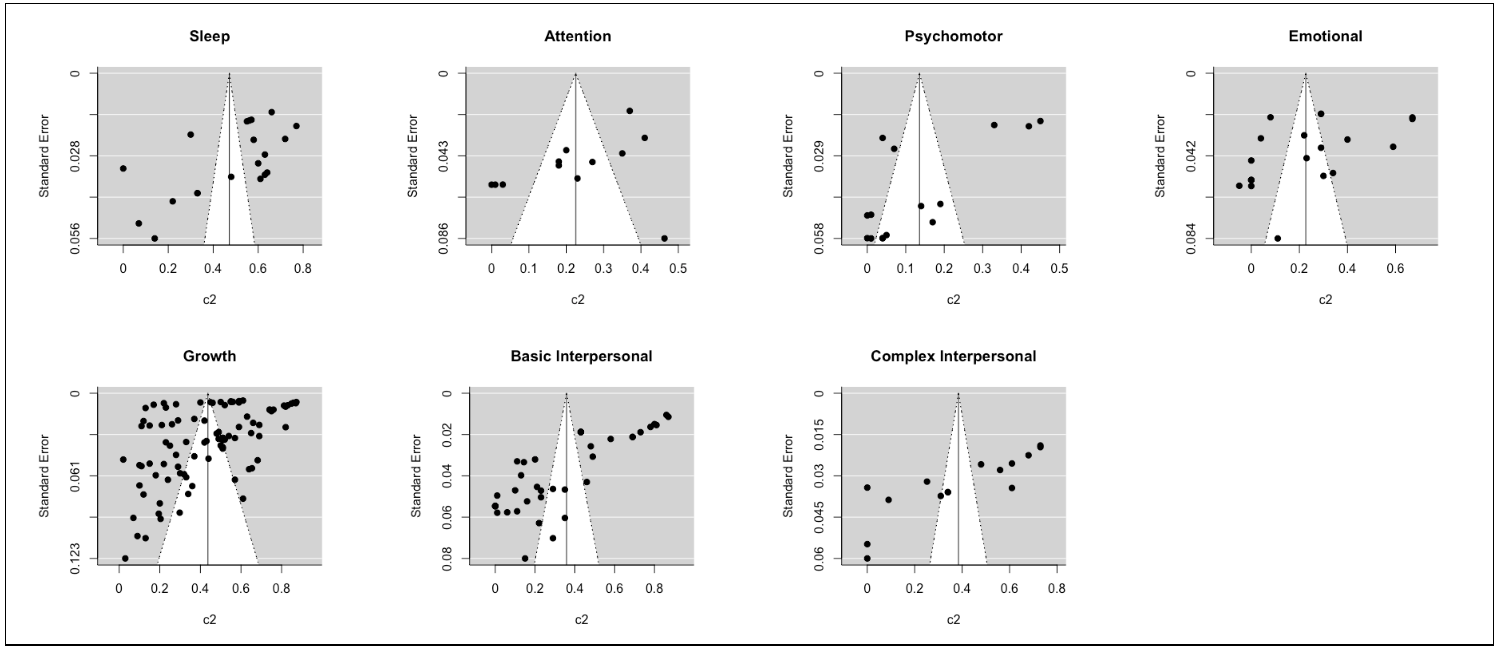

© 2022 Austerberry C et al. *JAMA Network Open.*

eFigure 18. Funnel Plots of Association Between Nonshared Environment and Standard Error in Phenotype Categories With ≥10 Estimates

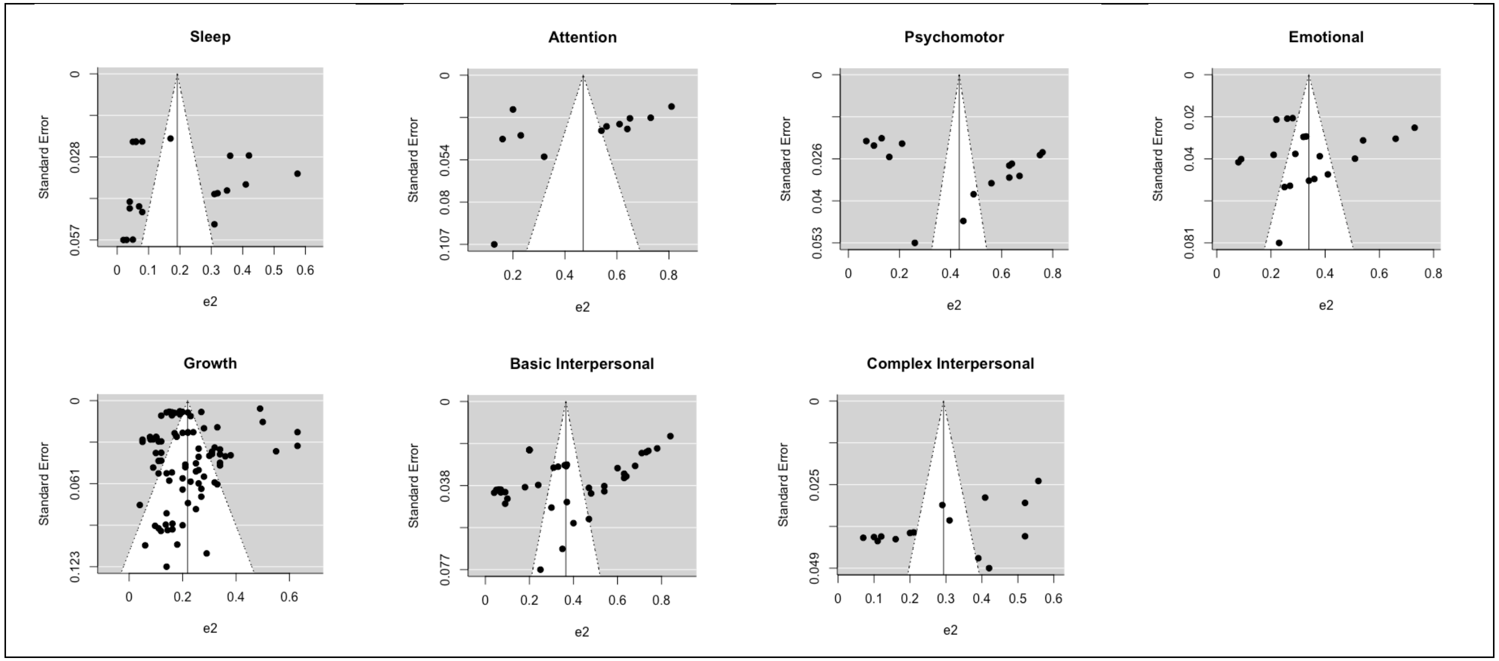

© 2022 Austerberry C et al. *JAMA Network Open.*

eFigure 19. Bar Plot of Quality Assessment Scores

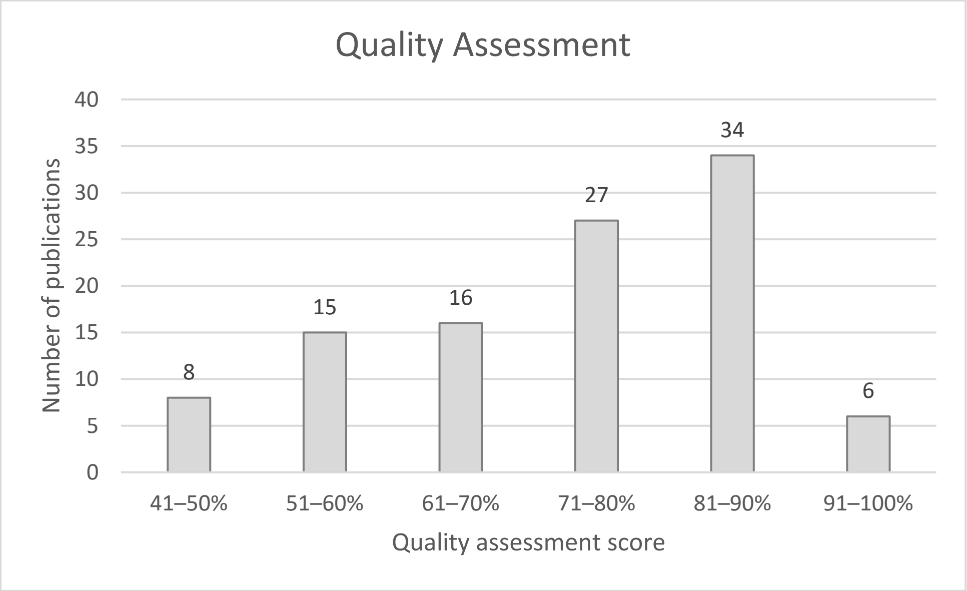

© 2022 Austerberry C et al. *JAMA Network Open.*

eFigure 20. Sustaining Attention Forest Plot

Study, Paper, Phenotype, Age and Zygosity	Estimate [95% CI]
**MZ Twin Pairs:**	
TEDS, Price 2005, ADHD, 24 mos., MZM	0.69 [0.38, 1.00]
TEDS, Price 2005, ADHD, 24 mos., MZF	0.66 [0.36, 0.96]
TCTH, Chen 1990, Distractibility, 6 mos., MZ	0.72 [0.08, 1.36]
TCTH, Chen 1990, Attention/persistence, 6 mos., MZ	0.85 [0.29, 1.41]
Pooled_sample, Goldsmith 1999, Duration of orienting, 3–16 mos., MZ	0.69 [0.19, 1.19]
MCTS, Stroganova 2000, Object orientation, 7–12 mos., MZ	0.87 [0.33, 1.41]
LTS, Matheny 1980, Task orientation, 9 mos., MZ	0.28 [−0.38, 0.94]
LTS, Matheny 1980, Task orientation, 3 mos., MZ	0.55 [−0.06, 1.16]
LTS, Davis 2015, Task orientation, 24 mos., MZ	0.30 [−0.21, 0.81]
LTS, Davis 2015, Task orientation, 18 mos., MZ	0.37 [−0.14, 0.88]
LTS, Davis 2015, Task orientation, 12 mos., MZ	0.40 [−0.09, 0.89]
LTS, Davis 2015, Task orientation, 6 mos., MZ	0.45 [−0.06, 0.96]
LTS, Matheny 1983, Task orientation, 12–24 mos., MZ	0.49 [−0.16, 1.14]
LTS, Matheny 1983, Task orientation, 6–18 mos., MZ	0.53 [−0.12, 1.18]
LTS, Matheny 1976, Object orientation, 18–30 mos., MZ	0.90 [0.40, 1.40]
LTS, Matheny 1976, Object orientation, 3–12 mos., MZ	0.82 [0.27, 1.37]
LTS, Matheny 1976, Looking, 18–30 mos., MZ	0.33 [−0.41, 1.07]
LTS, Matheny 1976, Looking, 3–12 mos., MZ	0.03 [−0.70, 0.76]
LTS, Matheny 1976, Listening, 18–30 mos., MZ	0.05 [−0.71, 0.81]
LTS, Matheny 1976, Listening, 3–12 mos., MZ	0.65 [0.02, 1.28]
LTS, Matheny 1976, Goal directedness, 18–30 mos., MZ	0.46 [−0.25, 1.17]
LTS, Matheny 1976, Goal directedness, 3–12 mos., MZ	0.53 [−0.14, 1.20]
LTS, Matheny 1976, Endurance, 18–30 mos., MZ	0.34 [−0.39, 1.07]
LTS, Matheny 1976, Endurance, 3–12 mos., MZ	0.62 [−0.02, 1.26]
LTS, Matheny 1976, Attention, 18–30 mos., MZ	0.44 [−0.28, 1.16]
LTS, Matheny 1976, Attention, 3–12 mos., MZ	0.52 [−0.15, 1.19]
JEP, Saudino 2008, Mastery Motivation, 24 mos., MZM	0.84 [0.46, 1.22]
JEP, Saudino 2008, Attention, 24 mos., MZM	0.77 [0.35, 1.19]
JEP, Saudino 2008, Mastery Motivation, 24 mos., MZF	0.78 [0.36, 1.20]
JEP, Saudino 2008, Attention, 24 mos., MZF	0.82 [0.42, 1.22]
CTR_TIP, DiLalla 1996, Task orientation, 9 mos., MZ	0.43 [−0.22, 1.08]
CTR_TIP, DiLalla 1996, Task orientation, 7 mos., MZ	0.07 [−0.60, 0.74]
CTR_LTS, Reznick 1997, Visual attentiveness, 24 mos., MZF	0.62 [0.03, 1.21]
CTR_LTS, Reznick 1997, Visual attentiveness, 20 mos., MZF	0.70 [0.13, 1.27]
CTR_LTS, Reznick 1997, Visual attentiveness, 14 mos., MZF	0.55 [−0.04, 1.14]
CTR_LTS, Reznick 1997, Visual attentiveness, 24 mos., MZM	0.62 [0.02, 1.22]
CTR_LTS, Reznick 1997, Visual attentiveness, 20 mos., MZM	0.64 [0.05, 1.23]
CTR_LTS, Reznick 1997, Visual attentiveness, 14 mos., MZM	0.52 [−0.07, 1.11]
CTR_LTS, Saudino 1996, Task orientation, 24 mos., MZ	0.23 [−0.31, 0.77]
CTR_LTS, Saudino 1996, Task orientation, 20 mos., MZ	0.37 [−0.16, 0.90]
CTR_LTS, Saudino 1996, Task orientation, 14 mos., MZ	0.17 [−0.38, 0.72]
CTR_LTS, Plomin 1993, Task orientation, 14–20 mos., MZ	0.47 [−0.14, 1.08]
CTR_LTS, Plomin 1993, Attention/persistence, 14–20 mos., MZ	0.32 [−0.31, 0.95]
CTR_LTS, Plomin 1993, Attention/persistence, 14–20 mos., MZ	0.38 [−0.24, 1.00]
CTR_LTS, Plomin 1993, Attention/persistence, 20 mos., MZ	0.32 [−0.31, 0.95]
CTR_LTS, Plomin 1993, Attention/persistence, 14 mos., MZ	0.38 [−0.23, 0.99]
CTR_LTS, Emde 1992, Attention/persistence, 14 mos., MZ	0.38 [−0.23, 0.99]
CPP, Goldsmith 1981, Pursuit persistence, 8 mos., MZ	0.42 [−0.15, 0.99]
BUTP, Flom 2018, ADHD, 24 mos., MZ	0.74 [0.28, 1.20]
RE Model for MZ Subset	0.60 [0.47, 0.72]

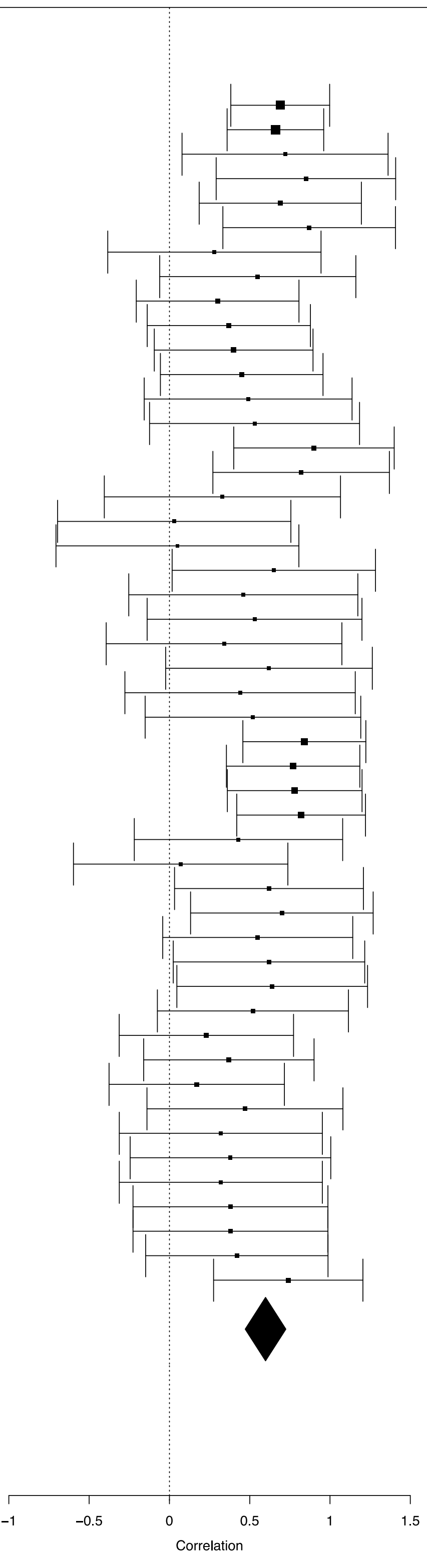

© 2022 Austerberry C et al. *JAMA Network Open.*

eFigure 20 (Continued)

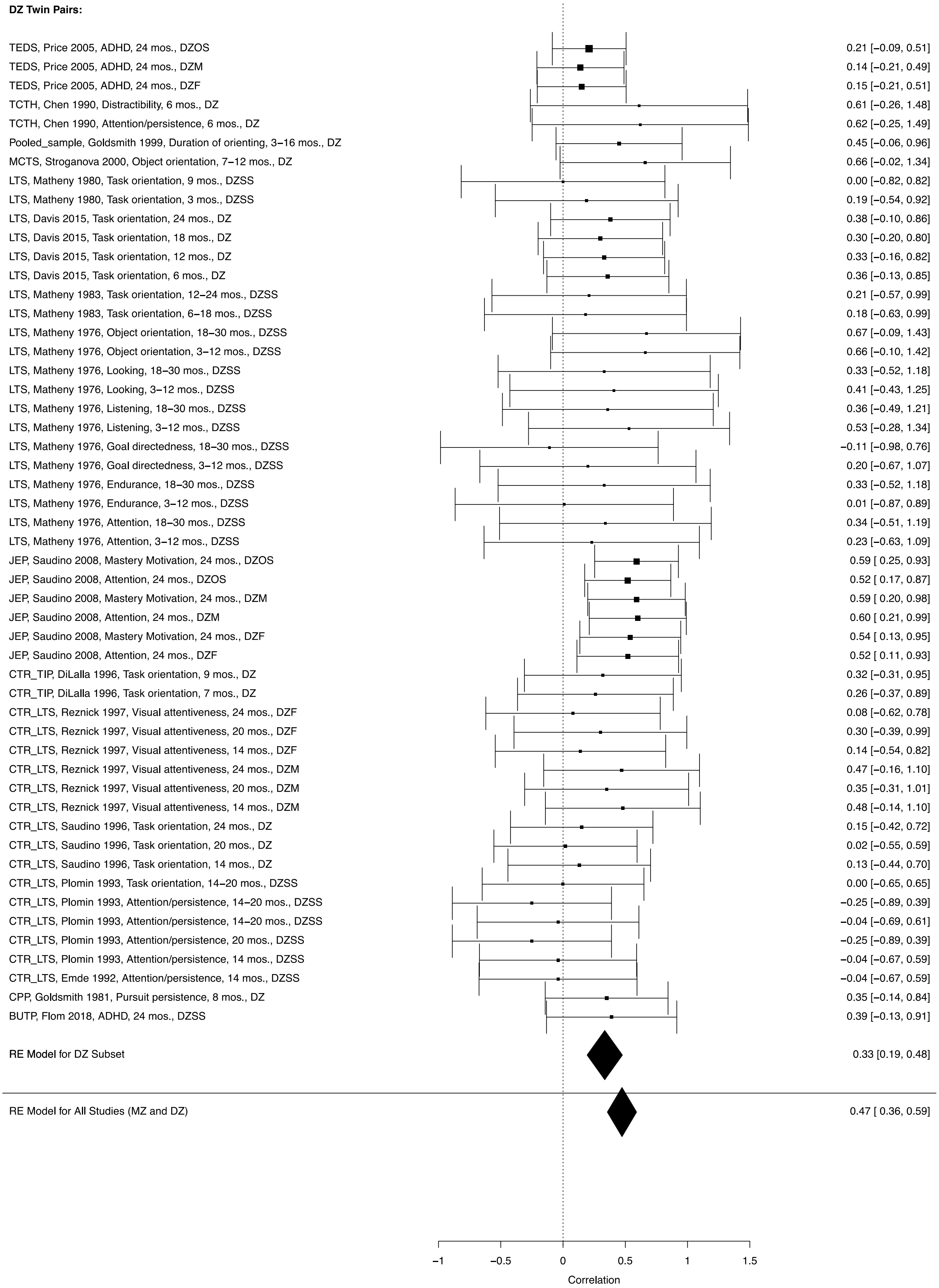

© 2022 Austerberry C et al. *JAMA Network Open.*

eFigure 21. Psychomotor Control Forest Plot

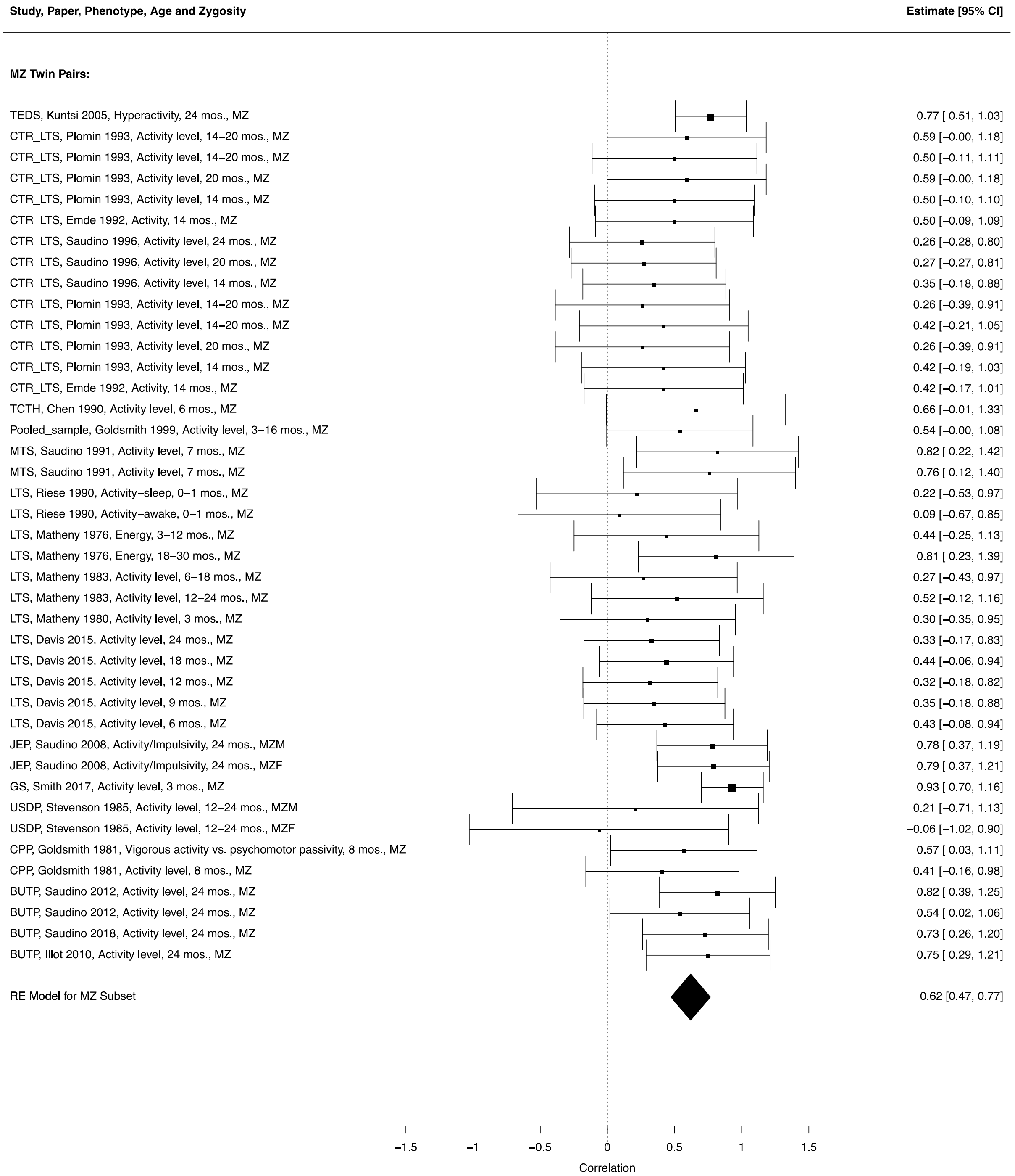

© 2022 Austerberry C et al. *JAMA Network Open.*

eFigure 21 (Continued)

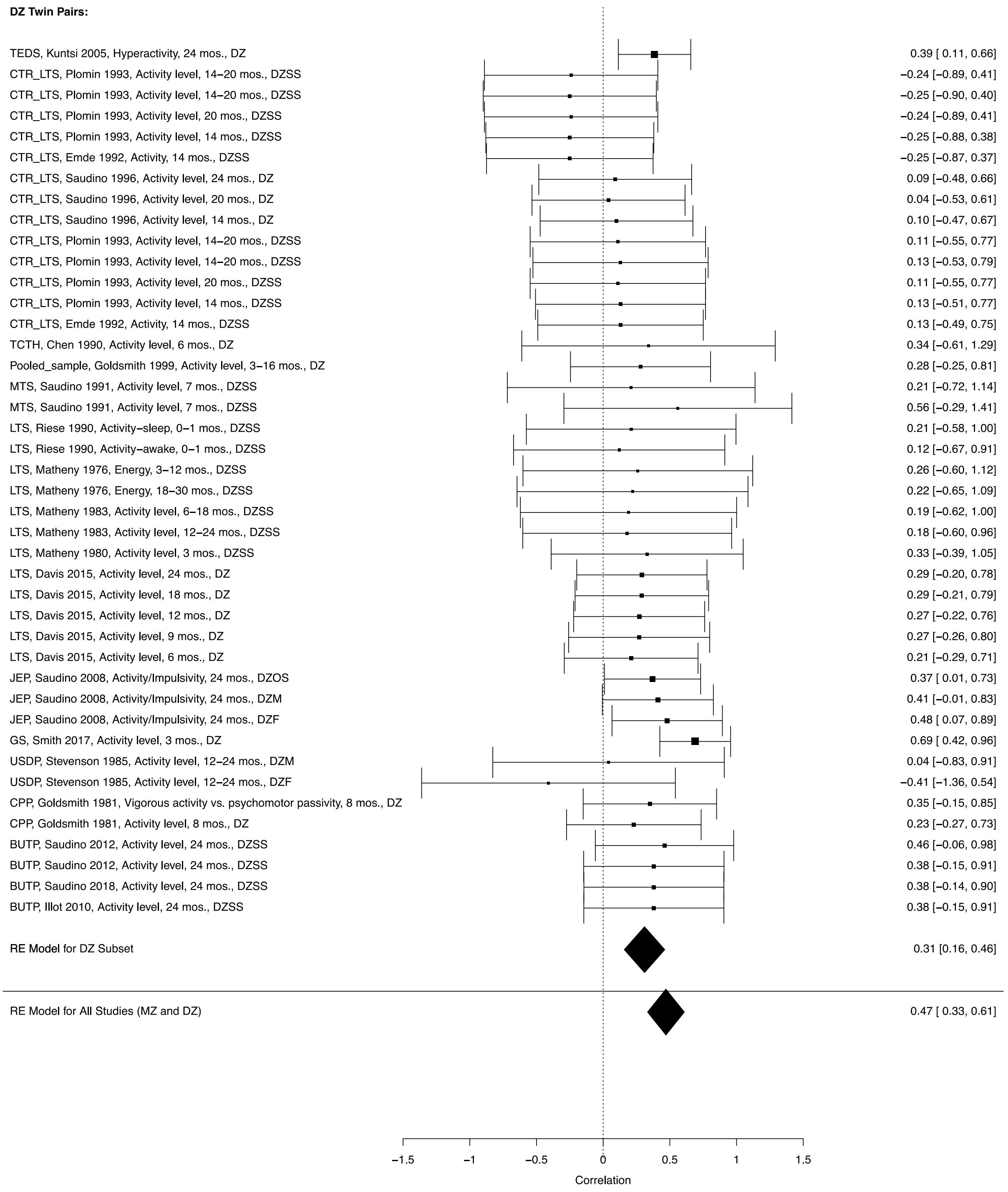

© 2022 Austerberry C et al. *JAMA Network Open.*

eFigure 22. Organization of Psychomotor Functions Forest Plot

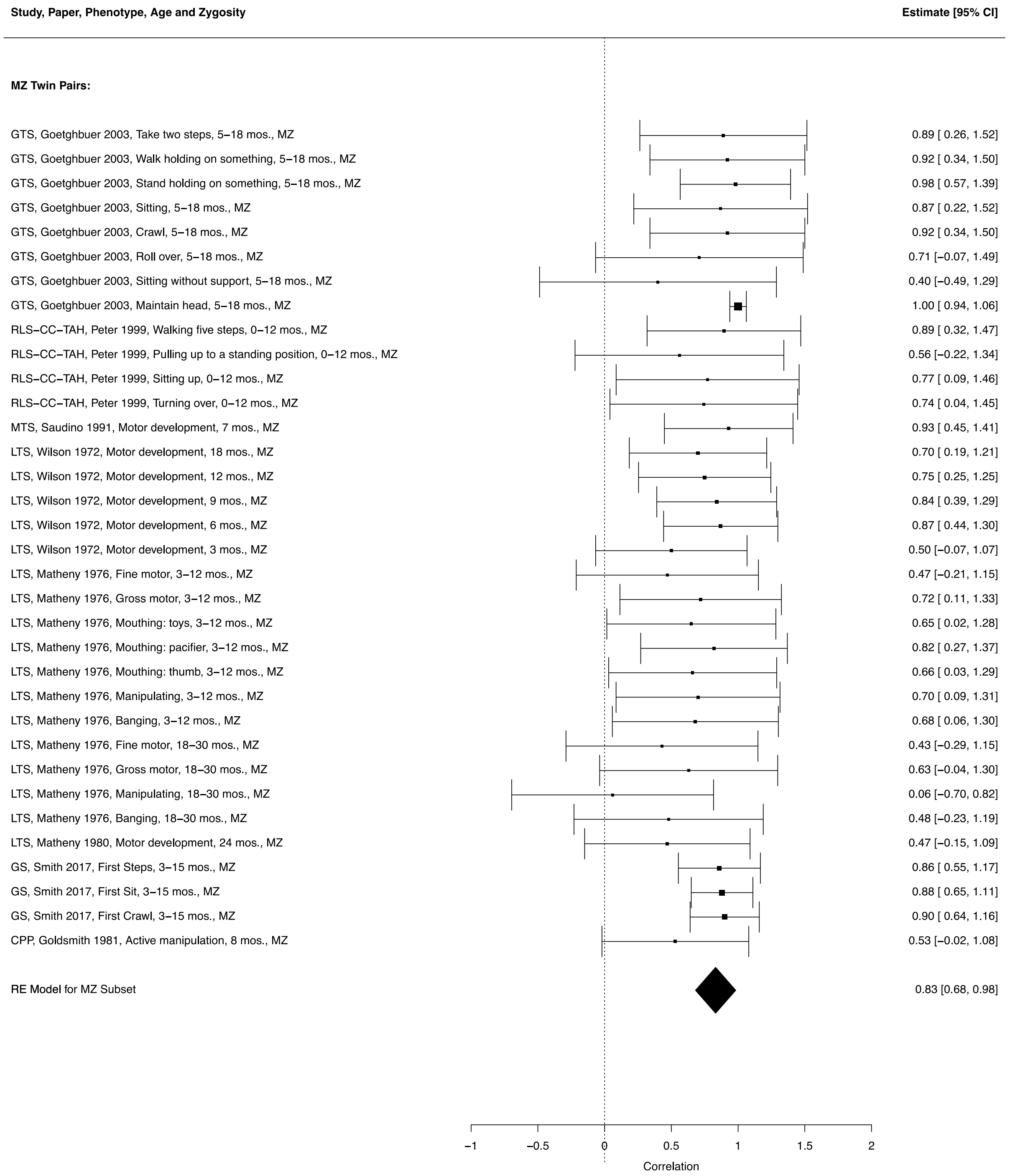

© 2022 Austerberry C et al. *JAMA Network Open.*

eFigure 22 (Continued)

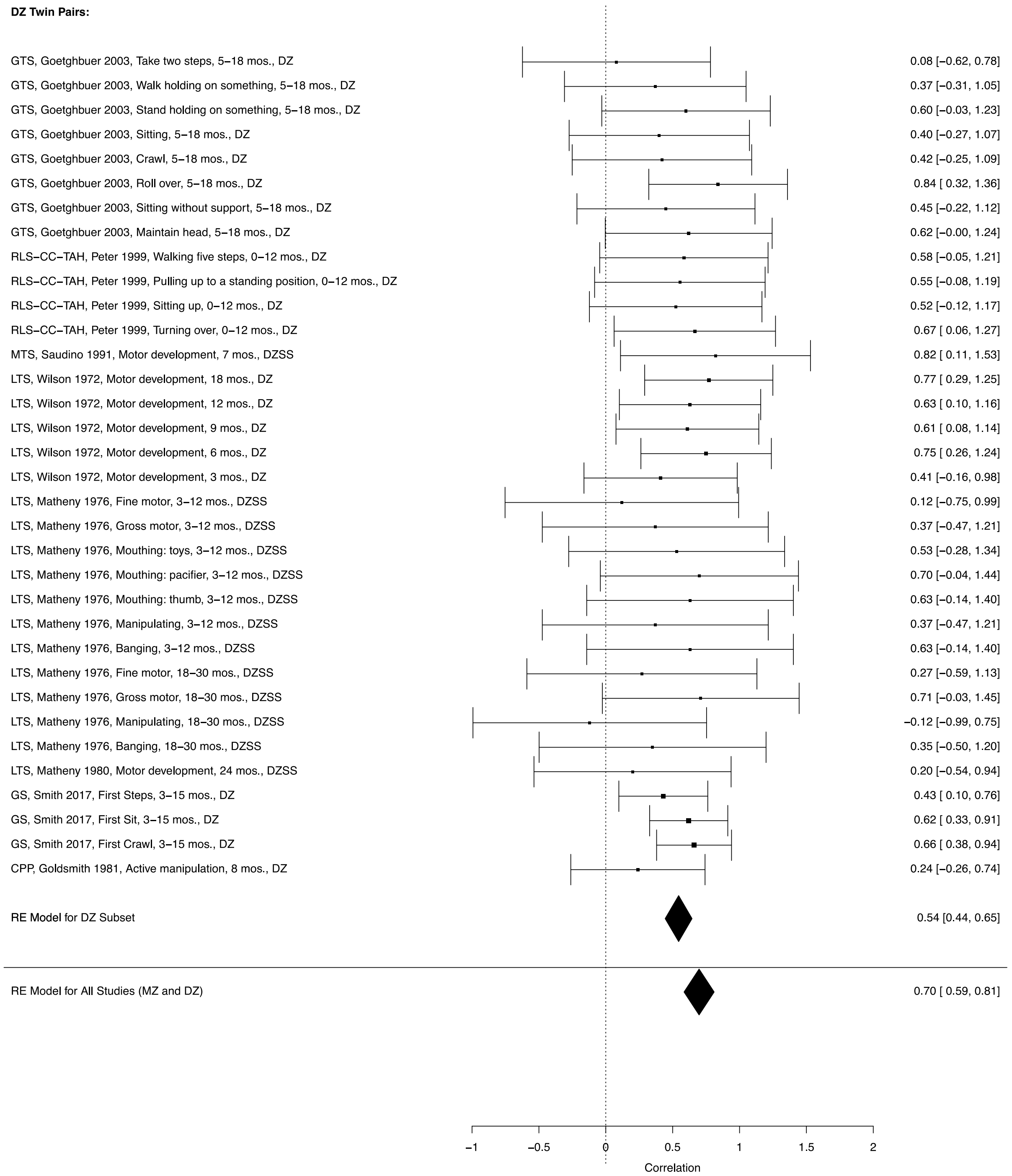

© 2022 Austerberry C et al. *JAMA Network Open.*

eFigure 23. Regulation of Emotion Forest Plot

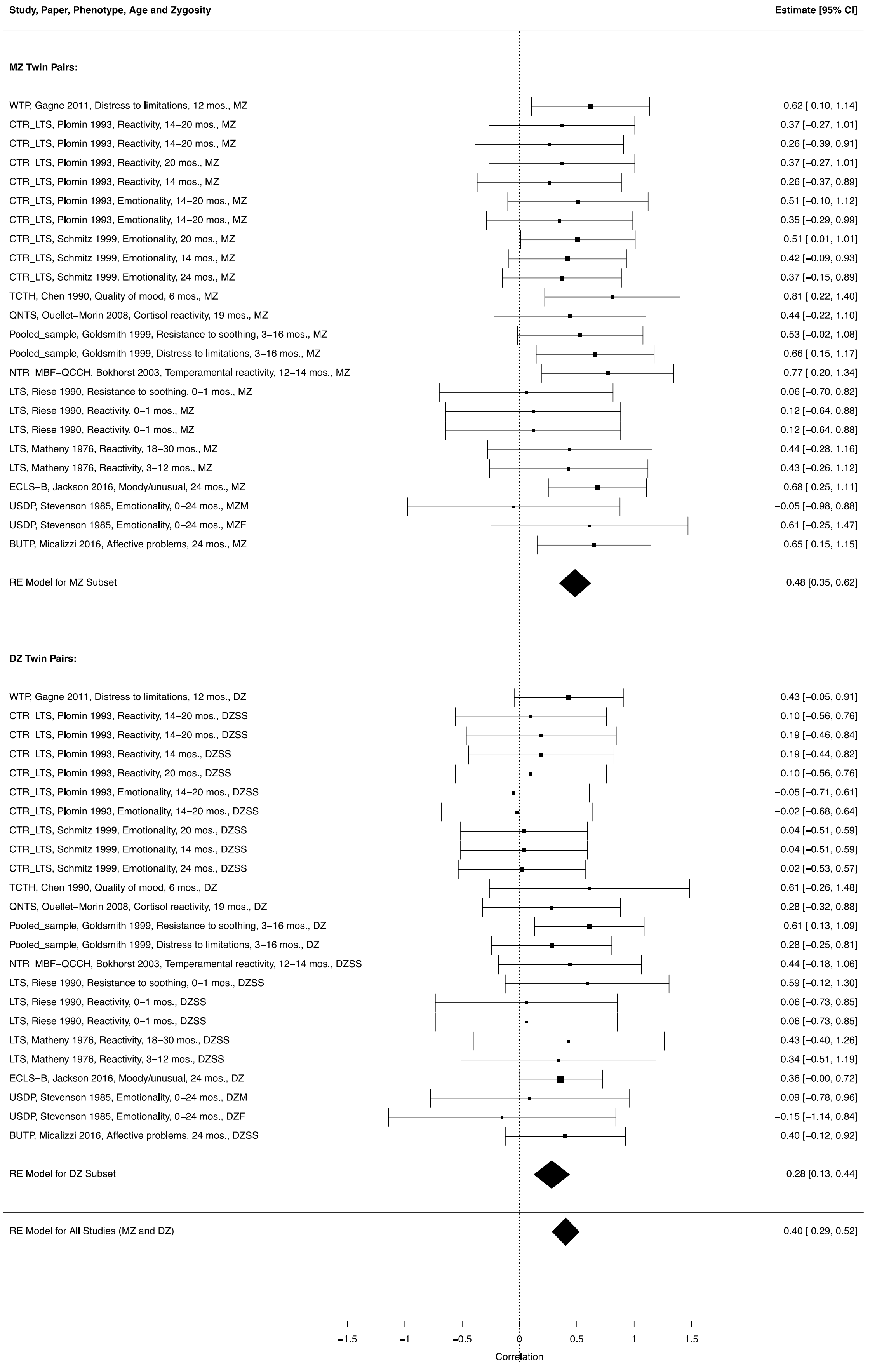

© 2022 Austerberry C et al. *JAMA Network Open.*

eFigure 24. Range of Emotion Forest Plot

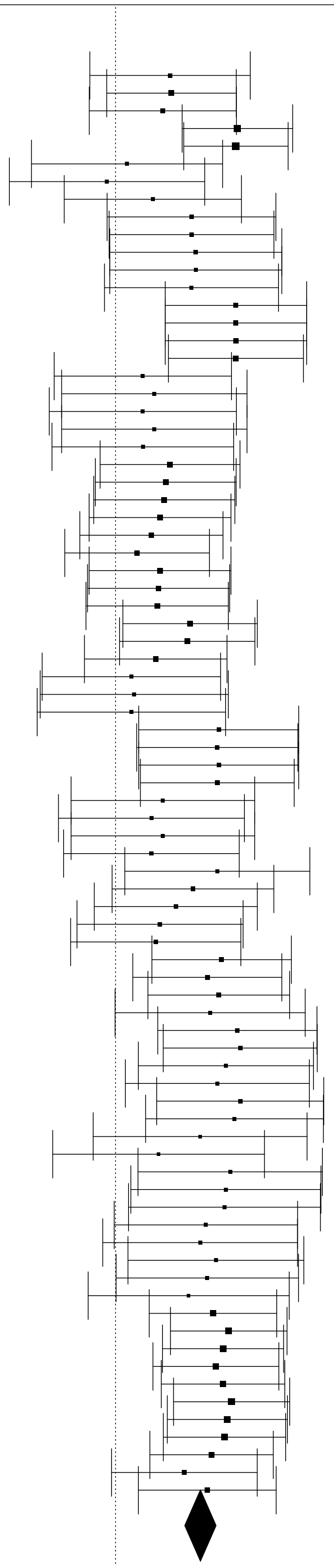

Study, Paper, Phenotype, Age and Zygosity	Estimate [95% CI]
**MZ Twin Pairs:**	
WTP, Gagne 2011, Anger, 12 mos., MZ	0.38 [−0.18, 0.94]
WC_BTTYP, Planalp 2017, Positive affect, 12 mos., MZ	0.39 [−0.06, 0.84]
WC_BTTYP, Planalp 2017, Positive affect, 6 mos., MZ	0.33 [−0.18, 0.84]
WC_BTTYP, Planalp 2017, Smiling and laughter, 6 mos., MZ	0.85 [0.46, 1.24]
WC_BTTYP, Planalp 2017, Smiling and laughter, 12 mos., MZ	0.84 [0.48, 1.20]
CTR_TIP, DiLalla 1996, Negative affect, 7 mos., MZ	0.08 [−0.59, 0.75]
CTR_TIP, DiLalla 1996, Negative affect, 9 mos., MZ	−0.06 [−0.74, 0.62]
CTR_LTS, Emde 1992, Frustration, 14 mos., MZ	0.26 [−0.36, 0.88]
CTR_LTS, Plomin 1993, Positive hedonic tone, 14 mos., MZ	0.53 [−0.06, 1.12]
CTR_LTS, Emde 1992, Positive hedonic tone, 14 mos., MZ	0.53 [−0.05, 1.11]
CTR_LTS, Plomin 1993, Positive hedonic tone, 20 mos., MZ	0.56 [−0.04, 1.16]
CTR_LTS, Plomin 1993, Positive affect, 14−20 mos., MZ	0.56 [−0.04, 1.16]
CTR_LTS, Plomin 1993, Positive affect, 14−20 mos., MZ	0.53 [−0.08, 1.14]
CTR_LTS, Plomin 1993, Positive affect, 14−20 mos., MZ	0.84 [0.35, 1.33]
CTR_LTS, Plomin 1993, Positive affect, 14−20 mos., MZ	0.84 [0.35, 1.33]
CTR_LTS, Plomin 1993, Positive affect, 20 mos., MZ	0.84 [0.35, 1.33]
CTR_LTS, Plomin 1993, Positive affect, 14 mos., MZ	0.84 [0.37, 1.31]
CTR_LTS, Emde 1992, Overall mood, 14 mos., MZ	0.19 [−0.43, 0.81]
CTR_LTS, Plomin 1993, Affect, 14−20 mos., MZ	0.27 [−0.38, 0.92]
CTR_LTS, Plomin 1993, Affect, 14−20 mos., MZ	0.19 [−0.46, 0.84]
CTR_LTS, Plomin 1993, Overall mood, 20 mos., MZ	0.27 [−0.38, 0.92]
CTR_LTS, Plomin 1993, Affect, 14 mos., MZ	0.19 [−0.44, 0.82]
CTR_LTS, Rhee 2012, Negative affect, 14 mos., MZ	0.38 [−0.11, 0.87]
CTR_LTS, Rhee 2012, Negative affect, 24 mos., MZ	0.35 [−0.14, 0.84]
CTR_LTS, Rhee 2012, Negative affect, 20 mos., MZ	0.34 [−0.15, 0.83]
CTR_LTS, Rhee 2012, Negative affect, 24 mos., MZ	0.31 [−0.19, 0.81]
CTR_LTS, Rhee 2012, Negative affect, 14 mos., MZ	0.25 [−0.25, 0.75]
CTR_LTS, Rhee 2012, Negative affect, 20 mos., MZ	0.15 [−0.35, 0.65]
CTR_LTS, Rhee 2012, Negative affect, 14 mos., MZ	0.31 [−0.19, 0.81]
CTR_LTS, Rhee 2012, Negative affect, 24 mos., MZ	0.30 [−0.20, 0.80]
CTR_LTS, Rhee 2012, Negative affect, 20 mos., MZ	0.29 [−0.21, 0.79]
CTR_LTS, Rhee 2012, Negative affect, 24 mos., MZ	0.52 [0.05, 0.99]
CTR_LTS, Rhee 2012, Negative affect, 20 mos., MZ	0.50 [0.03, 0.97]
CTR_LTS, Rhee 2012, Negative affect, 14 mos., MZ	0.28 [−0.22, 0.78]
CTR_LTS, Emde 1992, Negative hedonic tone, 14 mos., MZ	0.11 [−0.51, 0.73]
CTR_LTS, Plomin 1993, Negative affect, 14−20 mos., MZ	0.13 [−0.53, 0.79]
CTR_LTS, Plomin 1993, Negative affect, 14−20 mos., MZ	0.11 [−0.55, 0.77]
CTR_LTS, Plomin 1993, Negative affect, 14−20 mos., MZ	0.72 [0.16, 1.28]
CTR_LTS, Plomin 1993, Negative affect, 14−20 mos., MZ	0.71 [0.15, 1.27]
CTR_LTS, Plomin 1993, Negative affect, 20 mos., MZ	0.72 [0.16, 1.28]
CTR_LTS, Plomin 1993, Negative affect, 14 mos., MZ	0.71 [0.17, 1.25]
CTR_LTS, Plomin 1993, Affect, 14−20 mos., MZ	0.33 [−0.31, 0.97]
CTR_LTS, Plomin 1993, Affect, 14−20 mos., MZ	0.25 [−0.40, 0.90]
CTR_LTS, Plomin 1993, Affect, 20 mos., MZ	0.33 [−0.31, 0.97]
CTR_LTS, Emde 1992, Affect, 14 mos., MZ	0.25 [−0.36, 0.86]
TCTH, Chen 1990, Intensity of reaction, 6 mos., MZ	0.71 [0.06, 1.36]
QNTS, Schumann 2017, Negative affect, 18 mos., MZM	0.54 [−0.02, 1.10]
QNTS, Schumann 2017, Negative affect, 5 mos., MZF	0.42 [−0.15, 0.99]
QNTS, Schumann 2017, Negative affect, 18 mos., MZF	0.31 [−0.27, 0.89]
QNTS, Schumann 2017, Negative affect, 5 mos., MZM	0.28 [−0.31, 0.87]
Pooled_sample, Goldsmith 1999, Positive affect, 3−16 mos., MZ	0.74 [0.25, 1.23]
Pooled_sample, Goldsmith 1999, Negative affect, 3−16 mos., MZ	0.64 [0.12, 1.16]
Pooled_sample, Goldsmith 1999, Smiling and laughter, 3−16 mos., MZ	0.72 [0.23, 1.21]
MCTS, Stroganova 2000, Nonoriented discharges, 7−12 mos., MZ	0.66 [−0.00, 1.32]
MCTS, Stroganova 2000, Moderate tension, 7−12 mos., MZ	0.85 [0.29, 1.41]
MCTS, Stroganova 2000, Low tension, 7−12 mos., MZ	0.87 [0.33, 1.41]
MCTS, Stroganova 2000, High tension, 7−12 mos., MZ	0.77 [0.16, 1.38]
MCTS, Stroganova 2000, Fear, 7−12 mos., MZ	0.71 [0.07, 1.35]
LTS, Matheny 1989, Emotional tone, 24 mos., MZ	0.87 [0.29, 1.45]
LTS, Matheny 1989, Emotional tone, 18 mos., MZ	0.83 [0.21, 1.45]
LTS, Matheny 1989, Emotional tone, 12 mos., MZ	0.59 [−0.16, 1.34]
LTS, Riese 1990, Irritability, 0−1 mos., MZ	0.30 [−0.44, 1.04]
LTS, Matheny 1989, Fearfulness, 24 mos., MZ	0.80 [0.16, 1.44]
LTS, Matheny 1989, Fearfulness, 18 mos., MZ	0.77 [0.11, 1.43]
LTS, Matheny 1989, Fearfulness, 12 mos., MZ	0.76 [0.09, 1.43]
LTS, Matheny 1976, Tension, 3−12 mos., MZ	0.63 [−0.01, 1.27]
LTS, Matheny 1976, Tension, 18−30 mos., MZ	0.59 [−0.09, 1.27]
LTS, Matheny 1976, Fearfulness, 3−12 mos., MZ	0.70 [0.09, 1.31]
LTS, Matheny 1976, Emotional tone, 3−12 mos., MZ	0.64 [0.00, 1.28]
LTS, Matheny 1976, Emotional tone, 18−30 mos., MZ	0.51 [−0.19, 1.21]
JEP, Saudino 2008, Negative affect, 24 mos., MZM	0.68 [0.23, 1.13]
JEP, Saudino 2008, Internalizing, 24 mos., MZM	0.79 [0.38, 1.20]
JEP, Saudino 2008, General anxiety, 24 mos., MZM	0.75 [0.33, 1.17]
JEP, Saudino 2008, Depression withdrawal, 24 mos., MZM	0.70 [0.26, 1.14]
JEP, Saudino 2008, Negative affect, 24 mos., MZF	0.75 [0.32, 1.18]
JEP, Saudino 2008, Internalizing, 24 mos., MZF	0.81 [0.40, 1.22]
JEP, Saudino 2008, General anxiety, 24 mos., MZF	0.78 [0.36, 1.20]
JEP, Saudino 2008, Depression withdrawal, 24 mos., MZF	0.76 [0.33, 1.19]
ECLS−B, Jackson 2016, Demanding/angry, 24 mos., MZ	0.67 [0.24, 1.10]
ECLS−B, Roisman 2006, Fussiness and demanding behaviour, 9 mos., MZ	0.48 [−0.03, 0.99]
ECLS−B, Roisman 2006, Positive and negative affect, 9 mos., MZ	0.64 [0.16, 1.12]
RE Model for MZ Subset	0.59 [0.48, 0.70]

© 2022 Austerberry C et al. *JAMA Network Open.*

eFigure 24 (Continued)

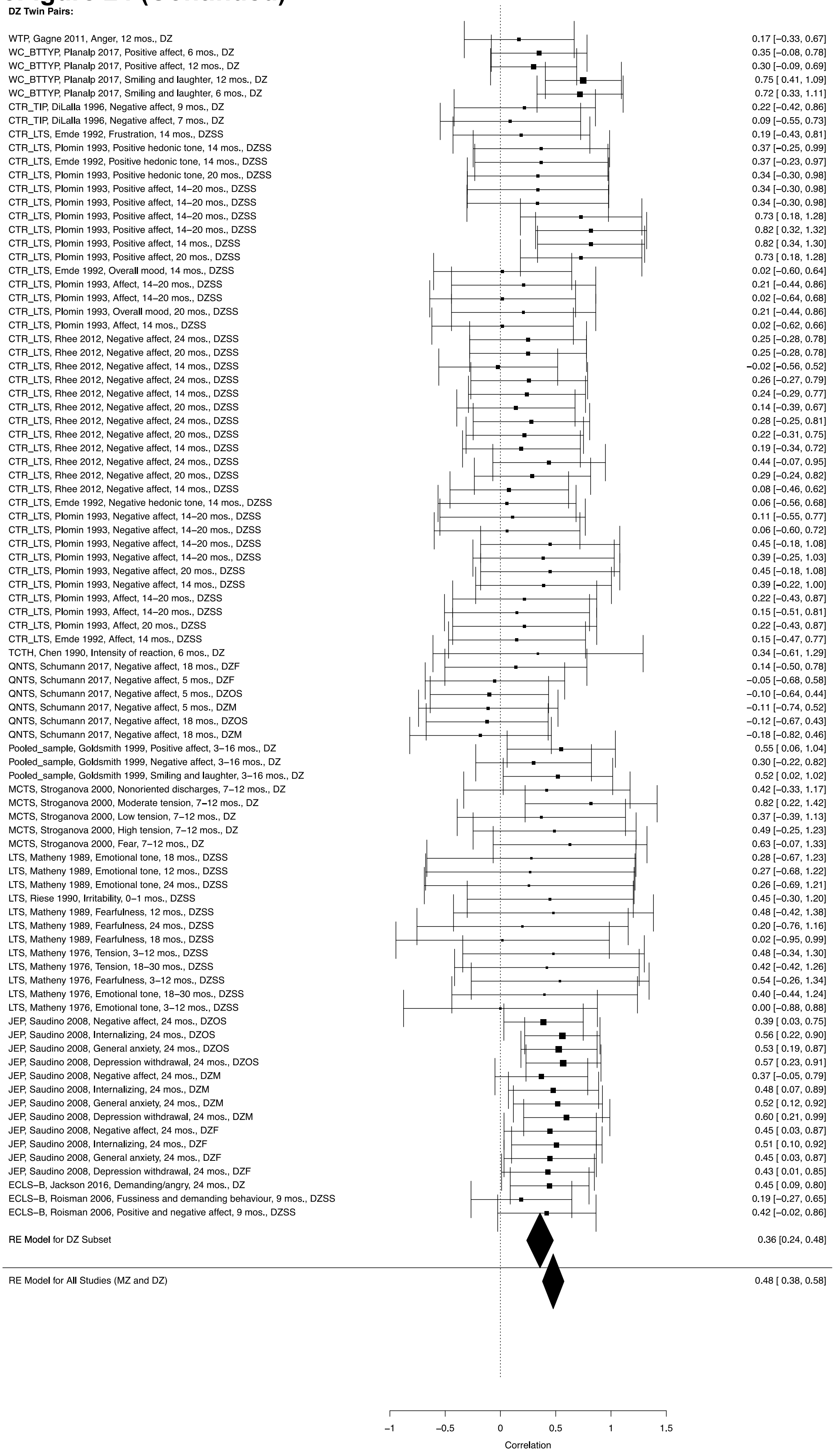

© 2022 Austerberry C et al. *JAMA Network Open.*

eFigure 25. Expression of Language Forest Plot

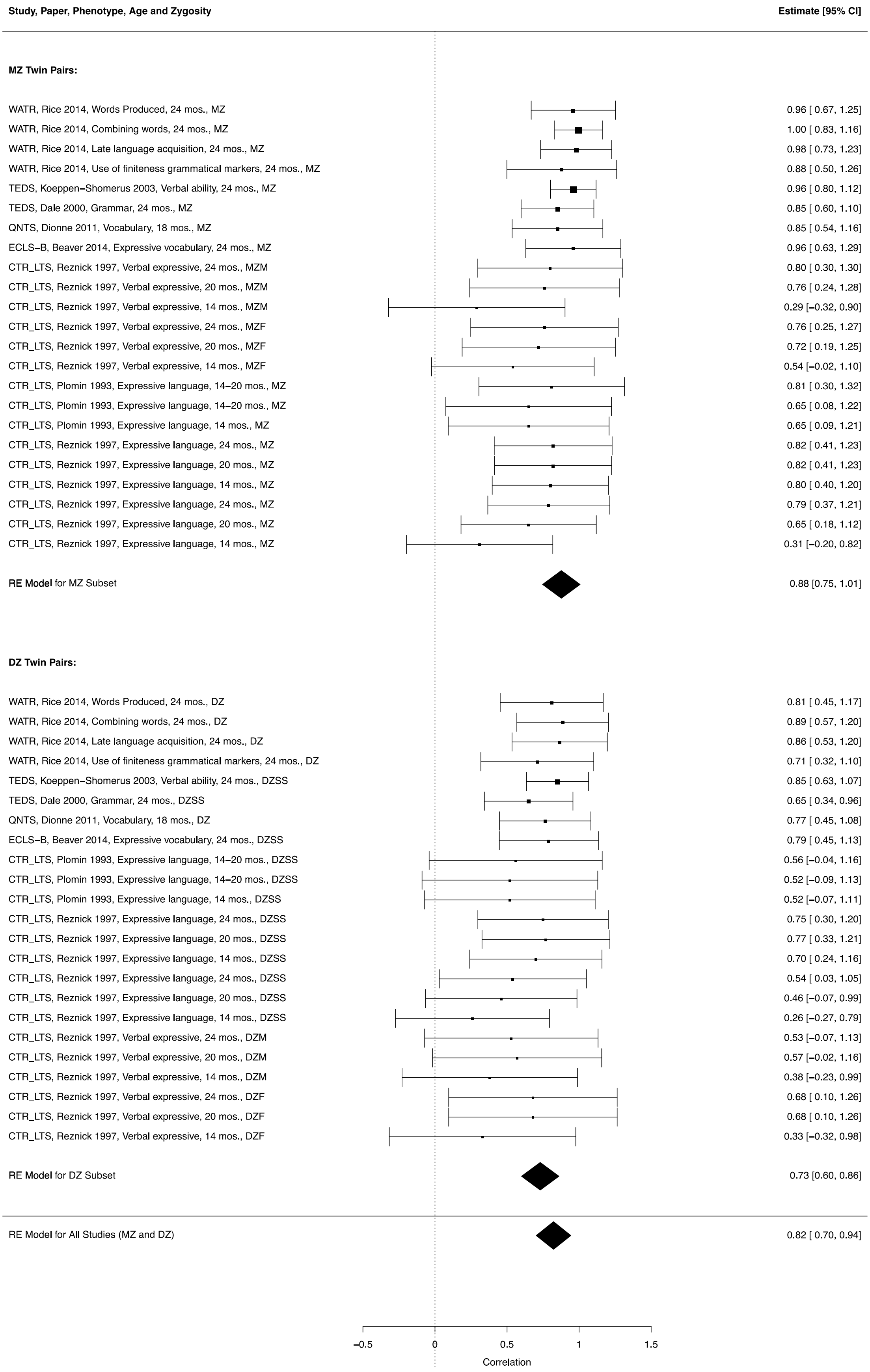

© 2022 Austerberry C et al. *JAMA Network Open.*

eFigure 26. Respect and Warmth in Relationships Forest Plot

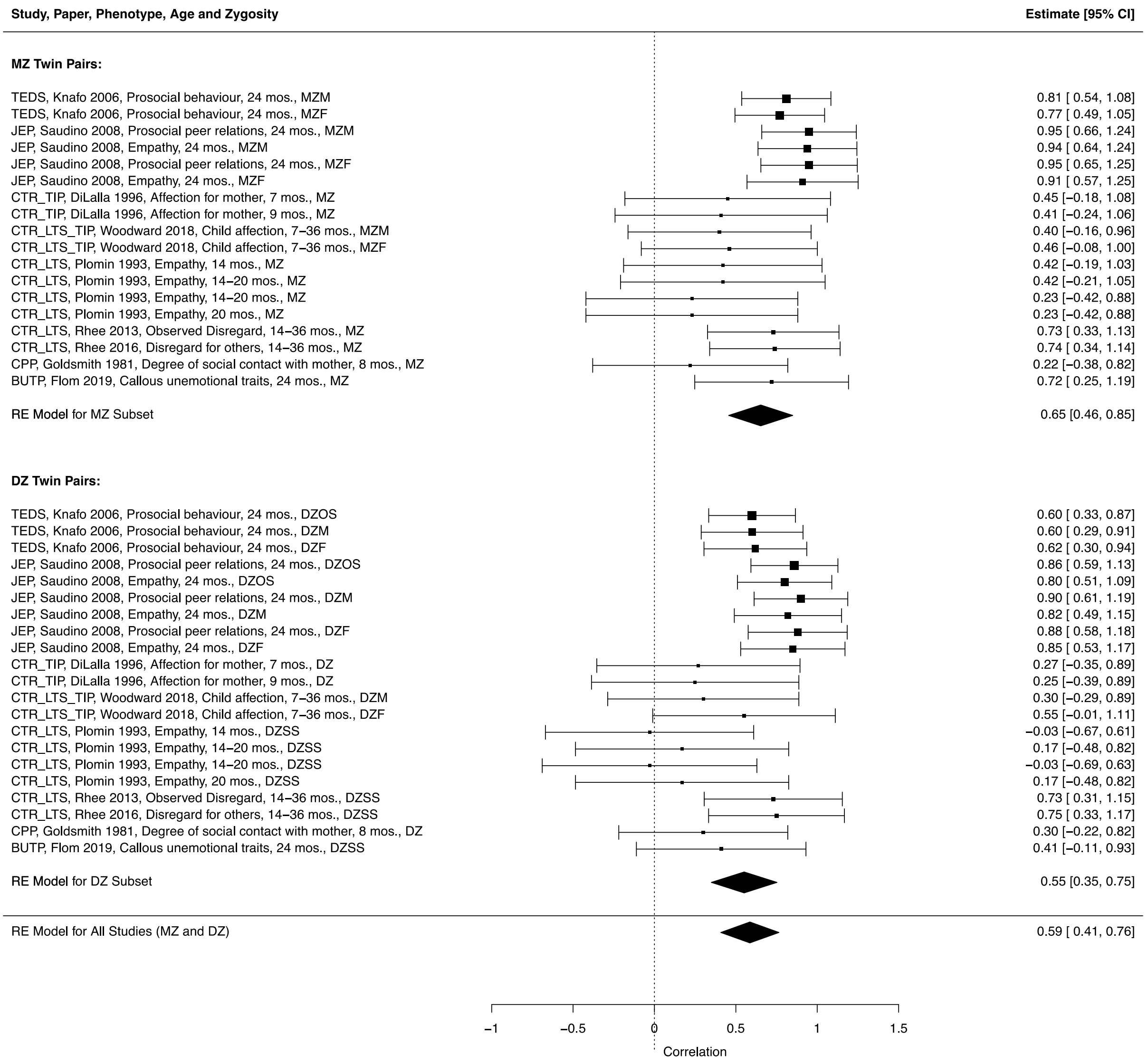

© 2022 Austerberry C et al. *JAMA Network Open.*

eFigure 27. Social Cues in Relationships Forest Plot

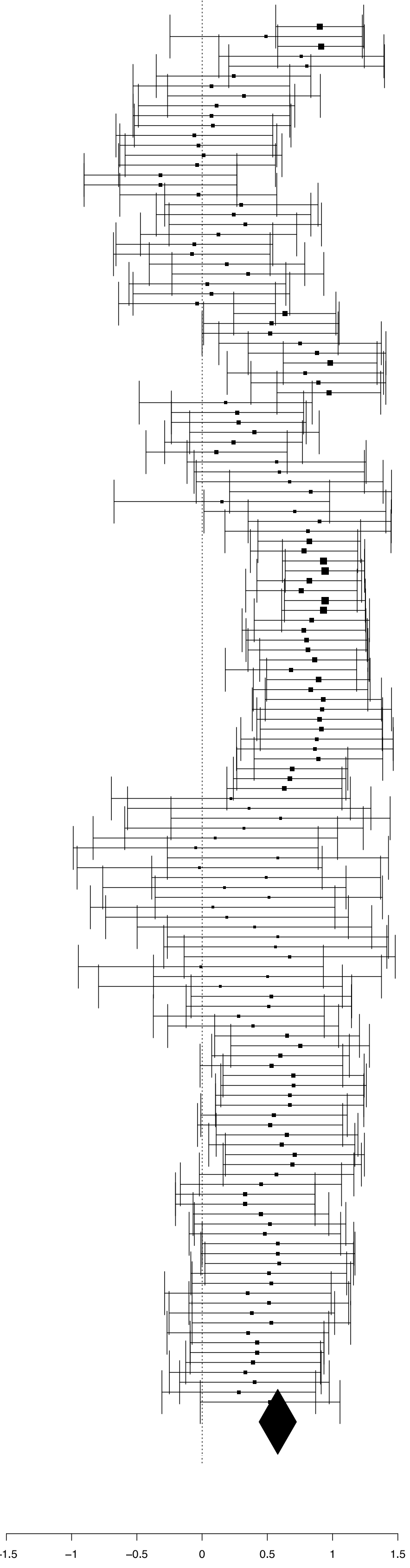

Study, Paper, Phenotype, Age and Zygosity	Estimate [95% CI]
**MZ Twin Pairs:**	
ToTCoP, Ando 2006, Mimic, point gazing, joint attention, 9–14 mos., MZM	0.90 [0.56, 1.24]
ToTCoP, Fujisawa 2012, Sociocognitive abilities, 19 mos., MZM	0.49 [−0.25, 1.23]
ToTCoP, Ando 2006, Mimic, point gazing, joint attention, 9–14 mos., MZF	0.91 [0.58, 1.24]
ToTCoP, Fujisawa 2012, Sociocognitive abilities, 19 mos., MZF	0.76 [0.13, 1.39]
TCTH, Chen 1990, Approach/withdrawal, 6 mos., MZ	0.80 [0.20, 1.40]
QNTS, Soussignan 2009, Gaze aversion, 5 mos., MZ	0.24 [−0.35, 0.83]
QNTS, Soussignan 2009, Gaze aversion, 5 mos., MZ	0.07 [−0.53, 0.67]
QNTS, Soussignan 2009, Social gaze, 5 mos., MZ	0.32 [−0.27, 0.91]
QNTS, Soussignan 2009, Social gaze, 5 mos., MZ	0.11 [−0.49, 0.71]
QNTS, Soussignan 2009, Social gaze, 5 mos., MZ	0.07 [−0.53, 0.67]
QNTS, Soussignan 2009, Social gaze, 5 mos., MZ	0.08 [−0.52, 0.68]
QNTS, Soussignan 2009, Social gaze, 5 mos., MZ	−0.06 [−0.66, 0.54]
QNTS, Soussignan 2009, Social gaze, 5 mos., MZ	−0.03 [−0.63, 0.57]
QNTS, Soussignan 2009, Social gaze, 5 mos., MZ	0.01 [−0.59, 0.61]
QNTS, Soussignan 2009, Gaze aversion, 5 mos., MZ	−0.04 [−0.64, 0.56]
QNTS, Soussignan 2009, Gaze aversion, 5 mos., MZ	−0.32 [−0.91, 0.27]
QNTS, Soussignan 2009, Emotional response to social stimuli, 5 mos., MZ	−0.32 [−0.91, 0.27]
QNTS, Soussignan 2009, Emotional response to social stimuli, 5 mos., MZ	−0.03 [−0.63, 0.57]
QNTS, Soussignan 2009, Emotional response to social stimuli, 5 mos., MZ	0.30 [−0.29, 0.89]
QNTS, Soussignan 2009, Emotional response to social stimuli, 5 mos., MZ	0.24 [−0.35, 0.83]
QNTS, Soussignan 2009, Emotional response to social stimuli, 5 mos., MZ	0.33 [−0.25, 0.91]
QNTS, Soussignan 2009, Emotional response to social stimuli, 5 mos., MZ	0.12 [−0.47, 0.72]
QNTS, Soussignan 2009, Emotional response to social stimuli, 5 mos., MZ	−0.06 [−0.66, 0.54]
QNTS, Soussignan 2009, Emotional response to social stimuli, 5 mos., MZ	−0.08 [−0.68, 0.52]
QNTS, Soussignan 2009, Gaze aversion, 5 mos., MZ	0.19 [−0.41, 0.79]
QNTS, Soussignan 2009, Gaze aversion, 5 mos., MZ	0.35 [−0.23, 0.93]
QNTS, Soussignan 2009, Gaze aversion, 5 mos., MZ	0.04 [−0.56, 0.64]
QNTS, Soussignan 2009, Gaze aversion, 5 mos., MZ	0.07 [−0.53, 0.67]
QNTS, Soussignan 2009, Gaze aversion, 5 mos., MZ	−0.04 [−0.64, 0.56]
PRINTS, Silberg 2005, Unsociability, 0–32 mos., MZ	0.63 [0.24, 1.02]
PRINTS, Silberg 2015, Sociability, 12 mos., MZ	0.53 [0.01, 1.05]
PRINTS, Silberg 2015, Inhibition, 12 mos., MZ	0.52 [−0.00, 1.04]
MCTS, Stroganova 2000, Reaction to mother's punishment , 7–12 mos., MZ	0.75 [0.13, 1.37]
MCTS, Stroganova 2000, Reaction to mother, 7–12 mos., MZ	0.88 [0.35, 1.41]
MCTS, Stroganova 2000, Reaction to father, 7–12 mos., MZ	0.98 [0.62, 1.34]
MCTS, Stroganova 2000, Orientation to humans, 7–12 mos., MZ	0.79 [0.19, 1.39]
MCTS, Stroganova 2000, Imitation of mother, 7–12 mos., MZ	0.89 [0.37, 1.41]
MCTS, Stroganova 2000, Imitation of father, 7–12 mos., MZ	0.97 [0.57, 1.37]
LTS, Matheny 1980, Affect–extraversion, 3 mos., MZ	0.18 [−0.48, 0.84]
LTS, Davis 2015, Affect–extraversion, 12 mos., MZ	0.27 [−0.24, 0.78]
LTS, Davis 2015, Affect–extraversion, 18 mos., MZ	0.28 [−0.24, 0.80]
LTS, Davis 2015, Affect–extraversion, 24 mos., MZ	0.40 [−0.10, 0.90]
LTS, Davis 2015, Affect–extraversion, 6 mos., MZ	0.24 [−0.29, 0.77]
LTS, Davis 2015, Affect–extraversion, 9 mos., MZ	0.11 [−0.43, 0.65]
LTS, Matheny 1976, Extraversion, 18–30 mos., MZ	0.57 [−0.12, 1.26]
LTS, Matheny 1976, Extraversion, 3–12 mos., MZ	0.59 [−0.06, 1.24]
LTS, Matheny 1989, Approach/withdrawal, 12 mos., MZ	0.67 [−0.05, 1.39]
LTS, Matheny 1989, Approach/withdrawal, 18 mos., MZ	0.83 [0.21, 1.45]
LTS, Matheny 1989, Approach/withdrawal, 24 mos., MZ	0.15 [−0.68, 0.98]
LTS, Matheny 1989, Behavioural inhibition, 12 mos., MZ	0.71 [0.01, 1.41]
LTS, Matheny 1989, Behavioural inhibition, 18 mos., MZ	0.90 [0.35, 1.45]
LTS, Matheny 1989, Behavioural inhibition, 24 mos., MZ	0.81 [0.17, 1.45]
JEP, Saudino 2008, Social relatedness, 24 mos., MZM	0.82 [0.43, 1.21]
JEP, Saudino 2008, Inhibition to novelty, 24 mos., MZM	0.78 [0.37, 1.19]
JEP, Saudino 2008, Imitation/Play, 24 mos., MZM	0.93 [0.61, 1.25]
JEP, Saudino 2008, Competence, 24 mos., MZM	0.94 [0.64, 1.24]
JEP, Saudino 2008, Social relatedness, 24 mos., MZF	0.82 [0.42, 1.22]
JEP, Saudino 2008, Inhibition to novelty, 24 mos., MZF	0.76 [0.33, 1.19]
JEP, Saudino 2008, Imitation/Play, 24 mos., MZF	0.94 [0.63, 1.25]
JEP, Saudino 2008, Competence, 24 mos., MZF	0.93 [0.61, 1.25]
ERSB, Marrus 2020, Social orienting, 20 mos., MZ	0.84 [0.40, 1.28]
ERSB, Marrus 2020, Social avoidance, 20 mos., MZ	0.78 [0.31, 1.25]
ERSB, Marrus 2020, Functional communication, 20 mos., MZ	0.80 [0.34, 1.26]
ERSB, Marrus 2020, Social motivation, 20 mos., MZ	0.81 [0.35, 1.27]
ERSB, Marrus 2020, Social orienting, 18 mos., MZ	0.86 [0.44, 1.28]
ERSB, Marrus 2020, Social avoidance, 18 mos., MZ	0.68 [0.18, 1.18]
ERSB, Marrus 2020, Functional communication, 18 mos., MZ	0.89 [0.49, 1.29]
ERSB, Marrus 2020, Social motivation, 18 mos., MZ	0.83 [0.39, 1.27]
ERSB, Marrus 2018, Reciprocal social behavior, 18 mos., MZ	0.93 [0.48, 1.37]
ERSB, Marrus 2015, Reciprocal social behavior, 18–24 mos., MZ	0.92 [0.38, 1.45]
ERSB, Hawks 2019, Reciprocal social behavior, 18 mos., MZ	0.90 [0.42, 1.38]
ERSB, Marrus 2018, Reciprocal social behavior, 18 mos., MZ	0.91 [0.44, 1.38]
ERSB, Marrus 2015, Reciprocal social behavior, 18–24 mos., MZ	0.88 [0.30, 1.46]
ERSB, Marrus 2015, Reciprocal social behavior, 18–24 mos., MZ	0.86 [0.26, 1.46]
ERSB, Hawks 2019, Competence, 18 mos., MZ	0.89 [0.40, 1.38]
ECLS–B, Jackson 2016, Seeks attention, 24 mos., MZ	0.69 [0.26, 1.12]
ECLS–B, Jackson 2016, Enjoys company, 24 mos., MZ	0.67 [0.24, 1.10]
ECLS–B, Jackson 2016, Avoids others/not sociable, 24 mos., MZ	0.63 [0.19, 1.07]
USDP, Stevenson 1985, Sociability, 0–24 mos., MZM	0.22 [−0.70, 1.14]
USDP, Stevenson 1985, Sociability, 0–24 mos., MSF	0.36 [−0.57, 1.29]
DTS, Plomin 1979, Smiling at mother, 22 mos., MZ	0.60 [−0.24, 1.44]
DTS, Plomin 1979, Quality of play with mother, 22 mos., MZ	0.32 [−0.59, 1.23]
DTS, Plomin 1979, Positive vocalization to mother, 22 mos., MZ	0.10 [−0.84, 1.04]
DTS, Plomin 1979, Looking at mother, 22 mos., MZ	−0.05 [−0.99, 0.89]
DTS, Plomin 1979, Smiling at stranger, 22 mos., MZ	0.58 [−0.27, 1.43]
DTS, Plomin 1979, Quality of play with stranger, 22 mos., MZ	−0.02 [−0.96, 0.92]
DTS, Plomin 1979, Positive vocalization to stranger, 22 mos., MZ	0.49 [−0.39, 1.37]
DTS, Plomin 1979, Looking at stranger, 22 mos., MZ	0.17 [−0.76, 1.10]
DTS, Plomin 1979, Latency to approach stranger, 22 mos., MZ	0.51 [−0.36, 1.38]
DTS, Plomin 1979, Smiling at stranger, 22 mos., MZ	0.08 [−0.86, 1.02]
DTS, Plomin 1979, Smiling at mother, 22 mos., MZ	0.19 [−0.74, 1.12]
DTS, Plomin 1979, Proximity to stranger, 22 mos., MZ	0.40 [−0.50, 1.30]
DTS, Plomin 1979, Positive vocalization to stranger, 22 mos., MZ	0.58 [−0.27, 1.43]
DTS, Plomin 1979, Positive vocalization to mother, 22 mos., MZ	0.56 [−0.29, 1.41]
DTS, Plomin 1979, Looking at stranger, 22 mos., MZ	0.67 [−0.14, 1.48]
DTS, Plomin 1979, Looking at mother, 22 mos., MZ	−0.01 [−0.95, 0.93]
DTS, Plomin 1979, Approaching stranger, 22 mos., MZ	0.50 [−0.37, 1.37]
DTS, Plomin 1979, Approaching mother, 22 mos., MZ	0.14 [−0.79, 1.07]
CTR_TIP, DiLalla 1996, Watch mother, 7 mos., MZ	0.53 [−0.09, 1.15]
CTR_TIP, DiLalla 1996, Watch mother, 9 mos., MZ	0.51 [−0.12, 1.14]
CTR_TIP, DiLalla 1996, Enthusiasm for interaction with mother, 7 mos., MZ	0.28 [−0.37, 0.93]
CTR_TIP, DiLalla 1996, Enthusiasm for interaction with mother, 9 mos., MZ	0.39 [−0.26, 1.04]
CTR_LTS, Cherny 1994, Shyness, 14 mos., MZ	0.65 [0.10, 1.20]
CTR_LTS, Cherny 1994, Shyness, 20 mos., MZ	0.75 [0.22, 1.28]
CTR_LTS, Cherny 1994, Shyness, 14 mos., MZ	0.60 [0.07, 1.13]
CTR_LTS, Cherny 1994, Shyness, 20 mos., MZ	0.53 [−0.02, 1.08]
CTR_LTS, Plomin 1993, Shyness, 14 mos., MZ	0.70 [0.16, 1.24]
CTR_LTS, Plomin 1993, Shyness, 14–20 mos., MZ	0.70 [0.14, 1.26]
CTR_LTS, Plomin 1993, Shyness, 14–20 mos., MZ	0.67 [0.10, 1.24]
CTR_LTS, Plomin 1993, Shyness, 20 mos., MZ	0.67 [0.10, 1.24]
CTR_LTS, Smith 2012, Behavioural inhibition, 14 mos., MZM	0.55 [−0.01, 1.11]
CTR_LTS, Smith 2012, Behavioural inhibition, 14 mos., MZF	0.52 [−0.04, 1.08]
CTR_LTS, Smith 2012, Behavioural inhibition, 20 mos., MZF	0.65 [0.11, 1.19]
CTR_LTS, Smith 2012, Behavioural inhibition, 20 mos., MZM	0.61 [0.05, 1.17]
CTR_LTS, Smith 2012, Behavioural inhibition, 24 mos., MZM	0.71 [0.18, 1.24]
CTR_LTS, Smith 2012, Behavioural inhibition, 24 mos., MZF	0.69 [0.16, 1.22]
CTR_LTS, Plomin 1993, Behavioural inhibition, 14–20 mos., MZ	0.57 [−0.02, 1.16]
CTR_LTS, Plomin 1993, Behavioural inhibition, 14–20 mos., MZ	0.45 [−0.17, 1.07]
CTR_LTS, Saudino 1996, Affect–extraversion, 14 mos., MZ	0.33 [−0.20, 0.86]
CTR_LTS, Saudino 1996, Affect–extraversion, 20 mos., MZ	0.33 [−0.20, 0.86]
CTR_LTS, Saudino 1996, Affect–extraversion, 24 mos., MZ	0.45 [−0.07, 0.97]
CTR_LTS, Smith 2012, Behavioural inhibition, 14 mos., MZM	0.52 [−0.06, 1.10]
CTR_LTS, Smith 2012, Behavioural inhibition, 14 mos., MZF	0.48 [−0.10, 1.06]
CTR_LTS, Smith 2012, Behavioural inhibition, 20 mos., MZF	0.58 [−0.00, 1.16]
CTR_LTS, Smith 2012, Behavioural inhibition, 20 mos., MZM	0.58 [−0.01, 1.17]
CTR_LTS, Smith 2012, Behavioural inhibition, 24 mos., MZF	0.59 [0.02, 1.16]
CTR_LTS, Smith 2012, Behavioural inhibition, 24 mos., MZM	0.51 [−0.09, 1.11]
CTR_LTS, Plomin 1993, Sociability, 14–20 mos., MZ	0.53 [−0.08, 1.14]
CTR_LTS, Plomin 1993, Sociability, 14–20 mos., MZ	0.35 [−0.29, 0.99]
CTR_LTS, Plomin 1993, Shyness, 14–20 mos., MZ	0.51 [−0.10, 1.12]
CTR_LTS, Plomin 1993, Shyness, 14–20 mos., MZ	0.38 [−0.25, 1.01]
CTR_LTS, Plomin 1993, Sociability, 20 mos., MZ	0.53 [−0.08, 1.14]
CTR_LTS, Plomin 1993, Sociability, 14 mos., MZ	0.35 [−0.27, 0.97]
CTR_LTS, Schmitz 1999, Shyness, 14 mos., MZ	0.42 [−0.09, 0.93]
CTR_LTS, Schmitz 1999, Shyness, 20 mos., MZ	0.42 [−0.09, 0.93]
CTR_LTS, Schmitz 1999, Shyness, 24 mos., MZ	0.39 [−0.13, 0.91]
CPP, Goldsmith 1981, Interest in persons, 8 mos., MZ	0.33 [−0.25, 0.91]
CPP, Goldsmith 1981, Degree of social acceptance of examiner, 8 mos., MZ	0.40 [−0.17, 0.97]
CPP, Goldsmith 1981, Interest in/responsiveness to people, 8 mos., MZ	0.28 [−0.31, 0.87]
BUTP, Ronald 2010, Social autistic–like traits, 24 mos., MZ	0.52 [−0.02, 1.06]
**RE Model** for MZ Subset	0.58 [0.44, 0.72]

© 2022 Austerberry C et al. *JAMA Network Open.*

eFigure 27 (Continued)

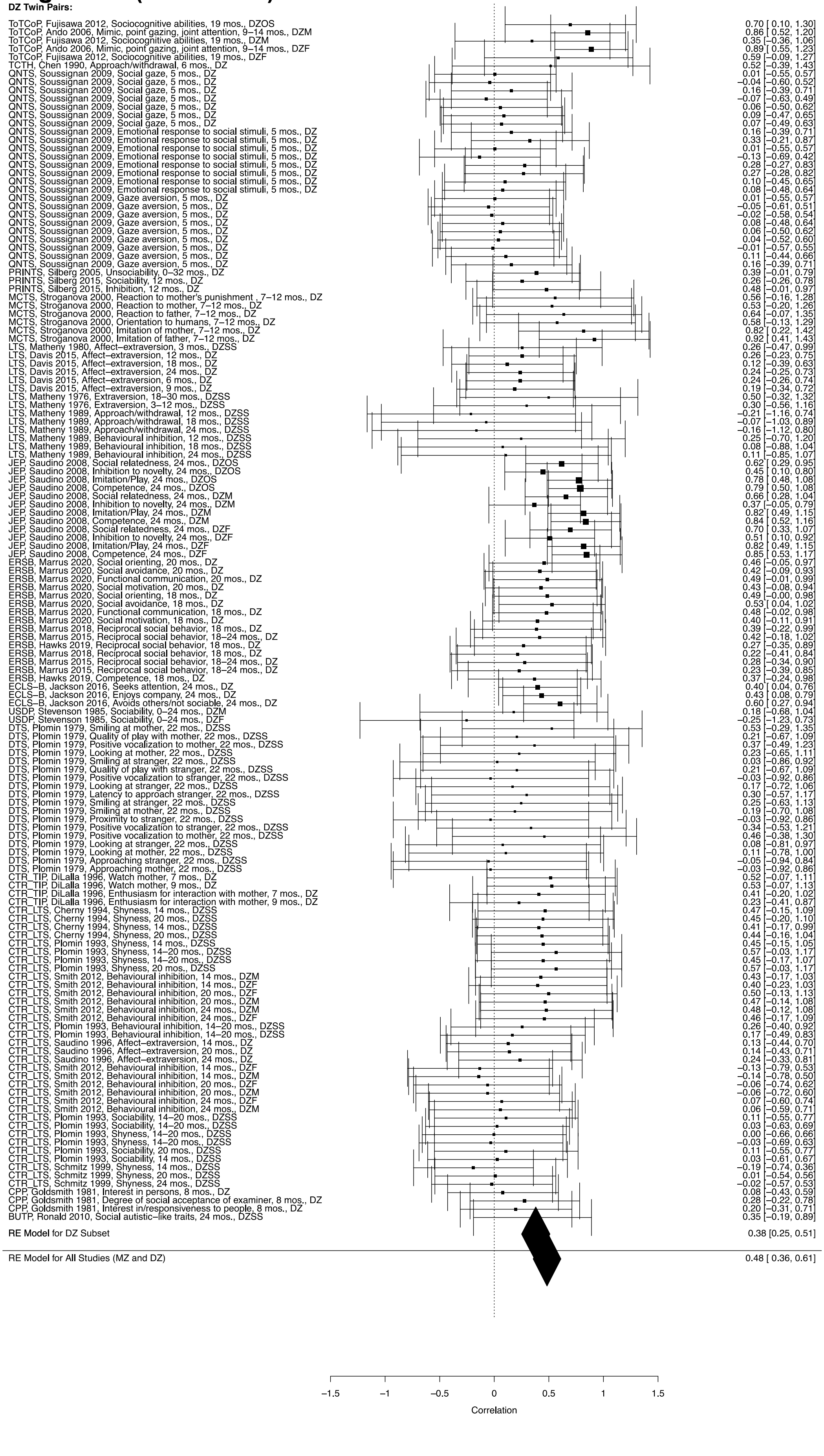

© 2022 Austerberry C et al. *JAMA Network Open.*

eFigure 28. Regulating Behaviors Within Interactions Forest Plot

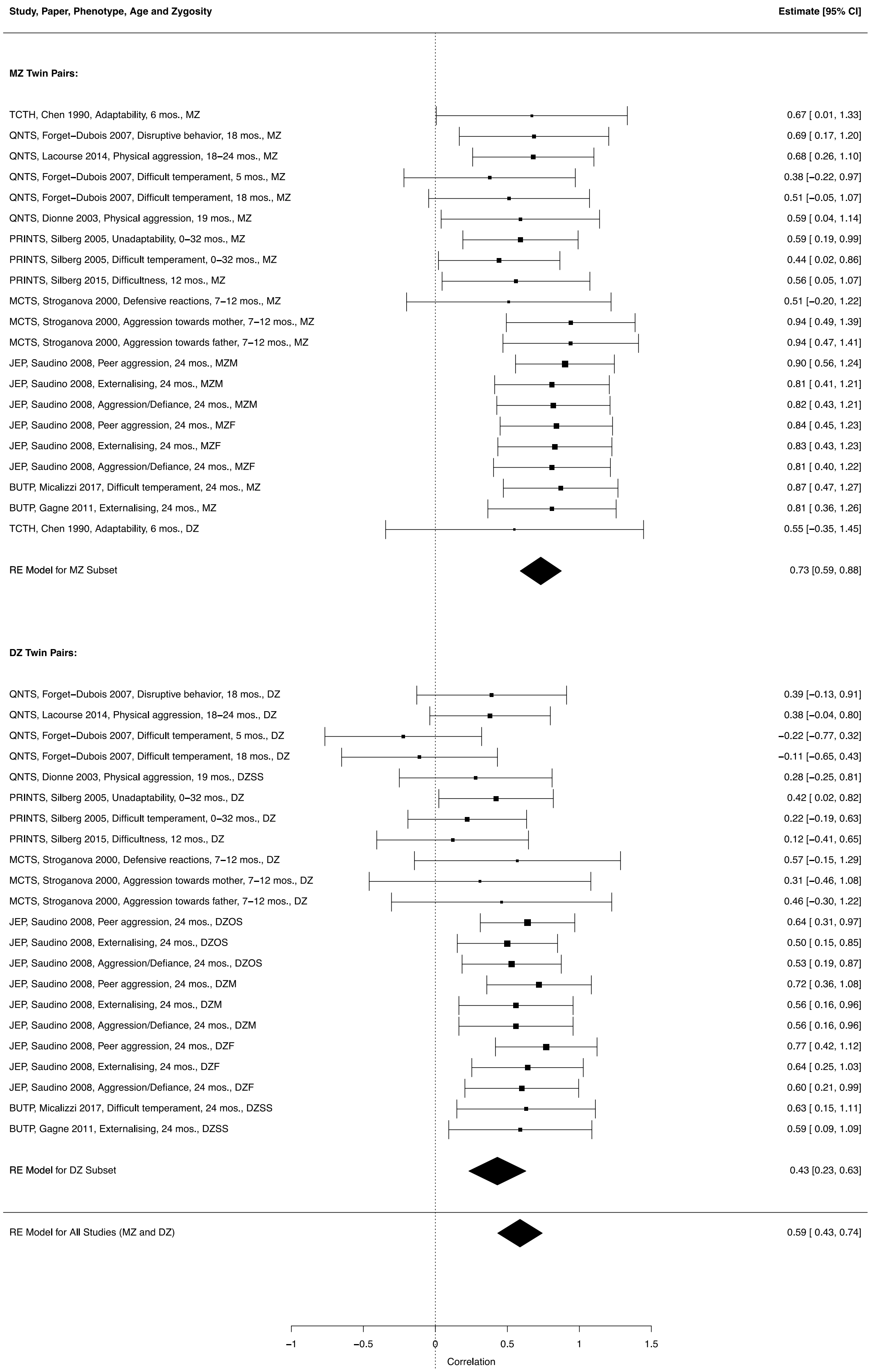

© 2022 Austerberry C et al. *JAMA Network Open.*

eFigure 29. Interacting According to Social Rules Forest Plot

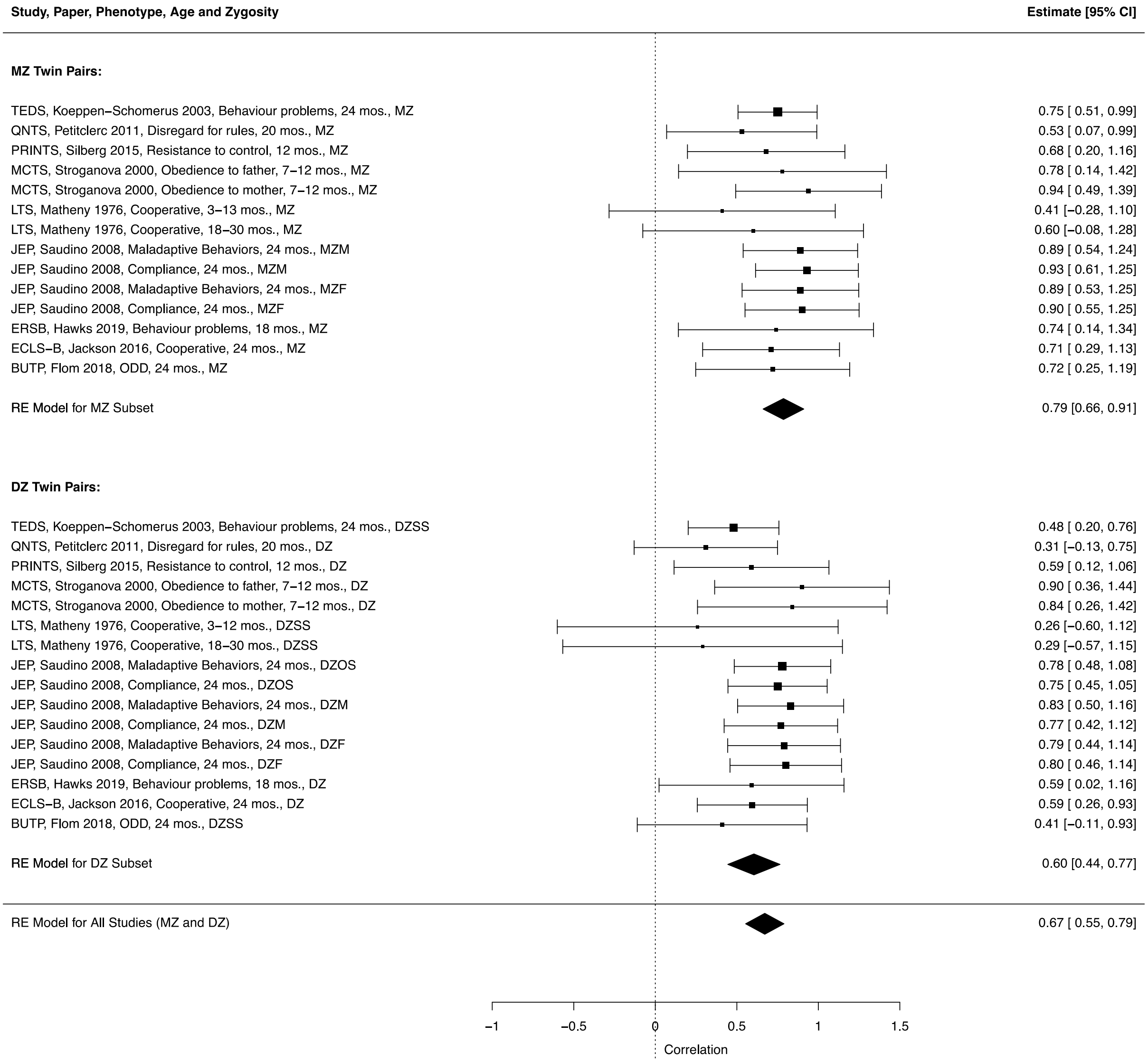

© 2022 Austerberry C et al. *JAMA Network Open.*

eFigure 30. Psychomotor Functions (Observer Report) Forest Plot

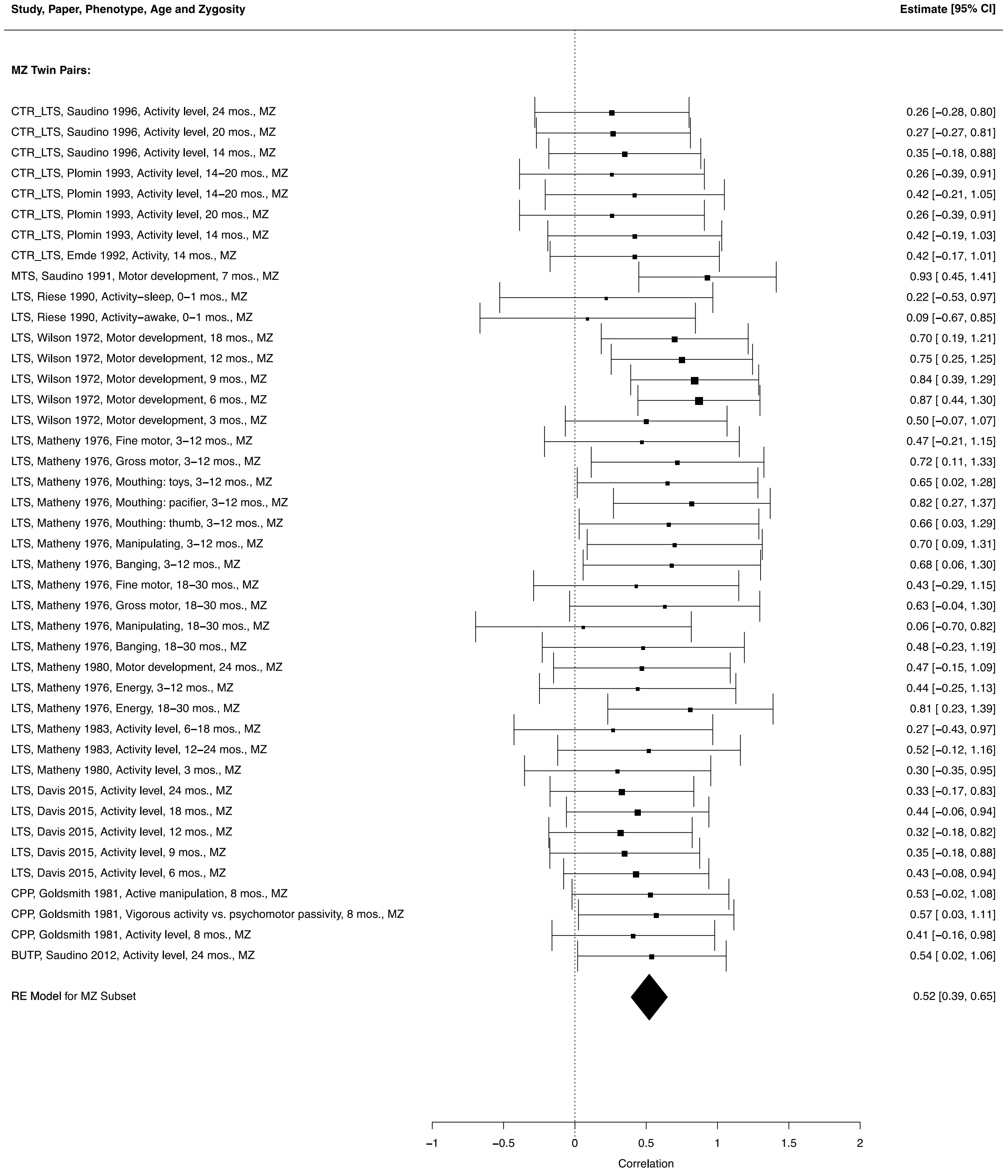

© 2022 Austerberry C et al. *JAMA Network Open.*

eFigure 30 (Continued)

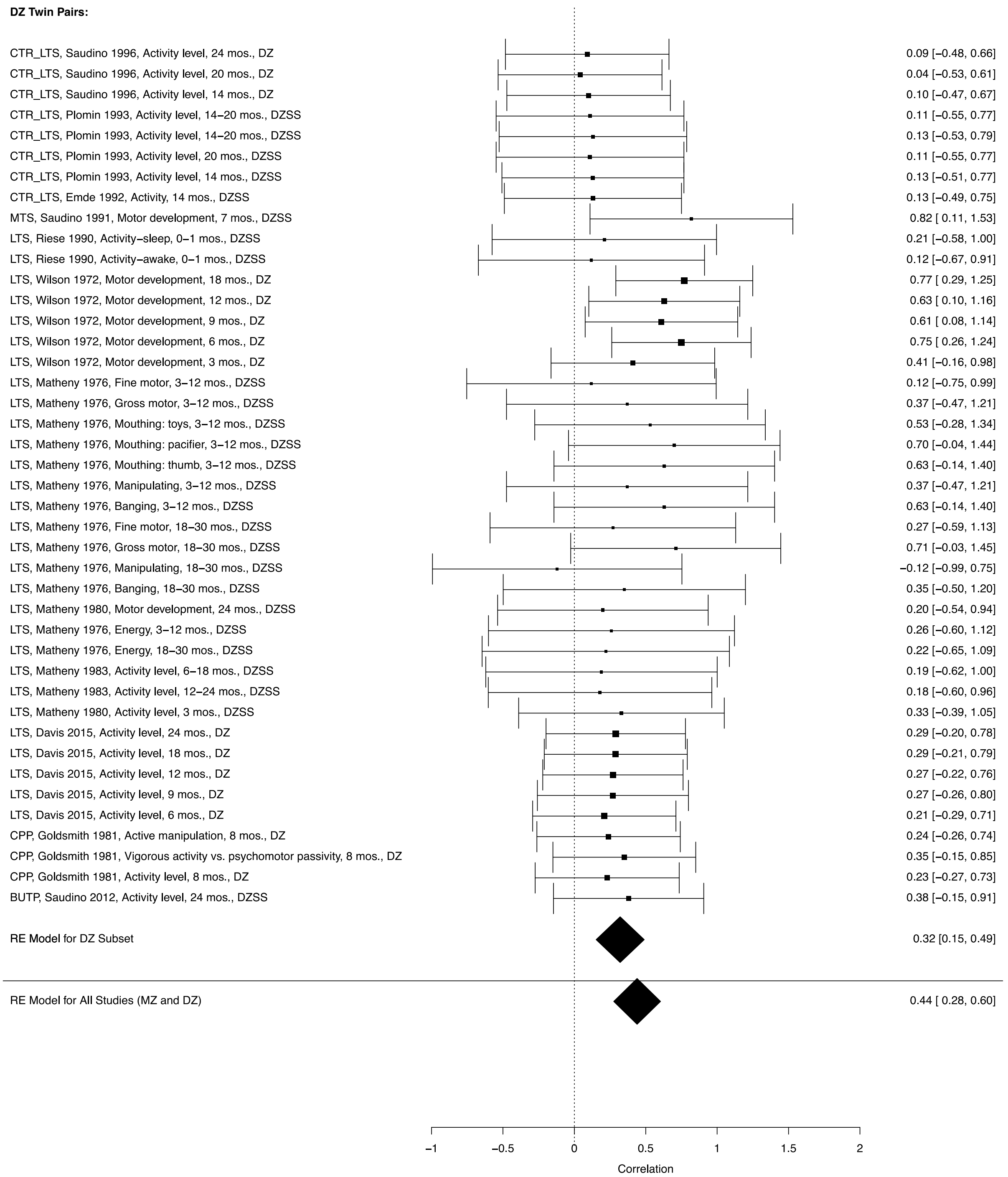

© 2022 Austerberry C et al. *JAMA Network Open.*

eFigure 31. Psychomotor Functions (Parent Report) Forest Plot

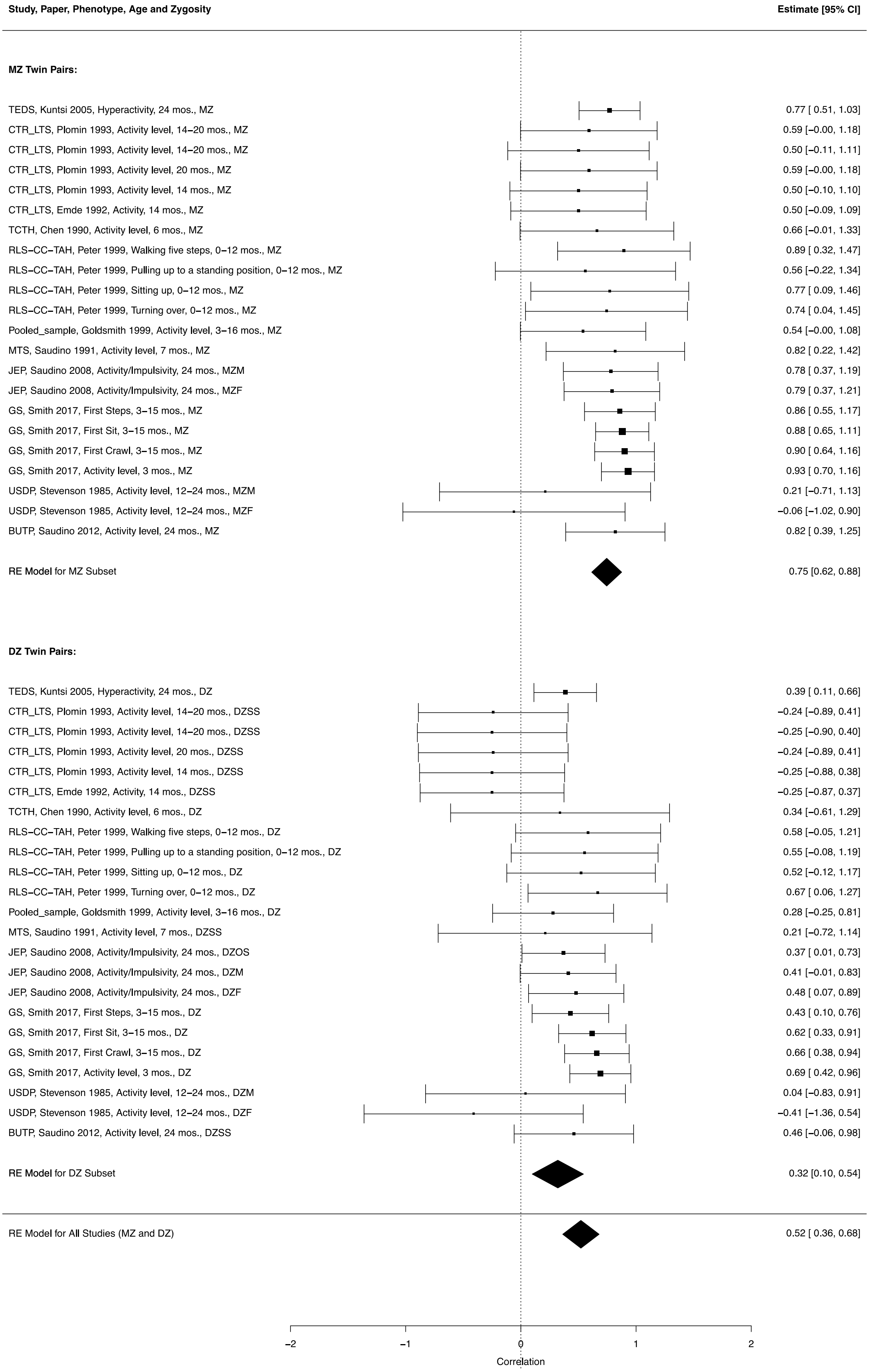

© 2022 Austerberry C et al. *JAMA Network Open.*

eFigure 32. Emotional Functions (Observer Report) Forest Plot

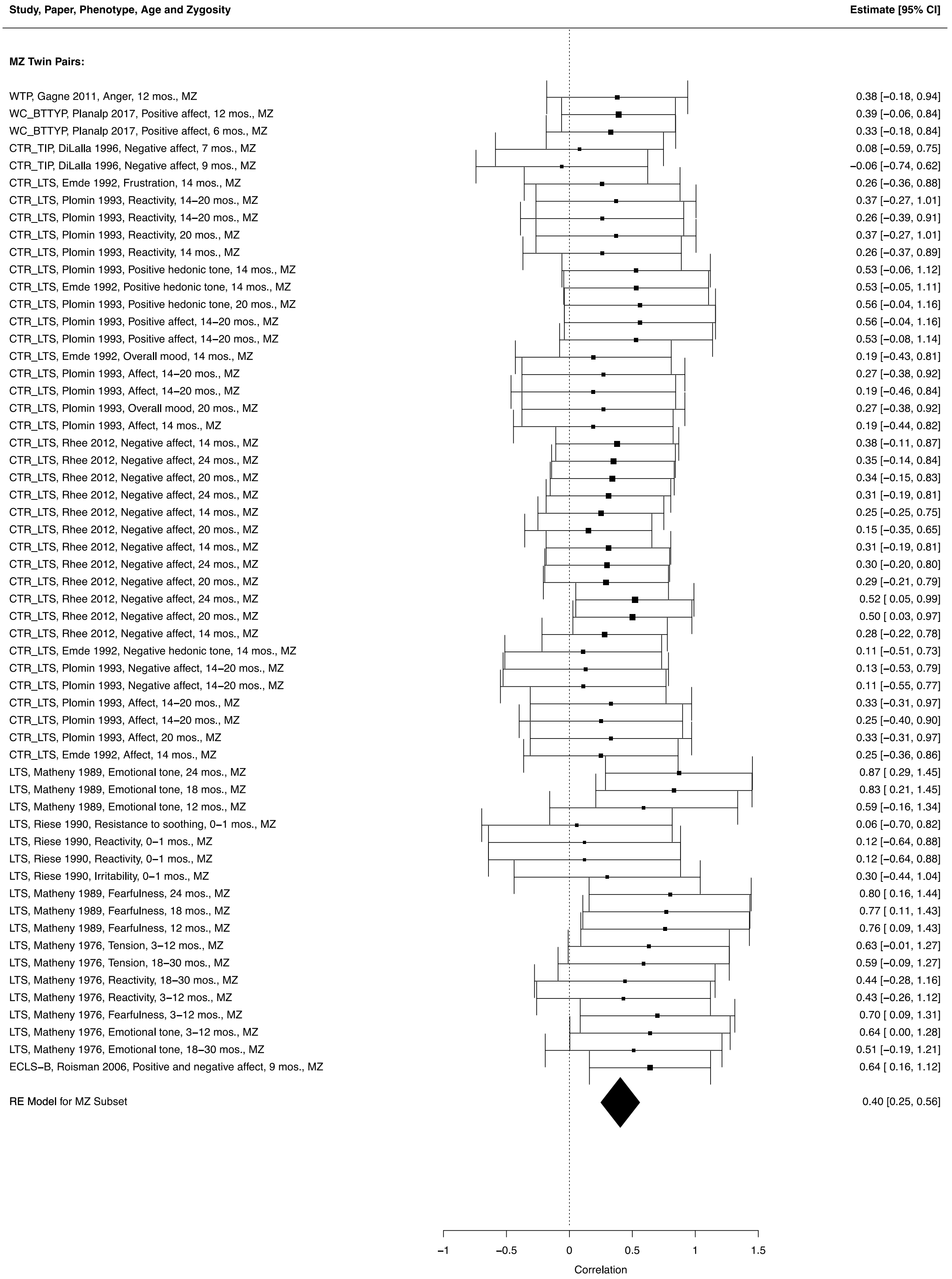

© 2022 Austerberry C et al. *JAMA Network Open.*

eFigure 32 (Continued)

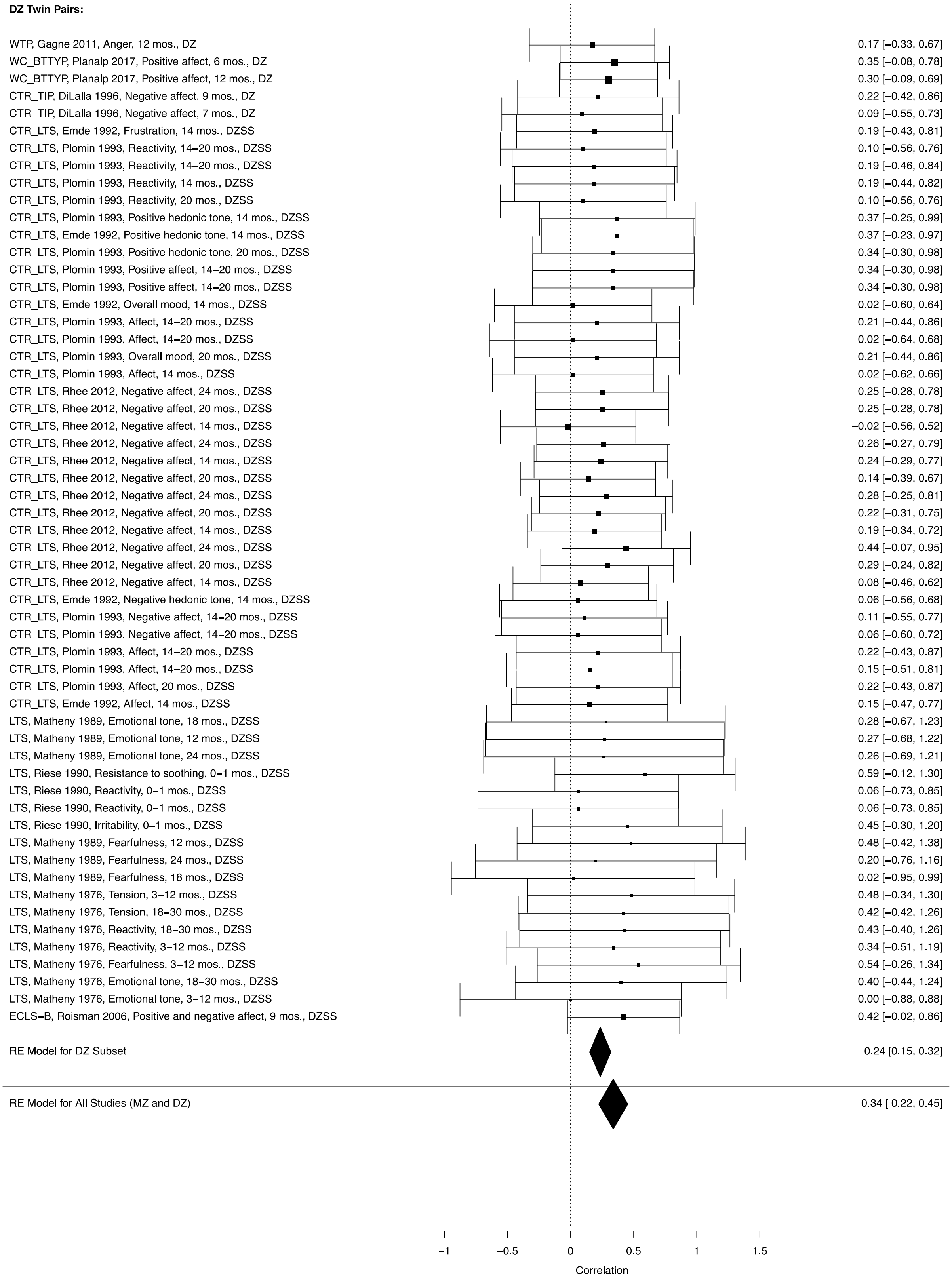

© 2022 Austerberry C et al. *JAMA Network Open.*

eFigure 33. Emotional Functions (Parent Report) Forest Plot

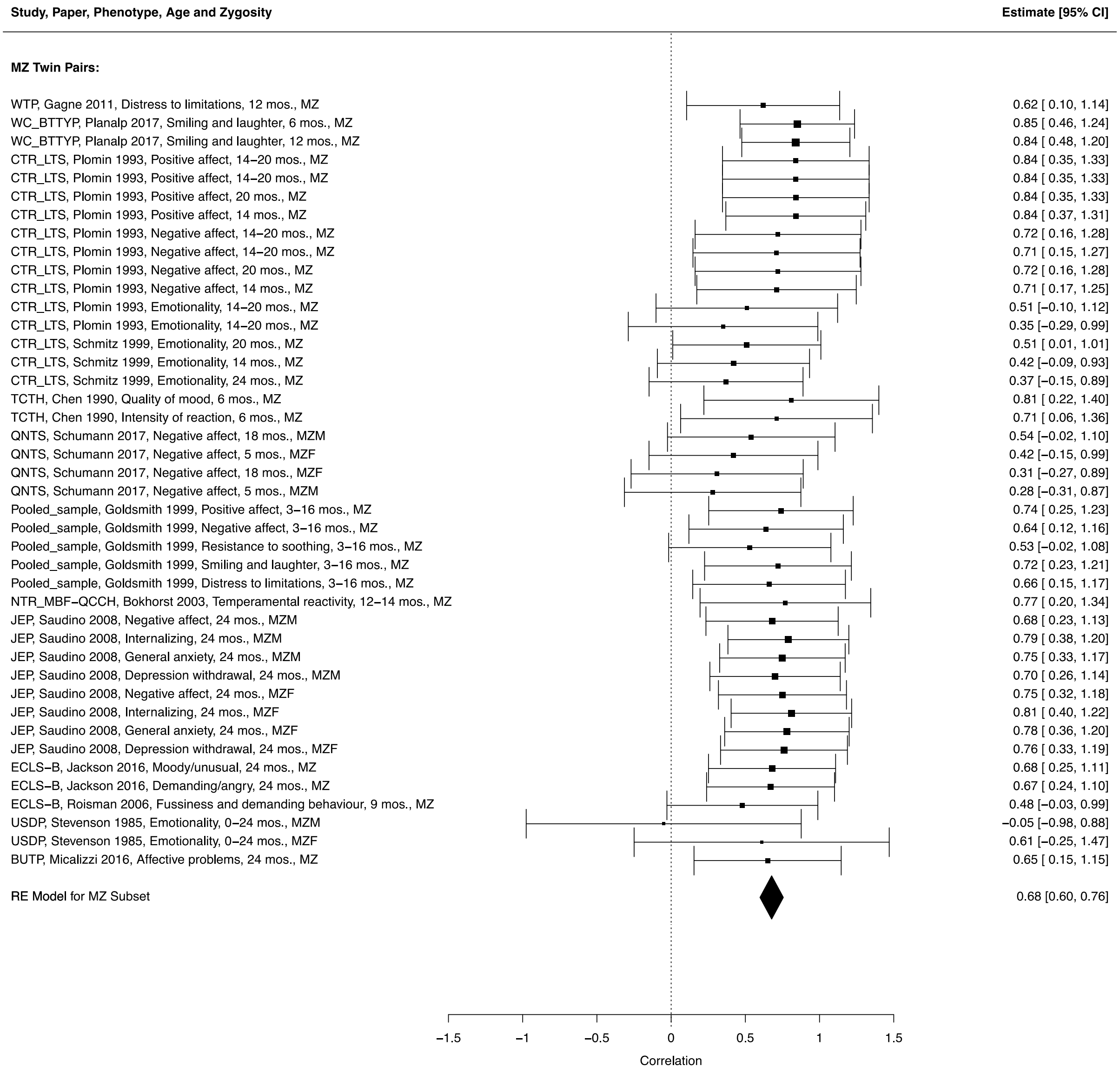

© 2022 Austerberry C et al. *JAMA Network Open.*

eFigure 33 (Continued)

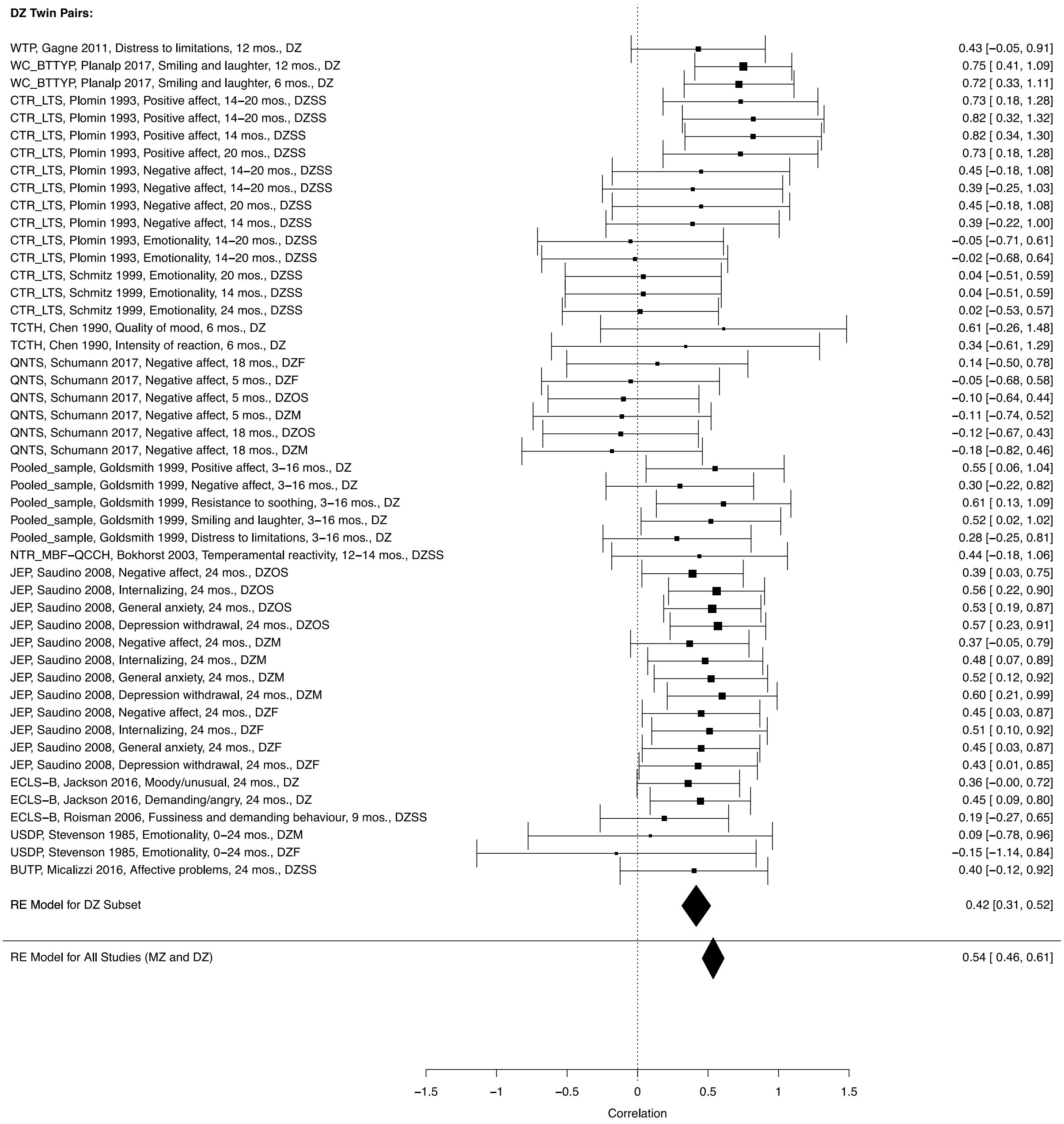

© 2022 Austerberry C et al. *JAMA Network Open.*

eFigure 34. Basic Interpersonal Interactions (Observer Report) Forest Plot

Study, Paper, Phenotype, Age and Zygosity	Estimate [95% CI]
**MZ Twin Pairs:**	
QNTS, Soussignan 2009, Gaze aversion, 5 mos., MZ	0.24 [−0.35, 0.83]
QNTS, Soussignan 2009, Gaze aversion, 5 mos., MZ	0.07 [−0.53, 0.67]
QNTS, Soussignan 2009, Social gaze, 5 mos., MZ	0.32 [−0.27, 0.91]
QNTS, Soussignan 2009, Social gaze, 5 mos., MZ	0.11 [−0.49, 0.71]
QNTS, Soussignan 2009, Social gaze, 5 mos., MZ	0.07 [−0.53, 0.67]
QNTS, Soussignan 2009, Social gaze, 5 mos., MZ	0.08 [−0.52, 0.68]
QNTS, Soussignan 2009, Social gaze, 5 mos., MZ	−0.06 [−0.66, 0.54]
QNTS, Soussignan 2009, Social gaze, 5 mos., MZ	−0.03 [−0.63, 0.57]
QNTS, Soussignan 2009, Social gaze, 5 mos., MZ	0.01 [−0.59, 0.61]
QNTS, Soussignan 2009, Gaze aversion, 5 mos., MZ	−0.04 [−0.64, 0.56]
QNTS, Soussignan 2009, Gaze aversion, 5 mos., MZ	−0.32 [−0.91, 0.27]
QNTS, Soussignan 2009, Emotional response to social stimuli, 5 mos., MZ	−0.32 [−0.91, 0.27]
QNTS, Soussignan 2009, Emotional response to social stimuli, 5 mos., MZ	−0.03 [−0.63, 0.57]
QNTS, Soussignan 2009, Emotional response to social stimuli, 5 mos., MZ	0.30 [−0.29, 0.89]
QNTS, Soussignan 2009, Emotional response to social stimuli, 5 mos., MZ	0.24 [−0.35, 0.83]
QNTS, Soussignan 2009, Emotional response to social stimuli, 5 mos., MZ	0.33 [−0.25, 0.91]
QNTS, Soussignan 2009, Emotional response to social stimuli, 5 mos., MZ	0.12 [−0.47, 0.72]
QNTS, Soussignan 2009, Emotional response to social stimuli, 5 mos., MZ	−0.06 [−0.66, 0.54]
QNTS, Soussignan 2009, Emotional response to social stimuli, 5 mos., MZ	−0.08 [−0.68, 0.52]
QNTS, Soussignan 2009, Gaze aversion, 5 mos., MZ	0.19 [−0.41, 0.79]
QNTS, Soussignan 2009, Gaze aversion, 5 mos., MZ	0.35 [−0.23, 0.93]
QNTS, Soussignan 2009, Gaze aversion, 5 mos., MZ	0.04 [−0.56, 0.64]
QNTS, Soussignan 2009, Gaze aversion, 5 mos., MZ	0.07 [−0.53, 0.67]
QNTS, Soussignan 2009, Gaze aversion, 5 mos., MZ	−0.04 [−0.64, 0.56]
LTS, Matheny 1980, Affect−extraversion, 3 mos., MZ	0.18 [−0.48, 0.84]
LTS, Davis 2015, Affect−extraversion, 12 mos., MZ	0.27 [−0.24, 0.78]
LTS, Davis 2015, Affect−extraversion, 18 mos., MZ	0.28 [−0.24, 0.80]
LTS, Davis 2015, Affect−extraversion, 24 mos., MZ	0.40 [−0.10, 0.90]
LTS, Davis 2015, Affect−extraversion, 6 mos., MZ	0.24 [−0.29, 0.77]
LTS, Davis 2015, Affect−extraversion, 9 mos., MZ	0.11 [−0.43, 0.65]
LTS, Matheny 1976, Extraversion, 18−30 mos., MZ	0.57 [−0.12, 1.26]
LTS, Matheny 1976, Extraversion, 3−12 mos., MZ	0.59 [−0.06, 1.24]
DTS, Plomin 1979, Smiling at mother, 22 mos., MZ	0.60 [−0.24, 1.44]
DTS, Plomin 1979, Quality of play with mother, 22 mos., MZ	0.32 [−0.59, 1.23]
DTS, Plomin 1979, Positive vocalization to mother, 22 mos., MZ	0.10 [−0.84, 1.04]
DTS, Plomin 1979, Looking at mother, 22 mos., MZ	−0.05 [−0.99, 0.89]
DTS, Plomin 1979, Smiling at stranger, 22 mos., MZ	0.58 [−0.27, 1.43]
DTS, Plomin 1979, Quality of play with stranger, 22 mos., MZ	−0.02 [−0.96, 0.92]
DTS, Plomin 1979, Positive vocalization to stranger, 22 mos., MZ	0.49 [−0.39, 1.37]
DTS, Plomin 1979, Looking at stranger, 22 mos., MZ	0.17 [−0.76, 1.10]
DTS, Plomin 1979, Latency to approach stranger, 22 mos., MZ	0.51 [−0.36, 1.38]
DTS, Plomin 1979, Smiling at stranger, 22 mos., MZ	0.08 [−0.86, 1.02]
DTS, Plomin 1979, Smiling at mother, 22 mos., MZ	0.19 [−0.74, 1.12]
DTS, Plomin 1979, Proximity to stranger, 22 mos., MZ	0.40 [−0.50, 1.30]
DTS, Plomin 1979, Positive vocalization to stranger, 22 mos., MZ	0.58 [−0.27, 1.43]
DTS, Plomin 1979, Positive vocalization to mother, 22 mos., MZ	0.56 [−0.29, 1.41]
DTS, Plomin 1979, Looking at stranger, 22 mos., MZ	0.67 [−0.14, 1.48]
DTS, Plomin 1979, Looking at mother, 22 mos., MZ	−0.01 [−0.95, 0.93]
DTS, Plomin 1979, Approaching stranger, 22 mos., MZ	0.50 [−0.37, 1.37]
DTS, Plomin 1979, Approaching mother, 22 mos., MZ	0.14 [−0.79, 1.07]
DTS, Plomin 1979, Cuddliness with stranger, 22 mos., MZ	0.44 [−0.45, 1.33]
DTS, Plomin 1979, Difference of response between mother and stranger − cuddliness, 22 mos., MZ	0.12 [−0.82, 1.06]
DTS, Plomin 1979, Cuddliness with mother, 22 mos., MZ	0.15 [−0.78, 1.08]
DTS, Plomin 1979, Difference of response between mother and stranger − smiling, 22 mos., MZ	0.37 [−0.53, 1.27]
DTS, Plomin 1979, Difference of response between mother and stranger − quality of play, 22 mos., MZ	0.12 [−0.82, 1.06]
DTS, Plomin 1979, Difference of response between mother and stranger − positive vocalizations, 22 mos., MZ	0.06 [−0.88, 1.00]
DTS, Plomin 1979, Difference of response between mother and stranger − looking, 22 mos., MZ	0.18 [−0.75, 1.11]
DTS, Plomin 1979, Touching stranger, 22 mos., MZ	−0.07 [−1.01, 0.87]
DTS, Plomin 1979, Touching mother, 22 mos., MZ	0.47 [−0.41, 1.35]
DTS, Plomin 1979, Difference of response between mother and stranger − touches, 22 mos., MZ	0.49 [−0.39, 1.37]
DTS, Plomin 1979, Difference of response between mother and stranger − smiling, 22 mos., MZ	0.38 [−0.52, 1.28]
DTS, Plomin 1979, Difference of response between mother and stranger − proximity, 22 mos., MZ	0.13 [−0.80, 1.06]
DTS, Plomin 1979, Difference of response between mother and stranger − positive vocalizations, 22 mos., MZ	0.58 [−0.27, 1.43]
DTS, Plomin 1979, Difference of response between mother and stranger − looking, 22 mos., MZ	0.65 [−0.17, 1.47]
DTS, Plomin 1979, Difference of response between mother and stranger − approach, 22 mos., MZ	0.20 [−0.73, 1.13]
CTR_TIP, DiLalla 1996, Watch mother, 7 mos., MZ	0.53 [−0.09, 1.15]
CTR_TIP, DiLalla 1996, Watch mother, 9 mos., MZ	0.51 [−0.12, 1.14]
CTR_TIP, DiLalla 1996, Enthusiasm for interaction with mother, 7 mos., MZ	0.28 [−0.37, 0.93]
CTR_TIP, DiLalla 1996, Enthusiasm for interaction with mother, 9 mos., MZ	0.39 [−0.26, 1.04]
CTR_TIP, DiLalla 1996, Affection for mother, 7 mos., MZ	0.45 [−0.18, 1.08]
CTR_TIP, DiLalla 1996, Affection for mother, 9 mos., MZ	0.41 [−0.24, 1.06]
CTR_LTS_TIP, Woodward 2018, Child affection, 7−36 mos., MZM	0.40 [−0.16, 0.96]
CTR_LTS_TIP, Woodward 2018, Child affection, 7−36 mos., MZF	0.46 [−0.08, 1.00]
CTR_LTS, Cherny 1994, Shyness, 14 mos., MZ	0.65 [0.10, 1.20]
CTR_LTS, Cherny 1994, Shyness, 20 mos., MZ	0.75 [0.22, 1.28]
CTR_LTS, Cherny 1994, Shyness, 14 mos., MZ	0.60 [0.07, 1.13]
CTR_LTS, Cherny 1994, Shyness, 20 mos., MZ	0.53 [−0.02, 1.08]
CTR_LTS, Plomin 1993, Shyness, 14 mos., MZ	0.70 [0.16, 1.24]
CTR_LTS, Plomin 1993, Shyness, 14−20 mos., MZ	0.70 [0.14, 1.26]
CTR_LTS, Plomin 1993, Shyness, 14−20 mos., MZ	0.67 [0.10, 1.24]
CTR_LTS, Plomin 1993, Shyness, 20 mos., MZ	0.67 [0.10, 1.24]
CTR_LTS, Smith 2012, Behavioural inhibition, 14 mos., MZM	0.55 [−0.01, 1.11]
CTR_LTS, Smith 2012, Behavioural inhibition, 14 mos., MZF	0.52 [−0.04, 1.08]
CTR_LTS, Smith 2012, Behavioural inhibition, 20 mos., MZF	0.65 [0.11, 1.19]
CTR_LTS, Smith 2012, Behavioural inhibition, 20 mos., MZM	0.61 [0.05, 1.17]
CTR_LTS, Smith 2012, Behavioural inhibition, 24 mos., MZM	0.71 [0.18, 1.24]
CTR_LTS, Smith 2012, Behavioural inhibition, 24 mos., MZF	0.69 [0.16, 1.22]
CTR_LTS, Plomin 1993, Behavioural inhibition, 14−20 mos., MZ	0.57 [−0.02, 1.16]
CTR_LTS, Plomin 1993, Behavioural inhibition, 14−20 mos., MZ	0.45 [−0.17, 1.07]
CTR_LTS, Saudino 1996, Affect−extraversion, 14 mos., MZ	0.33 [−0.20, 0.86]
CTR_LTS, Saudino 1996, Affect−extraversion, 20 mos., MZ	0.33 [−0.20, 0.86]
CTR_LTS, Saudino 1996, Affect−extraversion, 24 mos., MZ	0.45 [−0.07, 0.97]
CTR_LTS, Plomin 1993, Empathy, 14 mos., MZ	0.42 [−0.19, 1.03]
CTR_LTS, Plomin 1993, Empathy, 14−20 mos., MZ	0.42 [−0.21, 1.05]
CTR_LTS, Plomin 1993, Empathy, 14−20 mos., MZ	0.23 [−0.42, 0.88]
CTR_LTS, Plomin 1993, Empathy, 20 mos., MZ	0.23 [−0.42, 0.88]
CTR_LTS, Rhee 2013, Observed Disregard, 14−36 mos., MZ	0.73 [0.33, 1.13]
CPP, Goldsmith 1981, Interest in persons, 8 mos., MZ	0.33 [−0.25, 0.91]
CPP, Goldsmith 1981, Degree of social acceptance of examiner, 8 mos., MZ	0.40 [−0.17, 0.97]
CPP, Goldsmith 1981, Interest in/responsiveness to people, 8 mos., MZ	0.28 [−0.31, 0.87]
CPP, Goldsmith 1981, Degree of social contact with mother, 8 mos., MZ	0.22 [−0.38, 0.82]
RE Model for MZ Subset	0.34 [0.20, 0.48]

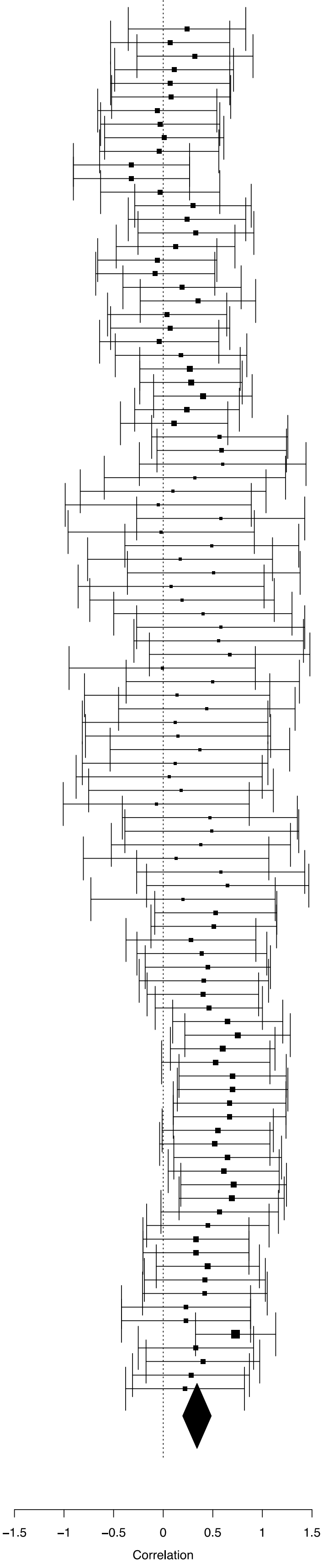

© 2022 Austerberry C et al. *JAMA Network Open.*

eFigure 34 (Continued)

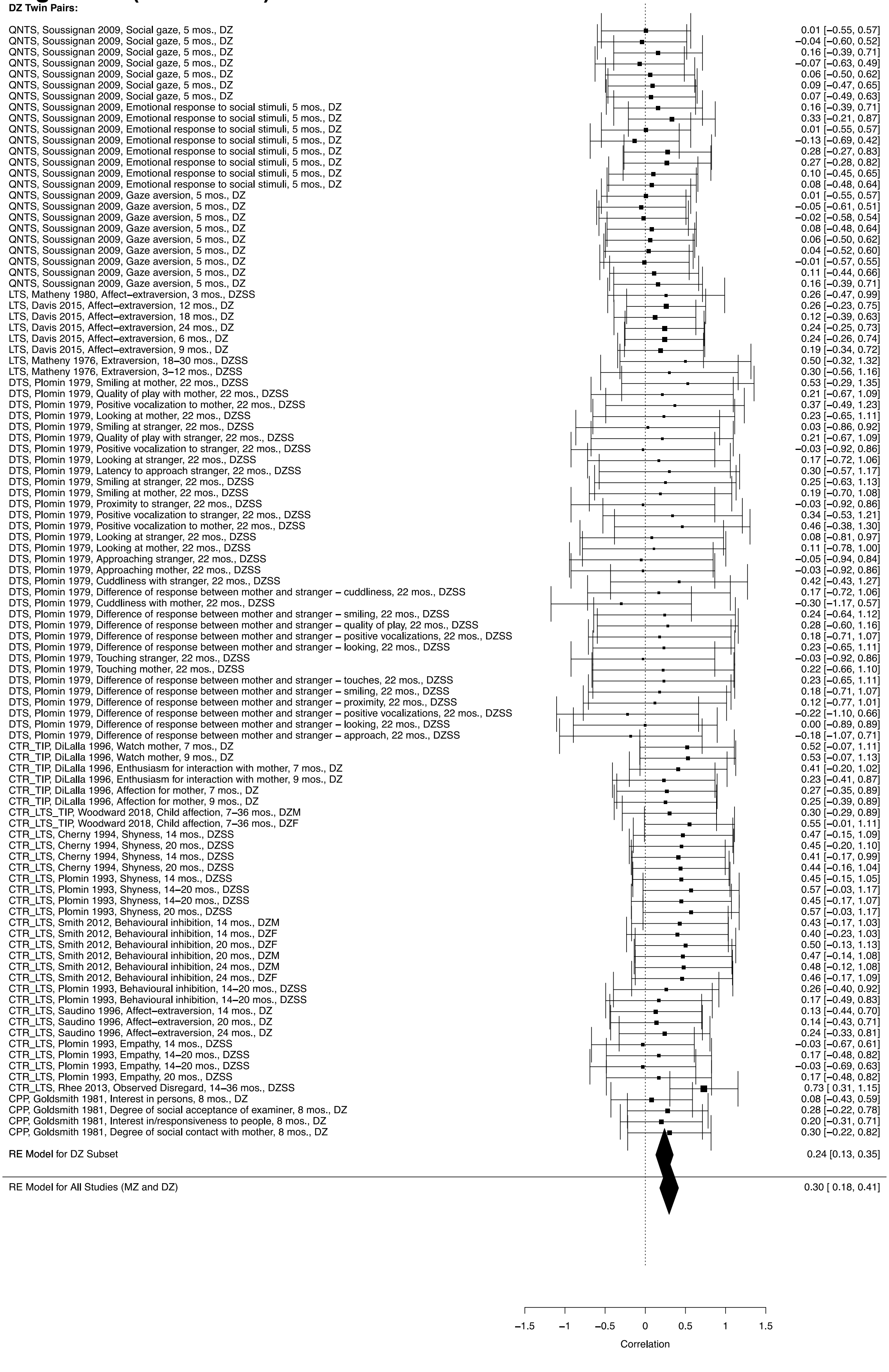

© 2022 Austerberry C et al. *JAMA Network Open.*

eFigure 35. Basic Interpersonal Interactions (Parent Report) Forest Plot

Study, Paper, Phenotype, Age and Zygosity	Estimate [95% CI]
**MZ Twin Pairs:**	
TEDS, Knafo 2006, Prosocial behaviour, 24 mos., MZM	0.81 [0.54, 1.08]
TEDS, Knafo 2006, Prosocial behaviour, 24 mos., MZF	0.77 [0.49, 1.05]
ToTCoP, Ando 2006, Mimic, point gazing, joint attention, 9–14 mos., MZM	0.90 [0.56, 1.24]
ToTCoP, Fujisawa 2012, Sociocognitive abilities, 19 mos., MZM	0.49 [−0.25, 1.23]
ToTCoP, Ando 2006, Mimic, point gazing, joint attention, 9–14 mos., MZF	0.91 [0.58, 1.24]
ToTCoP, Fujisawa 2012, Sociocognitive abilities, 19 mos., MZF	0.76 [0.13, 1.39]
TCTH, Chen 1990, Approach/withdrawal, 6 mos., MZ	0.80 [0.20, 1.40]
PRINTS, Silberg 2005, Unsociability, 0–32 mos., MZ	0.63 [0.24, 1.02]
PRINTS, Silberg 2015, Sociability, 12 mos., MZ	0.53 [0.01, 1.05]
PRINTS, Silberg 2015, Inhibition, 12 mos., MZ	0.52 [−0.00, 1.04]
LTS, Matheny 1989, Approach/withdrawal, 12 mos., MZ	0.67 [−0.05, 1.39]
LTS, Matheny 1989, Approach/withdrawal, 18 mos., MZ	0.83 [0.21, 1.45]
LTS, Matheny 1989, Approach/withdrawal, 24 mos., MZ	0.15 [−0.68, 0.98]
JEP, Saudino 2008, Social relatedness, 24 mos., MZM	0.82 [0.43, 1.21]
JEP, Saudino 2008, Prosocial peer relations, 24 mos., MZM	0.95 [0.66, 1.24]
JEP, Saudino 2008, Inhibition to novelty, 24 mos., MZM	0.78 [0.37, 1.19]
JEP, Saudino 2008, Imitation/Play, 24 mos., MZM	0.93 [0.61, 1.25]
JEP, Saudino 2008, Empathy, 24 mos., MZM	0.94 [0.64, 1.24]
JEP, Saudino 2008, Competence, 24 mos., MZM	0.94 [0.64, 1.24]
JEP, Saudino 2008, Social relatedness, 24 mos., MZF	0.82 [0.42, 1.22]
JEP, Saudino 2008, Prosocial peer relations, 24 mos., MZF	0.95 [0.65, 1.25]
JEP, Saudino 2008, Inhibition to novelty, 24 mos., MZF	0.76 [0.33, 1.19]
JEP, Saudino 2008, Imitation/Play, 24 mos., MZF	0.94 [0.63, 1.25]
JEP, Saudino 2008, Empathy, 24 mos., MZF	0.91 [0.57, 1.25]
JEP, Saudino 2008, Competence, 24 mos., MZF	0.93 [0.61, 1.25]
ERSB, Marrus 2020, Social orienting, 20 mos., MZ	0.84 [0.40, 1.28]
ERSB, Marrus 2020, Social avoidance, 20 mos., MZ	0.78 [0.31, 1.25]
ERSB, Marrus 2020, Functional communication, 20 mos., MZ	0.80 [0.34, 1.26]
ERSB, Marrus 2020, Social motivation, 20 mos., MZ	0.81 [0.35, 1.27]
ERSB, Marrus 2020, Social orienting, 18 mos., MZ	0.86 [0.44, 1.28]
ERSB, Marrus 2020, Social avoidance, 18 mos., MZ	0.68 [0.18, 1.18]
ERSB, Marrus 2020, Functional communication, 18 mos., MZ	0.89 [0.49, 1.29]
ERSB, Marrus 2020, Social motivation, 18 mos., MZ	0.83 [0.39, 1.27]
ERSB, Marrus 2018, Reciprocal social behavior, 18 mos., MZ	0.93 [0.48, 1.37]
ERSB, Marrus 2015, Reciprocal social behavior, 18–24 mos., MZ	0.92 [0.38, 1.45]
ERSB, Hawks 2019, Reciprocal social behavior, 18 mos., MZ	0.90 [0.42, 1.38]
ERSB, Marrus 2018, Reciprocal social behavior, 18 mos., MZ	0.91 [0.44, 1.38]
ERSB, Marrus 2015, Reciprocal social behavior, 18–24 mos., MZ	0.88 [0.30, 1.46]
ERSB, Marrus 2015, Reciprocal social behavior, 18–24 mos., MZ	0.86 [0.26, 1.46]
ERSB, Hawks 2019, Competence, 18 mos., MZ	0.89 [0.40, 1.38]
ECLS–B, Jackson 2016, Seeks attention, 24 mos., MZ	0.69 [0.26, 1.12]
ECLS–B, Jackson 2016, Enjoys company, 24 mos., MZ	0.67 [0.24, 1.10]
ECLS–B, Jackson 2016, Comfortable cuddly, 24 mos., MZ	0.64 [0.20, 1.08]
ECLS–B, Jackson 2016, Avoids others/not sociable, 24 mos., MZ	0.63 [0.19, 1.07]
USDP, Stevenson 1985, Sociability, 0–24 mos., MZM	0.22 [−0.70, 1.14]
USDP, Stevenson 1985, Sociability, 0–24 mos., MZF	0.36 [−0.57, 1.29]
CTR_LTS, Smith 2012, Behavioural inhibition, 14 mos., MZM	0.52 [−0.06, 1.10]
CTR_LTS, Smith 2012, Behavioural inhibition, 14 mos., MZF	0.48 [−0.10, 1.06]
CTR_LTS, Smith 2012, Behavioural inhibition, 20 mos., MZF	0.58 [−0.00, 1.16]
CTR_LTS, Smith 2012, Behavioural inhibition, 20 mos., MZM	0.58 [−0.01, 1.17]
CTR_LTS, Smith 2012, Behavioural inhibition, 24 mos., MZF	0.59 [0.02, 1.16]
CTR_LTS, Smith 2012, Behavioural inhibition, 24 mos., MZM	0.51 [−0.09, 1.11]
CTR_LTS, Plomin 1993, Sociability, 14–20 mos., MZ	0.53 [−0.08, 1.14]
CTR_LTS, Plomin 1993, Sociability, 14–20 mos., MZ	0.35 [−0.29, 0.99]
CTR_LTS, Plomin 1993, Shyness, 14–20 mos., MZ	0.51 [−0.10, 1.12]
CTR_LTS, Plomin 1993, Shyness, 14–20 mos., MZ	0.38 [−0.25, 1.01]
CTR_LTS, Plomin 1993, Sociability, 20 mos., MZ	0.53 [−0.08, 1.14]
CTR_LTS, Plomin 1993, Sociability, 14 mos., MZ	0.35 [−0.27, 0.97]
CTR_LTS, Schmitz 1999, Shyness, 14 mos., MZ	0.42 [−0.09, 0.93]
CTR_LTS, Schmitz 1999, Shyness, 20 mos., MZ	0.42 [−0.09, 0.93]
CTR_LTS, Schmitz 1999, Shyness, 24 mos., MZ	0.39 [−0.13, 0.91]
CTR_LTS, Rhee 2016, Disregard for others, 14–36 mos., MZ	0.74 [0.34, 1.14]
BUTP, Flom 2019, Callous unemotional traits, 24 mos., MZ	0.72 [0.25, 1.19]
BUTP, Ronald 2010, Social autistic–like traits, 24 mos., MZ	0.52 [−0.02, 1.06]
RE Model for MZ Subset	0.72 [0.62, 0.83]

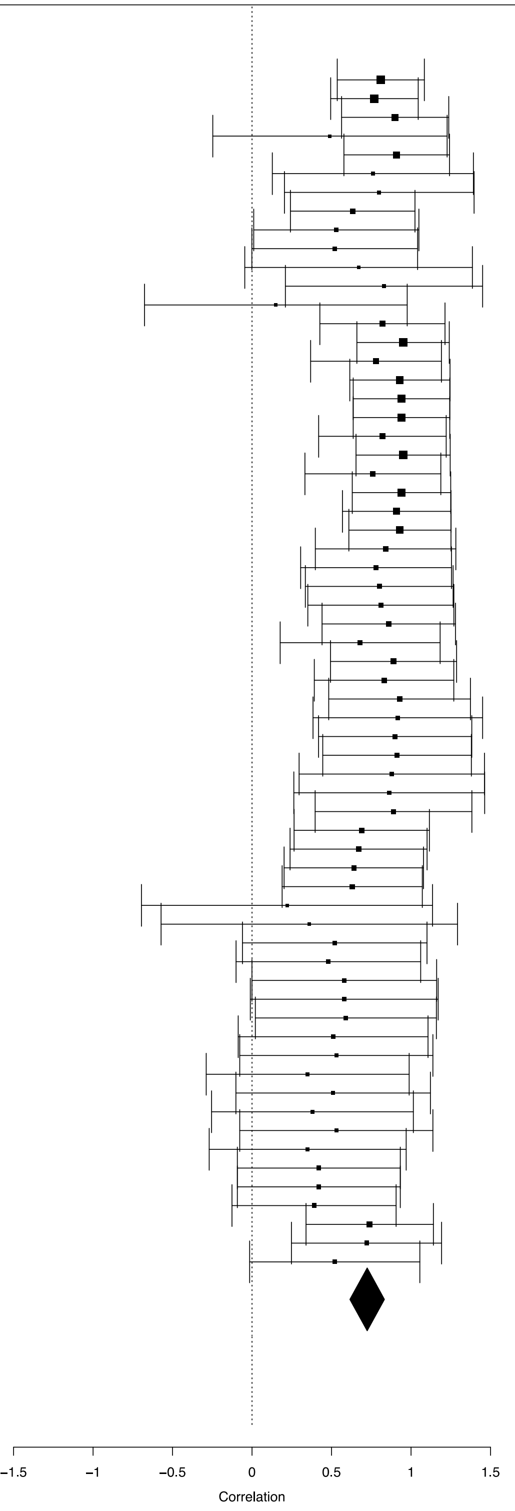

© 2022 Austerberry C et al. *JAMA Network Open.*

eFigure 35 (Continued)

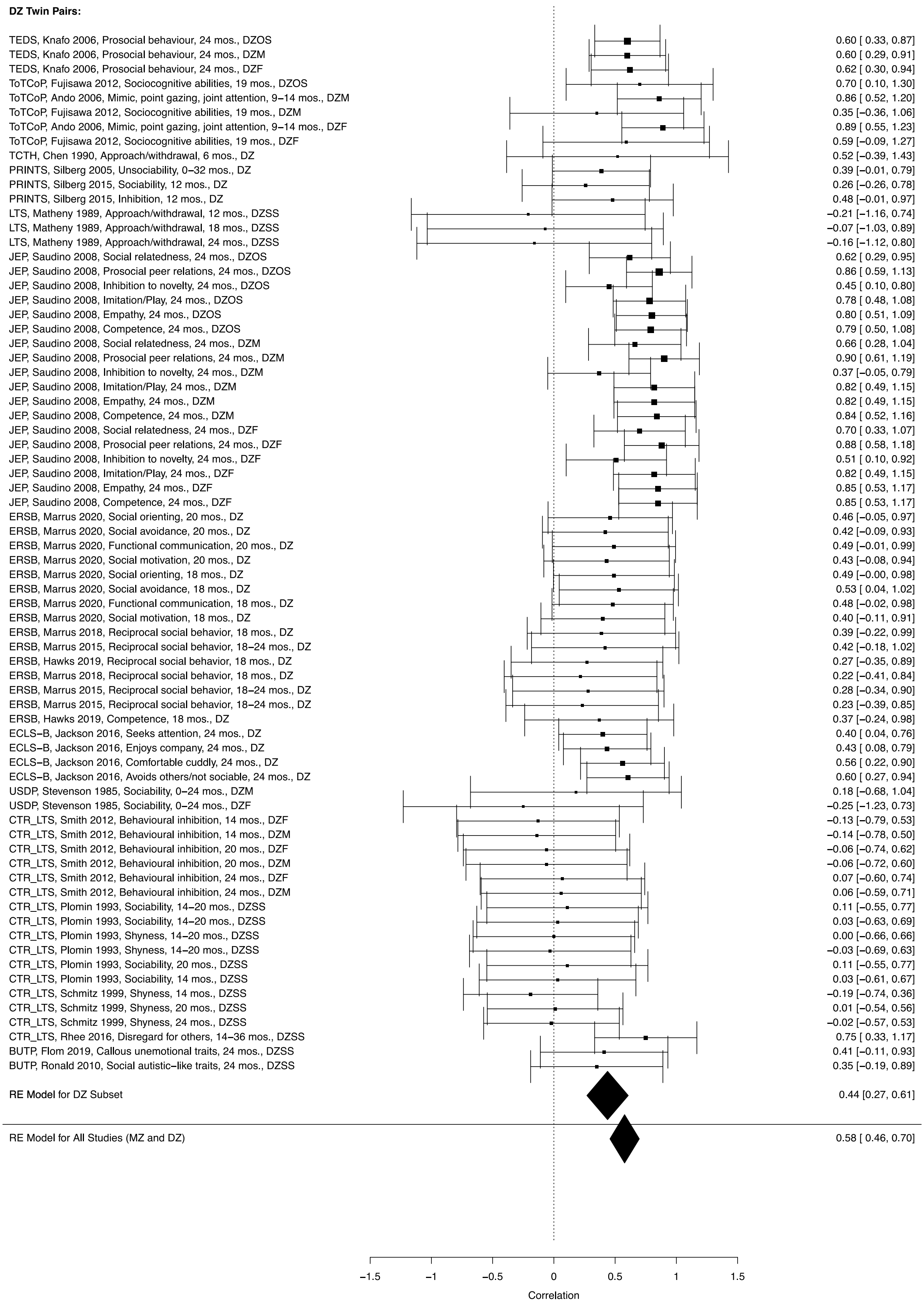

© 2022 Austerberry C et al. *JAMA Network Open.*

eReferences

1. Kmet LM, Lee RC, Cook LS. *Standard quality assessment criteria for evaluating primary research papers from a variety of fields.* Edmonton: Alberta Heritage Foundation for Medical Research (AHFMR);2004.
2. Falconer DS. *Introduction to quantitative genetics.* New York: Ronald Press Co; 1960.
3. Van den Noortgate W, Lopez-Lopez JA, Marin-Martinez F, Sanchez-Meca J. Three-level meta-analysis of dependent effect sizes. *Behavior Research Methods.* 2013;45(2):576–594.
4. World Health Organization. *International Classification of Functioning, Disability, and Health: Children & Youth Version: ICF-CY.* World Health Organization; 2007.
5. Page MJ, Sterne JAC, Higgins JPT, Egger M. Investigating and dealing with publication bias and other reporting biases in meta-analyses of health research: A review. *Research Synthesis Methods.* 2021;12(2):248-259.
6. Akerman BA, Fischbein S. Within-pair similarity in MZ and DZ twins from birth to eighteen years of age. *Acta Genet Med Gemellol (Roma).* 1992;41(2-3):155-164.
7. Ando J, Nonaka K, Ozaki K, et al. The Tokyo Twin Cohort Project: overview and initial findings. *Twin Res Hum Genet.* 2006;9(6):817-826.
8. Bakermans-Kranenburg MJ, van Uzendoorn MH, Bokhorst CL, Schuengel C. The importance of shared environment in infant-father attachment: a behavioral genetic study of the attachment q-sort. *J Fam Psychol.* 2004;18(3):545-549.
9. Beaver KM, Boutwell BB, Barnes J, Schwartz JA, Connolly EJ. A quantitative genetic analysis of the associations among language skills, peer interactions, and behavioral problems in childhood: Results from a sample of twins. *Merrill-Palmer Quarterly.* 2014;60(2):142-167.
10. Bishop E, Cherny SS, Corley R, Plomin R, DeFries JC, Hewitt JK. Development genetic analysis of general cognitive ability from 1 to 12 years in a sample of adoptees, biological siblings, and twins. *Intelligence.* 2003;31(1):31-49.
11. Bokhorst CL, Bakermans-Kranenburg MJ, Fearon RM, van IMH, Fonagy P, Schuengel C. The importance of shared environment in mother-infant attachment security: a behavioral genetic study. *Child Dev.* 2003;74(6):1769-1782.
12. Boomsma DI, Orlebeke JF, van Baal GC. The Dutch Twin Register: growth data on weight and height. *Behav Genet.* 1992;22(2):247-251.
13. Brant AM, Haberstick BC, Corley RP, Wadsworth SJ, DeFries JC, Hewitt JK. The developmental etiology of high IQ. *Behav Genet.* 2009;39(4):393-405.
14. Brescianini S, Volzone A, Fagnani C, et al. Genetic and environmental factors shape infant sleep patterns: a study of 18-month-old twins. *Pediatrics.* 2011;127(5):e1296-1302.
15. Brescianini S, Giampietro S, Cotichini R, Lucchini R, De Curtis M. Genetic and environmental components of neonatal weight gain in preterm infants. *Pediatrics.* 2012;129(2):e455-459.
16. Caramaschi D, Booij L, Petitclerc A, Boivin M, Tremblay RE. Genetic and environmental contributions to saliva testosterone levels in male and female infant twins. *Psychoneuroendocrinology.* 2012;37(12):1954-1959.
17. Chen CJ, Yu MW, Wang CJ, et al. Genetic variance and heritability of temperament among Chinese twin infants. *Acta Genet Med Gemellol (Roma).* 1990;39(4):485-490.
18. Chen CJ, Yu MW, Wang CJ, et al. Chronological changes in genetic variance and heritability of anthropometric characteristics among Chinese twin infants. *Acta Genet Med Gemellol (Roma).* 1990;39(4):479-484.
19. Cherny S, Cardon L, Fulker DW, DeFries J. Differential heritability across levels of cognitive ability. *Behavior Genetics.* 1992;22(2):153-162.
20. Cherny SS, Fulker DW, Corley RP, Plomin R, DeFries JC. Continuity and change in infant shyness from 14 to 20 months. *Behav Genet.* 1994;24(4):365-379.
21. Cherny SS, Fulker D, Emde R, et al. A developmental-genetic analysis of continuity and change in the Bayley Mental Development Index from 14 to 24 months: The MacArthur Longitudinal Twin Study. *Psychological Science.* 1994;5(6):354-360.

© 2022 Austerberry C et al. *JAMA Network Open.*

22. Custodio RJ, Junior CE, Milani SL, Simoes AL, de Castro M, Moreira AC. The emergence of the cortisol circadian rhythm in monozygotic and dizygotic twin infants: the twin-pair synchrony. *Clin Endocrinol (Oxf).* 2007;66(2):192-197.
23. Dale PS, Dionne G, Eley TC, Plomin R. Lexical and grammatical development: a behavioural genetic perspective. *J Child Lang.* 2000;27(3):619-642.
24. Davis DW, Finkel D, Turkheimer E, Dickens W. Genetic and Environmental Contributions to Behavioral Stability and Change in Children 6-36 months of Age Using Louisville Twin Study Data. *Behav Genet.* 2015;45(6):610-621.
25. DiLalla LF, Bishop EG. Differential maternal treatment of infant twins: effects of infant behaviors. *Behav Genet.* 1996;26(6):535-542.
26. DiLalla LF, Kagan J, Reznick JS. Genetic etiology of behavioral inhibition among 2-year-old children. *Infant Behavior and Development.* 1994;17:405-412.
27. Dionne G, Dale PS, Boivin M, Plomin R. Genetic evidence for bidirectional effects of early lexical and grammatical development. *Child Dev.* 2003;74(2):394-412.
28. Dionne G, Tremblay R, Boivin M, Laplante D, Perusse D. Physical aggression and expressive vocabulary in 19-month-old twins. *Dev Psychol.* 2003;39(2):261-273.
29. Dionne G, Touchette E, Forget-Dubois N, et al. Associations between sleep-wake consolidation and language development in early childhood: a longitudinal twin study. *Sleep.* 2011;34(8):987-995.
30. Dubois L, Girard M, Girard A, Tremblay R, Boivin M, Perusse D. Genetic and environmental influences on body size in early childhood: a twin birth-cohort study. *Twin Res Hum Genet.* 2007;10(3):479-485.
31. Dubois L, Ohm Kyvik K, Girard M, et al. Genetic and environmental contributions to weight, height, and BMI from birth to 19 years of age: an international study of over 12,000 twin pairs. *PLoS One.* 2012;7(2):e30153.
32. Emde RN, Plomin R, Robinson JA, et al. Temperament, emotion, and cognition at fourteen months: the MacArthur Longitudinal Twin Study. *Child Dev.* 1992;63(6):1437-1455.
33. Finkel D, Matheny AP, Jr. Genetic and environmental influences on a measure of infant attachment security. *Twin Res.* 2000;3(4):242-250.
34. Fisher A, van Jaarsveld CH, Llewellyn CH, Wardle J. Genetic and environmental influences on infant sleep. *Pediatrics.* 2012;129(6):1091-1096.
35. Flom M, Saudino KJ. Callous-unemotional behaviors in early childhood: Genetic and environmental contributions to stability and change. *Dev Psychopathol.* 2017;29(4):1227-1234.
36. Flom M, Saudino KJ. Do Genetic Factors Explain the Links Between Callous-Unemotional, Attention Hyperactivity and Oppositional Defiant Problems in Toddlers? *J Abnorm Child Psychol.* 2018;46(6):1217-1228.
37. Flom M, White D, Ganiban J, Saudino KJ. Longitudinal Links Between Callous-Unemotional Behaviors and Parenting in Early Childhood: A Genetically Informed Design. *J Am Acad Child Adolesc Psychiatry.* 2019.
38. Forget-Dubois N, Boivin M, Dionne G, Pierce T, Tremblay RE, Perusse D. A longitudinal twin study of the genetic and environmental etiology of maternal hostile-reactive behavior during infancy and toddlerhood. *Infant Behav Dev.* 2007;30(3):453-465.
39. Friedman NP, Miyake A, Robinson JL, Hewitt JK. Developmental trajectories in toddlers' self-restraint predict individual differences in executive functions 14 years later: a behavioral genetic analysis. *Dev Psychol.* 2011;47(5):1410-1430.
40. Fujisawa KK, Ozaki K, Suzuki K, Yamagata S, Kawahashi I, Ando J. Genetic and environmental relationships between head circumference growth in the first year of life and sociocognitive development in the second year: a longitudinal twin study. *Dev Sci.* 2012;15(1):99-112.
41. Gagne JR, Goldsmith HH. A longitudinal analysis of anger and inhibitory control in twins from 12 to 36 months of age. *Dev Sci.* 2011;14(1):112-124.
42. Gagne JR, Saudino KJ. Wait for it! A twin study of inhibitory control in early childhood. *Behav Genet.* 2010;40(3):327-337.

43. Gagne JR, Saudino KJ, Asherson P. The genetic etiology of inhibitory control and behavior problems at 24 months of age. *J Child Psychol Psychiatry.* 2011;52(11):1155-1163.
44. Gagne JR, Saudino KJ. The development of inhibitory control in early childhood: A twin study from 2-3 years. *Dev Psychol.* 2016;52(3):391-399.
45. Gagne JR, Asherson P, Saudino KJ. A Twin Study of Inhibitory Control at Age Two and ADHD Behavior Problems at Age Three. *Behav Genet.* 2020;50(4):289-300.
46. Galsworthy MJ, Dionne G, Dale PS, Plomin R. Sex differences in early verbal and non-verbal cognitive development. *Developmental Science.* 2000;3(2):206-215.
47. German A, Livshits G, Peter I, et al. Environmental rather than genetic factors determine the variation in the age of the infancy to childhood transition: a twins study. *J Pediatr.* 2015;166(3):731-735.
48. Gilmore JH, Schmitt JE, Knickmeyer RC, et al. Genetic and environmental contributions to neonatal brain structure: A twin study. *Hum Brain Mapp.* 2010;31(8):1174-1182.
49. Goetghebuer T, Ota MO, Kebbeh B, et al. Delay in motor development of twins in Africa: a prospective cohort study. *Twin Res.* 2003;6(4):279-284.
50. Goldsmith HH, Gottesman, II. Origins of variation in behavioral style: a longitudinal study of temperament in young twins. *Child Dev.* 1981;52(1):91-103.
51. Goldsmith HH, Lemery KS, Buss KA, Campos JJ. Genetic analyses of focal aspects of infant temperament. *Dev Psychol.* 1999;35(4):972-985.
52. Hawks ZW, Marrus N, Glowinski AL, Constantino JN. Early Origins of Autism Comorbidity: Neuropsychiatric Traits Correlated in Childhood Are Independent in Infancy. *J Abnorm Child Psychol.* 2018.
53. Herle M, Fildes A, Llewellyn C. Emotional eating is learned not inherited in children, regardless of obesity risk. *Pediatric Obesity.* 2018;13(10):628-631.
54. Hur YM. Genetic and environmental influences on birthweight in a sample of Korean twins. *J Korean Med Sci.* 2005;20(3):355-360.
55. Hur YM, Luciano M, Martin NG, et al. A comparison of twin birthweight data from Australia, the Netherlands, the United States, Japan, and South Korea: are genetic and environmental variations in birthweight similar in Caucasians and East Asians? *Twin Res Hum Genet.* 2005;8(6):638-648.
56. Ilott N, Saudino KJ, Wood A, Asherson P. A genetic study of ADHD and activity level in infancy. *Genes Brain Behav.* 2010;9(3):296-304.
57. Ilott NE, Saudino KJ, Asherson P. Genetic influences on attention deficit hyperactivity disorder symptoms from age 2 to 3: a quantitative and molecular genetic investigation. *BMC Psychiatry.* 2010;10:102.
58. Jackson DB. The Association Between Breastfeeding Duration and Attachment: A Genetically Informed Analysis. *Breastfeed Med.* 2016.
59. Jha SC, Xia K, Schmitt JE, et al. Genetic influences on neonatal cortical thickness and surface area. *Hum Brain Mapp.* 2018;39(12):4998-5013.
60. Johnson L, Llewellyn CH, van Jaarsveld CH, Cole TJ, Wardle J. Genetic and environmental influences on infant growth: prospective analysis of the Gemini twin birth cohort. *PLoS One.* 2011;6(5):e19918.
61. Knafo A, Plomin R. Prosocial behavior from early to middle childhood: genetic and environmental influences on stability and change. *Dev Psychol.* 2006;42(5):771-786.
62. Koeppen-Schomerus G, Spinath FM, Plomin R. Twins and non-twin siblings: different estimates of shared environmental influence in early childhood. *Twin Res.* 2003;6(2):97-105.
63. Kuntsi J, Rijsdijk F, Ronald A, Asherson P, Plomin R. Genetic influences on the stability of attention-deficit/hyperactivity disorder symptoms from early to middle childhood. *Biol Psychiatry.* 2005;57(6):647-654.
64. Lacourse E, Boivin M, Brendgen M, et al. A longitudinal twin study of physical aggression during early childhood: evidence for a developmentally dynamic genome. *Psychol Med.* 2014;44(12):2617-2627.
65. Levine RS, Hennekens CH, Jesse MJ. Genetic variance of weight and length in infant twins. *Am J Epidemiol.* 1987;126(5):929-935.

66. Liu Q, Yu C, Gao W, et al. Genetic and Environmental Effects on Weight, Height, and BMI Under 18 Years in a Chinese Population-Based Twin Sample. *Twin Res Hum Genet.* 2015;18(5):571-580.
67. Livshits G, Peter I, Vainder M, Hauspie R. Genetic analysis of growth curve parameters of body weight, height and head circumference. *Ann Hum Biol.* 2000;27(3):299-312.
68. Llewellyn CH, van Jaarsveld CH, Johnson L, Carnell S, Wardle J. Nature and nurture in infant appetite: analysis of the Gemini twin birth cohort. *Am J Clin Nutr.* 2010;91(5):1172-1179.
69. Marrus N, Glowinski AL, Jacob T, et al. Rapid video-referenced ratings of reciprocal social behavior in toddlers: a twin study. *J Child Psychol Psychiatry.* 2015;56(12):1338-1346.
70. Marrus N, Kennon-McGill S, Harris B, Zhang Y, Glowinski AL, Constantino JN. Use of a Video Scoring Anchor for Rapid Serial Assessment of Social Communication in Toddlers. *J Vis Exp.* 2018(133).
71. Marrus N, Grant JD, Harris-Olenak B, et al. Genetic architecture of reciprocal social behavior in toddlers: Implications for heterogeneity in the early origins of autism spectrum disorder. *Dev Psychopathol.* 2020;32(4):1190-1205.
72. Matheny AP, Jr. Bayley's infant behavior record: behavioral components and twin analyses. *Child Dev.* 1980;51(4):1157-1167.
73. Matheny AP. A longitudinal twin study of stability of components from Bayley's Infant Behavior Record. *Child Development.* 1983;54:356-360.
74. Matheny AP, Jr. Twin similarity in the developmental transformations of infant temperament as measured in a multi-method, longitudinal study. *Acta Genet Med Gemellol (Roma).* 1984;33(2):181-189.
75. Matheny AP, Jr. Children's behavioral inhibition over age and across situations: genetic similarity for a trait during change. *J Pers.* 1989;57(2):215-235.
76. Matheny AP, Jr., Dolan AB, Wilson RS. Twins: within-pair similarity on Bayley's Infant Behavior Record. *J Genet Psychol.* 1976;128(2d Half):263-270.
77. Micalizzi L, Ronald A, Saudino KJ. A Genetically Informed Cross-Lagged Analysis of Autistic-Like Traits and Affective Problems in Early Childhood. *J Abnorm Child Psychol.* 2016;44(5):937-947.
78. Micalizzi L, Wang M, Saudino KJ. Difficult temperament and negative parenting in early childhood: a genetically informed cross-lagged analysis. *Dev Sci.* 2017;20(2).
79. Mook-Kanamori DO, van Beijsterveldt CE, Steegers EA, et al. Heritability estimates of body size in fetal life and early childhood. *PLoS One.* 2012;7(7):e39901.
80. Nguyen BH, Perusse D, Paquet J, et al. Sleep terrors in children: a prospective study of twins. *Pediatrics.* 2008;122(6):e1164-1167.
81. Nichols PL, Broman SH. Familial resemblance in infant mental development. *Developmental Psychology.* 1974;10(3):442-446.
82. Orekhova EV, Stroganova TA, Posikera IN, Malykh SB. Heritability and "environmentability" of electroencephalogram in infants: the twin study. *Psychophysiology.* 2003;40(5):727-741.
83. Ouellet-Morin I, Boivin M, Dionne G, et al. Variations in heritability of cortisol reactivity to stress as a function of early familial adversity among 19-month-old twins. *Arch Gen Psychiatry.* 2008;65(2):211-218.
84. Ouellet-Morin I, Dionne G, Perusse D, et al. Daytime cortisol secretion in 6-month-old twins: genetic and environmental contributions as a function of early familial adversity. *Biol Psychiatry.* 2009;65(5):409-416.
85. Peter I, Vainder M, Livshits G. Genetic analysis of motor milestones attainment in early childhood. *Twin Res.* 1999;2(1):1-9.
86. Petitclerc A, Boivin M, Dionne G, Perusse D, Tremblay RE. Genetic and environmental etiology of disregard for rules. *Behav Genet.* 2011;41(2):192-200.
87. Pimpin L, Ambrosini GL, Llewellyn CH, et al. Dietary intake of young twins: nature or nurture? *Am J Clin Nutr.* 2013;98(5):1326-1334.
88. Planalp EM, Van Hulle C, Lemery-Chalfant K, Goldsmith HH. Genetic and environmental contributions to the development of positive affect in infancy. *Emotion.* 2017;17(3):412-420.

89. Plomin R, Rowe DC. Genetic and Environmental Etiology of Social Behavior in Infancy. *Developmental Psychology.* 1979;15(1):62-72.
90. Plomin R, Emde RN, Braungart JM, et al. Genetic change and continuity from fourteen to twenty months: the MacArthur Longitudinal Twin Study. *Child Dev.* 1993;64(5):1354-1376.
91. Price TS, Eley TC, Dale PS, Stevenson J, Saudino K, Plomin R. Genetic and environmental covariation between verbal and nonverbal cognitive development in infancy. *Child Dev.* 2000;71(4):948-959.
92. Price TS, Simonoff E, Asherson P, et al. Continuity and change in preschool ADHD symptoms: longitudinal genetic analysis with contrast effects. *Behav Genet.* 2005;35(2):121-132.
93. Pushina NN, Orekhova EV, Stroganova TA. Age-related and individual differences in the performance of a delayed response task (the A-not-B task) in infant twins aged 7-12 months. *Neurosci Behav Physiol.* 2005;35(5):481-490.
94. Reznick JS, Corley R, Robinson J. A longitudinal twin study of intelligence in the second year. *Monogr Soc Res Child Dev.* 1997;62(1):i-vi, 1-154; discussion 155-160.
95. Rhee SH, Cosgrove VE, Schmitz S, Haberstick BC, Corley RC, Hewitt JK. Early childhood temperament and the covariation between internalizing and externalizing behavior in school-aged children. *Twin Res Hum Genet.* 2007;10(1):33-44.
96. Rhee SH, Corley RP, Friedman NP, et al. The etiology of observed negative emotionality from 14 to 24 months. *Front Genet.* 2012;3:9.
97. Rhee SH, Friedman NP, Boeldt DL, et al. Early concern and disregard for others as predictors of antisocial behavior. *J Child Psychol Psychiatry.* 2013;54(2):157-166.
98. Rhee SH, Friedman NP, Corley RP, et al. An examination of the developmental propensity model of conduct problems. *J Abnorm Psychol.* 2016;125(4):550-564.
99. Rice ML, Zubrick SR, Taylor CL, Gayan J, Bontempo DE. Late language emergence in 24-month-old twins: heritable and increased risk for late language emergence in twins. *J Speech Lang Hear Res.* 2014;57(3):917-928.
100. Riese ML. Genetic influences on neonatal temperament. *Acta Genet Med Gemellol (Roma).* 1990;39(2):207-213.
101. Riese ML. Neonatal temperament in monozygotic and dizygotic twin pairs. *Child Dev.* 1990;61(4):1230-1237.
102. Robinson JL, Kagan J, Reznick JS, Corley R. The heritability of inhibited and uninhibited behavior: A twin study. *Developmental Psychology.* 1992;28(6):1030-1037.
103. Roisman GI, Fraley RC. The limits of genetic influence: a behavior-genetic analysis of infant-caregiver relationship quality and temperament. *Child Dev.* 2006;77(6):1656-1667.
104. Roisman GI, Fraley RC. A behavior-genetic study of parenting quality, infant attachment security, and their covariation in a nationally representative sample. *Dev Psychol.* 2008;44(3):831-839.
105. Ronald A, Edelson LR, Asherson P, Saudino KJ. Exploring the relationship between autistic-like traits and ADHD behaviors in early childhood: findings from a community twin study of 2-year-olds. *J Abnorm Child Psychol.* 2010;38(2):185-196.
106. Saudino KJ. Sources of continuity and change in activity level in early childhood. *Child Dev.* 2012;83(1):266-281.
107. Saudino KJ, Zapfe JA. Genetic influences on activity level in early childhood: do situations matter? *Child Dev.* 2008;79(4):930-943.
108. Saudino KJ, Eaton WO. Infant temperament and genetics: an objective twin study of motor activity level. *Child Dev.* 1991;62(5):1167-1174.
109. Saudino KJ, Plomin R, DeFries JC. Tester-rated temperament at 14, 20 and 24 months: Environmental change and genetic continuity. *British Journal of Developmental Psychology.* 1996;14(2):129-144.
110. Saudino KJ, Carter AS, Purper-Ouakil D, Gorwood P. The etiology of behavioral problems and competencies in very young twins. *J Abnorm Psychol.* 2008;117(1):48-62.
111. Saudino KJ, Wang M, Flom M, Asherson P. Genetic and environmental links between motor activity level and attention problems in early childhood. *Dev Sci.* 2018;21(5):e12630.

© 2022 Austerberry C et al. *JAMA Network Open.*

112. Schmitz S, Fulker DW, Plomin R, Zahn-Waxler C, Emde RN, DeFries JC. Temperament and problem behavior during early childhood. *International Journal of Behavioral Development.* 1999;23(2):333-355.
113. Schumann L, Boivin M, Paquin S, et al. Persistence and innovation effects in genetic and environmental factors in negative emotionality during infancy: A twin study. *PLoS One.* 2017;12(4):e0176601.
114. Silberg JL, Miguel VF, Murrelle EL, et al. Genetic and environmental influences on temperament in the first year of life: the Puerto Rico Infant Twin Study (PRINTS). *Twin Res Hum Genet.* 2005;8(4):328-336.
115. Silberg JL, Gillespie N, Moore AA, et al. Shared genetic and environmental influences on early temperament and preschool psychiatric disorders in Hispanic twins. *Twin Res Hum Genet.* 2015;18(2):171-178.
116. Silventoinen K, Pietilainen KH, Tynelius P, Sorensen TI, Kaprio J, Rasmussen F. Genetic and environmental factors in relative weight from birth to age 18: the Swedish young male twins study. *Int J Obes (Lond).* 2007;31(4):615-621.
117. Silventoinen K, Pietilainen KH, Tynelius P, Sorensen TI, Kaprio J, Rasmussen F. Genetic regulation of growth from birth to 18 years of age: the Swedish young male twins study. *Am J Hum Biol.* 2008;20(3):292-298.
118. Silventoinen K, Karvonen M, Sugimoto M, Kaprio J, Dunkel L, Yokoyama Y. Genetics of head circumference in infancy: a longitudinal study of Japanese twins. *Am J Hum Biol.* 2011;23(5):630-634.
119. Silventoinen K, Kaprio J, Yokoyama Y. Genetics of pre-pubertal growth: a longitudinal study of Japanese twins. *Ann Hum Biol.* 2011;38(5):608-614.
120. Silventoinen K, Kaprio J, Dunkel L, Yokoyama Y. Genetic and environmental influences on chest circumference during infancy: a longitudinal study of japanese twins. *Paediatr Perinat Epidemiol.* 2012;26(6):553-560.
121. Silventoinen K, Jelenkovic A, Sund R, et al. Genetic and environmental effects on body mass index from infancy to the onset of adulthood: an individual-based pooled analysis of 45 twin cohorts participating in the COllaborative project of Development of Anthropometrical measures in Twins (CODATwins) study. *Am J Clin Nutr.* 2016;104(2):371-379.
122. Smit DJ, Luciano M, Bartels M, et al. Heritability of head size in Dutch and Australian twin families at ages 0-50 years. *Twin Res Hum Genet.* 2010;13(4):370-380.
123. Smith AK, Rhee SH, Corley RP, Friedman NP, Hewitt JK, Robinson JL. The magnitude of genetic and environmental influences on parental and observational measures of behavioral inhibition and shyness in toddlerhood. *Behav Genet.* 2012;42(5):764-777.
124. Smith AD, Herle M, Fildes A, Cooke L, Steinsbekk S, Llewellyn CH. Food fussiness and food neophobia share a common etiology in early childhood. *J Child Psychol Psychiatry.* 2017;58(2):189-196.
125. Smith L, van Jaarsveld CHM, Llewellyn CH, et al. Genetic and Environmental Influences on Developmental Milestones and Movement: Results From the Gemini Cohort Study. *Res Q Exerc Sport.* 2017;88(4):401-407.
126. Soussignan R, Boivin M, Girard A, Perusse D, Liu X, Tremblay RE. Genetic and environmental etiology of emotional and social behaviors in 5-month-old infant twins: influence of the social context. *Infant Behav Dev.* 2009;32(1):1-9.
127. Spinath FM, Ronald A, Harlaar N, Price TS, Plomin R. Phenotypic g early in life: On the etiology of general cognitive ability in a large population sample of twin children aged 2-4 years. *Intelligence.* 2003;31(2):195-210.
128. Stevenson J, Fielding J. Ratings of temperament in families of young twins. *British Journal of Developmental Psychology.* 1985;3:143–152.
129. Stroganova T, Tsetlin M, Malykh S, Malakhovskaya E. Biological principles of individual differences of children of the second half-year of life: Communication II. The nature of individual differences in temperamental features. *Human Physiology.* 2000;26(3):281-289.
130. Touchette E, Dionne G, Forget-Dubois N, et al. Genetic and environmental influences on daytime and nighttime sleep duration in early childhood. *Pediatrics.* 2013;131(6):e1874-1880.

131. Touwslager RN, Gielen M, Derom C, et al. Determinants of infant growth in four age windows: a twin study. *J Pediatr.* 2011;158(4):566-572.e562.
132. Touwslager RN, Gielen M, Mulder AL, et al. Changes in genetic and environmental effects on growth during infancy. *Am J Clin Nutr.* 2011;94(6):1568-1574.
133. Tucker-Drob EM, Rhemtulla M, Harden KP, Turkheimer E, Fask D. Emergence of a Gene x socioeconomic status interaction on infant mental ability between 10 months and 2 years. *Psychol Sci.* 2011;22(1):125-133.
134. van Dommelen P, de Gunst MC, van der Vaart AW, Boomsma DI. Genetic study of the height and weight process during infancy. *Twin Res.* 2004;7(6):607-616.
135. Wang M, Saudino KJ. Genetic and environmental contributions to stability and change of sleep problems in toddlerhood. *J Pediatr Psychol.* 2012;37(6):697-706.
136. Whitfield JB, Treloar SA, Zhu G, Martin NG. Genetic and non-genetic factors affecting birthweight and adult body mass index. *Twin Res* 2001;4:365-370.
137. Wilson RS. Twins: early mental development. *Science.* 1972;175(4024):914-917.
138. Wilson RS. Twins: Mental development in the preschool years. *Developmental Psychology.* 1974;10(4):580-588.
139. Wilson RS. Synchronies in mental development: an epigenetic perspective. *Science.* 1978;202(4371):939-948.
140. Wilson RS. The Louisville Twin Study: developmental synchronies in behavior. *Child Dev.* 1983;54(2):298-316.
141. Wilson RS. Twins and chronogenetics: correlated pathways of development. *Acta Genet Med Gemellol (Roma).* 1984;33(2):149-157.
142. Wilson RS, Harpring EB. Mental and motor development in infant twins. *Developmental Psychology.* 1972;7(3):277-287.
143. Wilson RS, Matheny AP, Jr. Retardation and twin concordance in infant mental development: a reassessment. *Behav Genet.* 1976;6(3):353-358.
144. Woodward KE, Boeldt DL, Corley RP, et al. Correlates of Positive Parenting Behaviors. *Behav Genet.* 2018.

© 2022 Austerberry C et al. *JAMA Network Open.*
